# The Fishery Performance Indicators: A Management Tool for Triple Bottom Line Outcomes

**DOI:** 10.1371/journal.pone.0122809

**Published:** 2015-05-06

**Authors:** James L. Anderson, Christopher M. Anderson, Jingjie Chu, Jennifer Meredith, Frank Asche, Gil Sylvia, Martin D. Smith, Dessy Anggraeni, Robert Arthur, Atle Guttormsen, Jessica K. McCluney, Tim Ward, Wisdom Akpalu, Håkan Eggert, Jimely Flores, Matthew A. Freeman, Daniel S. Holland, Gunnar Knapp, Mimako Kobayashi, Sherry Larkin, Kari MacLauchlin, Kurt Schnier, Mark Soboil, Sigbjorn Tveteras, Hirotsugu Uchida, Diego Valderrama

**Affiliations:** 1 Institute for Global Food Systems, University of Florida, PO Box 110240, Gainesville, FL 32611, United States of America; 2 School of Aquatic and Fishery Sciences, University of Washington, Box 355020, Seattle, WA 98105, United States of America; 3 The World Bank, 1818 H Street NW, Washington, DC 20433, United States of America; 4 Department of Economics, University of Washington, Box 353330, Seattle, WA 98195, United States of America; 5 Department of Industrial Economics, University of Stavanger, Stavanger 4036, Norway; 6 Coastal Oregon Marine Experiment Station, Marine Resource Economics, Oregon State University, Hatfield Marine Science Center, 2030 Marine Science Drive, Newport, OR 97365, United States of America; 7 Nicholas School of the Environment, Duke University, Box 90328, Durham, NC 27708, United States of America; 8 Sustainable Fisheries Partnership, JL. Palem Putri IX/NO. 1, Taman Yasmin V, Bogor 16112, Indonesia; 9 MRAG Ltd., 18 Queen Street, London W1J 5PN, United Kingdom; 10 Department of Economics and Resource Management, Norwegian University of Life Sciences, Aas 1432, Norway; 11 South Australian Research and Development Institute (SARDI)—Aquatic Sciences, PO Box 120, Henley Beach, SA 5022, Australia; 12 United Nations University-World Institute for Development Economics Research, C/O Institute of Statistical, Social and Economic Research (ISSER), University of Ghana, P.O BOX LG 74, Legon, Ghana; 13 Department of Economics, University of Gothenburg, Gothenburg, Sweden; 14 Sustainable Fisheries Partnership, Block1, #5 El Rio Vista Phase 5, 8000 Davao City, Philippines; 15 Department of Agricultural Economics, PO Box 5187, Mississippi State University, Mississippi State, MS 39762, United States of America; 16 Conservation Biology Division, Northwest Fisheries Science Centre, National Marine Fisheries Service, National Oceanic and Atmospheric Administration, 2725 Montlake Blvd, Seattle, WA 98112, United States of America; 17 Institute of Social and Economic Research, University of Alaska Anchorage, 3211 Providence Drive, Anchorage, Alaska 99508, United States of America; 18 Environment and Natural Resource Management, The World Bank, 1818 H St. NW, Washington, DC 20433, United States of America; 19 Department of Food and Resource Economics, University of Florida, PO Box 110240, Gainesville, FL 32611, United States of America; 20 South Atlantic Fishery Management Council, 4055 Faber Place Dr., Suite 201, North Charleston, SC 29405, United States of America; 21 School of Social Sciences, Humanities and Arts, University of California Merced, 5200 North Lake Road, Merced, CA 95343, United States of America; 22 Marine Economic Development, Level 1 83–85 Victoria Rd, Devonport 0624, Aukland, New Zealand; 23 University of Stavanger, Stavanger 4036, Norway; 24 Department of Environmental & Natural Resource Economics, University of Rhode Island, 205 Kingston Coastal Institute, One Greenhouse Road, Kingston, RI 02881, United States of America; Hellenic Centre for Marine Research, GREECE

## Abstract

Pursuit of the triple bottom line of economic, community and ecological sustainability has increased the complexity of fishery management; fisheries assessments require new types of data and analysis to guide science-based policy in addition to traditional biological information and modeling. We introduce the Fishery Performance Indicators (FPIs), a broadly applicable and flexible tool for assessing performance in individual fisheries, and for establishing cross-sectional links between enabling conditions, management strategies and triple bottom line outcomes. Conceptually separating measures of performance, the FPIs use 68 individual outcome metrics—coded on a 1 to 5 scale based on expert assessment to facilitate application to data poor fisheries and sectors—that can be partitioned into sector-based or triple-bottom-line sustainability-based interpretative indicators. Variation among outcomes is explained with 54 similarly structured metrics of inputs, management approaches and enabling conditions. Using 61 initial fishery case studies drawn from industrial and developing countries around the world, we demonstrate the inferential importance of tracking economic and community outcomes, in addition to resource status.

## Introduction

The tragedy of the commons framework [1–3] has helped explain the decline in global fish stocks and indicated points of management intervention to facilitate stock rebuilding [4]. However, the continued struggles of fishing communities to leverage their resources into secure livelihoods [5] highlights a second, social tragedy of the commons. World fisheries fall short of their potential earnings by US$50–80 billion a year (e.g., [6–8]). Losses not only include foregone catch due to overfishing, but also excessive harvest cost, low processing yields, product waste, and a failure to reach the highest value markets [9]. The result is lost income to small-scale and industrial harvesters and processors, foregone high quality protein to consumers, and reduced food and income security for fishing dependent communities in both developed and developing regions.

Despite the need to understand how to best manage fisheries to capture these benefits, research on global fisheries performance emphasizes the effects of management primarily on stock and ecological conditions. While fisheries cannot be sustainable with degraded target stocks, high stock levels will not necessarily lead to an economically healthy industry that can support the community in which it resides. Even when social conditions are considered, emphasis is on those that are correlated with effective stock management [10] rather than on those that lead to desirable social and economic outcomes such as food security [11].

Resolving this social tragedy requires identifying whether and how fisheries are supporting the people who participate in them, and understanding how management influences human outcomes. Sustainable social-ecological systems require a sustainable resource stock, but also profitable businesses in the harvest and post-harvest sectors, and communities that accept and support those industries and the people involved in them [12–14]. Much as a sustainable stock is necessary, so too is social acceptability and continuous business investment.

Accordingly, leading fishery management and development organizations are integrating economic and community goals alongside stock health, and developing guiding principles that support the triple-bottom-line (TBL) [15,16]. In the US, the Magnuson-Stevens Fishery Conservation and Management Act balances National Standard 1 to “prevent overfishing” with National Standard 8’s mandate to provide for “sustained participation of communities” and “minimize adverse economic impacts” (16 US Code §1851). Similarly, the EU Common Fisheries Policy provides for “sustainable economic, environmental and social conditions” (CR 2371/2002 Article 2).

That more groups are articulating these three performance dimensions is a recognition that the sustainability of fish stocks, fishing industries, and fishing communities are interrelated, and that none can provide benefits without the others. The Rockefeller Foundation [17] concludes strategies focusing on “replenishment of fish stocks or conservation of marine biodiversity” have not supported “the success of the industry and as a critical link to poverty alleviation” in the long term, instead arguing for a “holistic approach” that incorporates economic and community outcomes. The Prince’s Charities note that “the fishing sector’s economic, environmental and social health can only be guaranteed if we view it in an holistic and integrated way” ([18] p. 4). The Blue Ribbon Panel [15] emphasizes that “a multidimensional indicator system …needs to be designed as an integral part of the measurement process.” Assessing progress toward these three dimensions of sustainability requires understanding the linkages within the social-ecological systems. Tracking and monitoring only ecosystem-related outcomes and performance is insufficient for understanding economic and community benefits. However, there is a lack of standard frameworks to measure outcomes on non-biological dimensions; instead, process implementation or adoption of community approaches to implementing ecological measures often serves as a proxy for advancing social goals (e.g., [19–21]).

Fishery science has gained considerable policy traction by analyzing the biological effects of alternative approaches to fishery management [4], including co-management (e.g., [10]), catch shares (e.g., [22,23]), individual transferable quotas (ITQs) (e.g., [24]), and marine protected areas (MPAs) (e.g., [25,26]). Many of these supporting studies have compiled existing data on catch volumes and stock health that are measured systematically across large numbers of fisheries around the world. These global analyses have influenced the agendas of regulatory agencies, non-governmental organizations (NGOs) and aid agencies, catalyzing policymakers to curtail overfishing. Adopting a similar approach for performance of the broader social-ecological system poses two significant challenges. First, economic and community factors are expressed through the political process of management. Social objectives are rarely explicit, vary widely by fishery [27], and differ by constituency within a fishery; often, there is no consensus regarding economic or community objectives analogous to preventing collapse or being at the biomass corresponding to maximum sustainable yield (B_MSY_) against which to evaluate social performance.

Second, there is a paucity of data on economic and community performance of individual fisheries. While most significant stocks—even in developing countries—have some form of landings data, basic economic data (e.g., ex vessel price) is tracked by regulators in far fewer fisheries, and supplemented by brokers only in widely traded commodity species. High quality harvesting cost data is rarely available. Data from the post-harvest sector—which generates considerable fishery-related employment, income and added value, key elements of economic and community performance—are almost nonexistent: processing costs and margins are typically considered proprietary, although they are derived from a public trust resource. Management expenditures are budgeted by agency, rather than by fishery. Metrics of community success are rarely collected outside short-term development investment projects, and even then are specifically tailored to the individual projects.

Despite these challenges, assessing progress on social-ecological outcomes presents an urgent need for new frameworks to evaluate how management approaches interact with resource, community and market conditions to not only assure stock health, but also create economic and community benefits. To meet these needs and challenges, we introduce the Fishery Performance Indicators (FPIs), a rapid assessment instrument for measuring the fishery-derived benefits being created not only in the fish stock in the water, but also in the harvest and post-harvest sectors and fishing communities. They are designed to provide insight into how management regimes interact with exogenous resource and community factors to affect whether and to whom benefits accrue.

## Methods

The FPIs were designed through an iterative, consultative process of extensive piloting and revision. The lead authors developed an initial instrument capturing the key objectives within a novel structure. With three initial case studies, the draft was reviewed at a two-day workshop hosted by The International Coalition of Fisheries Associations (ICFA) and The UK Department for International Development (DFID), with a panel of development agents and consultants, NGO representatives, academic economists, and global fishing industry participants. Based on advice received at the workshop, the draft was significantly revised prior to on-the-ground developing fishery field tests by the authors. The subsequently modified version was piloted by the US Department of Agriculture (USDA) W2004 group of academic economists, each of whom independently executed a case study based on a (predominantly industrialized) fishery with which they were familiar. These case studies—and the authors’ experience developing them—were presented at a one-day workshop where further instrument revisions were recommended. The World Bank then piloted the instrument with local country consultants in Indonesia and Philippines, and suggested further improvements through a process that included internal and external peer reviews [28]. Finally, all past case studies were updated to the current version, and a broader network of case studies was collected through World Bank and other agency development projects, and through collaboration with additional authors. This process built confidence among the authors that the instrument was supporting consistent scoring across the current database of 61 case studies.

## The Structure of the FPIs

To address the lack of standardized, reliable data on the social and economic pillars of the TBL, the FPIs reflect three distinctive structural features. First, rather than attempting to measure a few indicators with high precision, for each indicator we identify several dimensions of greatest interest. For each dimension, we then use multiple metrics that capture important aspects of that dimension using a 1 to 5 scale that can be scored—imprecisely but accurately—based on expert assessment. The use of discrete bins provides accurate scores by allowing experts to characterize the metric as being within a broad range, which can be expressed with high confidence, even when precise underlying data is not available. Combining multiple metrics facilitates robust dimension scoring in the face of uneven availability of information, fishery expert certainty or consensus, or metric applicability to a given fishery. Second, we conceptually separate dimensions that directly reflect performance on the pillars of the TBL from those that assess enabling environmental conditions, or elements of process that are conjectured to support outcomes and are therefore often used as proxies for those outcomes. Third, we incorporate flexibility in the application of the tool by providing two interpretive partitionings of the metrics into sets of three indicators. This allows users to aggregate metrics, and weight each component indicator, to reflect their individual objectives and priorities.

In both interpretive partitionings, the FPIs are designed to evaluate the performance of fishery management systems. Therefore, the scope of the management system is the primary scale at which the fishery unit for analysis is defined. This approach contrasts importantly with ecological analyses that focus on individual fish stocks as units for assessment. Many management systems regulate harvesting activity on multiple stocks, and many stocks are concurrently pursued by multiple groups of harvesters acting under different management rules. Since the effect of the management system may vary systematically with harvest technologies and the structure of the supply chain, the definition of fishery for analysis may subdivide the management system along fleet, market or jurisdictional lines to capture this heterogeneity, on a case-by-case basis. It is at this level—not the country level or the stock level—at which fishery management interacts with the integrated socio-ecological system to produce TBL outcomes.

### Output Indicators

**Fig 1:**
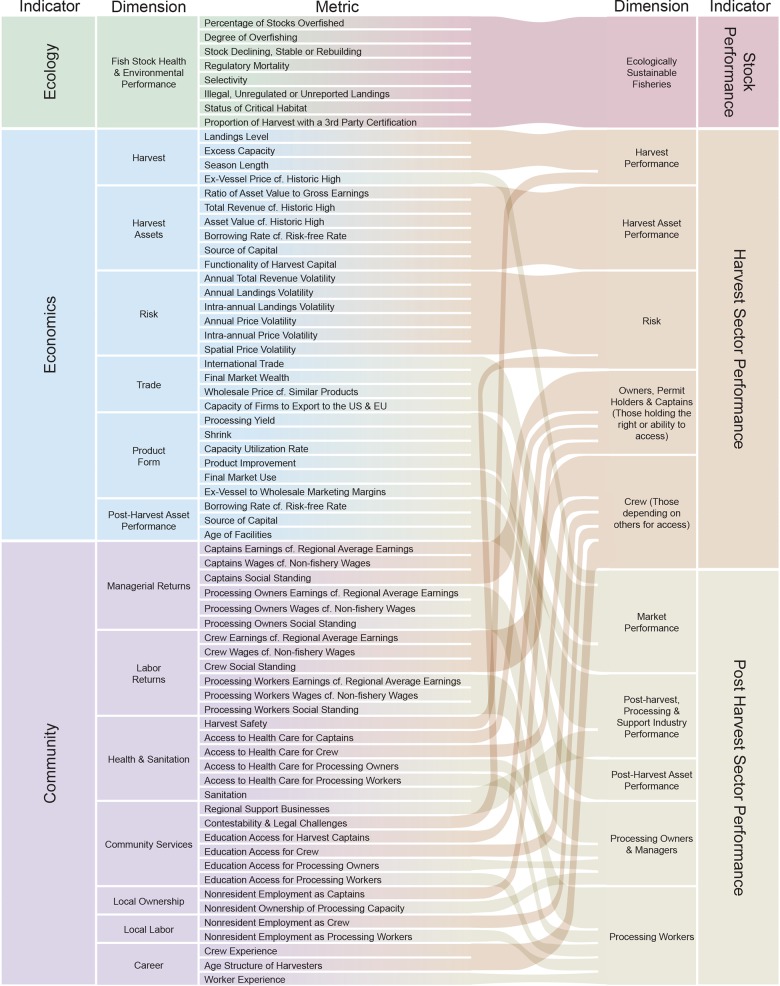
Schematic of the Output Indicators and the Associated Dimensions and Metrics.

Fig 1 shows how three indicators of the TBL are constructed using 68 metrics of fishery performance that are each coded in levels of 1 to 5, where 5 reflects better metric performance. The TBL partitioning, with indicators for performance on *Ecology*, *Economics* and *Community* outcomes, is represented on the far left. The *Ecology* indicator is captured in a single dimension composed of a relatively small number of metrics that summarize scientific assessment work, but with coding guidelines that facilitate accurate scoring based on the information available where formal stock assessments do not exist.

The *Economics* indicator measures whether the fishery is effectively generating market benefits and is reflected in six distinct dimensions. The *Harvest* dimension captures landings and revenue levels, and whether rent is dissipated through inefficiencies of excess capacity or derbies. Economic gains accumulate within the harvest sector through vessel profits and the value of permits, and to the post-harvest sector in the form of processing capital; these are captured in the *Harvest Assets* and *Post-Harvest Assets* dimensions. The *Risk* dimension captures several sources of volatility that affect business value. Finally, the *Trade* and *Product Form* dimensions assess the extent to which the product is reaching markets that yield the greatest potential profit.

The *Community* indicator captures the extent to which the fishery contributes to livelihoods and other benefits within its community. First, the *Managerial Returns* dimension assesses the fishery benefits accruing to boat captains, permit holders, and processing owners and managers; these earnings metrics are locally scaled, to place the livelihoods in the context of local opportunities and standards. The *Labor Returns* dimension similarly measures the scaled earnings, opportunity costs, and social status of harvesting crew and processing workers. Collectively, this reflects not only the relative level of income, but also the importance of the fishery to the community as part of its economy or culture, and the extent to which participants have sufficient voice within the community to ensure resources and support for effective management will sustain the fishery. By separately measuring capital owners and workers in each sector, the FPIs can identify to whom direct fishery wealth is accruing, and whether it is doing so in accordance with management goals [29]. Because labor earnings are measured relative to other occupations in the region available to those with comparable skill, cross-sectional variation in management can associate outcome differences between fishing and non-fishing sectors with features of the management system [30].

The *Health and Sanitation* dimension captures the health and safety environment of each group of stakeholders. A successful fishery will support the provision of benefits to the broader community, including education and other economic activity, captured in the *Community Support Services* dimension. The *Local Ownership* dimension scores the amount of harvesting and processing capital that is owned by nonresidents, while the *Local Labor* dimension measures the amount of employment on boats and in processing plants that is obtained by nonresidents, together capturing the extent to which fishery wealth stays within the community. Lastly, the *Career* dimension captures whether the fishery is generating stable, long-term employment for participants.

The individual metrics that comprise these indicators are regrouped in an alternative partitioning, represented on the far right of Fig 1, with indicators for *Stock Performance*, *Harvest Sector Performance* and *Post-Harvest Sector Performance*. This sector-based partitioning is useful for describing distributional outcomes and for potential investors in different segments of the fishery. The braiding shows how individual metrics are mapped into the dimensions of each indicator: in general, the *Harvest Sector Performance* and *Post-Harvest Sector Performance* indicators draw metrics from the *Community* and *Economics* indicators. The *Harvest Asset* and *Risk* dimensions map directly to *Harvest Sector Performance* dimensions, as does *Harvest* with the addition of the Safety metric. The *Owners*, *Permit Holders and Captains* dimension captures the earnings, community standing, and quality of life of those holding the capital needed to access to the fishery, while the *Crew* dimension represents the same information for people who work in harvesting but depend on others for access.

The *Post-Harvest Sector Performance* indicator dimensions map *Trade* and *Product Form* to *Market Performance* and *Post-Harvest Asset Performance*, respectively, *and Post-Harvest Assets* into *Post-Harvest Processing & Support Industry Performance* with the addition of the Support Industry metric. The *Processing Owners and Managers* dimension captures the earnings, community standing, and quality of life of those making processing decisions, while the *Processing Workers* dimension represents the same information for people employed in processing facilities.

### Enabling Factors

**Fig 2:**
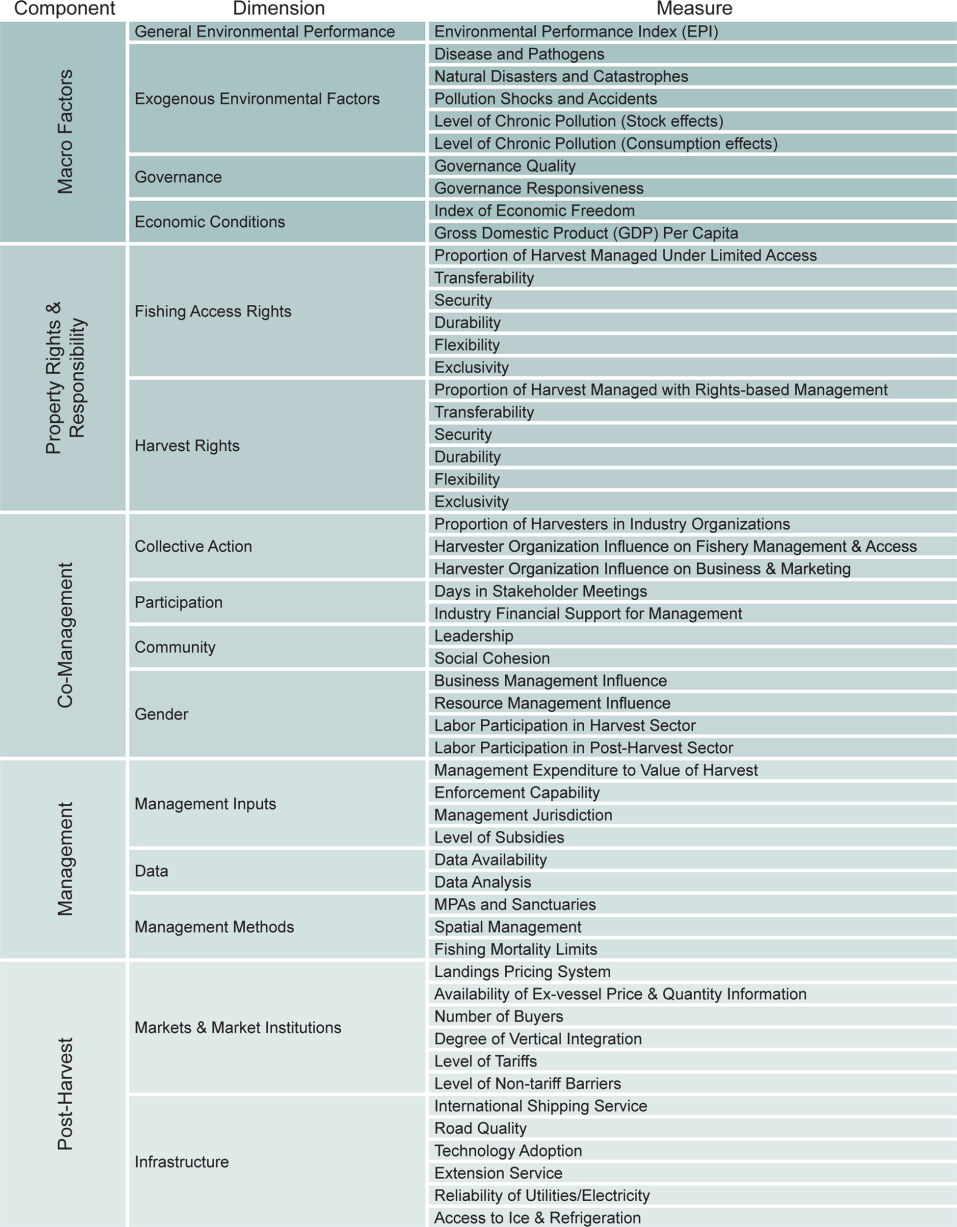
Schematic of the Input Components and the Associated Dimensions and Metrics.

To avoid conflating observed benefits with conditions or management regimes conjectured to support them, we separately identify 15 dimensions of enabling factors, reflecting five input components (Fig 2). The 54 constituent metrics capture management descriptors and exogenous enabling conditions that may affect output indicator scores. This list mirrors the empirical framework used for resource self-governance (e.g., [31–34]) by positing a link between a set of exogenous enabling conditions and management success. We extend that framework by specifying interrelated, but independent, ecological, economic and community indicators as the specific measurable notions of success. We also incorporate a broader set of enabling conditions to capture alternative management structures that can be evaluated to provide insight about management systems that are effective when enabling conditions for self-governance are not present.

Selecting a tractable and credible set of enabling factors to include is challenging since the fisheries management literature discusses many approaches, each supported by case studies of non-randomized (and nearly always successful by some measure) implementations. Some focus exclusively on technical measures (MPAs, gear innovations, TACs), others on the social institutions surrounding the resource (self-governance, property rights systems); all are influenced by macroeconomic, infrastructure and resource conditions. We draw on the highest profile mechanisms in the literature to associate measures across a broad cross section of fisheries in order to understand when each method is most effective. Dimensions and corresponding metrics are anchored in competing theories of fishery management, with the intent of testing their causal or conditional effects; it is not implied that higher input scores lead to higher output performance, or that effects are monotonic within metrics or linear across metrics.

The *Macro Factors* component of inputs reflects the institutional state of the country or region being evaluated. The *General Environmental Performance* dimension captures the condition of the environment at the national level [35], while the *Exogenous Environmental Factors* dimension identifies local levels of disease, pollution (both chronic and shocks) and natural disasters that may affect an individual fishery (e.g., [35–38]). In the *Governance* and *Economic Conditions* dimensions, national indicators reflect how much control individual citizens can exercise over social distributional decisions and resource management. High scores will reflect fisheries where participants can influence allocations, governance and enforcement to create more income, and interact freely in the market to encourage strong and sustainable partnerships among communities and businesses [27].

The *Property Rights & Responsibility* component reflects how much and what type of control individuals can exercise over the resource, and how much opportunity they have to establish and protect well-designed resource management structures. Many economists believe such rights to be critical to creating economically sustainable fisheries. The *Fishing Access Rights* dimension captures the nature and strength of the harvesters’ rights to exclude others from using the resource through limited access [39,40]. The *Harvest Rights* dimension captures the nature and strength of harvesters’ individual, or collective, rights to a specific quantity of the harvest [41].

The *Co-management* component measures the role that local stakeholders play in determining the management of the fishery, reflecting evidence that (even in the absence of strong national enforcement), local communities can improve outcomes or resolve the tragedy of the commons through cooperation [32,42]. The *Collective Action* dimension reflects the extent to which harvesters act as a group to manage the resource and support sustainable practices in place of rent dissipating ones. The *Participation* dimension measures the extent to which stakeholders are involved in crafting management, which makes management more effective [43] or increases compliance [44,45]. Other research suggests social features of the community contribute to co-management effectiveness, so Leadership and Social Cohesion metrics are included in the *Community* dimension [10,32]. Fishery management often affects the balance of power in households in a way that is either a concern of policymakers [46,47], or supports better outcomes [48], so the *Gender* dimension is included to reflect the role that women have in harvest and post-harvest activities.

The *Management* component considers how well the management system works to gather information, both from science and stakeholders, and integrate it into policymaking. The *Management Inputs* dimension reflects the state of enforcement efforts [49], extent of shared stock (e.g., [50]), the level of subsidies [51], and the amount of fishery income that is expended simply managing the fishery. Greater management resources may improve the understanding of fish stocks and lead to better management and larger sustainable harvests. In order to capture the role of quantitative analysis in management success, the *Data* dimension measures how frequently and to what extent data is collected and analyzed in order to set management targets [52]. The *Management Methods* dimension measures the application of alternative approaches to management, including MPAs [53], spatial management [54], and TACs [55,56]. All have been posited to improve ecological and economic outcomes, although further research is necessary to determine how such strategies interact with one-another, and other environmental and institutional enabling factors.

The *Post-Harvest* component reflects the extent to which a region has the economic and physical infrastructure available to enable the generation of sustainable livelihoods. The *Markets & Market Institutions* dimension captures the opportunities to generate income through sales of the fishery products, through competitive ex-vessel pricing [57–59] and through barriers to international high-value markets [60]. Under some conditions, access to high-value buyers through freely flowing competitive markets are critical to receiving the best prices [61], but in others access to these markets can be an avenue to exploitation [62]. The *Infrastructure* dimension captures the state of the technology that may enable improvements in product quality or product development, or allow access to higher value buyers.

### Metric Structure

Each of the metrics that comprise the output and input dimensions is assigned a score between 1 and 5 following criteria established for each score level. For output metrics, the level descriptors were chosen so that higher scores reflect better performance, with levels capturing (projected) quintiles or key performance benchmarks across global fisheries; we interpret a 3 as a level below which improvement could be considered. For input metrics, level descriptors were chosen to be monotonic, without presumption that higher scores are better or lead to monotonically higher output scores; endpoint scores sometimes capture extremes important for testing hypotheses in the literature (e.g., strong property rights) rather than quintiles. For a few metrics, quintile cutpoints can be established from extant global data (e.g., country-fishery landings in FISHSTAT Plus [63] was used for Annual Landings Volatility). However, for most metrics, standard data across fisheries does not exist, or it would be impossible to develop similarly scaled calculations across the range of case studies. For these, we drew on the extensive experience of our authors to develop qualitative descriptions of levels that capture rough quintiles or key benchmarks of performance across global fisheries, recasting and refining them through the pilot process.

Scoring the resulting metrics requires a basic set of data on the fishery that should be available in all significant fisheries (e.g., landings volumes and approximate prices), and local expert assessment of qualitative indicator levels. Metrics emphasize the current status of the fishery, some relative to the recent past. We avoid forecasts or sustainability estimates because data, analysis, and political processes across the range of fisheries do not support accurate projections about the status of fisheries in the future; instead, the rapid assessment nature of the FPIs facilitates repeat application that directly measures future conditions, and the role of management in determining them.

As an example, Table 1 shows the score descriptors for the *Crew Earnings Compared to Regional Average Earnings* metric; a more detailed description and scoring guidance with examples is given in the FPI manual (see *S2 Supplemental Information*). In the absence of primary data from surveys of fishermen and non-fishermen about their incomes, the scoring structure allows accurate coding: it asks the scorer to assess whether crew earnings are about the same as other jobs available in the region, a little better or worse, or a lot better or worse. With similar questions for harvester sector capital owners, processing owners, and processing workers, scores can be compared across groups to assess how rents are distributed within the fishery, and if various fishing jobs are desirable relative to other work in the community.

**Table 1:** Scoring Criteria for the *Crew Earnings Compared to Regional Average Earnings* Metric of the *Labor Returns* Dimension of the *Community* Indicator.

Earnings Compared to Regional Average Earnings	• **5**: More than 50% above the regional average;	Ratio of annual earnings from fishing per crew member to the regional average earnings. In many cases, the captain is an owner of a vessel or permit, but in other cases, captains are considered as crew. Crew is defined as those depending on others for access.
	• **4**: Between 10 and 50% above regional average;	
	• **3**: Within 10% of the regional average;	
	• **2**: Between 50% and 90% of the regional average;	
	• **1**: Less than half of the regional average	

In addition to the raw scores, each FPI metric score is also given a quality rating to reflect the scorer’s degree of certainty about the score assigned. An ‘A’ rating is given if the scorer is highly confident that the score is correct; a ‘B’ if the scorer is highly confident the true score is within one bin of the given score; and a ‘C’ reflects an educated guess about which the scorer is not able to be highly confident (see S1 Appendix). While reliable quantitative data will support calculations that lead to high confidence, high confidence can also come through the scorer’s extensive experience with the fishery or scoring descriptors that make a change between levels very unlikely.

### Comparison Sets

An essential element of the FPIs approach is to focus on defining scores from the perspective of the decision-making agent. For example, incomes are scored relative to the earner’s economic sphere and opportunity; investment risk and capital cost are assessed relative to other accessible capital sources. For some fisheries, these markets are well-functioning and international, but for many, these markets are local and poorly integrated with the broader national or global economy.

## Results

**Fig 3:**
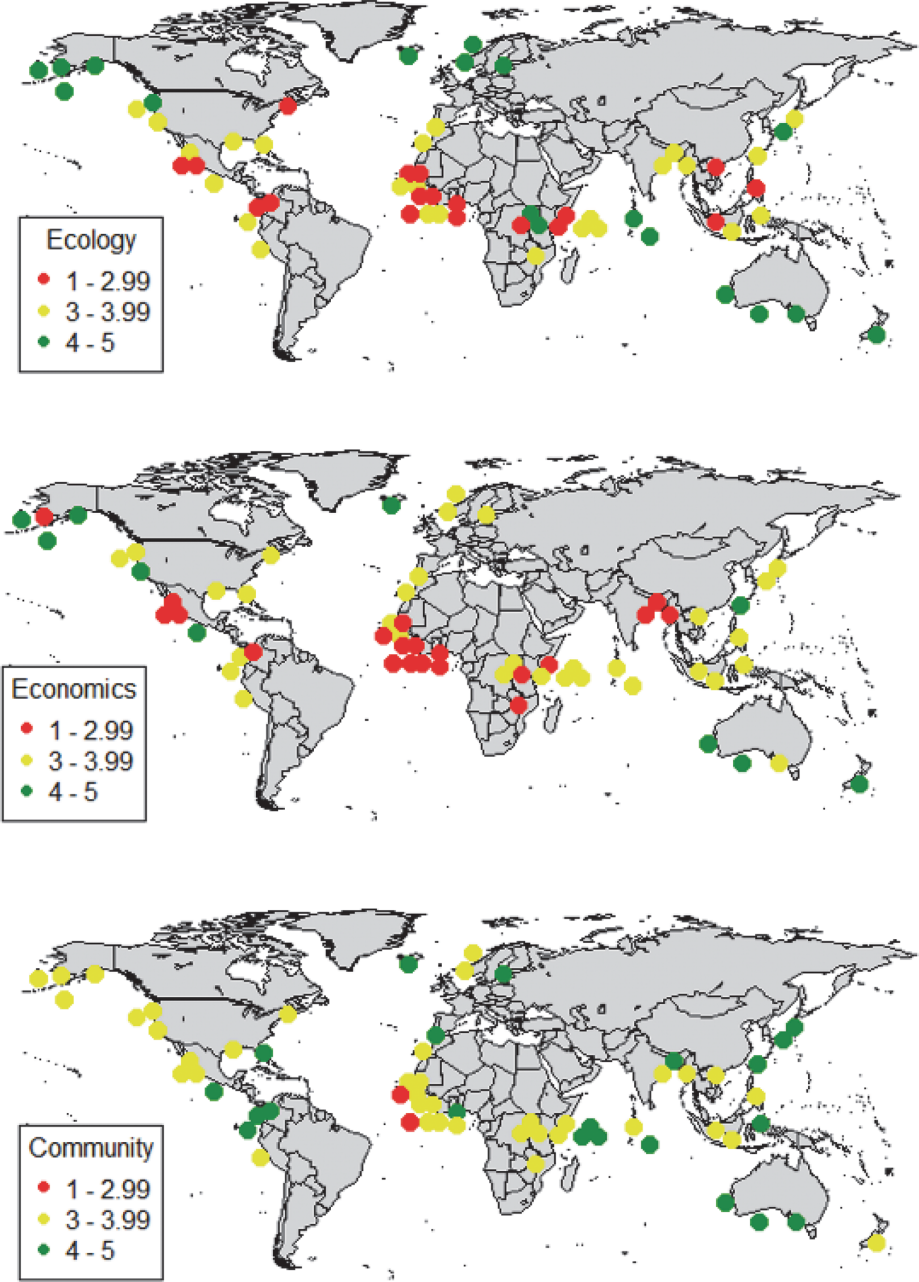
Map of Case Studies. Dot colors represent each case study fishery’s performance on *Ecology* (top), *Economic* (middle) and *Community* (bottom) indicators.

Fig 3 shows the locations and summary performance on the three TBL indicators of our 61 case studies from around the world, for fisheries ranging from of small freshwater village fisheries in Bangladesh to the highly industrialized fisheries operating in the North Pacific. *Ecology* scores are higher in the developed countries that dominate the high-latitude case studies, compared to the developing countries that dominate the equatorial sample. *Economics* scores have fewer high (green; very well performing) scores than does *Ecology*, even where fisheries are well managed, with a mix of yellow (well performing) and red (in need of improvement) across much of the world. *Community* outcomes have far fewer low (red) scores than either *Economics* or *Ecology*, and many green outcomes throughout the world, including in developing fisheries where *Ecology* and *Economics* outcomes are middling or low. Within fisheries, the TBL indicator scores are imperfectly correlated and there are very few fisheries whose scores place them within the same bin for each pillar of the TBL.

The selected case studies are best described as an opportunity sample, with developed country case studies included because one of the academic or agency co-authors was willing to complete the instrument, and developing country case studies included through author connections or World Bank involvement. As such, tractable developing country fisheries are oversampled since, as a lending organization, the World Bank choses projects where it expects the country will be able to repay the investment from increased revenue created by the investment. However, these cases are informative because they represent the range of fisheries in which investment in management change has the potential to generate social and ecological benefits, including poverty alleviation.

**Fig 4:**
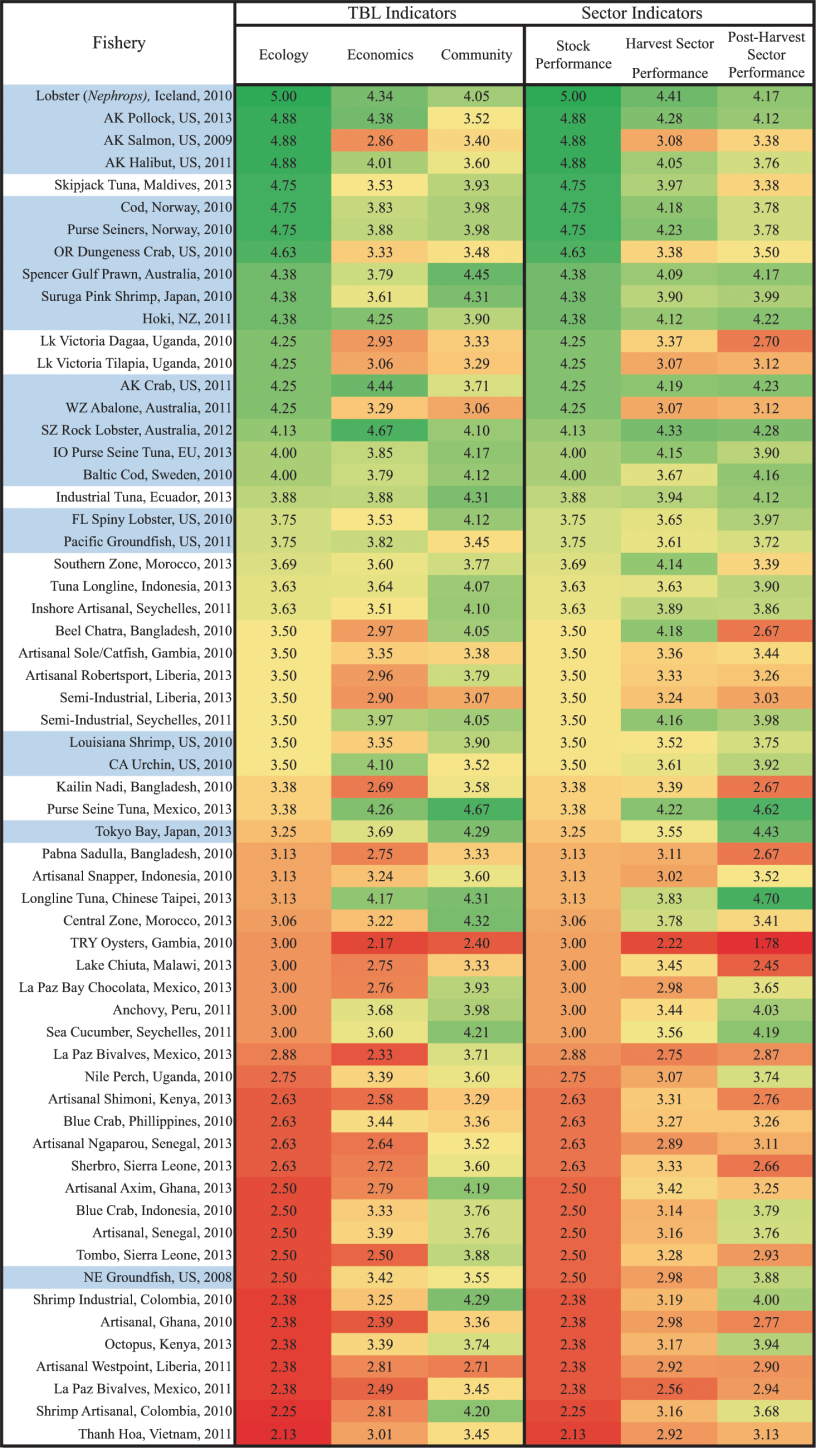
Fishery Performance Indicator Scores for 61 Global Case Studies. Case studies from countries with per capita GDP above the global median are shaded, and scores are color coded by performance (green = high; red = low).

Fig 4 lists the individual fisheries and the base year of their scoring, along with their unweighted scores for both the TBL indicators and sector indicators; rankings weighted by metric quality score are statistically indistinguishable (see S1 Appendix). Fisheries prosecuted by developed countries are highlighted in the left column. The case studies are sorted by performance on the *Ecology* indicator.

The ecological rankings are consistent with expectations: well managed fisheries, predominantly in the US and Europe and some certified by groups such as Marine Stewardship Council (MSC), rank near the top; open access developing country fisheries, along with prominent examples of overfished industrial fisheries such as New England groundfish *circa* 2008, rank near the bottom. Therefore, the discrete bin-based scoring system captures and aggregates variance in the data consistent with more precise assessments of the same fisheries.

The results of the other indicators validate the concerns that motivated the development of the FPIs. Although the *Economic* and *Community* indicators are not uncorrelated with the *Ecology* indicator (in fact, a Wilcoxon/Mann-Whitney rank sum test does not reject the hypothesis of identical rankings of the fisheries by indicator), we observe dramatic and frequent exceptions suggesting that ecology represents an unsatisfactory proxy for the other pillars of the TBL: each bottom line indicator must be monitored separately.

The case studies contain both socially successful fisheries based on depleted resources, and healthy resources that do not support high social outcomes. Economically low-performing but ecologically healthy fisheries occur where resources are abundant relative to market demand or where biologically effective management is in place, but management presents poor incentives for generating market return or minimizing cost. Striking examples include the MSC-certified Alaskan salmon and Oregon Dungeness crab derby fisheries, where revenues are spent on excess harvest capital and short seasons lead to supply gluts and low prices.

The Kenyan octopus and Seychellois sea cucumber fisheries, on the other hand, both have depleted resources but contribute significantly to their communities. In these cases, the export products command prices that are able to provide strong livelihoods and contribute to their communities, despite being poorly managed. One contributing factor is that overall lower standards of living mean that even relatively low levels of benefits from fishing can make participants well off compared to their non-fishing peers, and able to contribute meaningfully to the local economy. Another is that the fisheries may be in the process of being depleted and that infrastructure, reputations and perceptions established during this high-rent fishing-down phase persist as harvest levels fall; repeat application of the FPIs will illuminate which of these benefits of initial high levels of fishing are permanent and which are transient.

In addition to validating the need to track *Economic* and *Community* performance to complement ecological performance, the FPIs also were motivated by the need to capture the effects of the post-harvest sector. Overlooking this sector omits an important source of potential local and community benefits from fisheries—which can often exceed those from the harvest sector—and a constituency for supporting and implementing advantageous changes in fishery management [64]. Comparing the *Post-Harvest Sector Performance* indicator in the sector partitioning, we find that it deviates importantly from both the *Stock Performance* indicator and the *Harvest Sector Performance* indicator. This can occur as management strategies or market structure affect the division of rents between the harvest and post-harvest sectors. In many export fisheries, such as the Kenyan octopus and the Seychellois sea cucumber fisheries mentioned above, the *Post-Harvest Sector Performance* score is considerably higher than the *Harvest Sector Performance* score, as exporting considerably increases product value and the small number of exporters are able to capture much of the value they create. In New England groundfish, although there are a large number of dealers, they are less overcapitalized than the harvesters and thus account for much of the net economic benefit in the fishery. Broadly, cases where the post-harvest sector does worse than the harvest sector are those fisheries where processing consists mostly of preservation (smoking or drying) for consumption in local, low-value markets.

**Fig 5:**
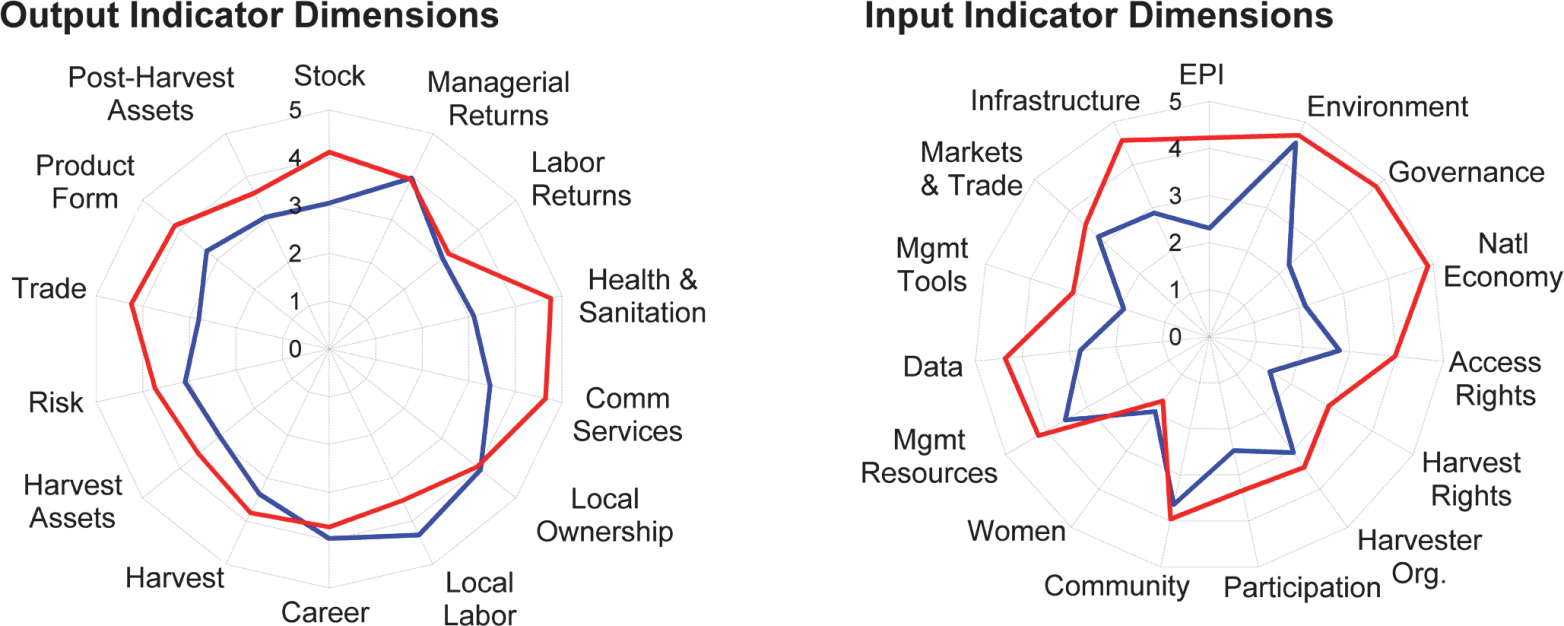
Average Triple Bottom Line FPI Scores. TBL input (left panel) and output dimensions (right panel), by developed (red) and developing (blue) country.

Fig 5 presents the average score of each dimension of the TBL partitioning, separated by developing and developed country fisheries. The stocks in our developed country case studies are about one score level higher than those in developing countries, reflecting the distribution of those cases in Fig 4. Perhaps surprisingly, developed country fisheries do not outperform on all dimensions: returns to capital owners are a level higher than laborers, but are equivalently good jobs—on average and relative to local standards—across developed and developing country fisheries. Similarly, developing countries are more likely to use local labor, and for a greater portion of working life, than developed country fisheries that attract immigrant labor at lower wages than local workers will accept.

The indicators reflect that developed countries offer better community services, generally attributable to more diverse economies, but also reveal where and how their fisheries generate more benefits. Developed country harvests, and harvest and post-harvest assets, outperform those in developing countries, reflecting that both the harvesting and processing sectors are creating more wealth from their fishery resources. They transform harvested fish into higher value products—nearly a one score level difference—and exceed developing countries at trade by more than a score level, creating value by selling products to those willing to pay the most for them.

These performance differences are associated with enabling condition disparities of nearly 2 levels in the strength of the national economies, infrastructure, governance and general environmental regulatory performance, not surprising as descriptors of the difference between developed and developing countries. While more case studies and repeated observations are needed to draw causal inferences, there are also differences in fisheries management: while scores on stakeholder participation dimensions are similar, there are large gaps in the resources for management, data collection and analysis, and the implementation of management tools, especially the strength of access rights.

**Fig 6:**
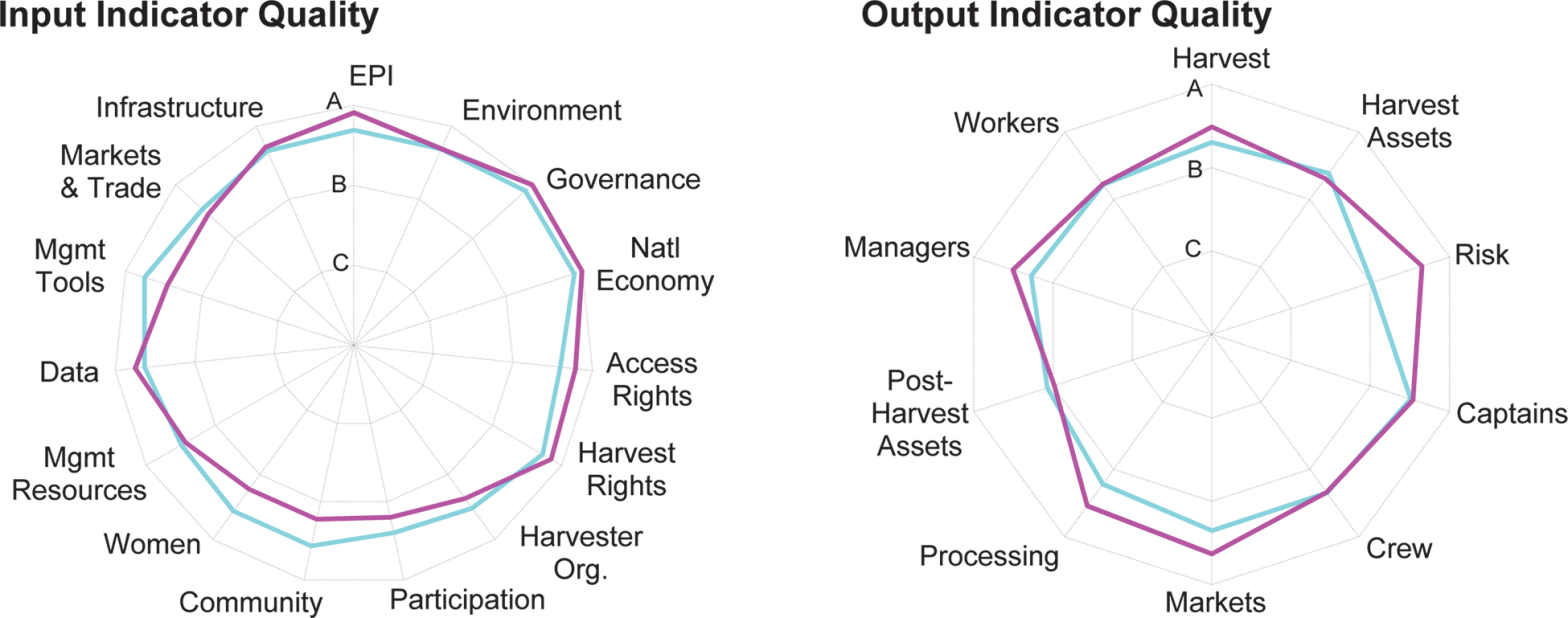
Average Sector FPI Score Quality Ratings. Sector output (left panel) and enabling factor (right panel) dimensions, by developed (pink) and developing (light blue) countries.

Fig 6 reports the score quality metadata reported by case study authors, averaged by dimension of the Sector partitioning (see S1 Appendix for metric-specific averages). These scores reveal a surprisingly small difference between the developed and developing country fisheries. The largest gap is on the *Risk* dimension, which ideally would be scored based on calculations with systematically collected time series of price and landings data; the gap reflects that developing countries often lack this data (and experience does not provide confidence on variance). It is noteworthy that in the postharvest sector, even in developed countries, many of the dimensions fall to ‘B’ information quality—typical of developing countries on many dimensions—because it is a data-poor sector, often neglected in fishery management evaluation despite being a source of considerable benefits. Assessors of developing country fisheries were able to score their inputs with the same degree of confidence as those in developed countries.

## Discussion

The FPIs are a tool for identifying management strategies that work under combinations of exogenous circumstances when the goal is not merely stock sustainability, but also to generate economic and community benefits from fishery resources. Analysis of 61 global case studies, drawn from an opportunity sample of developed and developing country fisheries, reveal the importance of measuring the elements of the TBL separately, rather than focusing on one and expecting a direct correlation with others. Further, the FPIs are able to assess data-poor fisheries—and sectors—with sufficient confidence and consistency to capture meaningful variation in performance among fisheries.

Adding dimensions to benchmarking and performance assessment is burdensome, and strains the disciplinary and sectoral expertise, as well as capacity, of assessors. Similarly, there are limitations and challenges associated with using expert assessments as a proxy for data (e.g., [65]). Therefore, there is and will continue to be a role for detailed case studies, but there are important uses for broad rapid assessment tools that can be applied simply and quickly by experienced experts with a range of backgrounds in nearly any fishery. We envision application by academics, local fishery managers, fishery researchers, and development agents and consultants, contributing to a large reference base of panel data. The FPIs’ strategy of foregoing precision (but not accuracy) for rapid assessment of individual fisheries facilitates the assembly of a large-N data set, which is a well-recognized challenge in the literature on the commons [66]. Further, by clearly delineating measures of performance and enabling factors, it provides a lexicon that can be used flexibly in linking different notions of success with concrete measures.

The FPIs can serve several roles and different audiences. Harvesters and processors in a fishery can use the FPIs to compare their performance with similar fisheries around the world to identify specific sources of foregone wealth and food security in their own fisheries, and to establish predicates for improvement. Suppliers and retailers who engage in international seafood trade can draw on the FPIs to identify fisheries where not just ecology and biological management aspects of the fishery are considered sustainable, but so is the local industry, assuring a supply stream that is reliable, high-quality and justifiable to consumers. The FPIs also provide a framework for investors and bankers to identify fisheries with high potential for return and low risk associated with improper management or dysfunctional communities.

For international development agencies, the FPIs can assist in identifying fisheries that are not meeting their potential as a platform for supporting local economic development that improves standards of living and alleviates poverty. Within those fisheries, it will illuminate specific dimensions where improvement is needed. While individual projects have specific objectives that need to be benchmarked and monitored in greater detail, over the course of a project the FPIs are an off-the-shelf low-resource requirement accountability tool with which progress across the TBL can be tracked, and compared to contemporaneous progress in other countries that did not receive the investment, or received different interventions. For supporting NGOs, the FPIs can provide data for their stakeholder outreach and political action materials and enhance the knowledge base of their contributors.

As a research tool to support science-based policy, the FPIs establish a common baseline dataset on global communities, management and success, supporting the development of nuanced, empirically-based models relating institutions and practices to outcomes. While previous global cross-sectional studies of fishery management have focused at the country level (e.g., [11,67]), the FPIs’ strategy of assessing individual fisheries—and the ability to reassess fisheries rapidly to produce panel data—will eventually provide a mechanism for understanding the causal and supporting relationships between each notion of success represented by the triad of outcome indicators, and the prevailing enabling conditions and alternative resource management strategies.

This tool capitalizes on concurrent fishery-to-fishery variation in management strategies and enabling conditions to provide hundreds of natural experiments in alternative management structures that can be used to draw lessons from successful fisheries to those that are not supporting their stakeholders or communities. Many leading hypotheses may be testable based on FPI data alone, and others that emerge can be tested by supplementing the FPI framework for specific fisheries. Additional indicators of sociological or ecological variables, perhaps requiring more detailed data collection, could also be merged for a subset of countries, allowing testing of additional hypotheses. With additional case studies, outcomes can be linked with context and management, and we can identify science-based strategies that capture the wealth the oceans have the potential to provide.

## Supporting Information

S1 Appendix Score Quality-Weighted Indicators. (PDF)

S1 Dataset Metric Scores. Metric scores and score quality ratings for reported case studies.
(PDF)

S1 Supporting Information FPI Scoring Worksheet. Worksheet used to actually score case studies, with abbreviated metric scoring descriptions and guidance.
(XLSX)

S2 Supporting Information FPI Manual v2.0. Detailed manual for applying and scoring FPI metrics.
(PDF)

## References

[1] Scott A. The Fishery: The Objectives of Sole Ownership. J Polit Econ. 1955;63: 116–24.

[2] Gordon HS. The Economic Theory of Common Property Resources. J Polit Econ. 1954;62: 124–42.

[3] Hardin G. The Tragedy of the Commons. Science. 1963;13: 1243–48. 5699198

[4] Worm B, Hilborn R, Baum J, Branch T, Collie J, et al Rebuilding Global Fisheries. Science. 2009;325: 578–85. 10.1126/science.1173146 19644114

[5] Allison EH, Ellis F. The Livelihoods Approach and Management of Small-scale Fisheries. Mar Policy. 2001;25: 377–388.

[6] The World Bank. The Sunken Billions: The Economic Justification for Fisheries Reform. Washington, DC: Agriculture and Rural Development; 2008.

[7] Sanchirico J, Wilen J. Global Marine Fisheries Resources: Status and Prospects Sustainable Development Issue Brief 02–17. Washington, DC: Resources for the Future; 2002.

[8] FAO (Food and Agriculture Organization of the United Nations) Marine Fisheries and the Law of the Sea: A Decade of Change The State of Food and Agriculture 1992. FAO Circular 853. Rome: FAO; 1993.

[9] Wilen J. Property Rights and the Texture of Rents in Fisheries In: Leal D, editor. Evolving Property Rights in Marine Fisheries. Lanham, MD: Rowman & Littlefield; 2005 pp 49–68.

[10] Gutiérrez NL, Hilborn R, Defeo O. Leadership, social capital and incentives promote successful fisheries. Nature. 2011; 470: 386–389. 10.1038/nature09689 21209616

[11] Smith M, Roheim C, Crowder L, Halpern B, Turnipseed M, Anderson J, et al Sustainability and Global Seafood. Science. 2010;327: 784–6. 10.1126/science.1185345 20150469

[12] Dietz T, Ostrom E, Stern P. Governing the Commons. Science. 2003;302: 1907–12. 14671286 10.1126/science.1091015

[13] Halpern B, Klein C, Brown C, Berger M, Grantham H, Mangubhai S, et al Achieving the Triple Bottom Line in the Face of Inherent Trade-Offs among Social Equity, Economic Return, and Conservation. PNAS. 2013;110: 6229–34. 10.1073/pnas.1217689110 23530207 PMC3625307

[14] Ehrlich PR, Kareiva PM, Daily GC. Securing Natural Capital and Expanding Equity to Rescale Civilization. Nature 2012;486: 68–73. 10.1038/nature11157 22678281

[15] The Blue Ribbon Panel (Chair, O. Hoegh-Guldburg) Indispensable Ocean: Aligning Ocean Health and Human Well-Being. Washington, DC: The Blue Ribbon Panel; 2013 Available: https://globalpartnershipforoceans.org/indispensable-ocean.

[16] Abbott J, Anderson J, Campling L, Hanneson R, Havice E, Lozier MS, et al Steering the Global Partnership for Oceans. Mar Resour Econ. 2014;29: 1–16.

[17] Rockefeller Foundation. Securing the Livelihoods and Nutritional Needs of Fish-Dependent Communities; 2013. 34pp. Available: http://www.rockefellerfoundation.org/blog/securing-livelihoods-nutritional-needs Accessed June 2014.

[18] The Prince’s Charities, International Sustainability Unit. Toward Global Sustainable Fisheries: The Opportunity for Transition; 2012. 47pp. Available: http://www.pcfisu.org/wp-content/uploads/2012/01/ISUMarineprogramme-towards-global-sustainable-fisheries.pdf Accessed August 2014.

[19] Wamukota AW, Cinner JE, McClanahan TE. Co-management of Coral Reef Fisheries: A Critical Evaluation of the Literature. Mar Policy. 2012;36: 481–88.

[20] Maliao R, Pomeroy R, Turingan R. Performance of Community-based Coastal Resource Management (CBCRM) Programs in the Philippines: A Meta-analysis. Mar Policy. 2009;33: 818–825.

[21] Plummer R, Armitage D. A Resilience-based Framework for Evaluating Adaptive Co-management: Linking Ecology, Economics and Society in a Complex World. Ecol Econ. 2007;61: 62–74.

[22] Costello C, Gaines S, Lynham J. Can Catch Shares Prevent Fisheries Collapse? Science. 2008;321: 1678–1681. 10.1126/science.1159478 18801999

[23] Essington TE, Melnychuk MC, Branch TA, Heppell SS, Jensen OP, Link JS, et al Catch Shares, Fisheries, and Ecological Stewardship: A Comparative Analysis of Resource Responses to a Rights-based Policy Instrument. Conserv Lett. 2012;5: 186–195.

[24] Branch TA. How do Individual Transferable Quotas Affect Marine Ecosystems? Fish Fish. 2009;10: 39–57.

[25] Lester S, Halpern B, Grorud-Colvert K, Lubchenko J, Ruttenberg B, Gaines S, et al Biological Effects within No-take Marine Reserves: A Global Synthesis. Mar Ecol Prog Ser. 2009;384 2: 33–46.

[26] Hilborn R, Stokes K, Maguire J-J, Smith T, Botsford LW, Mangel M, et al When Can Marine Reserves Improve Fisheries Management? Ocean Coast Manage. 2004;47: 197–205.

[27] Hilborn R. Moving to Sustainability by Learning from Successful Fisheries. AMBIO. 2007;36: 296–303. 17626466 10.1579/0044-7447(2007)36[296:mtsblf]2.0.co;2

[28] Chu J, Anderson JL, Anderson CM. Evaluation of New Fishery Performance Indicators (FPIs) ARD 52. Washington, DC: The World Bank Group; 2012 100 pp. Available: http://siteresources.worldbank.org/INTARD/825826-1111129171182/23192329/ARD_DP12_BlueCrab_web_final.pdf. Accessed March 2015.

[29] Cunningham S, Neiland A, Arbuckle M, Bostock T. Wealth-based Fisheries Management: Using Fisheries Wealth to Orchestrate Sounds Fisheries Policy in Practice. Mar Resour Econ. 2009;24: 271–87.

[30] Wilen J. The Challenges of Pro-Poor Fisheries Reform. Mar Resour Econ. 2013;28: 203–20.

[31] Ostrom E. A General Framework for Analyzing Sustainability of Social-Ecological Systems. Science. 2009;325: 419 10.1126/science.1172133 19628857

[32] Ostrom E. Governing the Commons: The Evolution of Institutions for Collective Action. London: Cambridge University Press; 1990.

[33] Agrawal A. Common Resources and Institutional Sustainability In: Ostrom E, et al, editors. The Drama of the Commons. Washington DC: National Academy Press; 2002.

[34] Cinner J, McClanahan T, MacNeil MA, Graham NAJ, Daw T, Mukminin A, et al Comanagement of Coral Reef Social-ecological Systems. PNAS. 2012;109: 5219–5222. 10.1073/pnas.1121215109 22431631 PMC3325732

[35] Esty DC, Kim C, Srebotnjak T, Levy MA, de Sherbinin A, Mara V. Environmental Performance Index New Haven, CT: Yale Center for Environmental Law and Policy and New York, NY: Columbia University; 2008.

[36] Cohen M. Technological Disasters and Natural Resource Damage Assessment: An Evaluation of the Exxon Valdez Oil Spill. Land Econ. 1995;71: 65–82.

[37] Edinger EN, Jompa J, Limmon GV, Widjatmoko W, Risk MJ. Reef Degradation and Coral Biodiversity in Indonesia: Effects of Land-Based Pollution, Destructive Fishing Practices and Changes Over Time. Mar Poll Bull. 1998;36: 617–630.

[38] Asche F, Hansen H, Tveteras R, Tveteras S. The Salmon Disease Crisis in Chile. Mar Resour Econ. 2009;24: 405–411.

[39] Anderson JL. Sustainable Aquaculture: What Does it Mean and How Do We Get There? In: Leung PS, Lee CS, O’Bryen PJ, editors. Species & System Selection for Sustainable Aquaculture. Ames, IA and Oxford, UK: Blackwell Publishing; 2007 pp 9–17.

[40] Anderson JL. Aquaculture and the Future: Why Economists Should Care. Mar Resour Econ. 2002;17: 133–151.

[41] Christy F. Fishermen Quotas: A Tentative Suggestion for Domestic Management. Occasional Paper No. 19 of the Law of the Sea Institute, University of Rhode Island; 1973.

[42] Jentoft S, McCay BJ. User Participation in Fisheries Management: Lessons Drawn from International Experiences. Mar Policy. 1995;19: 227–246.

[43] McCay BJ, Jentoft S. From the Bottom Up: Participatory Issues in Fisheries Management. Soc Nat Resour. 1996;9: 237–250.

[44] Pomeroy RS, Berkes F. Two to Tango: The Role of Government in Fisheries Co-management. Mar Policy. 1997;21: 465–480.

[45] Kuperan K, Sutinen J. Blue Water Crime: Deterrence, Legitimacy, and Compliance in Fisheries. L & Soc Rev. 1998;32: 309–338.

[46] Hapke HM. Petty Traders, Gender, and Development in a South Indian Fishery. Econ Geogr. 2001;77: 225–249.

[47] Geheb K, Kalloch S, Medard M, Nyapendi AT, Lwenya C, Kyangwa M. Nile Perch and the Hungry of Lake Victoria: Gender, Status and Food in an East African Fishery. Food Policy. 2008;33: 85–98.

[48] Harper S, Zeller F, Hauzer M, Pauly D, Sumaila UR. Women and Fisheries: Contribution to Food Security and Local Economies. Mar Policy. 2013;39: 56–63.

[49] Sutinen JG, Gauvin JR, Gordon DV. An Econometric Study of Regulatory Enforcement and Compliance in the Commercial Inshore Lobster Fishery of Massachusetts In: Neher PA, Arnason R, Mollett N, editors. Rights Based Fishing, NATO ASI Series. Netherlands: Springer; 1989 Pp 415–431.

[50] Munro GR. The Conservation and Management of Shared Fish Stocks: Legal and Economic Aspects. Rome: FAO; 2004.

[51] Sumaila UR, Khan AS, Dyck AJ, Watson R, Munro G, Tydemers P, et al A Bottom-up Re-estimation of Global Fisheries Subsidies. J Bioecon. 2010;12: 201–225.

[52] Zeller D, Froese R, Pauly D. On Losing and Recovering Fisheries and Marine Science Data. Mar Policy. 2005;29: 69–73.

[53] Roberts C, Hawkins J, Gell F. The Role of Marine Reserves in Achieving Sustainable Fisheries. Phil Trans R Soc B. 2005;360 1453: 123–132. 15713592 10.1098/rstb.2004.1578 PMC1636100

[54] Cancino JP, Uchida H, Wilen JE. TURFs and ITQs: Collective vs. Individual Decision Making. Mar Res Econ. 2007;22: 391–406.

[55] Daan N. TAC Management in North Sea Flatfish Fisheries. J Sea Res. 1997;37: 321–341.

[56] Copes P. A Critical Review of the Individual Quota as a Device in Fisheries Management. Land Econ. 1986;62: 278–291.

[57] Jensen R. The Digital Provide: Information (Technology), Market Performance, and Welfare in the South Indian Fisheries Sector. Q J Econ. 2007;122: 879–924.

[58] Matulich S, Sever M. Reconsidering the Initial Allocation of ITQs: The Search for a Pareto-safe Allocation between Fishing and Processing Sectors. Land Econ. 1999;75: 203–219.

[59] Adelaja A, Menzo J, McCay BJ. Market Power, Industrial Organization and Tradable Quotas. Rev Ind Organ. 1998;13: 589–601.

[60] Hannesson R. Effects of Liberalizing Trade in Fish, Fishing Services and Investment in Fishing Vessels. OECD Papers 1; 2001.

[61] Schmitt KM, Kramer DB. Road Development and Market Access on Nicaragua’s Atlantic Coast: Implications for Household Fishing and Farming Practices. Environ Conserv. 2009;36: 289–300.

[62] Liese C, Smith MD, Kramer R. Open Access in a Spatially Delineated Artisanal Fishery: The Case of Minahasa, Indonesia. Environ Dev Econ. 2007;12: 123–143.

[63] FAO (Food and Agriculture Organization of the United Nations) FishStat Plus- Universal Software for Fishery Statistical Time Series; 2011. Available: http://www.fao.org/fishery/topic/16073/en. Accessed December 2014.

[64] Foley P. The Political Economy of the Marine Stewardship Council Certification: Processors and Access in Newfoundland and Labrador’s Inshore Shrimp Industry. J Agrar Change. 2012;12(2&3): 436–57.

[65] Burgman MA, McBride M, Ashton R, Speirs-Bridge A, Flander L, Wintle B, et al Expert Status and Performance. PLoS ONE. 2011;6(7): e22998 10.1371/journal.pone.0022998 21829574 PMC3146531

[66] Poteete A, Ostrom E. Fifteen Years of Empirical Research on Collective Action in Natural Resource Management: Struggling to Build Large-N Databases Based on Qualitative Research. World Dev. 2008;36 1: 176–95.

[67] Halpern BS, Longo C, Hardy D, McLeod KL, Samhouri JF, Katona SK, et al An Index to Assess the Health and Benefits of the Global Ocean. Nature. 2012;488: 615–20. 10.1038/nature11397 22895186

