# Supplementary material for: The Fishery Performance Indicators: A Management Tool for Triple Bottom Line Outcomes
Source: PLoS One. 2015 May 6;10(5):e0122809. doi: 10.1371/journal.pone.0122809 (PMC4422616; doi:10.1371/journal.pone.0122809)
Supplement: S1 Appendix — (PDF) [file pone.0122809.s001.pdf]

Data Quality Appendix

For each metric scored, assessors also indicated their level of confidence in the numeric score given according to the following rubric:

A. Reviewer is highly confident (95%) the 1-5 score is correct. Confidence can come from familiarity with the fishery, the reliability of another expert source, a calculation based on reliable data, or large ranges of the underlying measure for the given score that make another score highly unlikely for the fishery. Note that it is confidence in the 1-5 score that matters, and thus wide ranges for the underlying measure associated with a score can support A quality, even in the case when information about the precise level of the underlying measure is poor.
B. Reviewer feels 1-5 score is more likely than others, and reviewer is highly confident (95%) that the true underlying measure would be within one of the given score.
C. Reviewer is making an educated guess based on best available information, but reviewer is not highly confident the true measure would be within one of the given score.

Average Metric Quality

Table QA1 shows the average quality score of each metric, coding alphabetic scores as 3.0 for A, 2.0 for B and 1.0 for C. The overall range is small, with nearly all averaging above a B (2.0), reflecting overall reasonable confidence on the part of reviewers (with the caveat that reviewers are often overconfident in their own judgments). More commonly, the drivers of differences in quality are based on the sector or case study, rather than one measure being uniformly lower quality across the dataset; eliminating lower quality measures would prevent measuring important dimensions of the indicators such as Risk and Labor Returns, which dominate the lower-quality metrics. That differences in quality are related to the prevalence of data programs in different fisheries (grouped usefully by developed or developing country) or by sector is reflected in Figure 6.

Table QA1: Average Reviewer Quality Score of Each Metric

Metric	Quality Score	Input/Output	Dimension
QOFCertified	2.933333333	Output	Fish Stock
QIGResponsiveness	2.931034483	Input	Governance
QINGDP	2.931034483	Input	Economic Conditions
QINIEF	2.929824561	Input	Economic Conditions
QIGQuality	2.913793103	Input	Governance
QIIIce	2.810344828	Input	Infrastructure
QIEChronicConsumption	2.793103448	Input	Exogenous Environmental Factors

QIADurability	2.771929825	Input	Access Rights
QIHDurability	2.771428571	Input	Harvest Rights
QI_EPI	2.766666667	Input	General Environmental Performance
QIITechnology	2.75862069	Input	Infrastructure
QIHSecurity	2.75	Input	Harvest Rights
QIHProportion	2.745762712	Input	Harvest Rights
QIIRoads	2.740740741	Input	Infrastructure
QIHExclusivity	2.717948718	Input	Harvest Rights
QIEDisasters	2.706896552	Input	Exogenous Environmental Factors
QIAProportion	2.706896552	Input	Access Rights
QIDAvailability	2.706896552	Input	Data
QILTACs	2.706896552	Input	Management Methods
QIIExtenstion	2.706896552	Input	Infrastructure
QIIElectricity	2.706896552	Input	Infrastructure
QIHTransfer	2.702702703	Input	Harvest Rights
QOHSeasonLen	2.7	Output	Harvest
QOOEdAccess	2.7	Output	Community Services
QIEDisease	2.689655172	Input	Exogenous Environmental Factors
QIASecurity	2.684210526	Input	Access Rights
QOONonresident	2.683333333	Output	Local Ownership
QIATransfer	2.681818182	Input	Access Rights
QIHFlexibility	2.675	Input	Harvest Rights
QOSFinalMarket	2.666666667	Output	Product Form
QILMPAs	2.655172414	Input	Management Methods
QOSUSEUExport	2.65	Output	Trade
QIWHarvest	2.642857143	Input	Gender
QIEPollutionShocks	2.637931034	Input	Exogenous Environmental Factors
QIAExclusivity	2.637931034	Input	Access Rights
QOFRegMort	2.633333333	Output	Fish Stock
QOSFinalWealth	2.633333333	Output	Trade
QITBuyers	2.631578947	Input	Markets
QITVerticalIntegration	2.631578947	Input	Markets
QIMEnforcement	2.620689655	Input	Management Inputs
QIDAnalysis	2.620689655	Input	Data
QOOHealthAccess	2.616666667	Output	Health & Sanitation
QIEChronicStock	2.603448276	Input	Exogenous Environmental Factors
QIOParticipation	2.596491228	Input	Collective Action
QITPricing	2.596491228	Input	Markets
QOFSelectivity	2.583333333	Output	Fish Stock
QILSpatial	2.578947368	Input	Management Methods
QIMSubsidies	2.571428571	Input	Management Inputs
QOACapFunction	2.566666667	Output	Harvest Assets
QIMJurisdiction	2.551724138	Input	Management Inputs
QOOSocialStanding	2.55	Output	Managerial Returns

QOPImprovement	2.542372881	Output	Product Form
QOMHealthAccess	2.516666667	Output	Health & Sanitation
QICCohesion	2.50877193	Input	Community
QOCHealthAccess	2.5	Output	Health & Sanitation
QOCNonresident	2.5	Output	Local Labor
QOMNonresident	2.5	Output	Local Ownership
QOALoanSource	2.483333333	Output	Harvest Assets
QORContest	2.483333333	Output	Community Services
QOFOverfished	2.475409836	Output	Fish Stock
QOFHabitat	2.466666667	Output	Fish Stock
QOWNonresident	2.466666667	Output	Local Labor
QIAFlexibilty	2.456140351	Input	Access Rights
QITPriceAvailabilty	2.456140351	Input	Markets
QOCExperience	2.45	Output	Career
QOSExport	2.45	Output	Trade
QIOMgmtInfluence	2.438596491	Input	Collective Action
QOCAge	2.433333333	Output	Career
QIWMgmtInfluence	2.410714286	Input	Gender
QICLeadership	2.403508772	Input	Community
QOHLandings	2.4	Output	Harvest
QOPSupport	2.4	Output	Community Services
QOMEdAccess	2.4	Output	Community Services
QOCEdAccess	2.396551724	Output	Community Services
QOPSanitation	2.389830508	Output	Health & Sanitation
QITNonTariffs	2.385964912	Input	Markets
QORAnnLandVol	2.379310345	Output	Risk
QOSPrice	2.372881356	Output	Harvest
QOOWage	2.366666667	Output	Managerial Returns
QOPUtilization	2.366666667	Output	Product Form
QITTariffs	2.363636364	Input	Markets
QIWBusInfluence	2.357142857	Input	Gender
QIOBusInfluence	2.350877193	Input	Collective Action
QOWHealthAccess	2.35	Output	Health & Sanitation
QIIShipping	2.344827586	Input	Infrastructure
QIWPostHarvest	2.339285714	Input	Gender
QIPDays	2.333333333	Input	Participation
QOSMargin	2.322033898	Output	Product Form
QOWSocialStanding	2.316666667	Output	Labor Returns
QOAAssetVal	2.310344828	Output	Harvest Assets
QORAnnPriceVol	2.293103448	Output	Risk
QOCSocialStanding	2.293103448	Output	Labor Returns
QIPFinancial	2.293103448	Input	Participation
QOHSafety	2.271186441	Output	Health & Sanitation
QOFIUU	2.266666667	Output	Fish Stock

QOMWage	2.266666667	Output	Managerial Returns
QOATotRevenue	2.263157895	Output	Harvest Assets
QOAAssetEarnings	2.254237288	Output	Harvest Assets
QOBLoanSource	2.25	Output	Post-Harvest Assets
QOWEdAccess	2.25	Output	Community Services
QOALoanRate	2.240740741	Output	Harvest Assets
QOMEarnings	2.224137931	Output	Managerial Returns
QOPYield	2.220338983	Output	Product Form
QOMSocialStanding	2.2	Output	Managerial Returns
QOOEarnings	2.186440678	Output	Managerial Returns
QOCWage	2.172413793	Output	Labor Returns
QORAnnRevVol	2.157894737	Output	Risk
QOFRebuild	2.147540984	Output	Fish Stock
QOFDegOverfishing	2.131147541	Output	Fish Stock
QIMExpenditure	2.107142857	Input	Management Inputs
QOWExperience	2.101694915	Output	Career
QOHExCapacity	2.083333333	Output	Harvest
QOPShrink	2.083333333	Output	Product Form
QORIntrAnnPriceVol	2.074074074	Output	Risk
QOWEarnings	2.016949153	Output	Labor Returns
QOSRelativePrice	2.016666667	Output	Trade
QOBCapFunction	2.016666667	Output	Post-Harvest Assets
QOWWage	2.016666667	Output	Labor Returns
QORIntraAnnLandVol	2	Output	Risk
QOCEarnings	1.98245614	Output	Labor Returns
QORSpatialPriceVOl	1.980769231	Output	Risk
QOBLoanRate	1.777777778	Output	Post-Harvest Assets

Results Robustness to Score Quality

Figure QA1 reconstructed Figure 4's rankings by calculating dimension scores by weighting individual metrics by 3 for A quality, 2 for B quality and 1 for C quality, then calculating the indicator values from the resulting dimension scores. The rankings are shown below; a rank correlation test fails to reject the hypothesis that these rankings match those in Figure 4 at conventional levels.

Figure QA1: Fishery Performance Indicator scores for 61 global case studies, weighted by score quality; case studies from countries with per capita GDP above the global median are shaded, and scores are color coded by performance (green=high; red=low)

FPI Output Scores

Fishery				Output Category					
				Ecology	Econ.	Comm.	Ecology	Harvest	Post-Harvest
AK Pollock	-	US	2013	4.75	4.00	3.30	4.75	3.97	3.77
Lobster (*Nephrops Norvegicus*)	-	Iceland	2010	4.74	4.02	3.80	4.74	4.08	3.91
AK Halibut	-	US	2011	4.64	3.62	3.06	4.64	3.55	3.36
Skipjack Tuna	-	Maldives	2013	4.54	3.06	3.54	4.54	3.53	2.90
OR Dungeness Crab	-	US	2010	4.52	3.20	3.39	4.52	3.29	3.40
AK Salmon	-	US	2009	4.51	2.61	3.02	4.51	2.69	3.00
Cod	-	Norway	2010	4.50	3.24	3.19	4.50	3.53	3.14
Purse Seiners	-	Norway	2010	4.50	3.27	3.19	4.50	3.56	3.14
Gulf Prawn	-	Australia	2010	4.38	3.50	4.13	4.38	3.90	3.83
Lake Victoria Dagaa	-	Uganda	2010	4.25	2.46	3.02	4.25	2.95	2.36
Lake Victoria Tilapia	-	Uganda	2010	4.25	2.48	3.04	4.25	2.60	2.72
Southern Zone Rock Lobster	-	Australia	2012	4.00	3.71	4.32	4.00	3.98	4.02
Baltic Cod	-	Sweden	2010	4.00	3.52	3.94	4.00	3.40	3.94
AK Crab	-	US	2011	3.93	3.91	2.35	3.93	3.56	2.99
Suruga Pink Shrimp	-	Japan	2010	3.88	3.07	3.77	3.88	3.55	3.29
Hoki	-	New Zealand	2011	3.86	3.04	2.93	3.86	3.33	3.01
Indian Ocean Purse Seine Tuna	-	EU	2013	3.84	3.49	3.84	3.84	3.76	3.60
NE Groundfish	-	US	2008	3.50	3.14	3.17	3.50	2.78	3.53
Industrial Tuna	-	Ecuador	2013	3.46	3.55	4.18	3.46	3.70	3.88
Nearshore Artisanal	-	Seychelles	2011	3.44	3.10	4.06	3.44	3.63	3.59
Pacific Groundfish	-	US	2011	3.33	3.07	2.75	3.33	3.10	2.68
W. Pacific Tuna Artisanal	-	Indonesia	2013	3.29	3.03	3.87	3.29	3.29	3.40
Western Zone Abalone	-	Australia	2011	3.27	2.82	3.18	3.27	3.13	2.88
Semi-Industrial	-	Seychelles	2011	3.21	3.56	4.05	3.21	3.83	3.84
Beel Chatra Region	-	Bangladesh	2010	3.19	2.53	3.59	3.19	3.68	2.10
FL Spiny Lobster	-	US	2010	3.15	3.16	3.62	3.15	3.36	3.47
Artisanal Sole and Catfish	-	Gambia	2010	2.98	2.83	2.96	2.98	2.96	2.85
Kailin Nadi Region	-	Bangladesh	2010	2.96	2.40	2.95	2.96	2.92	2.10
Purse Seine/Ranching Tuna	-	Mexico	2013	2.96	3.67	4.05	2.96	3.50	4.02
Tokyo Bay	-	Japan	2013	2.94	3.42	4.11	2.94	3.35	4.20
Louisiana Shrimp	-	US	2010	2.94	2.51	3.67	2.94	2.64	3.34
Small-Scale Longline Tuna	-	Chinese Taipei	2013	2.87	3.52	3.96	2.87	3.19	4.30
Sea Cucumber	-	Seychelles	2011	2.84	3.24	4.13	2.84	3.30	3.97
La Paz Bay Chocolata clam	-	Mexico	2013	2.79	2.48	3.72	2.79	2.71	3.35
Pabna Sadullaspracole Region	-	Bangladesh	2010	2.76	2.37	2.80	2.76	2.60	2.10
CA Urchin	-	US	2010	2.73	3.44	2.63	2.73	3.07	2.99
Ensenada de La Paz Bivalves	-	Mexico	2013	2.69	1.95	3.27	2.69	2.52	2.19
TRY Oysters	-	Gambia	2010	2.69	1.72	2.30	2.69	1.82	1.62
Lesser Sunda Artisanal Snapper	-	Indonesia	2010	2.65	2.48	2.06	2.65	2.19	2.25
C (Southern Zone)	-	Morocco	2013	2.55	3.23	3.24	2.55	3.62	2.83

Blue Crab	-	Phillippines	2010	2.50	2.79	3.03	2.50	2.96	2.62
Artisanal Robertsport /Marshal	-	Liberia	2013	2.43	2.13	2.89	2.43	2.44	2.45
Semi-Industrial	-	Liberia	2013	2.43	2.08	2.35	2.43	2.35	2.29
Artisanal	-	Senegal	2010	2.40	2.64	3.27	2.40	2.60	3.07
Ensenada de La Paz Bivalves	-	Mexico	2011	2.38	1.94	2.94	2.38	2.22	2.11
Artisanal Shimoni	-	Kenya	2013	2.37	2.01	2.58	2.37	2.89	2.07
Nile Perch	-	Uganda	2010	2.37	3.01	3.28	2.37	2.77	3.25
Artisanal Ngaparou	-	Senegal	2013	2.24	1.97	3.04	2.24	2.19	2.53
Sherbro	-	Sierra Leone	2013	2.20	1.82	3.16	2.20	2.37	2.09
Anchovy	-	Peru	2011	2.20	3.15	3.41	2.20	2.92	3.38
A+B (Central Zone)	-	Morocco	2013	2.17	2.91	3.80	2.17	3.38	2.89
Blue Crab	-	Indonesia	2010	2.16	3.02	3.54	2.16	2.78	3.51
Artisanal Axim	-	Ghana	2013	2.14	2.35	3.69	2.14	3.07	2.62
Artisanal Westpoint	-	Liberia	2011	2.12	2.43	2.36	2.12	2.58	2.54
Octopus	-	Kenya	2013	2.05	2.49	2.76	2.05	3.16	2.39
Shrimp Industrial	-	Colombia	2010	2.04	3.15	4.22	2.04	3.09	3.92
Tombo	-	Sierra Leone	2013	2.00	1.91	3.11	2.00	2.52	2.34
Shrimp Artisanal	-	Colombia	2010	1.94	2.71	4.10	1.94	2.99	3.59
Lake Chiuta	-	Malawi	2013	1.81	1.50	2.70	1.81	2.11	1.70
Artisanal	-	Ghana	2010	1.65	1.70	2.60	1.65	2.08	2.12
Thanh Hoa	-	Vietnam	2011	1.54	1.84	2.72	1.54	2.06	2.08
