# Supplementary material for: The Fishery Performance Indicators: A Management Tool for Triple Bottom Line Outcomes
Source: PLoS One. 2015 May 6;10(5):e0122809. doi: 10.1371/journal.pone.0122809 (PMC4422616; doi:10.1371/journal.pone.0122809)
Supplement: S1 Dataset — Metric scores and score quality ratings for reported case studies. (PDF) [file pone.0122809.s002.pdf]

Fishery	Country	FPIVers	Year	Fishtype	SingleMulti	OFOverfishe	OFDegOverfi	OFRebuild	OFRegMort
Spencer Gulf Praw	Australia	1.1	2011	Crustacean	Single	5	5	5	5
Pabna Sadullaspra	Bangladesh	1.1	2010	Finfish	Multi	3	3	2	5
Beel Chatra	Bangladesh	1.1	2010	Finfish	Multi	3	3	2	5
Kailin Nadi	Bangladesh	1.1	2010	Finfish	Multi	3	3	2	5
Shrimp Artisanal	Colombia	1.1	2010	Crustacean	Single	1	1	1	5
Shrimp Industrial	Colombia	1.1	2010	Crustacean	Single	1	1	1	5
Industrial Tuna	Ecuador	1.2	2013	Finfish	Multi	4	4	4	5
Artisanal Sole and	Gambia	1.0.1	2010	Finfish	Multi	3	4	3	5
TRY Oysters	Gambia	1.1	2010	Shellfish	Single	1	3	2	5
Artisanal	Ghana	1.1	2010	Finfish	Multi	2	3	2	4
Artisanal Axim	Ghana	1.1	2013	Finfish	Multi	1	2	1	5
Lobster (Nephrops	Iceland	1.1	2010	Crustacean	Single	5	5	5	5
Lesser Sunda Artisa	Indonesia	1.1	2010	Finfish	Multi	3	3	2	5
Blue Crab	Indonesia	1.0.1	2010	Crustacean	Single	1	2	1	4
Longline Tuna	Indonesia	1.2	2013	Finfish	Multi	3	4	4	5
Suruga Pink Shrim	Japan	1.1	2010	Crustacean	Single	5	5	5	5
Tokyo Bay	Japan	1.2	2013	Finfish	Multi	3	3	2	5
Artisanal Shimoni	Kenya	1.1	2013	Finfish	Multi	2	2	1	5
Octopus	Kenya	1.1	2013	Cephalopod	Single	1	2	1	5
Artisanal Westpoir	Liberia	1.0.1	2011	Finfish	Multi	1	2	1	4
Artisanal Robertsp	Liberia	1.1	2013	Finfish	Multi	3	3	4	5
Semi-Industrial	Liberia	1.1	2013	Finfish	Multi	3	3	4	5
Lake Chiuta	Malawi	1.1	2013	Finfish	Multi	4	4	3	4
Skipjack Tuna	Maldives	1.1	2013	Finfish	Single	5	5	5	5
La Paz Bivalves	Mexico	1.2	2011	Shellfish	Multi	1	1	1	5
La Paz Bivalves	Mexico	1.2	2013	Shellfish	Multi	1	1	3	5
La Paz Bay Chocola	Mexico	1.2	2013	Shellfish	Single	1	3	3	4
Purse Seine Tuna	Mexico	1.2	2013	Finfish	Single	3	3	3	5
Central Zone	Morocco	1.1	2013	Finfish	Multi	3	2	1	4.5
Southern Zone	Morocco	1.1	2013	Finfish	Multi	3	4	5	3

Fishery	OFSelectivity	OFIUU	OFHabitat	OFCertified	OHLandings	OHExCapacit	OHSeasonLe	OHSafety	OAAssetEarn
Spencer Gulf Praw	1	5	4	5	4	5	2	5	3
Pabna Sadullaspra	5	3	3	1	4	2	5	5	3
Beel Chatra	5	5	4	1	5	3	5	5	5
Kailin Nadi	5	4	4	1	5	1	5	5	1
Shrimp Artisanal	3	2	4	1	1	2	3	4	1
Shrimp Industrial	3	3	4	1	1	2	3	4	1
Industrial Tuna	4	4	5	1	4	3	4	3	1
Artisanal Sole and	2	5	5	1	4	3	5	1	1
TRY Oysters	3	4	5	1	1	2	2	1	1
Artisanal	1	3	3	1	1	3	5	5	
Artisanal Axim	4	2	3	2	2	2	5	5	1
Lobster (Nephrops	5	5	5	5	5	5	5	5	5
Lesser Sunda Artisa	5	3	3	1	2	2	5	1	1
Blue Crab	5	3	3	1	2	1	2	3	1
Longline Tuna	5	2	5	1	4	1	5	3	1
Suruga Pink Shrim	5	5	4	1	4	4	5	5	1
Tokyo Bay	5	4	3	1	4	4	5	2	1
Artisanal Shimoni	5	2	3	1	1	1	5	2	1
Octopus	5	1	3	1	1	1	5	3	1
Artisanal Westpoir	3	4	3	1	2	4	5	5	2
Artisanal Robertsp	4	4	4	1	4	3	5	1	1
Semi-Industrial	4	4	4	1	4	3	5	2	1
Lake Chiuta	3	2	3	1	4	3	5	5	3
Skipjack Tuna	4	4	5	5	4	3	5	5	1
La Paz Bivalves	5	1	4	1	1	1	5	5	1
La Paz Bivalves	5	4	3	1	3	1	1	5	1
La Paz Bay Chocola	5	2	5	1	2	3	3	5	1
Purse Seine Tuna	4	3	5	1	4	4	5	5	1
Central Zone	4	4	5	1	3	1	5		2
Southern Zone	3.5	5	5	1	5	5	5		5

Fishery	OATotReven	OAAssetVal	OALoanRate	OALoanSour	OACapFuncti	ORAnnRevVo	ORAnnLandV	ORIntraAnnL	ORAnnPriceV
Spencer Gulf Praw	2	2	4	3	4	4	5	4	4
Pabna Sadullaspra	5	5		1	2	4	3	2	5
Beel Chatra	5	5		1	3	4	3	3	5
Kailin Nadi	5	5		1	2	4	3	2	5
Shrimp Artisanal	2	5	2	2	2	2	2	3	4
Shrimp Industrial	2	5	3	3	2	2	2	3	4
Industrial Tuna	3	5	5	4	4	2	3	5	2
Artisanal Sole and	2	5	2	2	3	2	2	4	5
TRY Oysters	4	1	2	1	3	4	4	3	5
Artisanal			2	2	3	3	2	3	3
Artisanal Axim	1	5	1	2	3	5	5	2	2
Lobster (Nephrops	5	2	4	5	4	4	5	2	5
Lesser Sunda Artisa	4	5	2	1	3	4	4	4	4
Blue Crab	3	5	1	2	3	3	3	3	4
Longline Tuna	5	5	5	4	2	2	3	1	2
Suruga Pink Shrimp	5	5	4	3	4	2	1	3	1
Tokyo Bay	2	2	3	4	3	3	2		3
Artisanal Shimoni	5	5	2	2	2	3	3	1	3
Octopus	5	5		2	3	2	2	2	3
Artisanal Westpoir				2	2	2	1	3	2
Artisanal Robertsp	4	5	4	2	3	3	3	2	3
Semi-Industrial	4	5	4	2	3	3	3	2	3
Lake Chiuta	3	4	2	2	3	3	4	3	3
Skipjack Tuna	5	5	4	4	5	2	2	2	3
La Paz Bivalves	2	5	1	2	2	1	1	1	3
La Paz Bivalves	1	5	1	2	2	1	1	1	3
La Paz Bay Chocola	2	5	1	2	2	2	2	2	2
Purse Seine Tuna	5	5	5	5	4	3	4	1	2
Central Zone	3	4	3	3	3.5	5	5	2	4
Southern Zone	5	5	3	3	3.5	2	2	2	2

Fishery	ORIntrAnnPr	ORSpatialPri	ORContest	OOEarnings	OOWage	OOEdAccess	OOHealthAc	OOSocialStar	OONonreside
Spencer Gulf Praw	4	5	5	4	5	5	5	5	4
Pabna Sadullaspra	3		4	1	1	2	3	2	5
Beel Chatra	3		4	5	5	5	3	5	5
Kailin Nadi	3		4	2	2	4	3	3	5
Shrimp Artisanal	3	5	3	3	3	2	2	2	5
Shrimp Industrial	3	5	3	4	4	4	3	4	5
Industrial Tuna	4	5	4	5	5	5	4	5	5
Artisanal Sole and	5	5	4	5	5	3	3	4	3
TRY Oysters	4	5	3	1	1	1	1	1	4
Artisanal	3	3	1	3	3	3	2	5	2
Artisanal Axim	2	4	4	5	5	4	2	3	5
Lobster (Nephrops		5	4	4	4	5	5	4	4
Lesser Sunda Artis	4	1	3	4	4	4	2	3	4
Blue Crab	4	5	4	5	5	4	2	5	3
Longline Tuna	2	4	5	5	5	5	5	3	5
Suruga Pink Shrim	3	5	5	5	4	4	5	4	5
Tokyo Bay			4		4	5	5	5	5
Artisanal Shimoni	1	2	5	4	4	2	2	3	4
Octopus	2	4	5	5	5	3	3	4	5
Artisanal Westpoir			5	5	5	5	2	2	2
Artisanal Robertsp	2	2	3	3	4	4	2	5	4
Semi-Industrial	2	2	3	4	4	4	2	5	1
Lake Chiuta	4	4	4	3	2	3	2	2	5
Skipjack Tuna	3	5	5	5	5	3	3	5	5
La Paz Bivalves	2	3	2	2	2	3	2	2	5
La Paz Bivalves	2	3	3	2	5	4	3	2	5
La Paz Bay Chocola	4	2	3	3	4	4	3	3	5
Purse Seine Tuna	2	5	5	5	5	5	4	4	5
Central Zone	3	4	3	5	5	5	5	4	5
Southern Zone	4	4	3	5	5	5	5	5	3

Fishery	OCEarnings	OCWage	OCEdAccess	OCHealthAcc	OCSocialStan	OCNonreside	OCExperienc	OCAge	OSPrice
Spencer Gulf Praw	3	5	5	5	4	4	5	4	2
Pabna Sadullaspra	1	1	1	3	2	5	4	3	5
Beel Chatra	5	5	4	3	3	5	4	5	5
Kailin Nadi							5	2	5
Shrimp Artisanal							5	5	3
Shrimp Industrial	3	3	3	2	3	5	5	5	3
Industrial Tuna	4	4	4	4	3	5	5	4	5
Artisanal Sole and	4	4	2	3	3	2	4	5	5
TRY Oysters	1	1	1	1	1	4	5	3	5
Artisanal	3	3	4	4	2	2	5	5	1
Artisanal Axim	4	5	4	2	2	5	5	5	3
Lobster (Nephrops	5	5	5	5	3	3	4	5	5
Lesser Sunda Artis	3	3	2	2	2	3	4	5	4
Blue Crab	5	5	2	2	2	3	4	5	2
Longline Tuna	5	4	4	2	2	5	5	4	5
Suruga Pink Shrim	3	4	5	5	3	4	4	4	4
Tokyo Bay		3	4	5	3	5	4	2	5
Artisanal Shimoni	4	4	2	2	2	4	4	3	4
Octopus	4	4	2	2	2	5	3	3	4
Artisanal Westpoir	2	2	4	2	2	2	4	2	
Artisanal Robertsp	4	4	4	2	4	4	5	5	3
Semi-Industrial	4	4	4	2	4	2	5	4	3
Lake Chiuta	4	4	3	3	3	5	5	3	2
Skipjack Tuna	5	5	4	3	3	5	4	4	5
La Paz Bivalves	2	2	3	2	2	5	5	4	4
La Paz Bivalves	2	5	4	3	2	5	5	4	5
La Paz Bay Chocola	2	4	3	3	2	5	5	3	2
Purse Seine Tuna	5	5	5	4	4	5	4	5	4
Central Zone	5	5	3	5	2	5	4	5	5
Southern Zone	5	5	4	5	3	4	4	4	2

Fishery	OSFinalMark	OSExport	OSFinalWeal	OSRelativePr	OSUSEUExpo	OSMargin	OPYield	OPShrink	OPUtilization
Spencer Gulf Praw	5	1	5	5	5	4	5	5	4
Pabna Sadullaspra	3	2	1	2	2	3	4	3	5
Beel Chatra	3	2	1	2	2	3	4	3	5
Kailin Nadi	3	2	1	2	2	3	4	3	5
Shrimp Artisanal	3	2	2	2	5	2	4	4	3
Shrimp Industrial	4	4	5	3	5	3	4	4	3
Industrial Tuna	3	3	4	3	5	3	5	5	5
Artisanal Sole and	3	2	5	2	3	4	3	2	2
TRY Oysters	2	1	1	1	1		2	1	4
Artisanal	3	2	1	1	2	2	1	2	5
Artisanal Axim	3	2	1	2	4	2	5	3	5
Lobster (Nephrops	4	5	5	3	5	4	4	4	4
Lesser Sunda Artis	3	4	4	4	4	5	4	1	2
Blue Crab	4	5	5	4	5	3	4	3	5
Longline Tuna	4	5	5	2	5	5	3	4	2
Suruga Pink Shrim	4	1	4	4	4	4	5	5	3
Tokyo Bay	4	1	5	5	5	5	5	5	5
Artisanal Shimoni	3	2	1	1	2	2	4	4	5
Octopus	5	5	4	2	4	5	5	4	5
Artisanal Westpoir	4	2	5	3	2	1	3	4	5
Artisanal Robertsp	3	1	1	1	1	3	5	4	5
Semi-Industrial	3	1	1	1	1	3	5	4	5
Lake Chiuta	3	1	1	1	1	3	2	3	4
Skipjack Tuna	3	4	5	3	5	2	4	4	2
La Paz Bivalves	4	1	2	2	1	2	4	5	2
La Paz Bivalves	3	1	2	2	1	3	3	4	1
La Paz Bay Chocola	4	1	2	3	1	3	4	4	5
Purse Seine Tuna	5	5	5	5	5	5	5	4	4
Central Zone	1.5	3	3	5	5	4	3	2	2
Southern Zone	3	4	3	5	5	4	4	2	2

Fishery	OPImprovem	OPSanitation	OPSupport	OBLoanRate	OBLoanSour	OBCapFuncti	OMEarnings	OMWage	OMEdAccess
Spencer Gulf Praw	4	5	5	4	3	4	4	5	5
Pabna Sadullaspra	1	2	3		1	1	5	5	4
Beel Chatra	1	2	3		1	1	5	5	4
Kailin Nadi	1	2	3		1	1	5	5	4
Shrimp Artisanal	2	5	3	4	4	2	5	5	5
Shrimp Industrial	5	5	3	4	4	2	5	5	5
Industrial Tuna	5	4	4	5	4	3	5	5	5
Artisanal Sole and	1	2	4	4	4	4	5	5	4
TRY Oysters	1	2	5	2	1	1	1	1	1
Artisanal	2	2	3	2	2	4	4	4	4
Artisanal Axim	2	3	2	2	2	4	5	5	4
Lobster (Nephrops	5	5	5	4	4	4	5	4	5
Lesser Sunda Artis	3	4	4	3	3	3	5	5	5
Blue Crab	5	3	4	4	2	4	5	3	5
Longline Tuna	5	1	2	4	4	4	5	5	5
Suruga Pink Shrim	5	5	5	4	4	3	3	3	5
Tokyo Bay	5	5	5	4	4	3		4	5
Artisanal Shimoni	1	2	2	2	2	3	4	4	2
Octopus	4	5	3		3	4	5	5	4
Artisanal Westpoir	2	1	2		2	4	3	5	4
Artisanal Robertsp	2	2	1	4	2	5	5	5	3
Semi-Industrial	2	2	1	4	2	4	5	5	3
Lake Chiuta	1		1	2	2	5	2	3	2
Skipjack Tuna	2	3	3	4	3	2	5	5	4
La Paz Bivalves	1	3	5	5	4	2	1	1	3
La Paz Bivalves	1	3	5	5	4	2	1	1	3
La Paz Bay Chocola	3	3	5	5	4	3	5	5	5
Purse Seine Tuna	5	3	5	5	5	4	5	5	5
Central Zone	3		4	3	3	1	5	5	5
Southern Zone	3		1	3	3	4	5	5	5

Fishery	OMHealthAc	OMSocialSta	OMNonresid	OWEarnings	OWWage	OWEdAccess	OWHealthAc	OWSocialSta	OWNonresid
Spencer Gulf Praw	5	4	5	3	3	5	5	3	4
Pabna Sadullaspra	3	4	5	2	2	2	3	1	5
Beel Chatra	3	4	5	2	2	2	3	1	5
Kailin Nadi	3	4	5	2	2	2	3	1	5
Shrimp Artisanal	4	5	5	3	3	5	4	3	5
Shrimp Industrial	4	5	5	3	3	5	4	3	5
Industrial Tuna	4	5	4	3	3	4	4	2	5
Artisanal Sole and	4	4	2	3	4	4	3	3	3
TRY Oysters	1	1	4	1	1	1	1	1	4
Artisanal	4	2	5	4	4	1	3	2	3
Artisanal Axim	2	2	5	5	5	4	2	2	5
Lobster (Nephrops	5	4	3	3	4	4	5	2	3
Lesser Sunda Artis	4	3	4	3	2	3	3	3	5
Blue Crab	5	3	4	3	3	3	3	3	5
Longline Tuna	5	4	3	3	5	3	4	3	5
Suruga Pink Shrim	5	4	5	3	3	4	5	3	5
Tokyo Bay	5	5	5		3	5	5	4	5
Artisanal Shimoni	2	2	4	4	4	2	2	2	4
Octopus	4	5	2	5	4	3	3	3	4
Artisanal Westpoir	3	2	1	3	3	4	3	2	1
Artisanal Robertsp	2	5	5	4	3	3	2	3	4
Semi-Industrial	2	5	2	4	3	3	2	3	2
Lake Chiuta	2	3	2	2	2	3	2	3	4
Skipjack Tuna	3	4	5	3	3	3	2	3	2
La Paz Bivalves	3	4	5	1	1	2	2	2	5
La Paz Bivalves	3	4	5	1	1	2	2	2	5
La Paz Bay Chocola	4	5	4	2	5	4	2	2	5
Purse Seine Tuna	4	5	5	5	5	4	3	5	5
Central Zone	5	5	5	4	4	2.5	2	3	5
Southern Zone	5	5	3	4	4	2	2	3	3

Fishery	OWExperien	I_EPI	IEDisease	IEDisasters	IEPollutionSh	IEChronicSto	IEChronicCor	IGQuality	IGResponsive
Spencer Gulf Praw	5	5	5	5	5	5	5	5	5
Pabna Sadullaspra	2	1	5	4	3	5	5	2	1
Beel Chatra	2	1	5	5	5	5	5	2	1
Kailin Nadi	2	1	4	4	5	5	5	2	1
Shrimp Artisanal	5	4	5	5	5	4	5	3	1
Shrimp Industrial	5	4	5	5	5	4	5	3	1
Industrial Tuna	4	3	5	5	5	5	5	1	2
Artisanal Sole and	4	2	5	5	5	5	5	2	2
TRY Oysters	3	2	5	5	5	5	5	2	2
Artisanal	5	2	5	5	4	3	4	3	3
Artisanal Axim	5	2	1	1	2	5	5	3	3
Lobster (Nephrops	4	4	5	5	5	5	5	5	5
Lesser Sunda Artis	4	2	5	4	5	5	5	2	2
Blue Crab	4	3	5	3	4	3	5	2	2
Longline Tuna	3	2	5	4	5	5	5	2	2
Suruga Pink Shrim	4	5	5	5	3	4	5	5	5
Tokyo Bay		4	5	5	5	3	4	5	5
Artisanal Shimoni	4	3	5	3	5	5	5	2	2
Octopus	3	3	5	3	5	5	5	2	2
Artisanal Westpoir	4	2	4	5	5	4	5	3	3
Artisanal Robertsp	5	2	5	5	2	5	5	2	2
Semi-Industrial	5	2	5	5	2	5	5	2	2
Lake Chiuta	5	2	3	3	5	5	5	3	4
Skipjack Tuna	3	5	5	4	5	5	5	3	2
La Paz Bivalves	5	2	4	2	5	4	4	3	2
La Paz Bivalves	5	2	4	2	5	4	4	3	2
La Paz Bay Chocola	3	2	4	5	5	5	5	3	2
Purse Seine Tuna	4	2	5	5	5	5	5	3	2
Central Zone	1.5	1	4	3	4	5	4	3	2
Southern Zone	1.5	1	4	5	4	5	4	3	2

Fishery	INIEF	INGDP	IAProportion	IATransfer	IASecurity	IADurability	IAFlexibilty	IAExclusivity	IHProportion
Spencer Gulf Praw	5	5	5	5	5	5	1	5	1
Pabna Sadullaspra	2	1	4	4	4	2	4	3	4
Beel Chatra	2	1	5	4	4	2	4	4	5
Kailin Nadi	2	1	5	4	4	2	4	4	5
Shrimp Artisanal	4	3	1	1	1	3	3	1	1
Shrimp Industrial	4	3	4	1	1	3	3	1	1
Industrial Tuna	1	2	2	1	5	5	4	3	1
Artisanal Sole and	3	1	1	1	1	1	3	1	1
TRY Oysters	3	1	4	2	3	4	3	3	2
Artisanal	3	1	4	1	2	3	1	1	1
Artisanal Axim	3	1	4	1	4	5	2	2	1
Lobster (Nephrops	4	5	5	4	3	3	4	4	5
Lesser Sunda Artisa	2	2	3	1	2	3	3	2	1
Blue Crab	3	2	1		4	3	3	3	1
Longline Tuna	2	2	1		5	5	5	1	1
Suruga Pink Shrimp	4	5	5	2	5	5	3	4	5
Tokyo Bay	5	5	5	1	5	4	2	3	1
Artisanal Shimoni	2	1	4	1	4	3	3	2	1
Octopus	2	1	4	1	3	3	3	2	1
Artisanal Westpoir		1	4	1	4	2	1	2	1
Artisanal Robertsp	1	1	4	1	2	3	2	2	1
Semi-Industrial	1	1	4	1	2	3	2	2	1
Lake Chiuta	2	1	4	1	4	5	3	3	1
Skipjack Tuna	1	3	5	1	3	5	5	5	1
La Paz Bivalves	4	3	1		1	1	4	1	1
La Paz Bivalves	4	3	4	2	5	5	2	4	4
La Paz Bay Chocola	4	3	4	2	5	5	3	3	3
Purse Seine Tuna	4	3	2	1	5	5	5	4	5
Central Zone	3	2	5	4	3	3	3	4	1
Southern Zone	3	2	5	4	3	3	2	4	5

Fishery	IHTransfer	IHSecurity	IHDurability	IHFlexibility	IHExclusivity	IOParticipatio	IOMgmtInflu	IOBusInfluen	IPDays
Spencer Gulf Praw						5	3	3	4
Pabna Sadullaspra	1	3	1	4	3	4	5	5	4
Beel Chatra	1	3	1	4	4	3	5	5	4
Kailin Nadi	1	3	1	4	4	5	5	5	4
Shrimp Artisanal						2	2	1	2
Shrimp Industrial						5	2	1	2
Industrial Tuna						4	3	4	3
Artisanal Sole and						5	2	2	1
TRY Oysters	1	2	1	2	2	2	2	2	2
Artisanal						3	2	3	3
Artisanal Axim						5	4	2	2
Lobster (Nephrops	3	3	3	4	4	5	3	5	2
Lesser Sunda Artis						4	3	2	2
Blue Crab						4	3	4	3
Longline Tuna						2	2	2	4
Suruga Pink Shrim	1	5	5	5	5	5	4	4	5
Tokyo Bay						5	3	2	4
Artisanal Shimoni						5	4	1	4
Octopus						5	4	1	4
Artisanal Westpoir						2	1	5	2
Artisanal Robertsp						5	4	1	4
Semi-Industrial						5	4	1	4
Lake Chiuta						3	4	5	5
Skipjack Tuna						2	4	3	5
La Paz Bivalves						2	1	2	2
La Paz Bivalves	4	4	2	2	3	4	4	2	4
La Paz Bay Chocola	4	4	2	4	3	4	4	2	2
Purse Seine Tuna	4	5	3	5	4	3	3	1	4
Central Zone						4	3	5	5
Southern Zone	2	2	2.5	3	4	4	4	5	5

Fishery	IPFinancial	ICLeadership	ICCohesion	IWBusInfluer	IWMgmtInflu	IWHarvest	IWPostHarve	IMExpenditu	IMEnforceme
Spencer Gulf Praw	5	5	5	1	1	1	2	5	5
Pabna Sadullaspra	3	3	5	1	1	1	1	4	5
Beel Chatra	3	5	5	1	1	1	1	5	5
Kailin Nadi	3	4	5	1	1	1	1	5	5
Shrimp Artisanal	1	1	4	1	1	1	4	5	1
Shrimp Industrial	2	2	4	1	1	1	4	5	2
Industrial Tuna	1	4	5	1	1	1	4	5	3
Artisanal Sole and	1	3	4	2	2	1	2	5	2
TRY Oysters	1	5	3	5	5	5	5	5	2
Artisanal	2	2	3	4	1	1	4	4	2
Artisanal Axim	1	5	5	3	1	1	5	5	4
Lobster (Nephrops	4	4	4	1	1	1	2	5	5
Lesser Sunda Artis	1	4	3	1	2	1	5	5	2
Blue Crab	1	3	3	2	2	2	2	5	2
Longline Tuna	2	4	5	1	2	1	4	5	1
Suruga Pink Shrim	4	5	5	1	1	1	3	4	5
Tokyo Bay	1	5	5	2	2	2	2		5
Artisanal Shimoni	2	3	4	1	1	2	2	2	3
Octopus	3	3	5	1	1	2	1	3	3
Artisanal Westpoir	2	5	3					3	4
Artisanal Robertsp	1	5	3	3	1	1	4	1	3
Semi-Industrial	1	5	3	3	1	1	4	1	3
Lake Chiuta	1	5	4	3	1	2	3	5	5
Skipjack Tuna	1	5	4	1	1	1	2	5	4
La Paz Bivalves	1	2	3	2	1	1	1	5	2
La Paz Bivalves	3	4	4	2	1	1	1	1	4
La Paz Bay Chocola	4	4	4	2	2	1	1	4	3
Purse Seine Tuna	1	5	5	1	1	1	1	5	4
Central Zone	2	4	3					1	2
Southern Zone	2	4	3					1	2

Fishery	IMJurisdictio	IMSubsidies	IDAvailability	IDAnalysis	ILMPAs	ILSpatial	ILTACs	ITPricing	ITPriceAvaila
Spencer Gulf Praw	5	5	5	4	5	5	5	1	3
Pabna Sadullaspra	3	1	3	2	5	5	1	5	2
Beel Chatra	3	1	3	2	5	5	1	5	2
Kailin Nadi	3	1	4	2	5	5	1	5	2
Shrimp Artisanal	5	4	2	2	5	1	2	4	4
Shrimp Industrial	5	4	3	2	5	1	2	4	4
Industrial Tuna	4	3	4	3	2	1	1	5	4
Artisanal Sole and	1	5	2	2	1	1	1	1	3
TRY Oysters	5	5	1	1	1	3	1	1	3
Artisanal	3	4	3	4	1	1	1	5	2
Artisanal Axim	2	2	4	2	1	4	1	5	3
Lobster (Nephrops	5	5	5	5	2	1	5	1	5
Lesser Sunda Artis	3	4	2	3	2	4	1	1	1
Blue Crab	4	5	3	2	2	1	1	4	2
Longline Tuna	3	3	2	1	2	1	1	1	2
Suruga Pink Shrim	5	4	4	3	1	5	5	5	4
Tokyo Bay	3	4	4	3	2	5	3	5	2
Artisanal Shimoni	4	5	2	1	2	4	1	4	2
Octopus	5	5	3	1	2	5	1	1	2
Artisanal Westpoir	3	5	2	2	1	1	3	4	2
Artisanal Robertsp	3	5	2	2	2	2	1	2	1
Semi-Industrial	3	5	2	2	2	2	1	2	1
Lake Chiuta	5	5	3	2	1	4	1	3	1
Skipjack Tuna	4	2	3	4	1	4	1	1	3
La Paz Bivalves	5	3	2	1	1	1	1	1	2
La Paz Bivalves	5	3	4	4	5	4	1	1	2
La Paz Bay Chocola	5	3	3	4	2	4	3	2	2
Purse Seine Tuna	5	4	3	3	1	1	1	1	3
Central Zone	5	2	4.5	3	2	2	1	2	4
Southern Zone	3	2	4.5	3	2	2	1	2	4

Fishery	ITBuyers	ITVerticalInte	ITTariffs	ITNonTariffs	IIShipping	IIRoads	IITechnology	IIExtenstion	IIElectricity
Spencer Gulf Praw	4	2	5	5	5	3	5	5	5
Pabna Sadullaspra	5	1		2	4	2	2	4	2
Beel Chatra	5	1		2	4	2	3	4	2
Kailin Nadi	5	1	5	2	4	2	2	4	2
Shrimp Artisanal	4	1	5	5	4	3	2	2	4
Shrimp Industrial	4	2	5	4	4	3	4	2	4
Industrial Tuna	4	2	1	4	5	3	5	5	4
Artisanal Sole and	1	1	4	4	4	2	2	3	3
TRY Oysters	5	5	5	1	3	3	2	4	4
Artisanal	5	1	5	3	3	4	2	3	2
Artisanal Axim	3	1	2	5	4	3	3	2	5
Lobster (Nephrops	2	5	5	5	4	4	5	5	5
Lesser Sunda Artis	1	4	3	4	4	2	2	1	1
Blue Crab	5	3	1	4	4	2	4	1	4
Longline Tuna	1	1	5	1	2	1	1	3	3
Suruga Pink Shrim	5	1	5	5	4	5	5	4	5
Tokyo Bay	5	1	2	4	4	5	5	4	5
Artisanal Shimoni	5	1	5	5	4	1	2	3	2
Octopus	2	1	5	5	4	1	2	3	2
Artisanal Westpoir	5	4	4	5	3	1	4	2	1
Artisanal Robertsp	5	2	2	5	1	1	2	2	2
Semi-Industrial	5	2	2	5	1	1	2	2	2
Lake Chiuta	5	4	1	1	1	3	2	2	1
Skipjack Tuna	3	1	5	5	2		4	2	3
La Paz Bivalves	4	2	4	4	2	3	2	2	3
La Paz Bivalves	4	2	4	4	2	3	3	5	3
La Paz Bay Chocola	4	1	4	4	2	3	3	2	3
Purse Seine Tuna	3	1	3	5	5		5	5	5
Central Zone	1	1	4	4	3.5	3	4	3.5	4.5
Southern Zone	1	4	4	4	1	2	4	3.5	4.5

Fishery	IIIce	QOFCertified	QOFOverfish	QOFDegOver	QOFRebuild	QOFRegMort	QOFSelectivi	QOFIUU	QOFHabitat
Spencer Gulf Praw	5	A	A	A	A	A	A	A	A
Pabna Sadullaspra	2	A	B	B	B	A	A	B	B
Beel Chatra	2	A	B	B	B	A	A	A	B
Kailin Nadi	2	A	B	B	B	A	A	B	B
Shrimp Artisanal	5	A	A	B	A	B	A	B	B
Shrimp Industrial	5	A	A	B	A	B	A	B	B
Industrial Tuna	5	A	B	B	B	A	B	A	A
Artisanal Sole and	4	A	B	C	C	A	A	A	A
TRY Oysters	1	A	A	B	B	A	B	B	A
Artisanal	3	A	B	B	B	C	B	B	C
Artisanal Axim	4	B	A	B	B	A	B	B	B
Lobster (Nephrops	5	A	A	A	A	B	A	A	B
Lesser Sunda Artis	2	A	B	B	B	A	A	B	C
Blue Crab	5	A	A	B	B	B	A	B	B
Longline Tuna	1	A	B	B	B	A	A	B	A
Suruga Pink Shrim	5	A	B	B	B	A	A	A	B
Tokyo Bay	5	A	B	B	B	A	A	B	A
Artisanal Shimoni	1	A	B	B	B	A	A	B	B
Octopus	5	A	C	C	C	A	A	A	B
Artisanal Westpoir	2	A	B	B	B	A	B	A	B
Artisanal Robertsp	2	A	C	C	C	B	B	B	B
Semi-Industrial	2	A	C	C	C	B	B	B	B
Lake Chiuta	1	A	C	C	C	A	C	C	C
Skipjack Tuna	3	A	A	A	A	A	B	B	A
La Paz Bivalves	4	A	A	A	A	A	A	A	A
La Paz Bivalves	4	A	A	B	B	A	A	A	B
La Paz Bay Chocola	4	A	A	B	B	A	A	B	A
Purse Seine Tuna	5	A	B	B	B	A	B	B	A
Central Zone	5	A	A	B	C	C	C	C	A
Southern Zone	1	A	A	B	C	C	C	C	A

Fishery	QOHLanding	QOHExCapac	QOHSeasonL	QOHSafety	QOAAssetEa	QOATotReve	QOAAssetVa	QOALoanRat	QOALoanSou
Spencer Gulf Praw	B	A	A	A	B	A	A	B	B
Pabna Sadullaspra	A	B	B	C	A	A	A		A
Beel Chatra	A	B	B	C	B	A	A		A
Kailin Nadi	A	B	B	C	A	A	A		A
Shrimp Artisanal	A	A	A	B	A	A	A	A	A
Shrimp Industrial	A	A	A	B	A	A	A	A	A
Industrial Tuna	A	A	B	A	A	C	A	A	B
Artisanal Sole and	C	B	A	C	A	B	A	B	B
TRY Oysters	A	C	A	B	A	A	A	A	A
Artisanal	B	C	B	B				C	A
Artisanal Axim	A	A	A	B	A		B	B	A
Lobster (Nephrops	A	B	A	A	B	B	B	B	B
Lesser Sunda Artisa	B	B	A	B	A	B	B	B	A
Blue Crab	B	A	B	B	B	B	A	B	A
Longline Tuna	B	A	A	B	B	B	A	A	A
Suruga Pink Shrim	A	A	A	A	B	B	B	B	A
Tokyo Bay	B	B	A	A	C	A	C	B	B
Artisanal Shimoni	B	C	A	B	A	A	B		B
Octopus	B	C	A	B	C	B	A		B
Artisanal Westpoir	B	B	A	B	B				B
Artisanal Robertsp	C	C	A	B	C	B	B	B	B
Semi-Industrial	C	C	A	B	C	B	B	B	B
Lake Chiuta	C	C	C	C	C	C	C	C	C
Skipjack Tuna	A	B	A	B	A	B	B	B	B
La Paz Bivalves	A	A	A	A	A	C	A	A	B
La Paz Bivalves	A	A	A	A	A	C	A	A	A
La Paz Bay Chocola	B	B	A	A	B	B	A	B	A
Purse Seine Tuna	B	B	B	B	B	C	A	B	B
Central Zone	A	A	A	A	A	A	B	A	A
Southern Zone	A	A	A	A	A	A	B	A	A

Fishery	QOACapFunc	QORAnnRevV	QORAnnLanc	QORIntraAnr	QORAnnPrice	QORIntrAnnI	QORSpatialP	QORContest	QOOEarnings
Spencer Gulf Praw	A	A	A	A	A	A	A	A	B
Pabna Sadullaspra	A	B	B	C	B	B		B	B
Beel Chatra	A	B	B	C	B	B		A	A
Kailin Nadi	A	A	A	C	A	B		B	B
Shrimp Artisanal	A	A	A	A	A	A	B	A	A
Shrimp Industrial	A	A	A	A	A	A	B	A	A
Industrial Tuna	B	B	A	A	B	A	A	B	A
Artisanal Sole and	B	B	B	B	A	A	A	A	A
TRY Oysters	B	C	C	C	B	C	B	B	A
Artisanal	B	c	c	c	c	c	c	a	c
Artisanal Axim	B	B	A	B	A	A	B	B	B
Lobster (Nephrops	A	A	A	A	A		B	B	B
Lesser Sunda Artis	B	C	B	C	C	C	C	C	B
Blue Crab	A	B	B	B	B	B	B	A	B
Longline Tuna	B	C	C	B	C	C	C	A	A
Suruga Pink Shrim	A	A	A	C	A	C	A	A	A
Tokyo Bay	A	A	A		A			A	
Artisanal Shimoni	B							A	B
Octopus	B							A	B
Artisanal Westpoir	B		A		B			B	A
Artisanal Robertsp	B	C	C	C	C	B	C	B	C
Semi-Industrial	B	C	C	C	C	B	C	B	C
Lake Chiuta	C	C	C	C	C	C	C	C	B
Skipjack Tuna	A	B	A	A	B	C	B	B	A
La Paz Bivalves	A	B	B	C	B	C	C	B	C
La Paz Bivalves	A	B	B	C	B	C	C	B	A
La Paz Bay Chocola	A	B	B	A	A	A	A	B	B
Purse Seine Tuna	B	B	A	A	B	C	B	B	A
Central Zone	A	A	A	A	A	A	A	A	B
Southern Zone	A	A	A	A	A	A	A	A	B

Fishery	QOOWage	QOOEdAcces	QOOHealthA	QOOSocialSt	QOONonresi	QOCEarnings	QOCWage	QOCEdAcces	QOCHealthA
Spencer Gulf Praw	A	A	A	A	A	B	A	A	A
Pabna Sadullaspra	B	B	B	A	A	C	C	C	B
Beel Chatra	A	A	B	A	A	A	A	B	B
Kailin Nadi	B	B	B	B	A				
Shrimp Artisanal	A	A	A	A	A				
Shrimp Industrial	A	A	A	A	A	A	A	A	A
Industrial Tuna	A	A	A	A	A	A	A	B	A
Artisanal Sole and	A	B	B	A	A	B	A	B	B
TRY Oysters	A	A	A	B	A	A	A	A	A
Artisanal	c	b	b	a	c	c	c	b	b
Artisanal Axim	A	A	B	B	A	B	A	B	B
Lobster (Nephrops	B	A	A	A	A	A	A	A	A
Lesser Sunda Artis	B	A	A	B	B	C	C	B	B
Blue Crab	B	A	A	A	A	B	B	A	A
Longline Tuna	A	A	A	A	A	B	A	A	A
Suruga Pink Shrim	A	A	A	B	A	C	B	B	A
Tokyo Bay	A	A	A	A	A		A	B	A
Artisanal Shimoni	B	A	A	B	B	B	B	B	B
Octopus	B	B	B	B	A	B	B	B	B
Artisanal Westpoir	A	A	B	B	A	A	B	A	A
Artisanal Robertsp	B	B	C	A	B	C	C	B	C
Semi-Industrial	B	B	C	A	B	C	C	B	C
Lake Chiuta	B	A	B	A	A	C	C	C	C
Skipjack Tuna	A	B	B	A	A	A	A	B	B
La Paz Bivalves	C	B	B	A	A	C	C	B	A
La Paz Bivalves	A	B	B	A	A	A	A	B	B
La Paz Bay Chocola	B	B	B	A	A	B	B	A	A
Purse Seine Tuna	A	B	B	B	A	A	A	B	B
Central Zone	B	A	A	A	A	B	B	C	B
Southern Zone	B	A	A	A	A	B	B	C	B

Fishery	QOCSocialSta	QOCNonresid	QOCExperier	QOCAge	QOSPrice	QOSFinalMa	QOSExport	QOSFinalWe	QOSRelativeI
Spencer Gulf Praw	B	B	A	A	A	A	A	A	B
Pabna Sadullaspra	C	A	B	A	A	B	B	A	C
Beel Chatra	C	A	A	A	A	B	B	A	C
Kailin Nadi			B	A	A	B	B	A	C
Shrimp Artisanal			A	B	A	A	A	A	A
Shrimp Industrial	A	A	A	B	A	A	A	A	A
Industrial Tuna	A	A	B	A	A	A	A	A	B
Artisanal Sole and	B	B	B	A	B	B	A	B	B
TRY Oysters	B	A	A	B	B	A	A	A	A
Artisanal	c	c	b	a	a	b	b	a	a
Artisanal Axim	B	B	A	B	B	B	B	B	B
Lobster (Nephrops	A	B	A	A	A	A	A	A	B
Lesser Sunda Artis	C	C	C	C	B	B	B	B	A
Blue Crab	A	A	A	A	B	A	A	A	A
Longline Tuna	A	A	A	A	C	A	A	A	B
Suruga Pink Shrim	B	A	B	B	A	A	A	B	C
Tokyo Bay	A	A	A	A	A	A	A	A	A
Artisanal Shimoni	B	B	C	C	B	B	C	B	C
Octopus	B	B	B	B	B	B	A	B	C
Artisanal Westpoir	B	B	A	A		A	B	B	A
Artisanal Robertsp	B	B	A	A	C	B	C	C	B
Semi-Industrial	B	B	A	A	C	B	C	C	B
Lake Chiuta	C	A	A	B	C	C	C	C	C
Skipjack Tuna	A	A	B	A	B	A	A	A	A
La Paz Bivalves	A	A	A	A	C	B	A	A	B
La Paz Bivalves	A	A	A	A	A	B	A	A	B
La Paz Bay Chocola	A	A	A	B	B	A	A	A	B
Purse Seine Tuna	C	A	B	B	B	A	A	A	A
Central Zone	B	A	B	A	A	A	A	B	B
Southern Zone	B	A	B	A	A	A	A	B	B

Fishery	QOSUSEUExp	QOSMargin	QOPYield	QOPShrink	QOPUtilizatic	QOPImprove	QOPSanitatic	QOPSupport	QOBLoanRat
Spencer Gulf Praw	A	A	B	A	A	A	A	A	B
Pabna Sadullaspra	A	A	A	B	C	A	C	C	
Beel Chatra	A	A	A	B	C	A	C	C	
Kailin Nadi	A	A	A	B	C	A	C	C	
Shrimp Artisanal	A	A	B	B	A	A	A	A	A
Shrimp Industrial	A	A	B	B	A	A	A	A	A
Industrial Tuna	A	B	A	A	A	B	A	B	B
Artisanal Sole and	A	A	B	A	B	A	C	A	C
TRY Oysters	A		C	B	A	A	B	A	B
Artisanal	b	b	A	A	A	A	B	B	C
Artisanal Axim	B	B		B	B	B	B	B	B
Lobster (Nephrops	A	A	A	B	B	A	A	A	B
Lesser Sunda Artis	B	A	B	A	A	A	C	C	C
Blue Crab	A	B	B	B	A	A	B	B	B
Longline Tuna	A	B	B	B	B	A	B	A	B
Suruga Pink Shrim	C	B	A	A	B	A	A	B	B
Tokyo Bay	A	A	A	A	A	A	A	A	B
Artisanal Shimoni	B	B	C	C	B	B	B	B	
Octopus	B	C	C	C	C	C	C	C	
Artisanal Westpoir	B	A	A	A	B	B	B	A	
Artisanal Robertsp	B	B	B	B	B	B	A	B	B
Semi-Industrial	B	B	B	B	B	B	A	B	B
Lake Chiuta	C	B	C	B	B			B	C
Skipjack Tuna	A	B	B	B	A	A	B	B	C
La Paz Bivalves	A	C	B	C	C	A	B	A	B
La Paz Bivalves	A	B	B	C	B	A	B	A	B
La Paz Bay Chocola	A	A	B	B	A	B	A	A	B
Purse Seine Tuna	A	A	A	B	B	A	C	B	B
Central Zone	A	A	C	A	A	A	B	B	C
Southern Zone	A	A	C	A	A	A	B	B	C

Fishery	QOBLoanSou	QOBCapFunc	QOMEarning	QOMWage	QOMEdAcce	QOMHealthA	QOMSocialSt	QOMNonresi	QOWEarning
Spencer Gulf Praw	B	B	A	A	A	A	A	B	B
Pabna Sadullaspra	A	B	B	B	C	B	B	A	B
Beel Chatra	A	B	B	B	C	B	B	A	B
Kailin Nadi	A	B	B	B	C	B	B	A	B
Shrimp Artisanal	A	B	A	A	A	A	A	A	A
Shrimp Industrial	A	B	A	A	A	A	A	A	A
Industrial Tuna	B	A	A	A	A	A	A	A	A
Artisanal Sole and	A	B	A	B	B	B	B	B	B
TRY Oysters	B	A	A	A	A	A	B	A	A
Artisanal	B	C	C	C	B	B	A	A	C
Artisanal Axim	B	B	A	B	B	B	B	A	B
Lobster (Nephrops	A	A		A	A	A	A	A	A
Lesser Sunda Artis	B	C	C	C	C	C	C	C	C
Blue Crab	A	A	B	B	A	A	A	A	B
Longline Tuna	B	B	B	B	A	A	A	B	A
Suruga Pink Shrim	B	B	C	C	B	A	A	A	C
Tokyo Bay	B	B		A	A	A	A	B	
Artisanal Shimoni	B	B	B	B	B	B	B	B	C
Octopus	C	C	B	B	B	B	C	C	C
Artisanal Westpoir	A	A	B	B	B	B	B	B	B
Artisanal Robertsp	B	B	B	B	B	B	C	B	B
Semi-Industrial	B	B	B	B	B	B	C	B	B
Lake Chiuta	C	C	B	B	B	B	C	C	B
Skipjack Tuna	A	B	A	A	B	B	A	B	B
La Paz Bivalves	B	B	C	C	C	C	C	A	C
La Paz Bivalves	B	B	B	B	C	C	C	A	C
La Paz Bay Chocola	A	B	A	B	A	A	A	A	A
Purse Seine Tuna	B	B	A	A	B	B	C	B	A
Central Zone	C	B	A	A	A	A	A	A	B
Southern Zone	C	B	A	A	A	A	A	A	B

Fishery	QOWWage	QOWSocialSt	QOWEdAcce	QOWHealthA	QOWNonres	QOWExperie	QI_EPI	QIEDisease	QIEDisasters
Spencer Gulf Praw	B	A	A	A	B	B	A		
Pabna Sadullaspra	B	B	C	B	A	C	A	A	B
Beel Chatra	B	B	C	B	A	C	A	A	A
Kailin Nadi	B	B	C	B	A	C	A	A	A
Shrimp Artisanal	A	A	A	A	A	A	A	A	A
Shrimp Industrial	A	A	A	A	A	A	A	A	A
Industrial Tuna	A	A	B	A	A	A	A	A	A
Artisanal Sole and	B	B	A	B	A	B	A	A	A
TRY Oysters	A	A	A	B	A	B	A	A	A
Artisanal	C	A	A	B	B	A	A	B	A
Artisanal Axim	B	B	B	B	A	B	A	B	A
Lobster (Nephrops	A	A	C	B	B	A	A	B	B
Lesser Sunda Artisa	C	C	C	C	C	C	A	A	A
Blue Crab	B	A	A	A	A	A	A	A	B
Longline Tuna	A	A	B	B	A	B	A	A	B
Suruga Pink Shrim	C	B	B	A	B	B	A	A	B
Tokyo Bay	A	A	A	A	B		A	B	A
Artisanal Shimoni	C	A	A	A	A	B	B	A	A
Octopus	C	C	C	C	B	C	B	A	A
Artisanal Westpoir	B	B	B	B	B	B	A	A	A
Artisanal Robertsp	B	B	B	C	C	B	B	B	B
Semi-Industrial	B	B	B	C	C	B	B	B	B
Lake Chiuta	C	B	B	B	A	A	A	A	A
Skipjack Tuna	B	B	B	B	A	B	B	A	B
La Paz Bivalves	C	C	C	C	A	C	A	B	B
La Paz Bivalves	C	C	C	C	A	C	A	B	B
La Paz Bay Chocola	A	B	B	B	A	A	A	B	B
Purse Seine Tuna	A	A	B	B	A	B	A	A	A
Central Zone	B	B	B	B	A	A	A	A	A
Southern Zone	B	B	B	B	A	A	A	A	A

Fishery	QIEPollution	QIEChronicSt	QIEChronicC	QIGQuality	QIGResponsi	QINIEF	QINGDP	QIAProportic	QIATransfer
Spencer Gulf Praw									
Pabna Sadullaspra	A	A	A	A	A	A	A	B	B
Beel Chatra	B	A	A	A	A	A	A	A	B
Kailin Nadi	B	A	A	A	A	A	A	B	B
Shrimp Artisanal	A	A	A	A	A	A	A	A	
Shrimp Industrial	A	A	A	A	A	A	A	A	
Industrial Tuna	A	A	A	A	A	A	A	B	A
Artisanal Sole and	A	A	A	A	A	A	A	A	
TRY Oysters	A	A	A	A	A	A	A	A	
Artisanal	C	C	B	A	A	A	A	A	A
Artisanal Axim	B	B	A	A	A	A	A	B	B
Lobster (Nephrops	A	A	B	A	A	A	A	A	B
Lesser Sunda Artis	A	A	A	A	A	A	A	A	
Blue Crab	B	B	A	A	A	A	A	A	
Longline Tuna	B	A	A	A	A	A	A	A	
Suruga Pink Shrim	C	B	A	A	A	A	A	A	A
Tokyo Bay	A	B	B	A	A	A	A	A	A
Artisanal Shimoni	A	A	A	A	A	A	A	A	A
Octopus	A	A	A	A	A	A	A	A	A
Artisanal Westpoir	A	B	A	A	A		A	A	A
Artisanal Robertsp	B	B	B	A	A	A	A	B	
Semi-Industrial	B	B	B	A	A	A	A	B	
Lake Chiuta	B	C	B	B	A	A	A	B	
Skipjack Tuna	A	A	A	A	A	A	A	A	A
La Paz Bivalves	A	B	B	A	A	A	A	A	A
La Paz Bivalves	A	B	B	A	A	A	A	B	B
La Paz Bay Chocola	A	B	B	A	A	A	A	A	A
Purse Seine Tuna	A	A	A	A	A	A	A	B	C
Central Zone	A	A	A	A	A	A	A	A	A
Southern Zone	A	A	A	A	A	A	A	A	A

Fishery	QIASecurity	QIADurability	QIAFlexibilty	QIAExclusivit	QIHProportic	QIHTransfer	QIHSecurity	QIHDurability	QIHFlexibility
Spencer Gulf Praw					A	A			
Pabna Sadullaspra	B	A	B	B	B				A
Beel Chatra	B	A	B	A	A				A
Kailin Nadi	B	A	B	A	B				A
Shrimp Artisanal	A	A	A	A	A				
Shrimp Industrial	A	A	A	A	A				
Industrial Tuna	B	A	A	B	A				
Artisanal Sole and	B	A	B	B	A				
TRY Oysters	A	B	B	B	B	A	B	B	B
Artisanal	A	A	A	A	A	A	A	A	A
Artisanal Axim	B	B	B	B	B	C	B	A	A
Lobster (Nephrops	A	A	A	A	A	B	A	A	A
Lesser Sunda Artis	B	B	B	B	C	B	B	B	B
Blue Crab	B	B	B	B	A				
Longline Tuna	A	A	A	A	A				
Suruga Pink Shrim	A	A	A	A	A	A	A	A	A
Tokyo Bay	A	B	B	B	A				
Artisanal Shimoni	A	A	A	A	A	A	A	A	A
Octopus	A	A	A	A	A	A	B	B	B
Artisanal Westpoi	A	A	A	A	A	A	A	A	A
Artisanal Robertsp	B	B	B	B	A				
Semi-Industrial	B	B	B	B	A				
Lake Chiuta	B	A	C	B	A				C
Skipjack Tuna	A	A	A	A	A				
La Paz Bivalves	A	A	A	A		A	A	A	A
La Paz Bivalves	B	A	B	B	B	B	B	A	B
La Paz Bay Chocola	A	A	B	B	A	A	B	A	A
Purse Seine Tuna	B	B	B	B	A	C	B	C	B
Central Zone	A	A	B	A	A				
Southern Zone	A	A	B	A	A	A	A	A	A

Fishery	QIHExclusivit	QIOParticipa	QIOMgmtInfl	QIOBusInflue	QIPDays	QIPFinancial	QICLeadersh	QICCohesion	QIWBusInflu
Spencer Gulf Praw									
Pabna Sadullaspra	B	A	A	A	A	A	B	A	A
Beel Chatra	A	B	A	A	A	A	A	A	A
Kailin Nadi	A	A	A	A	A	A	A	A	A
Shrimp Artisanal		A	A	A	B	A	B	A	A
Shrimp Industrial		A	A	A	B	A	B	A	A
Industrial Tuna		B	B	B	C	A	B	A	A
Artisanal Sole and		A	B	B	A	A	B	B	B
TRY Oysters	A	A	B	B	A	A	A	A	A
Artisanal	A	A	A	B	C	B	B	B	B
Artisanal Axim	B	A	A	A	B	A	A	A	B
Lobster (Nephrops	A	A	A	A	A	A	C	B	B
Lesser Sunda Artis	B	B	B	B	B	B	B	B	B
Blue Crab		B	B	B	B	B	C	C	C
Longline Tuna		B	B	B	B	B	B	A	A
Suruga Pink Shrim	A	A	A	A	A	B	A	A	A
Tokyo Bay		A	B	B	A	B	B	A	A
Artisanal Shimoni	A	A	A	A	B	B	A	A	A
Octopus	B	A	A	A	B	B	B	A	A
Artisanal Westpoir	A	B	A	A	A	A	A	A	A
Artisanal Robertsp		A	B	B	B	B	A	A	A
Semi-Industrial		A	B	B	B	B	A	A	A
Lake Chiuta	B	B	B	B	A	C	A	A	A
Skipjack Tuna		B	B	B	C	B	A	A	A
La Paz Bivalves	A	A	A	A	A	A	A	A	B
La Paz Bivalves	B	A	A	A	A	B	A	A	B
La Paz Bay Chocola	B	B	B	B	A	B	B	A	B
Purse Seine Tuna	B					A			A
Central Zone		A	B	B	A	A	A	B	
Southern Zone	A	A	B	B	A	A	A	B	

Fishery	QIWMgmtInf	QIWHarvest	QIWPostHarv	QIMExpendit	QIMEnforcer	QIMJurisdicti	QIMSubsidie	QIDAvailabili	QIDAnalysis
Spencer Gulf Praw									
Pabna Sadullaspra	A	A	A	C	A	B	A	A	B
Beel Chatra	A	A	A	B	B	B	A	A	B
Kailin Nadi	A	A	A	B	A	B	A	A	B
Shrimp Artisanal	A	A	A	A	A	A	A	A	A
Shrimp Industrial	A	A	A	A	A	A	A	A	A
Industrial Tuna	A	A	B	B	B	A	B	B	B
Artisanal Sole and	B	B	C	B	A	A	A	B	B
TRY Oysters	A	A	A	A	B	A	A	A	A
Artisanal	B	B	C	C	B	C	B	B	B
Artisanal Axim	A	A	A		B	B	A	A	B
Lobster (Nephrops	B	B	C	B	B	B	B	A	B
Lesser Sunda Artisa	B	A	B	B	A	B	C	A	B
Blue Crab	C	C	C	B	C	C	B	A	A
Longline Tuna	B	A	A	A	A	A	B	B	A
Suruga Pink Shrimp	A	B	B	B	A	A	B	A	B
Tokyo Bay	A	A	B		A	B	B	B	B
Artisanal Shimoni	A	A	A	B	A	A	A	A	A
Octopus	A	A	B	B	A	B	A	B	A
Artisanal Westpoir	A	A	A	A	A	A	A	A	A
Artisanal Robertsp	A	B	B	C	B	B	A	A	A
Semi-Industrial	A	B	B	C	B	B	A	A	A
Lake Chiuta	A	A	A	A	A	A	A	A	C
Skipjack Tuna	A	A	A	B	A	A	B	B	B
La Paz Bivalves	A	A	A	C	A	A	A	A	A
La Paz Bivalves	A	A	A	A	B	A	A	A	A
La Paz Bay Chocola	B	A	A	B	B	A	A	B	B
Purse Seine Tuna	A	A	A	B	A	B		C	B
Central Zone				A	B	A	B	A	A
Southern Zone				A	B	A	B	A	A

Fishery	QILMPAs	QILSpatial	QILTACs	QITPricing	QITPriceAvai	QITBuyers	QITVerticalIn	QITTariffs	QITNonTariff
Spencer Gulf Praw									
Pabna Sadullaspra	A	A	A	A	B	A	A		B
Beel Chatra	A	A	A	A	B	A	A		B
Kailin Nadi	A	A	A	A	B	A	A	B	B
Shrimp Artisanal	A	A	A	A	A	A	A	A	A
Shrimp Industrial	A	A	A	A	A	A	A	A	A
Industrial Tuna	A	A	A	B	B	B	A	B	A
Artisanal Sole and	A	A	A	A	A	A	A	B	B
TRY Oysters	A	A	A	A	A	A	A	A	A
Artisanal	A	A	B	B	C	A	A	B	B
Artisanal Axim	A	A	A	A	B	B	C	B	A
Lobster (Nephrops	C	C	B	B	A	B	A	A	A
Lesser Sunda Artis	B	B	A	A	A	A	B	B	B
Blue Crab	B	B	B	B	B	A	A	A	A
Longline Tuna	A	A	A	A	A	B	A	A	B
Suruga Pink Shrim	A	A	A	A	B	A	A	B	B
Tokyo Bay	C	B	B	A	B	A	A	A	A
Artisanal Shimoni	A		A	B	B	B	A	B	C
Octopus	A	A	A	A	B	A	A		
Artisanal Westpoir	A	A	A	A	A	A	A	A	A
Artisanal Robertsp	A	A	A	B	B	B	B	B	B
Semi-Industrial	A	A	A	B	B	B	B	B	B
Lake Chiuta	A	B	C	B	B	B	B	C	C
Skipjack Tuna	A	B	A	A	B	A	A	B	B
La Paz Bivalves	A	A	A	A	A	A	A	C	C
La Paz Bivalves	A	B	A	A	A	A	A	C	C
La Paz Bay Chocola	B	B	B	B	B	A	A	C	C
Purse Seine Tuna	B	C	A					B	B
Central Zone	A	B	B	B	B	A	A	B	B
Southern Zone	A	B	B	B	B	A	A	B	B

Fishery	QIIShipping	QIIRoads	QIITechnolog	QIIExtenstior	QIIElectricity	QIIIce
Spencer Gulf Praw						
Pabna Sadullaspra	B	A	A	A	A	A
Beel Chatra	B	A	A	A	A	A
Kailin Nadi	B	A	A	A	A	A
Shrimp Artisanal	A	A	A	A	A	A
Shrimp Industrial	A	A	A	A	A	A
Industrial Tuna	A	B	B	A	A	A
Artisanal Sole and	B	A	A	A	A	A
TRY Oysters	B	A	A	A	B	B
Artisanal	B	B	B	A	B	A
Artisanal Axim	B		C	B	C	B
Lobster (Nephrops	A	A	A	A	A	A
Lesser Sunda Artis	B	A	A	A	A	B
Blue Crab	B	A	A	A	B	B
Longline Tuna	A	A	A	A	A	A
Suruga Pink Shrim	B	A	A	A	A	A
Tokyo Bay	A	A	A	B	A	A
Artisanal Shimoni	B	A	A	A	B	B
Octopus	B	A	A	B	B	A
Artisanal Westpoir	B	A	A	A	A	A
Artisanal Robertsp	B	B	B	A	A	A
Semi-Industrial	B	B	B	A	A	A
Lake Chiuta	B	B	B	A	B	B
Skipjack Tuna	B		A	B	B	A
La Paz Bivalves	B	B	A	A	A	A
La Paz Bivalves	B	B	A	A	A	A
La Paz Bay Chocola	B	A	A	B	A	A
Purse Seine Tuna	B		B	A	B	A
Central Zone	B	A	B	B	B	B
Southern Zone	B	A	B	B	B	B

Hoki	New Zealand	1.1	2011	Finfish	Single	5	4	4	4
Cod	Norway	1.1	2010	Finfish	Multi	5	5	5	5
Purse Seiners	Norway	1.1	2010	Finfish	Multi	5	5	5	5
Anchovy	Peru	1.1	2011	Finfish	Single	1	3	2	3
Blue Crab	Phillippines	1.1	2010	Crustacean	Single	1	3	2	5
Artisanal	Senegal	1.1	2010	Finfish	Multi	1	1	1	5
Artisanal Ngaparou	Senegal	1.1	2013	Finfish	Multi	3	3	2	3
Inshore Artisanal	Seychelles	1.1	2011	Finfish	Multi	3	3	4	5
Semi-Industrial	Seychelles	1.1	2011	Finfish	Multi	4	3	4	5
Sea Cucumber	Seychelles	1.1	2011	Other	Single	2	2	2	5
IO Purse Seine Tun	EU	1.2	2013	Finfish	Multi	5	5	5	5
Sherbro	Sierra Leone	1.1	2013	Finfish	Multi	3	2	1	5
Tombo	Sierra Leone	1.1	2013	Finfish	Multi	3	2	1	5
Baltic Cod	Sweden	1.1	2010	Finfish	Single	5	5	5	3
Longline Tuna	Taiwan	1.2	2013	Finfish	Multi	3	3	3	5
Lake Victoria Daga	Uganda	1.1	2010	Finfish	Single	5	5	5	5
Lake Victoria Tilapi	Uganda	1.1	2010	Finfish	Single	5	5	5	5
Nile Perch	Uganda	1.1	2010	Finfish	Multi	1	3	2	5
AK Pollock	US	1.1	2013	Finfish	Single	5	5	5	5
FL Spiny Lobster	US	1.1	2010	Crustacean	Single	5	5	5	3
Louisiana Shrimp	US	1.1	2010	Crustacean	Single	5	4	4	5
AK Salmon	US	1.1	2009	Finfish	Single	5	5	5	4
NE Groundfish	US	1.1	2008	Finfish	Multi	2	3	2	3
CA Urchin	US	1.1	2010	Other	Single	3	3	3	5
OR Dungeness Cra	US	1.1	2010	Crustacean	Single	5	5	5	4
AK Crab	US	1.1	2011	Crustacean	Single	5	5	5	3
AK Halibut	US	1.1	2011	Finfish	Single	5	5	5	5
Pacific Groundfish	US	1.1	2011	Finfish	Multi	4	4	5	4
Thanh Hoa	Vietnam	1.1	2011	Finfish	Single	1	1	1	5
Southern Zone Roc	Australia	1.1	2012	Crustacean	Single	4	4	5	5
Western Zone Aba	Australia	1.1	2011	Shellfish	Single	4	5	5	5

Hoki	4	5	4	5	3	5	5	5	5
Cod	5	4	5	4	4	4	4	5	4
Purse Seiners	5	4	5	4	4	4	5	5	5
Anchovy	4	4	4	3	4	2	4	1	3
Blue Crab	2	4	3	1	1	2	5	5	1
Artisanal	5	3	3	1	2	1	4	1	1
Artisanal Ngaparou	4	2	3	1	1	1	4	3	2
Inshore Artisanal	5	4	4	1	4	2	5	5	1
Semi-Industrial	3	3	5	1	4	5	4	5	1
Sea Cucumber	5	3	4	1	1	2	2	4	1
IO Purse Seine Tun	3	3	5	1	4	3	4	5	1
Sherbro	3	2	4	1	4	5	5	2	1
Tombo	3	2	3	1	1	3	3	5	1
Baltic Cod	4	5	4	1	3	3	3	5	1
Longline Tuna	4	1	5	1	4	2	5	3	1
Lake Victoria Daga	4	5	4	1	5	2	5		1
Lake Victoria Tilapi	4	5	4	1	2	3	5	1	1
Nile Perch	4	2	4	1	2	1	5	2	1
AK Pollock	4	5	5	5	5	5	5	4	4
FL Spiny Lobster	5	3	3	1	5	5	3	5	2
Louisiana Shrimp	1	4	4	1	4	2	4	5	2
AK Salmon	5	5	5	5	4	1	3	5	2
NE Groundfish	2	3	4	1	2	2	2	2	1
CA Urchin	5	4	4	1	3	4	4	4	2
OR Dungeness Cral	5	5	5	3	4	3	2	3	3
AK Crab	5	5	5	1	4	5	5	4	5
AK Halibut	4	5	5	5	4	4	4	4	5
Pacific Groundfish	3	5	4	1	3	2	5	2	2
Thanh Hoa	2	3	3	1	1	3	5	2	2
Southern Zone Roc	5	3	5	1	3	2	3	5	4
Western Zone Aba	5	3	5	1	3	2	5	4	4

Hoki	4	4	4	4	4	4	4	4	4
Cod	5	5	3	3	4	3	4	4	5
Purse Seiners	4	5	3	3	4	3	4	3	4
Anchovy	5	5	3	3	4	3	3	1	2
Blue Crab	2	5	5	2	4	3	5	5	5
Artisanal	4	5	2	2	4	4	4	3	4
Artisanal Ngaparou	5	4	1	2	3	2	2	2	2
Inshore Artisanal	5	5	4	3	2	2	4	3	2
Semi-Industrial	4	5	4	3	4	4	4	2	4
Sea Cucumber	5	5	4	3	2	3	2	3	3
IO Purse Seine Tun	2	3	5	5	5	3	4	3	4
Sherbro	5	5	2	2	1	2	2	3	4
Tombo	3	5	1	2	3	2	2	3	4
Baltic Cod	5	4	4	4	4	4	4	4	4
Longline Tuna	5	2	5	5	4	4	5	3	2
Lake Victoria Daga	5	5	2	2	3	3	3	4	3
Lake Victoria Tilapi	2	5	2	2	3	5	5	4	
Nile Perch	5	5	1	2	3	3	4	4	3
AK Pollock	4	4	5	4	4	4	4	4	4
FL Spiny Lobster	3	2	3	2	3	3	3	2	2
Louisiana Shrimp	1	4	4	3	3	4	4	2	3
AK Salmon	3	1	4	3	4	2	3	1	2
NE Groundfish	1	3	3	3	2	4	4	3	4
CA Urchin	4	5	4	3	4	4	5	4	4
OR Dungeness Cral	3	3	4	3	3	3	3	1	4
AK Crab	4	4	4	3	4	5	5	4	4
AK Halibut	5	5	4	4	4	4	5	3	3
Pacific Groundfish	3	5	4	4	4	4	5	3	5
Thanh Hoa	4	2	2	2	4	3	3	3	3
Southern Zone Roc	3	3	4	4	4	5	5	4	4
Western Zone Aba	3	3	4	4	4	5	5	5	5

Hoki	5	5	4	4	4	5	5	4	3
Cod	4	2	4	5	5	5	5	4	4
Purse Seiners	4	4	4	5	5	5	5	4	4
Anchovy	2	4	2	5	5	3	4	4	4
Blue Crab	5	3	4	2	1	3	2	3	5
Artisanal	4	3	4	5	5	2	3	4	4
Artisanal Ngaparou	2		3	4	4	4	3	3	5
Inshore Artisanal	4	5	4	5	5	5	5	3	5
Semi-Industrial	4	5	4	5	5	5	5	4	5
Sea Cucumber	5	5	4	5	5	5	5	3	5
IO Purse Seine Tun	4	5	5	5	5	5	5	5	5
Sherbro	3	5	3	2	5	3	3	3	4
Tombo	3	5	4	5	4	4	4	4	3
Baltic Cod	3	4	5	2	2	5	5	3	5
Longline Tuna	2	5	5	5	5	5	5	5	5
Lake Victoria Daga	4	4	5	4	4	2	2	4	5
Lake Victoria Tilapi	5	2	4	4	4	2	2	4	3
Nile Perch	4	2	4	5	5	2	2	4	5
AK Pollock	4	4	4	5	5	5	5	4	2
FL Spiny Lobster	3	5	5	4	5	5	5	3	4
Louisiana Shrimp	3	2	5	3	3	5	4	3	5
AK Salmon		2	4	2	3	5	5	3	3
NE Groundfish	5	4	2	2	3	5	5	3	3
CA Urchin	5	5	4	2	2	5	4	3	4
OR Dungeness Cra	3	5	3	3	2	5	5	4	4
AK Crab	5	5	4	4	4	5	5	3	3
AK Halibut	4	5	5	4	4	5	5	3	3
Pacific Groundfish	4	2	2	3	4	5	5	3	3
Thanh Hoa	3	3	5	2	3	3	3	3	5
Southern Zone Roc	4	5	5	5	5	5	5	5	5
Western Zone Aba	5	5	5	5	5	5	5	5	5

Hoki	3	3	5	5	3	3	4	2	4
Cod	5	5	5	5	2	4	4	4	4
Purse Seiners	5	5	5	5	2	4	4	4	4
Anchovy	5	5	3	4	3	4	4	4	5
Blue Crab	2	2	2	2	2	5	4	5	4
Artisanal	3	3	2	3	2	3	5	5	4
Artisanal Ngaparou	4	4	2	3	3	4	5	2	3
Inshore Artisanal	4	4	5	5	2	5	4	3	5
Semi-Industrial	5	5	5	5	2	4	4	3	5
Sea Cucumber	5	5	5	5	2	4	3	3	5
IO Purse Seine Tun	5	5	4	3	5	2	5	5	5
Sherbro	2	5	3	3	2	4	5	4	5
Tombo	3	3	3	4	3	3	5	5	5
Baltic Cod	3	3	5	5	2	4	4	2	4
Longline Tuna	4	4	3	3	3	2	4	3	4
Lake Victoria Daga	3	3	2	1	2	5	3	2	5
Lake Victoria Tilapi	3	3	2	2	2	3	4	3	5
Nile Perch	4	4	2	1	2	5	3	1	5
AK Pollock	4	5	5	5	3	2	5	4	5
FL Spiny Lobster	3	3	5	5	2	4	4	3	4
Louisiana Shrimp	4	4	5	4	3	4	5	2	3
AK Salmon	2	3	5	5	3	3	2	5	2
NE Groundfish	3	3	5	5	2	4	3	3	5
CA Urchin	1	1	4	4	2	4	5	2	4
OR Dungeness Cral	3	3	5	5	3	3	4	4	3
AK Crab	4	4	5	5	3	3	4	3	4
AK Halibut	3	4	4	5	2	3	4	4	4
Pacific Groundfish	3	4	5	5	3	3	4	5	5
Thanh Hoa	2	2	2	3	2	5	4	2	5
Southern Zone Roc	4	4	5	5	4	5	4	5	4
Western Zone Aba	4	4	5	5	4	5	4	5	4

Hoki	4	5	5	4	5	4	4	4	4
Cod	3	5	5	3	5	4	4	4	3
Purse Seiners	3	5	5	3	5	4	4	4	3
Anchovy	1	5	3	5	5	5	5	3	3
Blue Crab	3	3	5	2	4	4	2	4	3
Artisanal	5	4	4	4	5	5	5	3	2
Artisanal Ngaparou	4	4	1	2	4	2	1	5	4
Inshore Artisanal	3	2	2	3	2	5	4	5	4
Semi-Industrial	4	4	5	2	5	4	5	3	4
Sea Cucumber	5	5	5	3	4	3	5	4	4
IO Purse Seine Tun	3	5	5	4	5	2	4	5	5
Sherbro	2	2	1	1	1	1	2	1	5
Tombo	3	3	1	1	1	1	2	2	5
Baltic Cod	4	3	5	3	5	4	3	5	5
Longline Tuna	5	5	5	4	5	5	5	5	5
Lake Victoria Daga	2	3	1	3	1	1	4	2	5
Lake Victoria Tilapi	3	2	1	3	1	1	4	4	5
Nile Perch	4	4	5	3	5	2	4	2	2
AK Pollock	4	4	5	3	5	5	4	5	4
FL Spiny Lobster	4	4	5	3	5	3	5	5	5
Louisiana Shrimp	3	2	5	3	4	5	5	5	5
AK Salmon	4	4	5	2	5	4	2	3	2
NE Groundfish	4	3	5	4	5	4	4	4	5
CA Urchin	5	4	5	4	5	5	5	4	5
OR Dungeness Cra	4	2	4	3	5	4	4	4	3
AK Crab	5	5	5	3	5	5	5	5	5
AK Halibut	5	2	5	5	5	2	4	4	3
Pacific Groundfish	5	3	5	3	5	4	1	3	5
Thanh Hoa	3	3	2	3	3	4	2	2	4
Southern Zone Roc	5	5	5	5	4	2	5	4	4
Western Zone Aba	5	5	5	5	4	2	5	4	4

Hoki	4	5	5	4	4	4	5	4	5
Cod	3	5	5	3	3	4	2	4	5
Purse Seiners	3	5	5	3	3	4	2	4	5
Anchovy	4	2	4	4	4	4	5	5	5
Blue Crab	4	2	1	4	2	4	4	3	5
Artisanal	4	4	4	2	3	3	5	5	5
Artisanal Ngaparou	4	3	2	1	3	4	3	4	4
Inshore Artisanal	4	4	3	5	3	4	5	5	5
Semi-Industrial	4	4	4	5	3	4	5	5	5
Sea Cucumber	5	4	4	4	3	4	5	5	5
IO Purse Seine Tun	5	4	4	2	4	3	5	5	5
Sherbro	2	1	2	2	2	3	2	5	3
Tombo	2	1	3	1	2	4	4	4	3
Baltic Cod	5	5	4	4	4	3	4	4	5
Longline Tuna	5	5	5	5	5	3	5	5	5
Lake Victoria Daga	1	2	5	1	2	4	4	4	2
Lake Victoria Tilapi	4	5	5	2	2	4	4	4	2
Nile Perch	2	3	4	3	5	4	5	5	5
AK Pollock	5	5	4	5	5	3	5	5	5
FL Spiny Lobster	5	5	5	3	3	2	3	4	5
Louisiana Shrimp	2	5	5	3	4	3	3	4	5
AK Salmon	4	5	4	2	3	3	4	4	5
NE Groundfish	3	5	3	3	4	3	4	4	5
CA Urchin	5	5	5	4	3	3	3	4	5
OR Dungeness Cra	2	5	3	4	3	4	2	2	5
AK Crab	5	5	4	5	4	3	5	3	5
AK Halibut	4	5	4	3	4	4	4	4	5
Pacific Groundfish	4	5	5	4	4	4	2	2	5
Thanh Hoa	2	2	4	3	3	4	4	4	3
Southern Zone Roc	5	5	5	4	4	4	4	5	5
Western Zone Aba	2	5	5	4	4	4	4	5	5

Hoki	5	4	4	3	3	5	5	3	4
Cod	5	4	3	4	4	5	5	2	3
Purse Seiners	5	4	3	4	4	5	5	2	3
Anchovy	5	4	4	4	4	3	4	3	5
Blue Crab	5	5	3	2	2	2	2	3	4
Artisanal	4	4	3	4	5	3	3	2	5
Artisanal Ngaparou	3	3	1	3	4	4	3	3	5
Inshore Artisanal	5	4	1	3	3	5	5	3	5
Semi-Industrial	5	4	1	2	2	5	5	2	5
Sea Cucumber	5	4	4	4	4	5	5	2	5
IO Purse Seine Tun	5	5	5	3	2	4	3	2	3
Sherbro	3	3	5	2	4	3	3	2	5
Tombo	4	3	5	4	4	3	4	2	5
Baltic Cod	5	4	5	4	4	5	5	2	5
Longline Tuna	5	5	5	4	3	5	5	4	5
Lake Victoria Daga	2	2	5	3	2	2	2	2	5
Lake Victoria Tilapi	2	2	5	4	3	2	2	2	5
Nile Perch	3	5	2	3	3	5	3	3	5
AK Pollock	5	5	1	2	4	4	4	2	1
FL Spiny Lobster	5	4	5	3	4	5	5	2	4
Louisiana Shrimp	5	4	5	2	3	5	4	2	3
AK Salmon	5	4	3	3	3	5	5	2	2
NE Groundfish	5	4	4	3	2	5	5	2	3
CA Urchin	5	3	5	1	1	4	4	2	3
OR Dungeness Cral	5	3	3	3	3	5	5	3	2
AK Crab	5	4	3	4	3	5	5	2	3
AK Halibut	5	4	2	2	2	4	5	2	3
Pacific Groundfish	5	4	2	2	2	2	5	5	3
Thanh Hoa	3	3	5	2	2	2	2	2	5
Southern Zone Roc	5	4	5	4	4	3	5	5	5
Western Zone Aba	5	4	5	4	4	3	5	5	5

Hoki	4	4	5	5	5	5	5	5	4
Cod	2	5	5	5	5	5	5	5	5
Purse Seiners	2	5	5	5	5	5	5	5	5
Anchovy	4	4	5	4	5	5	5	3	3
Blue Crab	3	4	5	4	5	5	5	3	2
Artisanal	4	1	5	5	5	4	4	2	2
Artisanal Ngaparou	5	1	5	4	5	5	5	1	3
Inshore Artisanal	4	4	5	5	5	4	5	3	4
Semi-Industrial	4	4	5	5	2	5	5	3	4
Sea Cucumber	3	4	5	5	5	2	5	3	4
IO Purse Seine Tun	3	4	5	5	4	5	5	5	4
Sherbro	5	1	5	5	5	5	5	1	2
Tombo	5	1	5	5	2	2	4	1	2
Baltic Cod	4	5	5	5	3	2	4	5	5
Longline Tuna	5	4	5	5	5	5	5	5	4
Lake Victoria Daga	2	2	5	5	4	4	5	2	2
Lake Victoria Tilapi	3	2	5	5	4	4	5	2	2
Nile Perch	4	2	5	5	4	4	5	2	2
AK Pollock	4	5	5	5	5	5	5	5	5
FL Spiny Lobster	4	4	3	3	4	4	5	4	4
Louisiana Shrimp	2	4	5	5	3	4	4	5	4
AK Salmon	1	4	4	5	5	5	5	5	5
NE Groundfish	4	4	4	5	4	5	5	5	5
CA Urchin	4	3	5	4	5	4	5	5	4
OR Dungeness Cra	3	4	4	5	5	5	5	5	4
AK Crab	3	2	5	4	5	5	5	5	4
AK Halibut	4	4	4	5	5	5	5	5	5
Pacific Groundfish	3	4	5	5	5	5	5	5	4
Thanh Hoa	4	2	5	3	4	4	5	2	2
Southern Zone Roc	4	5	4	4	5	5	5	5	5
Western Zone Aba	4	5	4	4	5	5	5	5	5

Hoki	5	5	5	5	4	5	5	5	5
Cod	4	5	4	3	4	5	4	5	4
Purse Seiners	4	5	4	3	4	5	4	5	4
Anchovy	4	3	5	4	4	4	3	3	4
Blue Crab	3	2	1	1	1	1	5	1	1
Artisanal	2	1	4	1	2	4	4	2	1
Artisanal Ngaparou	2	1	4	1	3	3	2	1	1
Inshore Artisanal	2	4	1	1	2	1	4	1	1
Semi-Industrial	2	4	4	1	4	1	4	1	1
Sea Cucumber	2	4	5	1	4	2	2	4	1
IO Purse Seine Tun	5	5	3	1	5	4	4	4	1
Sherbro	1	1	4	1	3	3	2	2	1
Tombo	1	1	4	1	3	3	2	1	1
Baltic Cod	5		5	2	4	2	2	4	2
Longline Tuna	5	4	1		5	5	5	1	2
Lake Victoria Daga	3	1	4	1	2	2	3	2	1
Lake Victoria Tilapi	3	1	4	1	2	2	3	2	1
Nile Perch	3	1	4	1	2	2	3	2	1
AK Pollock	5	5	5	2	5	5	4	4	5
FL Spiny Lobster	5	5	4	4	4	5	3	3	1
Louisiana Shrimp	5	5	4	1	1	3	2	1	1
AK Salmon	5	5	5	3	5	5	1	4	1
NE Groundfish	5	5	5	4	5	5	2	4	4
CA Urchin	5	5	5	2	5	5	4	5	1
OR Dungeness Cral	5	5	5	3	4	4	2	4	1
AK Crab	5	5	5	5	5	5	3	5	5
AK Halibut	5	5	5	5	5	5	3	3	5
Pacific Groundfish	5	5	5	3	4	5	4	4	3
Thanh Hoa	2	1	4	3	1	4	4	1	1
Southern Zone Roc	5	5	5	5	5	5	4	4	5
Western Zone Aba	5	5	5	5	5	5	4	4	5

Hoki	5	5	5	5	5	5	4	2	5
Cod	4	4	5	4	4	4	3	2	4
Purse Seiners	4	4	5	4	4	4	3	3	4
Anchovy	3	4	4	3	3	4	4	2	1
Blue Crab						1	2	1	2
Artisanal						3	3	1	2
Artisanal Ngaparou						4		2	4
Inshore Artisanal						2	3	2	4
Semi-Industrial						5	3	3	2
Sea Cucumber						2	4	4	2
IO Purse Seine Tun						3	5	5	3
Sherbro						3	2	2	4
Tombo						4	3	2	5
Baltic Cod	2	4	2	2	4	5	4	1	2
Longline Tuna	3	5	3	5	1	4	3	3	3
Lake Victoria Daga						5	2	1	4
Lake Victoria Tilapi						5	2	1	4
Nile Perch						5	2	1	4
AK Pollock	3	5	5	4	4	5	5	4	5
FL Spiny Lobster						1	3	2	2
Louisiana Shrimp						1	3	2	4
AK Salmon						4	2	2	2
NE Groundfish	3	3	4	2	2	5	3	1	5
CA Urchin						5	4	3	3
OR Dungeness Cral						4	3	3	3
AK Crab	4	5	5	3	5	5	4	3	3
AK Halibut	5	5	5	3	3	3	3	2	3
Pacific Groundfish	3	4	5	4	4	4	3	2	5
Thanh Hoa						3	1	2	1
Southern Zone Roc	5	5	5	4	4	4	4	3	5
Western Zone Aba	5	5	5	4	4	4	4	3	5

Hoki	5	5	4	2	2	2	3	4	4
Cod	3	2	4	1	1	1	3	2	5
Purse Seiners	3	2	4	1	1	1	3	2	5
Anchovy	1	3	3	1	3	1	4	5	3
Blue Crab	1	1	3	3	3	1	5	5	2
Artisanal	2	3	4	3	2	1	5	5	2
Artisanal Ngaparou	3	2	4	3	1	1	4	3	3
Inshore Artisanal	1	5	4	1	1	1	3	4	2
Semi-Industrial	1	5	4	1	1	1	3	4	4
Sea Cucumber	2	5	4	1	1	1	2	5	4
IO Purse Seine Tun	1	5	5	1	1	1	4	5	3
Sherbro	1	2	3	3	3	1	5	5	2
Tombo	3	5	4	3	3	1	5	5	3
Baltic Cod	1	2	3	1	1	1	1	1	4
Longline Tuna	1	5	5	1	1	1	1	5	2
Lake Victoria Daga	2	2	2	2	2	1	5	5	2
Lake Victoria Tilapi	2	2	2	1	1	1	4	5	2
Nile Perch	2	2	2	2	2	1	3	4	2
AK Pollock	4	5	3	1	2	1	1	4	5
FL Spiny Lobster	3	3	3	1	1	1	1	5	4
Louisiana Shrimp	3	3	4	4	3	2	4	5	4
AK Salmon	1	3	3	3	2	1	3	3	5
NE Groundfish	2	4	2	2	2	1	2	4	2
CA Urchin	3	4	5	3	2	1	4	4	5
OR Dungeness Cral	3	3	3	2	3	1	2	4	5
AK Crab	3	5	3	1	3	1	2	3	3
AK Halibut	4	4	3	2	2	1	2	4	5
Pacific Groundfish	2	3	4	3	3	1	1	4	5
Thanh Hoa	1	4	3	2	3	1	1	5	2
Southern Zone Roc	5	5	5	1	2	1	1	4	4
Western Zone Aba	5	5	5	1	2	1	1	4	4

Hoki	5	5	4	4	4	5	5	3	4
Cod	4	4	5	4	2	2	3	4	5
Purse Seiners	4	4	5	4	2	2	3	4	5
Anchovy	3	5	5	4	2	2	4	1	2
Blue Crab	3	5	4	2	3	1	1	1	1
Artisanal	1	4	3	3	2	2	1	2	2
Artisanal Ngaparou	4	1	2	5	5	4	1	4	3
Inshore Artisanal	2	3	4	4	3	1	1	1	3
Semi-Industrial	5	3	4	4	1	1	1	1	3
Sea Cucumber	5	3	4	4	4	1	1	1	4
IO Purse Seine Tun	4	2	4	5	1	1	1	1	4
Sherbro	1	5	2	1	2	2	1	4	3
Tombo	3	5	3	2	1	1	1	5	3
Baltic Cod	4	2	5	5	3	2	4	2	4
Longline Tuna	4	3	2	2	1	1	1	4	2
Lake Victoria Daga	4	5	2	3	1	1	1	2	3
Lake Victoria Tilapi	4	5	2	3	1	1	1	2	3
Nile Perch	1	5	4	3	1	1	1	4	3
AK Pollock	5	5	5	5	3	1	5	1	4
FL Spiny Lobster	1	5	5	5	5	1	5	1	2
Louisiana Shrimp	4	5	5	4	2	1	1	5	5
AK Salmon	5	5	5	5	4	2	5	3	5
NE Groundfish	4	4	5	5	3	1	2	4	5
CA Urchin	5	5	4	3	3	1	1	2	3
OR Dungeness Cral	5	3	4	3	5	5	4	2	3
AK Crab	4	5	5	5	4	5	4	5	4
AK Halibut	5	4	5	4	2	2	5	1	4
Pacific Groundfish	5	5	5	5	4	1	5	1	3
Thanh Hoa	1	5	2	2	1	1	1	2	2
Southern Zone Roc	5	5	5	4	3	3	5	2	4
Western Zone Aba	5	5	5	4	3	3	5	2	4

Hoki	2	5	5	4	4	4	5	4	5
Cod	5	3	4	5	5	4	5	5	5
Purse Seiners	5	3	4	5	5	4	5	5	5
Anchovy	5	3	5	5	4	4	4	3	3
Blue Crab	5	1	1	5	3	2	2	2	2
Artisanal	2	3	1	4	3	2	3	2	3
Artisanal Ngaparou	5	4	5	3	4	2	5	5	2
Inshore Artisanal	5	4	5	5	4	3	2	2	4
Semi-Industrial	3	2	5	5	4	3	4	2	4
Sea Cucumber	4	3	5	5	4	3	4	2	4
IO Purse Seine Tun	3	3	5	5	4		5	5	4
Sherbro	5	5	5	5	1	1	2	3	3
Tombo	5	5	5	5	1	3	3	2	3
Baltic Cod	3	1	3	4	4	4	5	3	5
Longline Tuna	5	1	4	5	5	5	5	5	5
Lake Victoria Daga	5	3	3	5	3	2	2	2	3
Lake Victoria Tilapi	5	2	3	5	3	2	2	2	3
Nile Perch	5	2	5	4	3	2	2	3	3
AK Pollock	3	4	4	4	4	2	5	5	4
FL Spiny Lobster	3	2	5	4	4	4	5	5	5
Louisiana Shrimp	5	1	2	4	4	4	5	5	4
AK Salmon	2	2	5	5	4	2	5	3	4
NE Groundfish	4	2	5	5	5	5	5	4	5
CA Urchin	3	1	2	4	4	4	5	4	5
OR Dungeness Cra	3	2	5	4	4	4	5	4	5
AK Crab	2	2	5	5	5	5	5	5	5
AK Halibut	5	2	5	5	3	3	5	5	4
Pacific Groundfish	3	3	5	5	4	5	5	4	5
Thanh Hoa	2	2	5	4	4	2	2	3	4
Southern Zone Roc	3	4	4	4	5	4	5	4	5
Western Zone Aba	3	4	4	4	5	4	5	4	5

Hoki	5	A	A	B	B	B	B	A	B
Cod	5	A	A	A	A	A	A	C	A
Purse Seiners	5	A	A	A	A	A	A	C	A
Anchovy	3	A	B	B	B	C	C	B	B
Blue Crab	3	A	A	B	B	A	A	A	A
Artisanal	3	A	A	B	A	A	A	A	B
Artisanal Ngaparou	4	B	B	B	B	A	B	B	A
Inshore Artisanal	4	A	A	B	B	A	A	A	A
Semi-Industrial	5	A	B	B	B	A	A	A	A
Sea Cucumber	4	A	B	B	B	A	A	A	A
IO Purse Seine Tun	5	A	A	A	A	A	B	B	A
Sherbro	3	b	B	C	C	A	B	B	a
Tombo	4	b	B	C	C	A	B	B	b
Baltic Cod	5	A	A	A	A	A	A	A	A
Longline Tuna	5	A	B	B	B	A	A	B	A
Lake Victoria Daga	1	A	A	A	A	A	A	A	A
Lake Victoria Tilapi	3	A	A	A	A	A	A	A	A
Nile Perch	3	A	A	B	B	B	A	C	A
AK Pollock	5	A	A	A	A	A	A	B	A
FL Spiny Lobster	5	A	A	B	B	B	A	C	B
Louisiana Shrimp	4	A	A	B	B	A	A	B	C
AK Salmon	3	A	B	A	A	B	A	B	A
NE Groundfish	5	A	A	A	A	B	A	B	A
CA Urchin	5	A	C	C	C	A	A	B	B
OR Dungeness Cral	5	A	A	A	A	B	A	A	A
AK Crab	5	A	A	A	A	C	B	A	A
AK Halibut	5	A	A	A	A	A	B	B	A
Pacific Groundfish	5	A	A	B	B	A	B	A	B
Thanh Hoa	4	A	B	B	B	B	A	C	C
Southern Zone Roc	5	A	A	A	A	A	A	A	A
Western Zone Aba	5	A	A	A	A	A	A	A	A

Hoki	A	B	A	B	A	B	B	B	A
Cod	B	B	B	A	B	A	C	B	A
Purse Seiners	B	B	B	A	B	A	C	B	A
Anchovy	A	B	A	A	A	A	A	C	C
Blue Crab	A	B	A	A	B	B	A	A	A
Artisanal	C	A	A	A	A	B	C	B	B
Artisanal Ngaparou	A	C	A	A	C	C	C	B	A
Inshore Artisanal	A	B	A	B	A	C	A	A	A
Semi-Industrial	A	B	C	A	A	C	A	A	A
Sea Cucumber	A	B	A	B	A	C	A	A	A
IO Purse Seine Tun	A	B	A	A	A	B	B	B	A
Sherbro	c	c	a	c	c	c	c	b	a
Tombo	b	c	a	a	b	c	b	a	a
Baltic Cod	B	B	B	A	C	B	A	A	A
Longline Tuna	B	B	A	C	B	B	C	A	A
Lake Victoria Daga	A	C	A		A	A	A	B	B
Lake Victoria Tilapi	A	C	A	A	A	A	A	B	B
Nile Perch	A	A	A	C	A	A	A	A	B
AK Pollock	A	B	A	B	A	A	A	B	B
FL Spiny Lobster	A	B	B	A	B	A	A	A	A
Louisiana Shrimp	B	C	B	C	C	B	C	C	B
AK Salmon	B	B	B	B	B	A	A	B	B
NE Groundfish	A	B	A	A	A	A	B	B	A
CA Urchin	B	B	A	B	B	A	B	B	B
OR Dungeness Cra	A	A	A	B	B	A	B	A	A
AK Crab	A	A	A	A	B	A	C	C	C
AK Halibut	B	B	A	B	B	A	B	B	A
Pacific Groundfish	A	B	A	A	C	A	C	B	B
Thanh Hoa	C	B	B	C	C	C	C	C	B
Southern Zone Roc	B	A	A	A	B	A	A	B	B
Western Zone Aba	B	A	A	A	B	A	A	B	B

Hoki	A	B	A	B	C	C	C	B	B
Cod	B	A	A	A	A	A	A	B	B
Purse Seiners	B	A	A	A	A	A	A	B	B
Anchovy	C	A	A	A	A	A	B	B	B
Blue Crab	A	B	B	B	B	B	B	A	B
Artisanal	B	B	B	A	B	C	C	B	A
Artisanal Ngaparou	A	C	C	C	C	C		B	C
Inshore Artisanal	A	C	A	C	A	A	B	A	A
Semi-Industrial	A	C	A	C	A	A	B	A	A
Sea Cucumber	A	C	A	C	A	A	B	A	A
IO Purse Seine Tun	A	A	A	B	B	B	B	A	A
Sherbro	a	c	c	c	c	c	c	b	c
Tombo	a	c	c	c	c	c	c	b	c
Baltic Cod	A	A	A	A	A	B	A	A	B
Longline Tuna	B	C	C	C	A	A	C	A	A
Lake Victoria Daga	A	B	B	C	B	C	C	A	B
Lake Victoria Tilapi	A	A	B	C	C	B	C	A	B
Nile Perch	A	B	A	B	B	B	B	B	B
AK Pollock	A	A	A	B	A	B	B	A	A
FL Spiny Lobster	B	A	A	A	A	B	A	A	A
Louisiana Shrimp	B	C	C	C	C	C	C	A	C
AK Salmon	A	A	A	A	A		B	B	B
NE Groundfish	A	A	A	A	A	A	A	A	B
CA Urchin	B	A	A	A	A	A	B	B	B
OR Dungeness Cra	A	A	A	A	A	A	A	A	A
AK Crab	B	A	A	A	A	A	A	A	C
AK Halibut	A	A	A	B	A	B	B	A	B
Pacific Groundfish	B	A	A	A	A	A	A	A	C
Thanh Hoa	A	C	C	C	C	C	C	C	B
Southern Zone Roc	A	A	A	A	A	A	A	A	B
Western Zone Aba	A	A	A	A	A	A	A	A	B

Hoki	B	A	A	B	C	C	C	A	A
Cod	B	A	A	B	C	B	B	B	B
Purse Seiners	B	A	A	B	C	B	B	B	B
Anchovy	B	B	B	A	B	B	B	A	B
Blue Crab	B	B	A	B	A	B	A	B	A
Artisanal	A	A	B	B	A	B	B	A	B
Artisanal Ngaparou	C	B	A	B	A	C	C	B	A
Inshore Artisanal	A	A	A	A	A	A	A	A	A
Semi-Industrial	A	A	A	A	A	A	A	A	A
Sea Cucumber	A	A	A	A	A	A	A	A	A
IO Purse Seine Tun	B	A	A	B	A	A	A	C	C
Sherbro	b	b	a	b	a	c	b	b	a
Tombo	b	b	a	a	b	c	c	b	a
Baltic Cod	B	A	A	B	A	B	B	A	A
Longline Tuna	A	A	A	A	A	B	B	B	B
Lake Victoria Daga	A	A	A	A	A	B	B	A	A
Lake Victoria Tilapi	A	A	A	A	A	B	B	A	A
Nile Perch	A	A	A	B	A	B	B	A	A
AK Pollock	A	A	A	A	B	B	B	A	A
FL Spiny Lobster	B	A	A	B	A	B	B	A	A
Louisiana Shrimp	B	A	A	A	A	C	B	A	A
AK Salmon	B	A	B	B	A	A	A	A	B
NE Groundfish	B	A	A	A	B	B	B	A	A
CA Urchin	B	A	B	C	B	C	C	B	B
OR Dungeness Cral	A	A	A	A	A	A	A	A	A
AK Crab	C	A	A	A	C	C	B	A	A
AK Halibut	B	A	A	B	B	B	B	B	A
Pacific Groundfish	C	A	A	C	A	C	C	A	A
Thanh Hoa	B	B	B	B	A	B	B	A	B
Southern Zone Roc	A	A	A	A	A	B	A	A	A
Western Zone Aba	A	A	A	A	A	B	A	A	A

Hoki	B	C	C	C	C	B	B	C	C
Cod	B	B	B	B	B	A	A	A	A
Purse Seiners	B	B	B	B	B	A	A	A	A
Anchovy	A	A	B	C	A	A	A	A	A
Blue Crab	B	A	B	A	B	B	B	A	C
Artisanal	B	B	B	A	C	B	A	B	C
Artisanal Ngaparou	A	A	A	B	C	A	C	A	B
Inshore Artisanal	A	A	A	A	A	A	A	A	B
Semi-Industrial	A	A	A	A	A	A	A	A	B
Sea Cucumber	A	A	A	A	A	A	A	A	C
IO Purse Seine Tun	A	B	A	B	B	A	A	A	B
Sherbro	a	a	a	b	b	a	c	a	c
Tombo	a	b	a	b	b	a	b	a	c
Baltic Cod	B	A	A	B	B	A	B	A	B
Longline Tuna	B	A	C	B	B	A	A	A	B
Lake Victoria Daga	A	A	B	C	A	A	A	A	C
Lake Victoria Tilapi	A	A	B	B	A	A	B	A	B
Nile Perch	A	A	B	A	A	A	A	A	C
AK Pollock	B	A	A	A	A	B	B	A	A
FL Spiny Lobster	B	B	B	B	A	A	B	A	B
Louisiana Shrimp	A	A	A	A	B	A	A	A	B
AK Salmon	B	B	B	A	A	A	A	A	A
NE Groundfish	B	B	B	B	A	A	A	A	A
CA Urchin	C	B	A	A	B	A	B	A	B
OR Dungeness Cra	A	A	A	B	A	A	B	B	B
AK Crab	A	C	C	C	A	A	A	A	A
AK Halibut	A	B	B	B	A	A	C	A	A
Pacific Groundfish	C	A	A	A	A	A	B	B	B
Thanh Hoa	B	B	B	B	B	B	C	C	C
Southern Zone Roc	B	B	A	A	A	A	A	A	B
Western Zone Aba	B	B	A	A	A	A	A	A	B

Hoki	B	C	C	C	C	C	A	B	C
Cod	A	A	B	D	C	B	A	A	B
Purse Seiners	A	A	B	C	C	B	A	A	B
Anchovy	B	A	A	C	A	A	B	B	C
Blue Crab	C	B	B	C	C	B	A	B	B
Artisanal	B	B	A	C	A	A	A	B	B
Artisanal Ngaparou	A	B	C	C	A	B	A	A	A
Inshore Artisanal	A	B	B	A	B	B	A	B	B
Semi-Industrial	A	B	A	A	A	B	A	A	B
Sea Cucumber	A	B	A	A	A	B	B	A	B
IO Purse Seine Tun	A	B	A	A	A	A	B	A	C
Sherbro	a	b	b	c	a	b	a	a	c
Tombo	a	b	b	b	a	b	a	a	a
Baltic Cod	A	A	B	A	A	A	A	A	A
Longline Tuna	A	B	A	A	B	A	B	A	B
Lake Victoria Daga	A	C	B	C	B	A	B	A	C
Lake Victoria Tilapi	A	B	B	C	B	B	A	A	C
Nile Perch	A	C	A	C	A	B	B	B	C
AK Pollock	A	A	A	A	A	A	A	A	B
FL Spiny Lobster	A	B	B	B	A	B	A	A	B
Louisiana Shrimp	A	A	A	B	A	A	A	A	C
AK Salmon	A	A	B	B	B	B	B	B	B
NE Groundfish	A	B	A	C	B	A	A	A	B
CA Urchin	A	B	A	B	B	A	A	A	C
OR Dungeness Cral	A	A	A	B	A	A	A	A	A
AK Crab	A	A	A	A	A	A	A	C	C
AK Halibut	A	B	A	B	B	A	A	A	B
Pacific Groundfish	C	C	C	C	A	C	C	C	B
Thanh Hoa	C	C	C	A	B	B	B	C	C
Southern Zone Roc	A	A	B	A	A	A	A	A	B
Western Zone Aba	A	A	B	A	A	A	A	A	B

Hoki	B	B	B	B	A	A	B	B	C
Cod	B	C	B	B	A	A	B	B	A
Purse Seiners	B	C	B	B	A	A	B	B	A
Anchovy	C	B	B	B	A	A	B	A	B
Blue Crab	B	B	B	B	A	A	B	B	B
Artisanal	B	A	A	A	B	B	C	A	B
Artisanal Ngaparou	A	B	B	C	B	A	B	A	C
Inshore Artisanal	A	B	A	A	A	A	A	A	A
Semi-Industrial	A	A	A	A	A	A	A	A	A
Sea Cucumber	A	A	A	A	A	A	A	A	B
IO Purse Seine Tun	A	B	A	A	A	A	A	A	B
Sherbro	a	c	c	c	b	a	b	a	c
Tombo	a	b	c	c	b	a	c	b	c
Baltic Cod	A	B	B	A	A	A	A	A	B
Longline Tuna	B	B	A	A	A	A	A	A	B
Lake Victoria Daga	A	A	B	A	B	B	B	A	B
Lake Victoria Tilapi	A	A	B	A	B	B	B	A	B
Nile Perch	B	A	B	B	B	B	A	A	A
AK Pollock	B	B	A	A	A	A	A	A	B
FL Spiny Lobster	B	B	B	B	A	A	B	B	B
Louisiana Shrimp	B	C	B	B	A	A	A	A	B
AK Salmon	B	A	B	B	A	B	B	A	B
NE Groundfish	A	A	B	B	A	A	A	A	B
CA Urchin	C	C	C	B	A	B	C	C	B
OR Dungeness Cra	A	A	A	A	A	A	A	A	A
AK Crab	C	C	C	C	C	C	C	C	C
AK Halibut	A	B	B	B	B	A	B	B	B
Pacific Groundfish	B	C	C	C	A	A	C	A	C
Thanh Hoa	C	C	B	A	C	B	C	B	B
Southern Zone Roc	B	B	A	A	A	A	A	B	B
Western Zone Aba	B	B	A	A	A	A	A	B	B

Hoki	C	B	B	A	B	C	A	A	A
Cod	A	C	A	A	C	C	A	A	A
Purse Seiners	A	C	A	A	C	C	A	A	A
Anchovy	B	B	B	A	A	B	A	A	B
Blue Crab	B	A	A	A	B	B	B	A	A
Artisanal	C	A	C	C	A	B	A	A	A
Artisanal Ngaparou	C	B	B	A	A	A	B	B	A
Inshore Artisanal	A	A	A	A	A	A	B	A	A
Semi-Industrial	A	A	A	A	A	A	B	A	A
Sea Cucumber	A	A	A	A	A	B	B	A	A
IO Purse Seine Tun	B	B	B	B	A	B	A	A	A
Sherbro	c	b	b	a	a	a	b	b	a
Tombo	c	b	b	a	b	a	b	b	a
Baltic Cod	B	B	A	A	A	A	A	A	A
Longline Tuna	B	A	A	A	A	B	A	A	A
Lake Victoria Daga	A	A	B	A	B	B	A	A	A
Lake Victoria Tilapi	B	A	B	A	A	B	A	A	A
Nile Perch	A	A	B	B	A	A	A	A	A
AK Pollock	B	B	B	B	A	B	A	B	A
FL Spiny Lobster	B	B	A	A	B	B	A	B	B
Louisiana Shrimp	B	A	A	A	A	A	A	A	A
AK Salmon	B	A	A	B	A	A	A	A	A
NE Groundfish	B	B	A	A	B	B	A	B	A
CA Urchin	B	B	B	C	C	C	B	B	B
OR Dungeness Cral	A	A	A	A	A	B	A	A	A
AK Crab	C	C	C	C	C	C	A	B	B
AK Halibut	B	B	A	A	B	B	B	B	A
Pacific Groundfish	C	C	A	A	C	C	A	B	A
Thanh Hoa	C	A	B	B	B	B	A	A	C
Southern Zone Roc	B	A	A	A	B	B	A	A	A
Western Zone Aba	B	A	A	A	B	B	A		

Hoki	A	A	A	A	A	A	A	B	A
Cod	A	A	A	A	A	A	A	A	A
Purse Seiners	A	A	A	A	A	A	A	A	A
Anchovy	A	A	A	A	A	A	A	A	A
Blue Crab	A	A	A	B	B	B	B	A	A
Artisanal	A	A	A	A	A	A	A	B	A
Artisanal Ngaparou	B	B	A	B	B	B	B	B	B
Inshore Artisanal	A	A	A	A	A	A	A	A	A
Semi-Industrial	A	A	A	A	A	A	A	A	A
Sea Cucumber	A	A	A	A	A	A	A	A	A
IO Purse Seine Tun	B	A	A	A	A	A	A	B	B
Sherbro	b	b	a	b	b	b	b	b	b
Tombo	b	a	a	b	b	b	b	b	b
Baltic Cod	B	B	A	A	A	A	A	A	B
Longline Tuna	A	A	A	A	A	A	A	A	
Lake Victoria Daga	A	A	A	A	A	A	A	A	
Lake Victoria Tilapi	A	A	A	A	A	A	A	A	
Nile Perch	A	A	A	A	A	A	A	A	
AK Pollock	A	B	A	A	A	A	A	A	B
FL Spiny Lobster	B	B	A	A	A	A	A	B	A
Louisiana Shrimp	A	A	A	A	A	A	A	A	A
AK Salmon	A	A	A	A	A	A	A	A	A
NE Groundfish	A	A	A	A	A	A	A	A	A
CA Urchin	A	B	B	A	A	A	A	B	A
OR Dungeness Cra	A	A	A	A	A	A	A	A	A
AK Crab	B	B	B	A	A	A	A	A	A
AK Halibut	A	A	A	A	A	A	A	A	A
Pacific Groundfish	A	A	A	A	A	A	A	A	A
Thanh Hoa	C	C	B	A	A	A	A	A	A
Southern Zone Roc	A	A	A	A	A	A	A	A	A
Western Zone Aba									

Hoki	A	A	A	A	A	A	A	A	A
Cod	A	A	B	A	A	A	A	A	A
Purse Seiners	A	A	B	A	A	A	A	A	A
Anchovy	A	A	A	B	A	A	A	A	B
Blue Crab	A	A	A	A	A	A	A	A	A
Artisanal	A	A	A	B	A	A	B	B	A
Artisanal Ngaparo	B	B	B	B	B	A	A	A	A
Inshore Artisanal	A	A	A	A	A	A	A	A	A
Semi-Industrial	A	A	A	A	A	A	A	A	A
Sea Cucumber	A	A	A	A	A	A	A	A	A
IO Purse Seine Tun	A	A	B	B	A				
Sherbro	b	b	b	b	b				
Tombo	b	b	b	a	b				
Baltic Cod	A	B	B	A	C	B	A	B	B
Longline Tuna	B	A	B	A	B	C	A	B	B
Lake Victoria Daga	A	A	A	A	A				
Lake Victoria Tilapi	A	A	A	A	A				
Nile Perch				A	A				
AK Pollock	A	A	B	A	A	B	A	A	B
FL Spiny Lobster	A	A	B	A	B	A	B		B
Louisiana Shrimp	A	A	A	A	A	A	A	A	A
AK Salmon	A	A	A	A	A	A	A	A	A
NE Groundfish	A	A	A	A	A	A	A	A	A
CA Urchin	A	A	B	B	A				
OR Dungeness Cra	A	A	A	A	A	A	A	A	A
AK Crab	A	A	A	A	A	A	A	A	A
AK Halibut	A	A	B	B	A	A	A	A	B
Pacific Groundfish	A	A	A	A	B	A	A	A	A
Thanh Hoa	B	B	B	A	A	A	A	A	A
Southern Zone Ro	A	A	A	A	A	A	A	A	A
Western Zone Aba					A				

Hoki	A	A	A	A	A	A	A	A	A
Cod	A	A	B	C	C	B	B	B	B
Purse Seiners	A	A	B	C	C	B	B	B	B
Anchovy	B	B	B	B	A	A	B	B	B
Blue Crab	A	A	A	A	B	A	B	B	A
Artisanal	A	B	B	A	C	C	A	B	B
Artisanal Ngaparou	B	B	B	A	B	C	A	A	A
Inshore Artisanal	A	A	B	B	A	A	A	A	B
Semi-Industrial	A	A	B	B	A	A	A	A	B
Sea Cucumber	A	A	B	B	A	A	A	A	B
IO Purse Seine Tun		B	A	B	C	A	B	B	A
Sherbro		b	b	a	b	c	a	a	a
Tombo		b	a	a	a	c	a	a	a
Baltic Cod	A	A	A	C	B	C	B	B	C
Longline Tuna	A	B	C	C	C	B	C	C	C
Lake Victoria Daga		A	A	A	A	B	B	B	B
Lake Victoria Tilapi		A	A	A	A	B	B	B	B
Nile Perch		A	A	A	A	B	B	B	B
AK Pollock	A	A	A	B	A	B	B	B	A
FL Spiny Lobster	B	A	A	B	B	B	A	A	B
Louisiana Shrimp	A	C	B	B	A	B	A	A	A
AK Salmon	A	B	B	B	B	B	B	B	B
NE Groundfish	A	A	B	A	A	A	B	B	B
CA Urchin		C	B	C	C	B	C	C	C
OR Dungeness Cra	A	A	A	A	A	B	A	A	B
AK Crab	A	A	A	A	C	C	A	B	C
AK Halibut		B	B	B	B	A	B	B	B
Pacific Groundfish	A	A	C	B	B	A	B	B	C
Thanh Hoa	A	B	B	B	A	C	C	B	C
Southern Zone Roc	A	A	A	A	A	A	A	A	A
Western Zone Aba									

Hoki	A	B	A	B	B	A	A	A	A
Cod	B	B	C	C	B	B	A	A	A
Purse Seiners	B	B	C	C	B	B	A	A	A
Anchovy	B	A	A	B	B	B	B	B	A
Blue Crab	A	A	A	B	B	A	A	A	A
Artisanal	B	B	A	A	B	B	A	B	B
Artisanal Ngaparou	B	A	A	B	A	A	A	A	A
Inshore Artisanal	B	A	B	A	A	B	A	A	A
Semi-Industrial	B	A	B	A	A	B	A	A	A
Sea Cucumber	B	A	B	A	A	B	A	A	A
IO Purse Seine Tun	A	A	B	B	A	A	C	A	B
Sherbro	b	a	a	c	a	a	a	a	a
Tombo	a	a	a	c	a	a	a	a	a
Baltic Cod	C	C	C	B	A	A	A	A	A
Longline Tuna	C	A	A	A	B	A	C	B	B
Lake Victoria Daga	B	B	B	B	A	A	A	B	A
Lake Victoria Tilapi	B	B	B	B	A	A	A	B	A
Nile Perch	B	A	B	B	A	A	A	A	A
AK Pollock	A	A	A	B	A	A	A	A	A
FL Spiny Lobster	A	A	B	A	A	A	A	A	A
Louisiana Shrimp	A	A	A	C	B	C	B	A	A
AK Salmon	B	A	B	B	A	A	B	A	B
NE Groundfish	A	A	A	B	A	A	A	A	A
CA Urchin	C	B	C	B	A	B	C	B	B
OR Dungeness Cra	A	A	A	A	A	A		A	A
AK Crab	C	C	C	C	A	B	B	A	A
AK Halibut	B	A	B	B	A	A	A	A	A
Pacific Groundfish	C	A	B	B	A	A	A	A	A
Thanh Hoa	B	B	B	B	A	A	C	B	B
Southern Zone Roc	A	A	A	A	A	A	A	A	A
Western Zone Aba									

Hoki	A	B	B	B	B	B	A	B	B
Cod	B	B	B	B	A	A	B	A	A
Purse Seiners	B	B	C	B	A	A	B	A	A
Anchovy	A	A	B	A	B	B	B	A	A
Blue Crab	B	A	A	A	A	A	A	B	B
Artisanal	A	B	A	B	B	B	B	B	A
Artisanal Ngaparou	B	B	A	A	A	A	A	A	A
Inshore Artisanal	A	A	A	A	A	A	A	A	A
Semi-Industrial	A	A	A	A	A	A	A	A	A
Sea Cucumber	A	A	A	A	A	A	A	A	A
IO Purse Seine Tun	A	A	A	A	A	B	A	A	A
Sherbro	b	b	a	a	a	a	a	a	a
Tombo	b	a	a	a	a	a	a	a	a
Baltic Cod	B	B	A	A	A	A	C	B	A
Longline Tuna	A	A	A	B	B	A	A	A	A
Lake Victoria Daga	A	A	A	A	A	A	A	A	A
Lake Victoria Tilapi	A	A	A	B	A	A	A	A	A
Nile Perch	A	A	A	B	A	A	A	A	A
AK Pollock	B	A	A	A	B	B	B	B	A
FL Spiny Lobster	A	A	A	B	B	B	C	A	B
Louisiana Shrimp	C	B	B	A	A	B	A	B	A
AK Salmon	B	B	A	B	B	B	B	A	B
NE Groundfish	A	A	B	A	A	B	A	A	A
CA Urchin	B	B	A	B	C	C	B	C	C
OR Dungeness Cral	A	A	B	A	A	A	A	A	A
AK Crab	A	A	B	B	B	B	B	C	C
AK Halibut	A	B	A	B	A	A	A	A	A
Pacific Groundfish	A	A	A	A	C	A	C	C	C
Thanh Hoa	A	A	A	A	A	C	B	B	B
Southern Zone Roc	A	A	A	A	A	A	A	A	A
Western Zone Aba									

Hoki	B	A	A	B	A	A
Cod	A	B	B	B	A	A
Purse Seiners	A	B	B	B	A	A
Anchovy	A	B	B	B	B	B
Blue Crab	A	A	A	A	A	A
Artisanal	B	A	A	A	A	A
Artisanal Ngaparou	B	A	A	A	A	A
Inshore Artisanal	A	A	A	A	A	A
Semi-Industrial	A	A	A	A	A	A
Sea Cucumber	A	A	A	A	A	A
IO Purse Seine Tun	B		A	A	B	A
Sherbro	b	a	a	a	a	a
Tombo	b	a	a	a	a	a
Baltic Cod	A	A	A	B	A	A
Longline Tuna	A	A	A	A	B	A
Lake Victoria Daga	A	A	A	A	A	A
Lake Victoria Tilapi	A	A	A	A	A	A
Nile Perch	A	A	A	A	A	A
AK Pollock	A	B	A	A	A	A
FL Spiny Lobster	B	B	A	A	A	A
Louisiana Shrimp	A	A	A	A	A	B
AK Salmon	B	A	A	B	A	B
NE Groundfish	A	A	A	A	A	A
CA Urchin	C	B	B	C	A	A
OR Dungeness Cral	A	A	A	A	A	A
AK Crab	A	A	A	A	A	A
AK Halibut	B	B	A	A	B	A
Pacific Groundfish	C	A	A	C	A	A
Thanh Hoa	C	A	A	A	B	A
Southern Zone Roc	A	A	A	A	A	A
Western Zone Aba						
