# Supplementary material for: The Fishery Performance Indicators: A Management Tool for Triple Bottom Line Outcomes
Source: PLoS One. 2015 May 6;10(5):e0122809. doi: 10.1371/journal.pone.0122809 (PMC4422616; doi:10.1371/journal.pone.0122809)
Supplement: S2 Supporting Information — Detailed manual for applying and scoring FPI metrics. (PDF) [file pone.0122809.s004.pdf]

Fishery Performance Indicators

Version 1.2

James L. Anderson[1], Christopher M. Anderson[2], Jingjie Chu[3] & Jennifer Meredith[4]

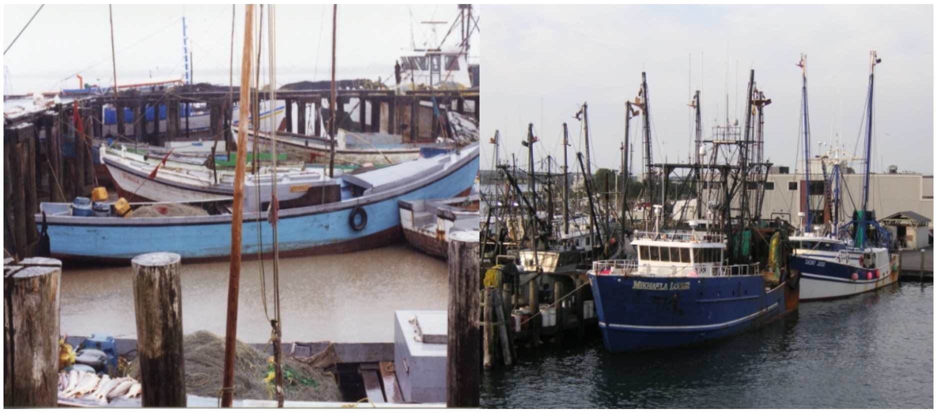

Revised
August 22, 2014

[1] Natural Resource Economist, Fisheries and Aquaculture Adviser, World Bank, Washington, DC
[2] Associate Professor, School of Aquatic & Fishery Sciences, University of Washington, Seattle, WA
[3] Natural Resource Economist, Africa Region, World Bank, Washington, DC
[4] PhD Student, Economics Department, University of Washington, Seattle, WA

Fishery Performance Indicators

Version 1.2

James L. Anderson[5], Christopher M. Anderson[6], Jingjie Chu[7] & Jennifer Meredith[8]

This project was funded by

ALLFISH (Alliance for Responsible Fisheries), a public-private partnership created by the seafood industry, the World Bank, the UN's Food and Agriculture Organization, and the Global Environment Facility

Cover Photos: James L. Anderson

Revised
August 22, 2014

[5] Natural Resource Economist, Fisheries and Aquaculture Adviser, World Bank, Washington, DC
[6] Associate Professor, School of Aquatic & Fishery Sciences, University of Washington, Seattle, WA
[7] Natural Resource Economist, Africa Region, World Bank, Washington, DC
[8] PhD Student, Economics Department, University of Washington, Seattle, WA

PRACTICAL SCORING GUIDANCE

COMPLETING THE FPI WORKSHEET IN EXCEL

1. Fill in the first tab of the worksheet "Cover Page" with country, location, fishery, single/multi species, species names, date, and author information.

2. It is essential to fill in the column of quality scores for both the input and output tables. Note the quality score guidelines:

   A: Reviewer is highly confident (95%) the 1-5 score is correct. Confidence can come from familiarity with the fishery, the reliability of another expert source, a calculation based on reliable data, or large ranges of the underlying metric for the given score that make another score highly unlikely for the fishery. Note that it is confidence in the 1-5 score that matters, and thus wide ranges for the underlying metric associated with a score can support "A" quality, even in the case when information about the precise level of the underlying metric is poor.

   B: Reviewer feels 1-5 score is more likely than others, and reviewer is highly confident (95%) that the true underlying metric would be within one of the given score.

   C: Reviewer is making an educated guess based on best available information, but reviewer is not highly confident the true metric would be within one of the given score.

   Note that uncertainty about the interpretation of the metric should be resolved through consultation with the FPI materials or personnel, rather than giving the score a lower quality. Interpretations can be explained in the notes.

3. All metrics should be scored with a 1, 2, 3, 4, 5 or NA. Intermediate scores of 1.5, 2.5, etc. are not acceptable. NA is only acceptable if the metric truly does not apply to the fishery (example: in a fishery with no harvest rights you should score the transferability of harvest rights as NA). If a score cannot be given due to missing data, the metric should not be left blank: an educated guess as to the score should be made and the metric should be given a quality score of C.

4. The explanation column should be filled in for each metric so that reviewers know the rationale behind the given score. Explanations can be brief but it should be clear from the explanation and data source column which information sources are being used.

5. An FPI Fishery Profile should be completed for each fishery in order to provide important context and background information for the scores. Completion of the fishery profile does not mean that scores no longer require an explanation in the worksheet.

II. FISHERY PERFORMANCE INDICATORS

Outputs

MEASURING ECOLOGICAL, ECONOMIC, AND COMMUNITY OUTCOMES

II. Fishery Performance Indicators — Outputs

This section identifies 68 metrics of ecosystem health and human well-being (see Table 1 below). Each metric (found in the third column in Table 1) is individually explained in the following pages. To facilitate scoring, the metrics are organized in the manual according to the sector partitioning, as data on each sector tends to be available from similar sources. The last column in Table 1 indicates the whether the metric fits into ecological, economic, or community performance. Each indicator is presented alongside practical scoring guidance and examples that are derived from the existing set of case studies or from theoretical situations that could arise.

Table 1. Fishery Performance Indicators—Outputs

Indicator	Dimension	Metric
Stock Performance	Fish Stock Health & Environmental Performance	Percentage of Stocks Overfished
		Degree of Overfishing
		Stock Declining, Stable or Rebuilding
		Regulatory Mortality
		Selectivity
		Illegal, Unregulated or Unreported Landings
		Status of Critical Habitat
		Proportion of Harvest with a 3rd Party Certification
Harvest Sector Performance	Harvest Performance	Landings Level
		Excess Capacity
		Season Length
		Harvest Safety
	Harvest Asset Performance	Ratio of Asset Value to Gross Earnings
		Total Revenue Compared to Historic High
		Asset (Permit, Quota) Value Compared to Historic High
		Borrowing Rate Compared to Risk-free Rate
		Source of Capital
		Functionality of Harvest Capital
	Risk	Annual Total Revenue Volatility
		Annual Landings Volatility
		Intra-annual Landings Volatility
		Annual Price Volatility
		Intra-annual Price Volatility
		Spatial Price Volatility
		Contestability & Legal Challenges
	Owners, Permit Holders & Captains (Those holding the right or ability to access)	Earnings Compared to Regional Average Earnings
		Fishery Wages Compared to Non-fishery Wages
		Social Standing of Boat Owners and Permit Holders
		Education Access
		Access to Health Care
		Proportion of Nonresident Employment
	Crew (Those depending on others for access)	Earnings Compared to Regional Average Earnings
		Fishery Wages Compared to Non-fishery Wages
		Social Standing of Crew
		Education Access
		Access to Health Care
		Proportion of Nonresident Employment
		Crew Experience
		Age Structure of Harvesters
Post Harvest Sector Performance	Market Performance	Ex-vessel Price Compared to Historic High
		International Trade
		Final Market Wealth
		Wholesale Price Compared to Similar Products
		Capacity of Firms to Export to the US & EU
		Final Market Use
		Ex-vessel to Wholesale Marketing Margins
	Post-harvest, Processing & Support Industry Performance	Processing Yield
		Shrink
		Capacity Utilization Rate
		Product Improvement
		Sanitation
		Regional Support Businesses
	Post-Harvest Asset Performance	Borrowing Rate Compared to Risk-free Rate
		Source of Capital
		Age of Facilities
	Processing Owners & Managers	Earnings Compared to Regional Average Earnings
		Manager Wages Compared to Non-fishery Wages
		Social Standing of Processing Managers
		Education Access
		Access to Health Care
		Nonresident Ownership of Processing Capacity
	Processing Workers	Earnings Compared to Regional Average Earnings
		Worker Wages Compared to Non-fishery Wages
		Social Standing of Processing Workers
		Education Access
		Access to Health Care
		Proportion of Nonresident Employment
		Worker Experience

Dimension	Indicator
Ecologically Sustainable Fisheries	Ecology
Harvest	Economics
Harvest Assets	
Risk	
Trade	
Product Form	
Post-Harvest Asset Performance	
Managerial Returns	Community
Labor Returns	
Health & Sanitation	
Community Services	
Local Ownership	
Local Labor	
Career	

STOCK PERFORMANCE

ECOLOGICALLY SUSTAINABLE FISHERIES

Percentage of Stocks Overfished

RATIONALE:
The percentage of stocks considered to be overfished reflects the extent to which overfishing has compromised the ability to generate sustainable livelihoods. Overfished stocks cannot be harvested at a level that maximizes incomes until they are recovered.

METRIC:
Percentage of commercial stocks within the management plan that are considered overfished, to be experiencing overfishing, or in generally unknown stock status (whether actively managed or not).

5	None overfished
4	1-25% of stocks overfished
3	26-50% of stocks overfished
2	51-75% of stocks overfished
1	76-100% of stocks overfished

SCORING GUIDANCE:
Calculation: Number of overfished stocks/Number of stocks in fishery
This should be the proportion of stocks in the fishery (counting separately assessed stocks of the same species as different stocks) whose current biomass level indicates that they are overfished. Single stock fisheries will always be scored 1 or 5. (Whether the stock is currently recovering or being overfished is the next metric.) There are single species fisheries with more than one stock that could receive a score between 1 and 5.

It may be difficult to disentangle stock decline arising from overfishing, rather than other sources of decline such as pollution. Attempt to get data on catch per unit effort (CPUE), historic number of vessels, days at sea, or other effort variables and look at trends over time. In an open access fishery, it is highly likely that declining stocks can be attributed to overfishing.

For US fisheries, refer to: http://www.nmfs.noaa.gov/fishwatch/, which specifically classifies stocks.

Technical definitions from NMFS:

Subject to overfishing: A stock that has a harvest rate above the level that provides for the Maximum Sustainable Yield (MSY).

Overfished: A stock that has a biomass level below its prescribed biological threshold (typically 50% of B_{MSY} or the minimum stock size at which rebuilding to B_{MSY} will occur within 10 years when fishing at the maximum fishing mortality threshold.

EXAMPLES:

- A multispecies artisanal fishery in Bangladesh received a 3 because one of the main species was not biologically threatened while other species were threatened by overfishing and the introduction of invasive stocked species.
- The multispecies New England groundfish fishery received a 2 because out of its 21 stocks, 6 were not overfished (based on stock assessment) which means that 15/21 or 71% were overfished.
- The Alaska Salmon fishery has five species with strictly enforced regulations; it scored a 5 because there are different escapement goals for each individual stock within all five species and nearly all river-specific stocks meet their escapement goals.

STOCK PERFORMANCE

ECOLOGICALLY SUSTAINABLE FISHERIES

Degree of Overfishing – Stock Status

RATIONALE:

The current status of overfishing reflects the extent to which diminished stock levels have compromised the fisheries' ability to generate sustainable incomes. Overfished stocks cannot be harvested at a level that generates sustainable incomes until they are recovered. Distinguishing between stocks that are severely overfished and those that are only mildly overfished is important because it allows researchers to more precisely determine the ecological state of the fishery resources.

METRIC:

Current status of stock. Two alternate scoring systems are offered. The first is for fisheries where stock levels are not known with any precision. The second is for fisheries where scientific stock assessments are being conducted and measures of B (biomass level) and B_{MSY} (biomass level required to obtain maximum sustainable yield) are available. Bins were based on a paper (Costello et al. 2012) that estimated median B/ B_{MSY} for assessed fisheries of 0.97 and unassessed fisheries of 0.64.

5	Stock is not overfished or is rebuilt; $B/B_{MSY} \geq 1$
4	Stock is mildly overfished ; $0.75 \leq B/B_{MSY} < 1$
3	Stock is moderately overfished ; $0.5 \leq B/B_{MSY} < 0.75$
2	Stock is seriously overfished; $0.25 \leq B/B_{MSY} < 0.5$
1	Stock is severely overfished and in danger of collapse; $0.25 > B/B_{MSY}$

SCORING GUIDANCE:

For multi-stock fisheries, score each of the top three significant stocks 1-5 then take a value-weighted average (determine the percentage contribution of each species to total revenue and then weight by this fraction). Based on pilot case studies, an open access fishery will rarely score above a 3. The following website may be helpful: http://www.fao.org/fishery/countryprofiles/search/en

EXAMPLES:

- A single species crab fishery in Indonesia where stock assessments are not conducted has seen increasing effort levels coupled with declining catch; there is no concrete data on stock levels but they score the fishery a 2 with a quality score of B because they know that the fishery is definitely worse than mildly overfished.
- A single species tuna fishery in the East Pacific has recent scientific estimates of B/ B_{MSY} that indicate that this ratio is 0.6 so the fishery scores a 3 with a quality score of A.
- The Alaska Pollock fishery scored a 5 because the stocks are stable at high levels and there is a strict management in place to ensure that they are not overfished.
- Consider the data from a multispecies coastal fishery in Kenya:

English Name	Family Name	Kg	KES	USD	Percentage Revenue
Rabbit fish	*Siganidae*	49,226	7,745,597	109,876	0.380362
Scavengers	*Lethrinidae*	36,610	5,608,830	65,986	0.244283
Parrot fish	*Scaridae*	31,383	4,676,830	55,022	0.203694
Mixed pelagics		19,663	2,886,050	33,954	0.125699
Mackerel	*Scombridae*	14,819	2,042,880	24,034	0.088975

Five species are harvested, but the top three on the chart make up more than 80% of total revenue so ignore mixed pelagics and mackerel and focus on the remaining three species. Rabbit fish scores a 3 but scavengers and parrot fish score a 1. The final score for this fishery is 3*(.3373) + 1*(.2442+.2037) = 1.56 which is rounded up to a 2.

STOCK PERFORMANCE

ECOLOGICALLY SUSTAINABLE FISHERIES

Declining, Stable or Rebuilding – Stock Dynamics

RATIONALE: The current status of overfishing reflects the extent to which diminished stock levels have compromised the fisheries' ability to generate sustainable incomes. Overfished stocks cannot be harvested at a level that generates sustainable incomes until they are recovered. Distinguishing between stocks that are being rebuilt and stocks that continue to be overfished is important as it allows researchers to more precisely determine the ecological state of the fishery resources.

METRIC:

Extent to which current effort levels affect stock status. This metric is scored by taking the fishery's score in the previous question and then adding or subtracting points depending on whether the fishery is rebuilding or declining. The maximum score for this metric is a 5 and the minimum score is a 1. If the fishery scored a 5 in the previous question, the score here is automatically a 5. If the fishery scored less than 5 in the previous metric, then take the fishery's score from the previous metric and apply the following:

+2	Stock is rapidly rebuilding
+1	Stock is rebuilding
+0	Stock is stable
-1	Stock is declining
-2	Stock is rapidly declining

SCORING GUIDANCE:

Note that rapid rebuilding should imply more than one bin change (in the previous question) within the past 3 years. For example, if New England groundfish scored a 3 for current Degree of Overfishing and current stock assessments indicate that the stock has been rapidly rebuilding. In this case, we would expect that the fishery had a B/B_{MSY} less than 0.25 and scored a 1 three years ago.

For multi-stock fisheries, score each of the top three significant stocks 1-5 then take a value-weighted average (determine the percentage contribution of each species to total revenue and then weight by this fraction).

EXAMPLES:

- The Alaska Pollock fishery is not overfished and $B/BMSY \geq 1$ so the fishery scored a 5 in the previous question. Since it scored a 5 in the previous question it automatically scores a 5 in this question as well.
- The Mexican chocolate clam fishery was seriously overfished so it scored a 2 in the previous metric, however there are strict no-take areas in place which means that the stock is rapidly rebuilding so it scores a 4 in this metric (add 2 to the previous metric's score).

- Consider the Kenyan multispecies example from the previous metric. Rabbit fish were moderately overfished (score of 3) while the other top two species were severely overfished (score of 1). A TURF has been established and scavengers are stable while rabbit fish and parrot fish stocks are in decline. This fishery would score a 2 for rabbit fish, 1 for scavengers and 1 for parrot fish (since 1 is the minimum); a value weighted average of these 3 scores (.38*2+.24*1+.20*1= 1.2) leads to a score of 1 for this metric.

Stock Performance

Ecologically Sustainable Fisheries

Regulatory Mortality

Rationale:

Regulatory mortality is a direct measure of waste and potentially foregone income. This represents fish that possibly could have been sold, but were not. Estimating the impact of regulation on fish stock health allows management systems to measure potential downsides to management regime shifts.

Metric:

Ratio of estimated regulatory mortality to actual landings of the assessed target species. Regulatory mortality is defined as fish loss that is induced by regulation (such as size restrictions).

5	No regulatory mortality of the target species
4	Regulatory mortality is less than 5% of total catch
3	5-25%
2	25-50%
1	For every 100 lbs of fish caught, more than 50 lbs are discarded

Scoring Guidance:

Note that in fisheries where there is no management restriction on size or quantity of fish, we would not expect to see any regulatory mortality.

Examples:

- In many artisanal fisheries, the fishermen land every fish that they catch and all of these fisheries score a 5.
- In the New England groundfish fishery, there is evidence of high-grading and species-grading as fishermen throw back flounder that are not a good use of the trip limit or that are too small to land legally and the fragile fish do not survive. Suppose that in the flounder fishery, 30% of the fish are thrown back due to size regulations and only 20% of the fish thrown back survive. This means that 2.4 out of every 10 fish caught are killed through regulation, and regulatory mortality is 24% of total catch so this fishery scores a 3.
- Suppose that in a lobster fishery 40% of the catch is thrown back due to size regulations but the fish are much hardier so 90% of the discards survive. Only 0.4 out of every ten lobsters landed are killed through regulation and regulatory mortality is 4% so this fishery would score a 4.
- In a shellfish fishery in Mexico, there are no size restrictions, but harvesters are required to help the authorities conduct a stock assessment each season that requires removing shellfish from the water and measuring them. Very rarely, this practice causes shellfish to die, but this is scientific rather than commercial mortality so it does not impact the score and the fishery scores a 5.

STOCK PERFORMANCE

ECOLOGICALLY SUSTAINABLE FISHERIES

Selectivity

RATIONALE:

The selectivity of the fishery is a measure of ecological well-being because in highly selective fisheries where non-target catch is low, harvest imposes no direct externalities on other species or on the ecosystem as a whole. There are fisheries where selectivity is an important issue in the decision-making of harvesters and management because the catch of non-target species imposes heavy costs on harvesters and is frequently the subject of scrutiny from environmental groups and stakeholders in the non-target fishery.

METRIC:

Percentage of total catch that is made up of non-target species. Note that non-target species are distinct from multi-species fisheries in that the catch of non-target species does not increase the value of fishing or imposes costs on the target fishery.

5	There is virtually no non-target catch
4	Less than 5% of catch is non-target species
3	5-25%
2	25-50%
1	For every 100 lbs of fish caught, more than 50 lbs are non-target species

SCORING GUIDANCE:

Note that in artisanal fisheries where every species caught is landed and consumed, there is typically no non-target catch so the fisheries score a 5. In fisheries where there is concern about the gear ensnaring marine mammals or seabirds, this would be counted as non-target catch. The calculation should be weight of non-target catch / total weight of catch (it is not based on value).

EXAMPLES:

- In the Gambian sole fishery, the gear is not selective and 25-50% of landings are non-target catfish so the fishery scored a 2.
- In the Alaska Pollock fishery, the trawl gear leads to less than 5% of catch being composed of non-target salmon which must be counted by observers and could cause the Pollock fishery to be closed once the catch limit is reached so the fishery scores a 4.
- The Florida spiny lobster fishery uses traps that are highly selective and there is virtually no non-target catch so the fishery scores a 5.
- Diving for sea urchins in the California fishery or for shellfish in a Mexican fishery has nearly perfect selectivity and has no potential for non-target catch so these fisheries score a 5.

Stock Performance
Ecologically Sustainable Fisheries

Illegal, Unregulated or Unreported Landings

Rationale:
IUU is a direct measure of waste and potentially foregone ecosystem health. This represents fish that are being harvested outside of the fishery's management jurisdiction and may represent foregone income for the local community. The proportion of IUU catch gives an estimate of the ability of management to assess the stock and enforce regulation.

Metric:
Proportion of landings from the managed stock using illegal gear, area, methods, etc., or that goes unreported or falls outside of the regulatory structure.

5	There is virtually no IUU catch
4	Less than 5% of catch is IUU
3	5-25%
2	25-50%
1	For every 100 lbs of fish caught, more than 50 lbs are IUU

Since this is a metric of unreported and illegal activity, it will by definition be difficult to obtain hard data, but it should be possible to get an estimate of whether and how frequently such activity occurs. If there is no regulatory reporting requirement, then there is no unreported catch for the purposes of this metric. Similarly, if there are no regulations on size, gear, area, etc., then there is no violation that would be considered illegal or unregulated. In fisheries where there is competition from recreational fishers that fall outside the commercial fisheries' regulations, this would be sometimes be considered unregulated catch that should be counted in this metric. If the recreational fishery falls within historical norms for the allocation of the recreational sector and has effective management then this does not count as IUU. However, if the recreational sector has no limit on the amount of fish they can land or routinely exceeds their allocation then this would count as IUU. In fisheries where there are no formal or written regulations but where fishermen have an agreement not to target juveniles or not to use certain types of gear, then violation of these informal agreements should be considered IUU.

Examples:
- In a Malawian lake fishery, 25% of landings come from vessels that are unregistered and 10% of landings come from the use of illegal seine nets that have been outlawed by the beach management unit. This fishery has 35% IUU and scores a 2.
- The Alaska crab fishery has a high degree of observer coverage which leads to all catch being reported and no illegal fishing methods so this fishery scored a 5.
- In the Seychelles sea cucumber fishery, poaching is common as is the use of prohibited spear guns. In addition, unlicensed fishermen often intrude in the limited access fishery which led to an IUU estimate of 20% and the fishery scored a 3.
- In the Tokyo Bay trawl fishery in Japan, there is competition from recreational harvesters who can harvest an unlimited number of fish and have recently been increasing their

catch. This is considered IUU and since it represents less than 5% of catch, the fishery scores a 4.

Stock Performance

Ecologically Sustainable Fisheries

Status of Critical Habitat

Rationale:
The status of the fish stock's critical habitat is a direct measure of the ecological benefits that are accumulating in this fishery. Ecological well-being is measured not only by the health of the stock but by the health of the entire ecosystem and the habitat status also indicates whether the ecological state is sustainable.

Metric:
Portion of critical habitat that is damaged or dysfunctional. Critical habitat is defined as that playing a significant role in the life cycle of the fish. Portion damaged is based on area, and all sources of damage are considered, including fishing damage, pollution, and development.

5	Critical habitat is healthy and not threatened
4	Less than 25% is degraded or dysfunctional
3	25-75% is degraded or dysfunctional
2	More than 75% of critical habitat is destroyed
1	Nearly all critical habitat is damaged or dysfunctional

Scoring Guidance:
Note that this metric refers to the status of the habitat, not the fish stock. If areas near shore are polluted, but the fish lives and breeds in deep water then this pollution does not contribute to the life cycle of the fish and should not be considered critical habitat.

Examples:
- In the Alaska pollock fishery, the critical habitat is the Bering Sea which is not considered to be threatened and so the fishery was given a 5.
- In a Japanese shrimp fishery, pollution from industrial waste water (paper mills in particular) and other urban water pollution is present, but the relative size of the unpolluted bay led to the fishery scoring a 4.
- In the Kenya octopus fishery, the critical habitat is coastal reefs and the majority of these reefs have been degraded so the fishery scored a 3.
- In an artisanal fishery in Sierra Leone, which is located close to major cities, the critical habitat is affected by mangrove deforestation, coastal erosion, mining, and agricultural activities. Estimates given by the community led to the fishery scoring a 3.

Stock Performance

Ecologically Sustainable Fisheries

Proportion of Harvest with a Third Party Certification

RATIONALE:
Fish stocks must be healthy in order to generate sustainable returns and one goal of certification programs is to ensure that the resource is being harvested sustainably. Certification may also be essential for market access in developed countries.

METRIC:
The proportion of harvest (quantity) harvested under one of the recognized third party programs that certify ecological sustainability, such as the Marine Stewardship Council (MSC) certification.

5	76-100% of landings are certified
4	51-75%
3	26-50%
2	1-25%
1	No landings have third party certification

SCORING GUIDANCE:
In a multi-species fishery where only a portion of the species are certified, weight individual species by their proportion of landings value. Any certification program that meets *The Food and Agriculture Organization (FAO) Code of Conduct for Responsible Fisheries* (1995) and *The FAO Guidelines for Ecolabelling of Fish and Fishery Products from Marine Capture Fisheries* (2005/2009) will count as being certified. Certification by local or federal governments does not count. This does not include Friends of the Sea certification. This also does not include pocket/wallet cards. However, fisheries that have entered Sustainable Fisheries Partnership's Fishery Improvement Programs (http://www.sustainablefish.org/fisheries-improvement) will count as half-certified. Fisheries that have only undergone pre-assessment are not certified.

EXAMPLES:

- The Peruvian anchovy fishery is not MSC certified but all of Peruvian anchovy landings have been entered into a Fishery Improvement Program so this fishery should score a 3 (100% of landings in improvement program which counts at 50% certified = 50%).
- The US West Coast Dungeness crab fishery scored a 3 because out of the three states where crab is landed, only Oregon is MSC certified and Oregon's landings make up 26-50% of the total catch.

Harvest Sector Performance

Harvest Performance

Landings Level

Rationale:
Harvests at the level of maximum economic yield (MEY) reflect management and/or harvest policies that reflect economic goals. This is primarily a measure of the extent to which the fishery is realizing its potential wealth over time, ensuring the future reproductive value remains in the water.

Metric:
Average annual harvest over the last three years.

5	Harvest is less than MSY (stock is above MSY level) to increase profit
4	Harvest is approximately at MSY
3	Harvest reduced to promote recovery
2	Harvest is constraining stock recovery
1	Harvest is causing overfishing (stock is below MSY and declining)

Scoring Guidance:
Note that this metric refers to MSY but there are many fisheries where the lack of stock assessments and reliable data mean that estimates of MSY are unattainable. In such fisheries, attempt to discern the management's goals when setting total allowable catch or making other regulations. Most fully-exploited open access fisheries with no limit on entry, effort, or catch will score a 1 or 2 dependent on effort levels. Only fisheries where management intervenes out of concern for stock levels or falling incomes should receive higher than a 2, unless it is an example of an underexploited fishery. In fisheries where the main goal is biological sustainability (maintain the maximum amount of fish) then the score should be a 4. Only fisheries that actively try to manipulate markets and harvest fewer fish in order to gain a higher price should receive a 5.

Examples:

- Although there were no estimates of MSY in the Kenyan octopus fishery, the trend in landings had been declining over time as effort had been increasing with no management attempts to decrease effort so the fishery scored a 1.
- In the Ugandan Nile Perch fishery, harvests are stable at low levels so the fishery scored a 2.
- Processors in the Alaska pollock fishery actively advocate for the managing agency to set lower TACs below the ABC in abundant years so that harvests are below MSY and they make higher profits, indicating a 5.
- In the California sea urchin fishery, the lack of a TAC and coarse input controls, make it likely that total stock is below MSY level but efforts to limit harvest mean that this fishery scores a 3.

- Many regulated fisheries such as Norwegian cod or Alaskan salmon where biologists help set the TAC in order to ensure that harvest levels do not threaten biological sustainability score a 4.
- In a Mexican shellfish fishery, the harvest levels had been drastically reduced in order to promote recovery so this fishery scored a 3.

HARVEST SECTOR PERFORMANCE

HARVEST PERFORMANCE

Excess Capacity

RATIONALE:
Excess capacity in the fishing fleet reflects management that has either allowed the stock to decline so that a once-efficient harvesting operation scale is now too large, or that has induced a derby wherein harvesters have had to purchase inefficiently large vessels, or both. These inefficiently large vessels are more expensive to operate and maintain than necessary, reducing wealth in the harvesting sector.

METRIC:
In the absence of a fishery-specific metric of overfishing, use estimated standardized vessel-days required to catch the maximum sustainable yield (MSY) compared to the number of standardized vessel-days available. Days are considered not to be restricted by trip limits.

5	Within 5% of days required; there is no evidence of excess capacity
4	90-95%
3	75-90%
2	50-75%
1	less than 50%, of days required; excess capacity imposes heavy costs in the fishery

SCORING GUIDANCE:
Calculation: # Vessel-days required to catch MSY/# Vessel-days available

What we are trying to estimate here is whether the fishery is spending too much to catch the fish that they are currently catching. If there are no estimates of the days it takes to catch MSY, then scores should be based on anecdotal evidence of a race to the fish resulting in an over accumulation of harvest capital or of declining stock levels leading to too many vessels with an inefficient level of catch per vessel. Fisheries that extract the maximum amount of economic wealth will have an efficient level of technology and number of participants so the ratio should be close to 1. We would expect to see the number of vessel-days to catch MSY being less than the number of vessel-days available (a ratio less than one) in fisheries where there are too many boats or where the boats are too large. There is no excess capacity if boats are scaled appropriately for their fishery: vessels being scaled to participate in other fisheries does not affect this.

EXAMPLES:

- The Alaska crab fishery scored a 5 because the number of vessels declined after the transition to quotas and currency capacity levels reflect a market-based quota equilibrium.
- In contrast, the Alaska salmon fishery is managed with limited entry and a significant derby occurs that leads to vessels being inefficiently large and a resulting score of 1.

- In a Mexican shellfish fishery, the stocks were so far depleted that harvesters estimated that they could catch their remaining quota in only five days while the fishery was open for 8 months. This fishery scored a 1.
- In the Alaska halibut fishery some of the vessels participate in multiple fisheries and larger vessels appropriate for the salmon and crab fisheries may be employed; this did not affect the score for excess capacity in this fishery and the fishery scored a 4.

HARVEST SECTOR PERFORMANCE

HARVEST PERFORMANCE

Season Length

RATIONALE:
The length of the season reflects the extent to which management allows harvesters to determine when to harvest and how much. Choosing how and when to harvest allows harvesters to land when the prices are highest, or to spread the harvest over a long period of time to stabilize ex-vessel prices at high levels, and allow processors to time product flow to implement efficient methods.

METRIC:
Ratio of number of days on which fishing occurs to the number of days the species is available in economically feasible quantities.

5	Virtually no regulatory closures
4	90-99%
3	50-90%
2	10-50%
1	Less than 10%

SCORING GUIDANCE:
This is primarily a measure of the extent of derby (including short regulatory seasons to limit total effort), not lack of biological availability or efficiency-enhancing closures to prevent within-season growth overfishing. In fisheries where there is a limit on total allowable catch, but no individual harvest rights, empirical evidence shows that harvesters will race and the season will close quickly which causes inefficiencies. In open access fisheries where harvesters are free to harvest anytime the score is typically a 5.

EXAMPLES:

- The Alaska halibut fishery used to be regulated with a TAC (and no ITQ) and the fishing season would close in 2 days. Now that an ITQ system has been established, the fishing season lasts for 8 months and the fishery scores a 4.
- The artisanal fishery in Sierra Leone has no TAC, but the chief fishermen insists that harvesters not fish on Tuesdays. This is an example of a regulatory closure as a result of derby and not one that is designed to prevent within-season growth overfishing so the fishery is open 6/7 or 86% of the time that the species is available and the fishery scores a 3.
- Suppose that in an artisanal fishery, one month out of the year is closed to fishing in order to allow stocks to rebuild, this is also an example of a shorter regulatory season to limit total effort so the ratio is 11/12=92% and the fishery would theoretically score a 4.
- In the Louisiana shrimp fishery, the shrimp grow larger throughout the season, and regulations are in place to ensure that harvesters do not fish until they can expect to harvest mature shrimp. This is an example of an efficiency-enhancing closure to prevent within-season growth overfishing and the fishery is not reduced in score.

Harvest Sector Performance

Harvest Performance

Harvest Safety

RATIONALE:

The extent to which harvesters are able to safely exploit fishery resources and then return to their families is a direct measure of well-being that accrues to the community. Hazardous fisheries where there is significant harvester mortality inflict emotional and financial burdens on the local community.

METRIC:

Number of harvester (captain or crew) on-the-job deaths, per thousand person fishing season. We consider there to be one season per year, but do not annualize mortality if the fishing season is less than a year.

5	Less than 0.1 deaths per thousand person season
4	Between 0.1-0.5 deaths per thousand person season
3	Between 0.5-1 deaths per thousand person season
2	Between 1-5 deaths per thousand person season
1	More than 5 deaths per thousand person season

SCORING GUIDANCE:

Calculation: # deaths per season / (# employed in fishery per season / 1000)

For large US fisheries, data can be found here:
http://www.cdc.gov/mmwr/preview/mmwrhtml/mm5927a2.htm?s_cid=mm5927a2_x#tab2

EXAMPLE:

- The Tokyo Bay trawl fishery in Japan has 75 participants and in the past 10 seasons there has been one death. The calculation is (1/10)/(75/1000) = 1.33 so the score is 2. This demonstrates that even when deaths are rare, if the number of participants is low then the fishery can score low in harvest safety.

HARVEST SECTOR PERFORMANCE

HARVEST ASSET PERFORMANCE

Ratio of Asset Value to Gross Earnings

RATIONALE:
In addition to income, fishery wealth can also accumulate to the harvesters through the value of the assets that allow access and participation in the fishery. The price of the privilege or right to access a fishery in the form of a vessel, license, lease or quota, is a direct measure of the accumulation of wealth from the fishery to the harvest sector. The price of access should reflect the present discounted value of the stream of profits arising from accessing the fishery. This will include expectations for changes in management, harvest levels, prices and harvesting costs. Gross earnings is used to normalize the asset value to the levels of the fishery. Gross earnings are a proxy for net earnings because cost data is rarely available, and this normalization is standard in agricultural frameworks. For a fixed level of gross earnings, if the fishery's income is highly uncertain, or costs are excessive, then the ratio will be lower.

METRIC:
Extent to which fishery wealth is accumulated in access capital (e.g., quota, permits or vessels). Ratio of average price of capital and licenses required to access the fishery over the last five years to the average annual gross earnings for a similarly scaled access right in the same period.

5	10 or higher
4	7.5-10
3	5-7.5
2	2.5-5
1	2.5 or below

SCORING GUIDANCE:
Calculation: Annual cost of access /Annual gross earnings per access capital (average for last five years)

Typically a 1 if vessels are small scale and are not limited by regulation; the highest scores are associated with tradable quota-based fisheries. When determining the price of capital same business or same family sales are excluded, where they can be identified. Try to capture at least 80% of the asset value required for access; for example, if participation requires a permit, vessel, and net, but data on net prices is hard to obtain, just focus on permit and vessel values. Even fisheries that are not managed with quota or permits will still require assets in order to access (vessels, traps, etc…). Use data on purchase value and not lease value of assets. The price of access should represent the cost to a new entrant and not the present value of existing harvesters' vessels/nets.

EXAMPLES:

- Suppose that in an artisanal fishery licenses, vessels and nets are required to access. The average price of a license is $20, a vessel $6000 and nets $300. The average vessel earns $2000 per season so the ratio is 6320/200 = 3.16 and the fishery would score a 2. Even if

data on license cost and nets was unavailable the score would be unchanged because the vessel price is the majority of the asset value.

- In the Alaska halibut and crab fisheries and the Norwegian purse seine fishery recent changes in regulations have led quota assets to accumulate value and these fisheries scored a 5.
- In many small scale fisheries, the revenue per vessel is always much greater than the value of the harvest capital and these fisheries score a 1.

HARVEST SECTOR PERFORMANCE

HARVEST ASSET PERFORMANCE

Total Revenue Compared to Historic High

RATIONALE:
If the fishery is generating wealth, it is expected that the total revenue for the fishery is likely to increase to some sustainable maximum range. Fisheries with declining total revenue are likely to be in decline as a result of overfishing, poor marketing, and distribution. In contrast, a fishery managed for wealth creation should be harvested sustainably, and the sector is likely to orient toward market access and innovation. This should be observable in stable or increasing total revenue.

METRIC:
The indicator is the ratio of total real revenue (in local currency) to the average of the three highest total real revenues in the past 10 years. Adjust by local CPI if inflation was significant.

5	Above 95 percent
4	85 to 95 percent
3	70 to 85 percent
2	50 to 70 percent
1	Below 50 percent

SCORING GUIDANCE:
In fisheries where there is no historic data on total revenue, try to get participants to estimate general trends about whether revenue has been rising or falling and by how much. If data is available, please report in Excel spreadsheet's 'Historical Data' tab and fill in data as below.
For US fisheries: http://www.st.nmfs.noaa.gov/st1/commercial/landings/annual_landings.html

EXAMPLES:
Suppose the following data comes from an artisanal fishery in Liberia:

Total Revenue:		Average inflation rate: 6% (no need to adjust by local CPI)
2012	200,000 LRD	Average of 3 highest: 225,000 LRD
2011	198,000 LRD	Ratio: 200,000 LRD (current)/225,000 LRD = 0.89
2010	180,000 LRD	Score is a 4
2009	**210,000 LRD**	
2008	**230,000 LRD**	
2007	**235,000 LRD**	
2006	200,000 LRD	
2005	190,000 LRD	
2004	120,000 LRD	
2003	100,000 LRD	

Some fisheries that scored a 5: Alaska halibut, Uganda Nile Perch, and Icelandic lobster; These fisheries have seen successful management regimes generate increasing revenue or increasing price trends.

3: Alaska salmon, Pacific groundfish; Although stocks are well-managed, there is competition from aquaculture and other fisheries that has led to falling prices.
1: Louisiana shrimp, New England groundfish; Declining stocks led to very low revenue in these fisheries.

HARVEST SECTOR PERFORMANCE

HARVEST ASSET PERFORMANCE

Asset Value Compared to Historic High

RATIONALE:
If the fishery is generating wealth, it is expected that the value of the physical capital, permit, quota, or other right to the fishery is likely to increase to some sustainable maximum range. Fisheries with declining assets are likely to be in decline as a result of overfishing, poor marketing, distribution, or other constraints to innovation. In contrast, a fishery managed for wealth creation should be harvested sustainably; the sector is likely to orient toward improved marketing and innovation.

METRIC:
The indicator is the ratio of the current value of the asset to the average of the three highest asset values in the past 10 years. Adjust by local CPI if inflation was significant

5	Above 95 percent
4	85 to 95 percent
3	70 to 85 percent
2	50 to 70 percent
1	Below 50 percent

SCORING GUIDANCE:
Typically 1 if wealth is not accumulating in vessels, permits, or quota. Again, no need to spend time acquiring data on the price of every piece of gear required to participate in the fishery. Try to capture at least 80% of the harvest asset value required for access; for example, if participation requires a permit, vessel, and net, but data on net prices are hard to obtain, just focus on permit and vessel values. Even fisheries that are not managed with quota or permits will still require assets in order to access (vessels, traps, etc…). Use data on purchase value and not lease value of assets. In fisheries where there is no historic data on asset values, try to get participants to estimate general trends about whether they have been rising or falling and by how much. If data is available, please report in excel spreadsheet's 'Historical Data' tab and fill in data for past 10 years.

EXAMPLES:
See previous metric for a numerical example of how to calculate such a ratio from 10 years of historic data.

- Because data was unavailable for the West Coast groundfish fishery, the scorer guessed that asset values were up, but almost surely at least 85% of the historic high, due to the recent establishment of an ITQ system that led to an additional quota asset. The fishery scored a 5 with a quality score of B.
- In Liberia, there was also a lack of data on boat prices but a sense that they had been trending upwards as wood became scarcer so this fishery also scored a 5.

- In the Gambian oyster fishery there was no data on asset values, but there was a sense that wealth was not accumulating due to open access so it was scored a 1.

HARVEST SECTOR PERFORMANCE

HARVEST ASSET PERFORMANCE

Borrowing Rate Compared to Risk-Free Rate

RATIONALE:
The size of the premium the capital market demands to make loans in the fishery is a direct measure of financial risk in the industry. It is locally normalized to reflect the overall riskiness in the region and the opportunities available to local capital.

METRIC:
Average ratio between the interest rate on loans made to harvesters in the industry to risk-free rates over the last three years.

5	Ratio less than 1.75; cf. 30-year conforming mortgage
4	Ratio less than 2.5; cf. personal bank loan
3	Ratio less than 4; cf. good credit card rates
2	Ratio less than 7; cf. bad credit card rates
1	Ratio greater than 7; usury

SCORING GUIDANCE:
Calculation: Interest rate in the harvesting industry / Risk-free interest rate (average over last 3 years).

Note that if harvesting businesses can access international credit markets, then the international risk-free rate (US 10 year Treasury bill) is an appropriate comparison, but if businesses are forced to use local credit markets then the benchmark should be local risk-free rates (non-exporting Mexican shellfish harvesters use the Mexican bond, for example). Typically national/municipal government bonds will be the best representative of local risk-free rates. When scoring, it is often easier to ask the next question about the source of capital funds first and then ask about the rates that they pay. As long as there are credit transactions in the fishery this metric should not be NA; strive to get some estimate of the interest rates that harvesters pay.

In some fisheries, there are cultural or religious prohibitions on interest-based lending. If capital is paid out of cash flow, this can be NA. If proxy metric s are used to capture time value of capital, develop a best guess for the metric.

EXAMPLES:

- Alaska crab fishermen are able to take out loans from local banks to buy boats and they pay 9% interest; these harvesters have access to international credit markets and the 10 year US Treasury Bill rate in 2013 is 2.8% so the ratio is 9/2.8=3.2 and the score is a 3.
- Suppose that fishermen in an artisanal fishery take loans from a local microfinance organization and it is difficult to get precise data on the rates that they pay but anecdotal evidence suggests that it is more than 5 times the rate on national treasury bonds so the score is a 2 with a quality score of B.

- Some governments subsidize boat loans, making these interest rates artificially low; the subsidized loan rate should be scored as is. For example, if the government in the example above decided to replace the local microfinance corporation with a national loan program that offered loans for less than 2 times the rate on national treasury bonds then the score would be a 4 and the fishery would not be penalized with a lower score just because this is not the interest rate provided by the free market.

HARVEST SECTOR PERFORMANCE

HARVEST ASSET PERFORMANCE

Source of Capital

RATIONALE:
Whether lending capital from a particular source is even available is a direct measure of how the capital market assesses risk in the fishery. If a certain type of lender or investor is not willing to make capital available in the fishery at any price, it reveals the fishery is much riskier than other available investments.

METRIC:
Points to be assigned based on the category of lenders or investors that are most typically used by harvesters in the fishery. Second scoring method offered (after the semi-colon) if the supply chain (e.g., traders, processors, exporters) are the primary source of capital.

5	Unsecured business loans from banks/Venture capital;
4	Secured business loans from banks/Public stock offering; investment from elsewhere in the supply chain
3	Loans from banks secured by personal (not business) assets/Government subsidized private lending/Government-run loan programs/International aid agencies; secured loans from elsewhere in supply chain
2	Microlending/Family/Community-based lending/ Harvester association lending group; loans from supply chain that significantly reduce margins
1	Mafia/No capital available; exploitative relationship from elsewhere in supply chain

SCORING GUIDANCE:
This metric is less refined than the relative rate, but much easier to obtain.
Please note in the worksheet explanation which the scoring method used; i.e. whether or not the supply chain is the primary source of capital

EXAMPLES:
- In a Lake fishery in Malawi, harvesters finance their operations by borrowing from their family members who are farmers so this scores a 2.
- In the Florida spiny lobster fishery, fishermen discussed not being able to use trap certificates as collateral and in general new entrants not being able to get loans from banks, etc. They did obtain informal loans from family and friends which makes this score a 2 also.
- In the artisanal fishery in Ghana harvesters primarily obtain the funds to purchase fuel and nets from processors in exchange for lower ex-vessel prices later on; this is also a 2 because these types of loans significantly reduce margins but are not exploitative in the opinion of harvesters.
- The only fishery currently in the database that has scored a 5 is the Icelandic lobster.
- In Alaska pollock harvesters can use their quota/permit as collateral with banks so the score is 4.

- In Bangladesh, there is no borrowing due to Muslim culture so the score was a 1.
- In Peruvian anchovies, the artisanal and industrial sectors were being scored simultaneously and the artisanal mainly borrow from family while the industrial get secured business loans from banks so these two were averaged for a final score of 3.
- If the government is subsidizing boat loans, as in the previous metric's example then the fishery should score a 3.

HARVEST SECTOR PERFORMANCE

HARVEST ASSET PERFORMANCE

Functionality of Harvest Capital

RATIONALE:

The functionality of the vessels and other capital used in harvesting (e.g., weirs, traps, docks/marinas, and ice production) reflects wealth in several ways. First, it is a direct measure of wealth that has been accumulated from the fishery and reinvested in capital. Second, it is a measure of the potential wealth in the fishery, as newer facilities will be more efficient and less costly to operate. Third, if harvesters are willing to invest in new capital, it reflects their assessment that the fishery will be profitable into the future. Finally, if new facilities are funded by private loans, newer facilities reflect the capital markets' assessment that the fishery is sufficiently low risk to warrant investment.

METRIC:

Average age of the key durable harvesting capital unit (vessels, weirs). Ages are not assigned to scores due to differences in expected useful life, but buildings and industrial vessels have expected life of roughly 20 years.

5	Capital is new
4	Capital is older but well maintained, e.g., freshly painted
3	Capital is moderately well maintained
2	Maintenance is poor
1	Serious concerns about seaworthiness or safety throughout fishery

SCORING GUIDANCE:

This semi-industrial boat in Liberia is 10 years old but it is well-maintained so it scores a 4.

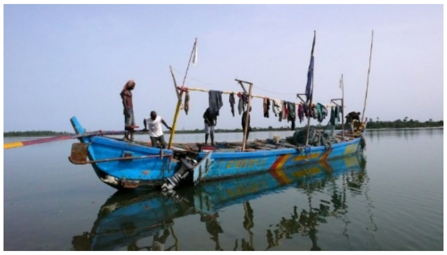

On the other hand, this dugout canoe in Liberia is old and not well-maintained, so it scores a 2.

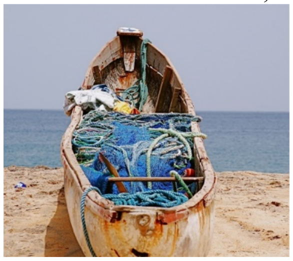

This industrial boat in Mexico is also 10 years old but well-maintained and also scores a 4.

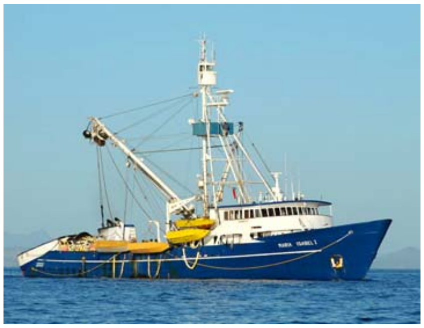

This industrial boat in Mexico is also older and not freshly painted or well-maintained, so it scores a 2.

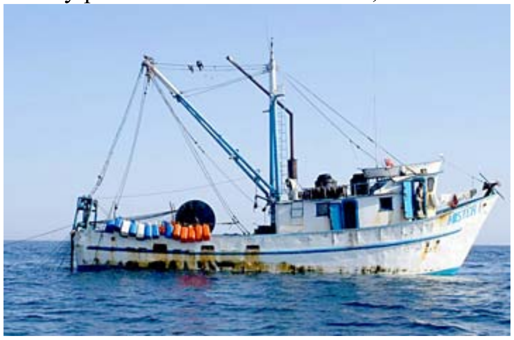

HARVEST SECTOR PERFORMANCE

RISK

Annual Total Revenue Volatility

RATIONALE:
Annual total revenue volatility is primarily a measure of the riskiness of the fishery. When future harvests are variable, it is difficult to make investment decisions and secure capital because future income streams are highly uncertain. High landings volatility also presents an obstacle to developing final product markets in non-specialty fisheries, as large processors and exporters prefer to deal with products for which they can develop long-term contracts.

METRIC:
Ratio of the standard deviation of the first differences of annual total revenue to the mean of total revenue over the last 10 years. Best guess may be calculated based on shorter time series if data not available.

5	Less than 0.15
4	0.15-0.22
3	0.22-0.40
2	0.40-1
1	Greater than 1

SCORING GUIDANCE:
See Volatility Worksheet (final tab in FPI scores worksheet) for assistance with calculations. Make note of the years used for the calculation in the worksheet and make sure to assess whether the data from a shorter time-series is reasonable/representative. Note that this metric is de-trended, so scores cannot be determined by simply looking at trends.

EXAMPLES:
- The global whitefish market is minimally volatile and total revenue does not fluctuate very much. Alaska Pollock scored a 4 using the above calculation.
- Conversely, the Japanese and Colombian shrimp fisheries are much more volatile and prices tend to vary widely across seasons so these fisheries both scored a 2.
- The Alaska salmon fishery is also very volatile as total revenue is highly dependent on the timing and size of the annual salmon run; this fishery also scored a 2.

HARVEST SECTOR PERFORMANCE

RISK

Annual Landings Volatility

RATIONALE:

Annual landings volatility is primarily a measure of the riskiness of the fishery. When future harvests are variable, it is difficult to make investment decisions and secure capital because future income streams are highly uncertain. High landings volatility also presents an obstacle to developing final product markets in non-specialty fisheries, as large processors and exporters prefer to deal with products for which they can develop long-term contracts.

METRIC:

Ratio of the standard deviation of the first differences of annual total landings to the mean of total landings over the last 10 years. Best guess may be calculated based on shorter time series if data not available.

5	Less than 0.15
4	0.15-0.22
3	0.22-0.40
2	0.40-1
1	Greater than 1

SCORING GUIDANCE:

See Volatility Worksheet (final tab in FPI scores worksheet) for assistance with calculations. Make note of the years used for the calculation in the worksheet and make sure to assess whether the data from a shorter time-series is reasonable/representative. Note that this metric is de-trended so scores cannot be determined by simply looking at trends. Need to get an estimate of landings over time and fill in a volatility worksheet. If precise historical data on landings is not available, ask for an estimate of whether they were higher or lower last year, then ask if they were 10% different, 20% different, etc… This should be able to be done for at least the past 3 years.

EXAMPLES:

Some species are biologically or environmentally prone to volatile landings as their growth is highly dependent on weather/natural disaster/other variations. Pelagic species with wide migrations may also show high volatility.

- Some fisheries that scored a 5: Australia Gulf Prawn, Icelandic Lobster, Philippines Blue Crab
- 4: Gambian oysters, New Zealand Hoki, Norwegian Cod, Alaska pollock
- 3: Alaska salmon, Dungeness Crab, Bangladesh artisanal floodplain
- 2: Gambia sole, Colombia shrimp, Seychelles sea cucumber, Senegal artisanal demersal
- 1: Liberia artisanal demersal

Harvest Sector Performance

Risk

Intra-annual Landings Volatility

Rationale:

High frequency (weekly or monthly, as available) landings volatility is primarily a measure of the potential for wealth generation in the fishery. High volatility may reflect a seasonality of the availability of the fish for harvest, or management that limits the harvest season directly, or induces a derby. Spikes in landings during certain parts of the year hinder wealth creation in several ways. First, concentrating landings in a short period spikes supply and often suppresses ex-vessel prices. Second, processing capacity must be established to handle the spikes, and if it is not applied to other fisheries, it will be underutilized and costly per unit processed. Third, spikes in processing volume often compromise the yield and quality of the processed product. Finally, intra-annual volatility can make it difficult for processors to make forward contracts for their products; thus they receive lower prices.

Metric:

Ratio of the standard deviation of the weekly/monthly total landings over the last three years to the mean of total weekly/monthly landings. Observations of zero landings are included if there is biological availability. Best guess may be calculated based on shorter time series if data not available (i.e. if detailed data is only available for the past year this is fine).

5	Less than 0.15
4	0.15-0.22
3	0.22-0.40
2	0.40-1
1	Greater than 1

Scoring Guidance:

See Volatility Worksheet (final tab in FPI scores worksheet) for assistance with calculations. If precise historical data on landings is not available, ask for an estimate of by what percentage they fluctuate each month. Make note of the years used for the calculation in the worksheet and make sure to check to see if the data from a shorter time-series is reasonable/representative. If the biological season is so short that there is not meaningful variation at the monthly level, this metric can be NA.

Examples:

Some species are biologically or environmentally prone to volatile intra-annual landings as in the Alaska salmon fishery where migratory species return to the rivers to spawn and the size of the salmon run varies widely from week to week. Although there are salmon available, there is uncertainty regarding how much will be available in any given week. In contrast, whitefish are often available in the same quantity throughout the season.

- Some fisheries that scored a 5: Philippines Blue Crab
- 4: Uganda Lake Victoria (perch, tilapia, and dagaa), Alaska Pollock, Norwegian cod
- 3: Malawi Lake Chiuta, Colombia shrimp

- 2: Liberia artisanal and semi-industrial, Icelandic lobster, Louisiana shrimp
- 1: Liberia artisanal demersal, Alaska salmon, Oregon Dungeness crab

HARVEST SECTOR PERFORMANCE

RISK

Annual Price Volatility

RATIONALE:

Annual price volatility complements annual harvest volatility to capture the wealth generation potential in the fishery. When future revenues are variable, it is difficult to make investment decisions and secure capital because future income streams are highly uncertain. High price volatility may reflect obstacles to developing final product markets in non-specialty fisheries, as large processors and exporters prefer to deal with products for which they can develop long-term contracts.

METRIC:

Ratio of the standard deviation of the first differences of annual ex-vessel prices to the mean of ex-vessel price over the last 10 years. Best guess may be calculated based on shorter time series if data not available

5	Less than 0.13
4	0.13-0.20
3	0.20-0.30
2	0.30-0.85
1	Greater than 0.85

SCORING GUIDANCE:

See Volatility Worksheet (final tab in FPI scores worksheet) for assistance with calculations. Make note of the years used for the calculation in the worksheet and make sure to assess whether data from a shorter time-series is reasonable/representative. Note that this metric is de-trended so scores cannot be determined by simply looking at trends. Need to get an estimate of prices over time and fill in a volatility worksheet. Price data may not be available for vertically integrated fisheries where price is not determined for transfers within a company. If ex-vessel prices are not available then wholesale prices should be used. If precise historical data on prices is not available, ask for an estimate of whether they were higher or lower last year, then ask if they were 10% different, 20% different, etc… This should be able to be done for at least the past 3 years.

EXAMPLES:

Some fisheries are prone to volatile prices as the demand for their product fluctuates. In general, the demand for whitefish is relatively stable while the demand for luxury products such as crab and lobster is more likely to be affected by economic conditions.

- Some fisheries that scored a 5: US Pacific groundfish, Norwegian cod, Bangladesh artisanal
- 4: Baltic cod, Alaska Pollock, Colombian shrimp
- 3: Alaska halibut, Louisiana shrimp
- 2: Alaska salmon, Florida spiny lobster, Peruvian anchovy
- 1: Japanese Suruga shrimp

HARVEST SECTOR PERFORMANCE

RISK

Intra-annual Price Volatility

RATIONALE:
Intra-annual price volatility complements intra-annual harvest volatility to capture the wealth generation potential in the fishery. Price changes arise from: 1) shifts in demand stemming from seasonal changes in tastes (e.g., traditional holiday fish dishes) or 2) changes in supply stemming from the seasonal availability of fish or management-induced periods of high effort. If price volatility is high, unconstrained harvesters could shift landings from a period of low price to a period of higher price and increase fishery rent. Periods of high landings at low prices are associated with fishing derbies and the problems associated with high intra-annual landings volatility.

METRIC:
Ratio of the standard deviation of average monthly ex-vessel prices over the last three years to the mean ex-vessel price. Observations of zero landings are included if there is biological availability. Best guess may be calculated based on shorter time series if data not available.

5	Less than 0.13
4	0.13-0.20
3	0.20-0.30
2	0.30-0.85
1	Greater than 0.85

SCORING GUIDANCE:
See Volatility Worksheet (final tab in FPI scores worksheet) for assistance with calculations. If precise historical data on landings is not available, ask for an estimate of by what percentage they fluctuate each month. Make note of the years used for the calculation in the worksheet and make sure to assess whether data from a shorter time-series is reasonable/representative. Price data may not be available for vertically integrated fisheries where price is not determined for transfers within a company. If ex-vessel prices are not available then wholesale prices should be used. Want to capture the extent to which prices vary over an entire season so daily/weekly observations can be averaged to larger periods so that there are 10-20 observations during each season. If the biological season is so short that there is not meaningful variation at the monthly level, this metric can be NA. The prices used should be within-season when there is fish available and landings are not zero; this is because the metric aims to capture the level of uncertainty that inhibits the ability of harvesters to flexibly change their landings within the season.

EXAMPLES:
Some fisheries are prone to volatile intra-annual prices due to shifts in demand or restricted supply.

- Some fisheries that scored a 5: US New England groundfish, US California Sea Urchin
- 4: US Pacific groundfish, Alaska Pollock, Norwegian cod
- 3: Japanese Suruga shrimp, Florida spiny lobster, Louisiana shrimp
- 2: Senegal artisanal demersal, Peruvian anchovy

HARVEST SECTOR PERFORMANCE

RISK

Spatial Price Volatility

RATIONALE:
The extent to which ex-vessel price for the same product varies across different ports within the fishery reflects market integration and opportunities for arbitrage across space within the fishery. A market that is well integrated spatially will have similar prices at different ports, whereas isolated landings ports or ports that are differentially well connected to markets, and therefore posing greater financial risk, will have higher levels of spatial volatility.

METRIC:
Ratio of the standard deviation across data collection regions[9] of average annual ex-vessel price to the mean of ex-vessel price across data collection regions. Metric should be averaged over last three years. Best guess may be calculated based on shorter time series if data not available.

5	Less than 0.13
4	0.13-0.20
3	0.20-0.30
2	0.30-0.85
1	Greater than 0.85

SCORING GUIDANCE:
See Volatility Worksheet (final tab in FPI scores worksheet) for assistance with calculations. If precise historical data on prices is not available, ask for an estimate of by what percentage they fluctuate each year. Make note of the years used for the calculation in the worksheet and make sure to check to see if the data from a shorter time-series is reasonable/representative. If landing sites are not selling into distinct markets then this metric may be NA. Price data may not be available for vertically integrated fisheries where price is not determined for transfers within a company. If ex-vessel prices are not available then wholesale prices should be used. Data collection regions may include landing sites that are not currently feasible for the typical harvester because this metric is meant to capture opportunities for further market integration.

EXAMPLES:
Some fisheries are prone to spatial volatility due to a lack of infrastructure or trade barriers prohibiting market integration.

- Some fisheries that scored a 5: Australia Gulf Prawn, Colombian Shrimp, Alaskan crab and halibut
- 4: Alaska Pollock, New England groundfish, Peruvian anchovy
- 3: Philippines Blue Crab
- 2: Norwegian cod, Uganda Nile perch and tilapia, Alaska salmon

---

[9] Data collection regions can either be fishery relevant or politically relevant. Defining this generally allows local standards to establish which is most important.

HARVEST SECTOR PERFORMANCE

RISK

Contestability and Legal Challenges

RATIONALE:

Legal challenges, protests, and contentious public hearings reflect discontent with the management system. It is an indicator of a lack of social acceptance and a source of considerable risk. This diminishes the welfare that is accruing to the community if the fishery is a source of discord.

METRIC:

This captures the degree to which political activity limits the ability to implement effective fishing regulations.

5	No significant legal challenges, civil actions, or protests regarding the fishery management system
4	Minor legal challenges slow implementation
3	Legal challenges, civil actions, or protests impede some management measures
2	Legal challenges, civil actions, or protests suspend major elements of the management system
1	Legal challenges, civil actions, or protests suspend or prohibit implementation of key management reforms and regulation

EXAMPLES:

Some fisheries are prone to contestability due to cultural norms and institutions while in others contestability develops as a response to ineffective management.

- Some fisheries that scored a 5: Historically these fisheries have not seen any protests (Maldives skipjack tuna, Japanese Suruga shrimp).
- 4: There have been lawsuits and non-violent protests that have made passing ITQ systems slower (Icelandic lobster, Alaska crab)
- 3: Sometimes protests erupt over efforts to change the open access nature of the fisheries (Liberia artisanal, Colombian industrial shrimp).
- 2: The legal system is unable to enforce rulings and penalties imposed by the authorities on fishing companies (Peruvian anchovy, New England groundfish).
- 1: Consistent protests and laws are in place to ensure that the managing authority cannot limit access (Ghana artisanal).

HARVEST SECTOR PERFORMANCE

OWNERS, PERMIT HOLDERS & CAPTAINS

Earnings Compared to Regional Average Earnings

RATIONALE:
This is a direct measure of the type of agents who are attracted to this fishery and become the owners of harvesting capital. Scaling earnings by regional average earnings reflects whether the fishery is able to attract the most talented workers in the community and is doing well at wealth generation relative to regional standards.

METRIC:
Ratio of annual earnings per owner to the average earnings in the region. In many cases, the captain is an owner of a vessel or permit, but in other cases, captains are considered as crew. The owners are defined as those holding the ability to access, including rights and capital.

5	More than 50% above the regional average
4	Between 10 and 50% above regional average
3	Within 10% of the regional average
2	Between 50% and 90% of the regional average
1	Less than half of the regional average

SCORING GUIDANCE:
This is meant to measure what type of people this fishery attracts so we want to count all their income for an entire year from any sources. These earnings should be compared to regional/national levels depending on the economic sphere of the captains. Economic sphere is defined as the region where the owners conduct the majority of their economic activity, i.e. village if all economic activity is within the village, but nation if participates in national markets as a consumer.

This is a tough metric in seasonal or part-time fisheries where most harvesters also have other jobs that provide some, or even primary, income but an effort should be made to estimate the earnings of the typical owner from all sources. Make sure that this variable and the following metrics in the owner category are scored for the people who hold the ability to access; this would only be boat owners if there is no quota/permit system but it would also include permit/quota owners in a system where those regulations are in place. In fisheries where most captains do not own vessels or permits, their outcomes should be averaged in with the crew metrics.

EXAMPLES:

- In the Alaska Pollock fishery, captains are hired contractors that fish quota held by companies. Since they serve more as executives overseeing a large crew of skilled and semi-skilled laborers (who are scored in the crew metric), these hired captains are considered in this metric, along with the management of the companies that employ them.
- In the Alaska crab fishery, most captains of vessels are quota holders. Typically, these captains receive income primarily from fishing, live in Seattle, and do not take other jobs

in the off season. In this case, the ratio would be their annual income from crab fishing / average annual incomes in the US (assume this is $100,000/$50,000 which is 2 and a score of 5).

- In the New England groundfish fishery, captains make half their income from the groundfish fishery and half from other, separately regulated fisheries; they tend to be locals from New England but participate in national markets. In this case, the ratio would be their annual income from groundfish and scallops divided by the New England average annual income (assume this is $35,000/$50,000 which is 0.7 and the score is 2).
- In the Kenyan octopus fishery, most boat captains also spend part of the year farming or doing snorkeling trips for tourists. They do not usually travel outside their home village. In this case the ratio is their income from all three sources divided by average incomes in the village (assume this is $1800/$1200 which is 1.5 and the score is 4).
- On Lake Victoria, there is no data on captain's earnings or typical household incomes. However, it is clear that captains earn at least a little more than other households in their villages because they live in more expensive homes and are able to afford vehicles. This was scored a 4 with a quality score of B since the scorer was sure that the captains were above the regional average but was unsure by what percentage.

HARVEST SECTOR PERFORMANCE

OWNERS, PERMIT HOLDERS & CAPTAINS

Owner/Permit Holder/Captain Wages Compared to Non-fishery Wages

RATIONALE:
This is a direct measure of fishery-produced wealth accumulating to harvesters. Scaling wages by average local earnings in relevant alternatives reflects whether the fishery is able to attract the most talented workers in the community and is doing well at wealth generation relative to local standards.

METRIC:
Ratio of captain's average daily wage in this fishery to average daily wage in the captain's alternative occupations within their economic sphere (e.g., jobs in the village that the captain qualifies for if all economic activity is within the village, but if labor markets are fluid then this should be national average wages in jobs that the captain expects to be able to obtain).

5	More than 50% above the alternative wage
4	Between 10 and 50% above alternative wage
3	Within 10% of the alternative wage
2	Between 50% and 90% of the alternative wage
1	Less than half the alternative wage

SCORING GUIDANCE:
This is meant to measure the average personal opportunity cost of participating in this fishery, thus the alternative wage should be the answer to the question of "If you couldn't fish in this fishery, how much would you get paid?" Look at the average daily wage for captains (or harvest capital owners, see above metric) when participating in the fishery and then compare it to the wage in their next best alternative. If the captains think that without the fishery they would be construction workers, then score based on construction worker wages, but if they think that they would be subsistence farming, then compare it to that wage. Again, this should all be relative to wages within their economic sphere so consider national wages if labor markets are fluid, but restrict the comparison to wages within the village/region if captains would not leave their local community to find work.

EXAMPLES:

- Suppose that in a theoretical fishery, most permit holders believe that the next best alternative they are qualified for is working a construction job that earns $200/day (a national average) but as permit holders they make $260/day so the ratio is 260/200=1.3 and the score would be a 4.
- In Bangladesh, captains know that if they could not fish then they would be farmers within the same community, and they estimate that they would earn more than 50% less than they currently do so the score is 5.

HARVEST SECTOR PERFORMANCE
OWNERS, PERMIT HOLDERS & CAPTAINS

Education Access

RATIONALE:
A community that is successfully using its resources will be able to provide high levels of education to its children, ensuring a step beyond resource dependence in the next generation. If capture fishing is an important part of this community, the boat owners or captains' families will have access to education.

METRIC:
This metric is based on the highest level of education that is politically and financially accessible to families of harvesters, rather than the actual attainment levels of current harvesters. The level of education accessible to (available and affordable) the families (i.e., children) of permit holders and captains.

5	Higher education is accessible
4	High school level education or advanced technical training is accessible
3	Middle school level education or simple technical training is accessible
2	Basic literacy and arithmetic training is accessible
1	Formal education is not accessible

SCORING GUIDANCE:
This metric is meant to capture whether captains can afford to provide education for their children. Just because there is a high school in the village does not mean that captains can afford to send their children – consider school fees, tuition, and opportunity cost. Note that learning to fish as an on the job apprenticeship does not count as formal technical training. Advanced technical training involves science/technology and most apprenticeships in LDCs do not count and should be classified as simple technical training at the most.

EXAMPLES:
- In Uganda, children of captains seldom go to school beyond primary levels because they are needed to work in the fields or on the boats and the score is a 2.
- In the Alaska Pollock fishery, most captains are not college educated but they make high enough incomes to send their children to globally competitive universities so the score is a 5.
- In some rural fisheries, captains make enough money to afford university tuition, but the university is far away and they cannot afford to pay for the transport and housing so most of their children only attend high school in the local village and do not go to university; this is a 4.
- In Mexico, there is a shellfish fishery where the cultural barriers to attending school beyond junior high are very strong; although the high schools are cheap and the harvesters can afford it they do not value education and it is not the norm in their community. The children of permit holders seldom attend high school, but this fishery

scored a 4 because the captains could afford to send their children to the local high school (not the local university) even though they choose not to enroll.

HARVEST SECTOR PERFORMANCE

OWNERS, PERMIT HOLDERS & CAPTAINS

Access to Health Care

RATIONALE:
A community that is successfully using its resources will be able to provide high levels of health care, ensuring a quality of life and decreasing health risk. If capture fishing is an important part of this community, harvester's families will have access to the best available health care.

METRIC:
This metric is based on the quality of health care that is politically and financially accessible to harvesters. The level of health care accessible to (available and affordable) the owners/permit holders/captains and their families.

5	Global standard treatment for illness is accessible
4	Licensed doctors provide trauma, surgical and drug treatments
3	Nurses or medical practitioners provide emergency and routine drug treatments
2	Basic and simple drug treatment is accessible
1	Medical or drug treatment is not accessible

SCORING GUIDANCE:
This metric is meant to capture whether captains can afford to provide health care for themselves and their families. Just because there is a clinic in the village does not mean that captains can afford it – consider medical fees, travel time and opportunity cost. The WHO ranking of health care quality is used as a basis for identifying whether local care facilities are capable of providing global standard care (http://www.who.int/whr/2000/en/whr00_en.pdf). If there is a global-standard hospital located in a major city a day's journey away, then global standard treatment for illness is not fully accessible because if there was a major trauma then the injured party would likely die before reaching medical assistance (unless there is a Coast Guard helicopter assigned to transport injured harvesters/family members). Score based on the health facilities that are used most frequently for routine procedures and somewhat urgent issues. Fisheries that have established protocol to care for harvesters in the event of emergencies should score slightly higher than those where there are no such measures in place.

EXAMPLES:

- In the Alaska crab fishery, most of the harvesters' families live in suburban communities in Seattle where any kind of health care is accessible. In addition there are designated safety measures in place to ensure that harvesters on crab boats get access to health care quickly in the event of an emergency even if they are at sea so the score is a 5.
- In rural Bangladesh, there is a small clinic with nurses equipped for emergencies but the sanitation is poor and there are no doctors, except in cities. Harvesters seldom travel to the hospital in the capitol because the cost of travel is prohibitive. This is a 3.
- In rural Mexico, the harvesters do not qualify for health insurance coverage so they cannot afford to go to the clinics and their families rely on drug treatments which is a 2.

HARVEST SECTOR PERFORMANCE

OWNERS, PERMIT HOLDERS & CAPTAINS

Social Standing of Boat Owners and Permit Holders

RATIONALE:
This is a proxy for income associated with boat and permit ownership, which may be much easier to collect than actual income information. It also allows informal incorporation of part-time harvesting jobs into other careers. Social standing reflects whether the fishery is able to attract the most talented workers in the community and is doing well at wealth generation relative to local standards.

METRIC:
This metric is based on the social standing of owners/permit holders/captains within the community where they spend the majority of their time.

5	Among the most respected in the community, comparable with civic and religious leaders and professionals, such as doctors and lawyers
4	Comparable to management and white collar jobs
3	Comparable to skilled labor jobs
2	Comparable to unskilled blue collar or service jobs
1	Among the least respected, such as slaves or indentured servants

SCORING GUIDANCE:
This is meant to reflect the amount of esteem that harvesters receive in their local community. Make sure that answers for this metric make sense relative to the social status of the other fishery occupations (crew, processing managers, and processing workers). Consider their social status within their primary community. The comparison group should be the region where captains spend the majority of their time and income.

EXAMPLES:

- Captains in the Alaska salmon fishery are held in very high esteem in the small rural Alaskan villages where they land their fish, but many live in Seattle where they are seen as comparable to skilled labor jobs so the score is a 3.
- Captains in Ghana do not leave the village and often find themselves in positions of leadership within the community so the score is a 5.

Harvest Sector Performance

Owners, Permit Holders & Captains

Proportion of Nonresident Employment

Rationale:

The ability of a country or region to improve itself using its resources depends on its ability to maintain local economic multipliers by keeping resource-based earnings within the region. A large portion of nonresident harvesters reflects that much of the harvesting wealth will be leaving the region, failing to boost the regional economy. In developing regions, it may also reflect an inability of local resource users to generate sufficient capital to harvest.

Metric:

Proportion of captains/permit holders who are local. "Local" is defined as coming from, and spending their earnings within, the local fishing community. Nationals who are transient nonresidents, or considered outsiders in the fishing community, are not local.

5	95-100% local
4	70-95% local
3	35-70% local
2	5-35% local
1	Virtually no local owners/captains/permit holders

Scoring Guidance and Examples:

- This may be difficult to score in rural fisheries where the majority of the population are not residents year-round. For example, in the Alaska salmon fishery the majority of landings occur in rural villages where the population is highly seasonal. In this case, local should be defined as coming from the state of Alaska and not restricted to residents of rural Alaska.
- Foreign nationals who are not technically citizens but who live, raise their families, and spend their money in the region should be considered local. For example, in Liberia there is a large population of Ghanaians who have lived in Liberia for generations and do not use their earnings to support people in other regions so they are considered local.

HARVEST SECTOR PERFORMANCE

CREW

Earnings Compared to Regional Average Earnings

RATIONALE:
This is a direct measure of the type of agents who are attracted to this fishery and become the harvesting crew. Scaling earnings by average regional earnings reflects whether the fishery is able to attract the most talented workers in the community and is doing well at wealth generation relative to local standards.

METRIC:
Ratio of annual earnings per crew member to the regional average earnings. In many cases, the captain is an owner of a vessel or permit, but in other cases, captains are considered as crew. Crew is defined as those depending on others for access.

5	More than 50% above the regional average
4	Between 10 and 50% above regional average
3	Within 10% of the regional average
2	Between 50% and 90% of the regional average
1	Less than half of the regional average

SCORING GUIDANCE:
This is meant to measure what type of people this fishery attracts so all their income for an entire year from any source is included. These earnings should be compared to regional/national levels depending on the economic sphere of the crew. Economic sphere is defined as the region where the crew conduct the majority of their economic activity, i.e. village if all economic activity is within the village, but nation if participate in national markets as a consumer. This is a tough metric in seasonal or part-time fisheries where most harvesters also have other jobs that provide some, or even primary, income but an effort should be made to estimate the earnings of the typical crew from all sources. Make sure that this variable and the following metrics in the crew category are scored for the people who depend on others for the ability to access; this would could include moderately skilled boat captains if the vessels are owned by companies who contract temporary captains. If crew are typically paid on a share system, rather than salary, it can be estimated based on landings, prices, and the share scheme. Make sure that the answers to the crew metrics make sense relative to the captains/owners.

EXAMPLES:

- In the Alaska crab fishery, most crew typically work construction jobs in the off season and live in Washington State. In this case, the ratio would be their annual income from crab fishing and construction / average annual incomes in the US (assume this is $70,000/$50,000 which is 1.4 and a score of 4).
- Suppose that in an artisanal fishery, most crew do not travel outside their home village. In this case the ratio is their income from all sources divided by average incomes in the village (assume this is $1300/$1200 which is 1.08 and the score is 3).

- On Lake Victoria, there is no data on crew earnings or typical household incomes. However, it is clear that crew earn approximately the same amount as other households in their villages because they live in the same areas and can afford similar homes. This was scored a 3 with a quality score of B since the scorer was sure that the crew were within 50% of the regional average.

HARVEST SECTOR PERFORMANCE

CREW

Crew Wages Compared to Non-fishery Wages

RATIONALE:
Crew wage is a direct measure of the fishery wealth that accumulates to crew. It is normalized by wages typical of the available alternatives to provide a relative standard of living afforded to crew, and also reflect whether the fishery is able to attract the most skilled workers.

METRIC:
Ratio of crew's average daily wage in this fishery to average daily wage in the crew's alternative occupations within their economic sphere (e.g., jobs in the village that the crew qualify for if all economic activity is within the village, but if labor markets are fluid then this should be national average wages in jobs that the crew expect to be able to obtain).

5	More than 50% above the alternative wage
4	Between 10 and 50% above alternative wage
3	Within 10% of the alternative wage
2	Between 50% and 90% of the alternative wage
1	Less than half the alternative wage

SCORING GUIDANCE:
This is meant to measure the average personal opportunity cost of participating in this fishery, thus the alternative wage should be the answer to the question of "If you couldn't fish how much would you get paid?" Look at the average daily wage for crew members (this includes contract captains, see above metric) when participating in the fishery and then compare it to the wage in their next best alternative. If the crew think that without the fishery they would be construction workers then look up construction worker wages, but if they think that they could be subsistence farming then compare it to that wage. Again, this should all be relative to wages within their economic sphere so consider national wages if labor markets are fluid, but restrict the comparison to wages within the village/region if crew seldom leave their local community and do not have the means to do so.

EXAMPLES:

- In the Alaska crab fishery, most crew believe that the next best alternative they are qualified for is working a construction job that earns $200/day (a national average) so the ratio is 225/200=1.13 and the score is 4. In this instance, they face the same alternatives as the captains/owners because they have equal educational attainment.
- In Bangladesh, crew know that if they could not fish then they would be farmers and they estimate that they would earn more than 50% less than they currently do so the score is 5.

HARVEST SECTOR PERFORMANCE

CREW

Education Access

RATIONALE:

A community that is successfully using its resources will be able to provide high levels of education to its children, ensuring a step beyond resource dependence in the next generation. If capture fishing is an important part of this community, harvester's families will have access to education.

METRIC:

This metric is based on the highest level of education that is politically and financially accessible to families of harvesters, rather than the actual attainment levels of current harvesters. The level of education accessible to (available and affordable) the families (e.g., children) of crew.

5	Higher education is accessible
4	High school level education or advanced technical training is accessible
3	Middle school level education or simple technical training is accessible
2	Basic literacy and arithmetic training is accessible
1	Formal education is not accessible

SCORING GUIDANCE:

This metric is meant to capture whether crew can afford to provide education for their children. Just because there is a high school in the village does not mean that crew can afford to send their children – consider school fees, tuition, and opportunity cost. Note that learning to fish as an on the job apprenticeship does not count as formal technical training. Advanced technical training involves science/technology and most apprenticeships in LDCs do not count and should be classified as simple technical training at the most.

EXAMPLES:

- In Uganda, children of crew seldom go to school beyond primary levels because they are needed to work in the fields or on the boats and the score is a 2.
- In the Alaska Pollock fishery, most crew are not college educated but they make high enough incomes to send their children to university so the score is a 5.
- In some rural fisheries, crew make enough money to afford university tuition, but the university is far away and they cannot afford to pay for the transport and housing so most of their children only attend high school in the local village and do not go to university; this is a 4.

HARVEST SECTOR PERFORMANCE

CREW

Access to Health Care

RATIONALE:

A community that is successfully using its resources will be able to provide high levels of health care, ensuring a quality of life and decreasing health risk. If capture fishing is an important part of this community, harvester's families will have access to the best available health care.

METRIC:

This metric is based on the quality of health care that is politically and financially accessible to harvesters. The level of health care accessible to (available and affordable) the crew and families of crew.

5	Global standard treatment for illness is accessible
4	Licensed doctors provide trauma, surgical and drug treatments
3	Nurses or medical practitioners provide emergency and routine drug treatments
2	Basic and simple drug treatment is accessible
1	Medical or drug treatment is not accessible

SCORING GUIDANCE:

This metric is meant to capture whether crew can afford to provide health care for themselves and their families. Just because there is a clinic in the village does not mean that crew can afford it – consider medical fees, travel time and opportunity cost. The WHO ranking of health care quality is used as a basis for identifying whether local care facilities are capable of providing global standard care (http://www.who.int/whr/2000/en/whr00_en.pdf). If there is a hospital located in the main city 4 hours away then global standard treatment for illness is not accessible because if there was a major trauma then the injured party would likely die before reaching medical assistance (unless there is a Coast Guard helicopter assigned to transport injured harvesters/family members). Note that the scores for this metric are likely to be the same across crew and captains if they and their families live in the same communities and both make enough money to afford the health care that is offered there. It will be different if captains/owners can afford to travel to urban centers for emergency or surgical procedures while crew cannot.

EXAMPLES:

- In the Alaska crab fishery, most of the harvesters' families live in suburban communities where any kind of health care is accessible, in addition there are designated safety measures in place to ensure that harvesters on crab boats get access to health care quickly in the event of an emergency so the score is a 5.
- In rural Bangladesh, there is a small clinic with nurses equipped for emergencies but the sanitation is poor and there are no doctors. Harvesters seldom travel to the hospital in the capitol because the cost of travel is prohibitive. This is a 3.
- In rural Mexico, the harvesters do not qualify for health insurance coverage so they cannot afford to go to the clinics and their families rely on drug treatments which is a 2.

HARVEST SECTOR PERFORMANCE

CREW

Social Standing of Crew

RATIONALE:
This is a proxy for income associated with crewing on fishing boats, which may be much easier to collect than actual wage information. It also allows informal incorporation of part-time harvesting jobs into other careers. Social standing reflects whether the fishery is able to attract the most talented workers in the community and is doing well at wealth generation relative to local standards.

METRIC:

5	Among the most respected in the community, comparable with civic and religious leaders and professionals, such as doctors and lawyers
4	Comparable to management and white collar jobs
3	Comparable to skilled labor jobs
2	Comparable to unskilled blue collar or service jobs
1	Among the least respected, such as slaves or indentured servants

SCORING GUIDANCE:
This is meant to reflect the amount of esteem that crew receive in their local community. Make sure that answers for this metric make sense relative to the social status of the other fishery occupations (owners, processing managers, and processing workers). Consider their social status within their primary community. The comparison group should be the region where crew spend the majority of their time and income.

EXAMPLES:

- Crew in the Alaska salmon fishery are held in fairly high esteem in the small rural Alaskan villages where they land their fish, but they spend most of their time home in Seattle where they are seen as comparable to skilled labor jobs so the score is a 3.
- In the Liberian artisanal fishery, fishing is prized by the local community and crew are held in as high esteem as management/white collar jobs so the score is a 4.

HARVEST SECTOR PERFORMANCE

CREW

Proportion of Nonresident Employment

RATIONALE:
The ability of a country or region to improve itself using its resources depends on its ability to maintain local multipliers by keeping wealth within the region. A large portion of nonresident harvesters reflects that much of the harvesting wealth will be leaving the region, failing to boost the regional economy. In developing regions, it may also reflect an inability of local resource users to generate sufficient capital to harvest.

METRIC:
Proportion of crew who are local. "Local" is defined as coming from, and spending their earnings within, the local fishing community. Nationals who are transient nonresidents, or considered outsiders in the fishing community, are not local.

5	95-100% local
4	70-95% local
3	35-70% local
2	5-35% local
1	Virtually no local crew

SCORING GUIDANCE AND EXAMPLES:

- This may be difficult to score in rural fisheries where the majority of the population are not residents year-round. For example, in the Alaska salmon fishery the majority of landings occur in rural villages where the population is highly seasonal. In this case, local should be defined as coming from the state of Alaska and not restricted to residents of rural Alaska.
- Foreign nationals who are not technically citizens but who live, raise their families, and spend their money in the region should be considered local. For example, in Liberia there is a large population of Ghanaians who have lived in Liberia for generations and do not use their earnings to support people in other regions so they are considered local.

HARVEST SECTOR PERFORMANCE

CREW

Crew Experience

RATIONALE:

The rate at which the crew force turns over in the fishery is an indirect measure of several key variables. First, it reflects wealth accumulation to crew because a crew member will only stay in the fishery if the wage is comparable to, or better than, other jobs he could obtain. Second, crew longevity often means they are resident in the community, and thus their earnings stay in the community and are spent locally, rather than being sent away by itinerant or immigrant crews. Third, experienced crew develop specialized knowledge and refined skills that make harvesting more efficient, so the fishery is better able to reach its wealth-generating potential. Finally, many crew will stay in the fishery if they believe the future to be worthwhile and that they will have the means to succeed to captain.

METRIC:

Average years of experience of crew members.

5	More than 10 years (skilled career crew)
4	5-10 years
3	3-5 years
2	1-3 years
1	0 full years of experience (mostly new crew each season)

EXAMPLES:

- In the New England groundfish fishery the average years of experience are 5 so the fishery scores a 3.
- There is no precise data on crew age in the Mexican shellfish fishery, but harvesters are certain that the average harvester has more than 3 years of experience so the score is a 4 with a quality score of B.

HARVEST SECTOR PERFORMANCE

CREW

Age Structure of Harvesters

RATIONALE:

A widely distributed age structure is an indirect measure of several key variables. Broadly, it reflects both that experienced older crew is willing to stay in the fishery, possibly as captains, but also that younger crew members are willing to enter and that job opportunities in the fishery are available. First, it reflects wealth accumulation to crew because an experienced crew member will only stay in the fishery, and a new crew member will only enter, if the wage is comparable to, or better than, other jobs he could obtain. Second, crew longevity often means the crew are resident in the community, and thus their earnings stay in the community and are spent locally, rather than being sent away by itinerant or immigrant crews. Third, experienced crew develop specialized knowledge and refined skills that make harvesting more efficient, so the fishery is better able to reach its wealth-generating potential. Finally, many crew will only enter (young) or stay in (older) the fishery if they believe the future to be worthwhile and that they will have the means to succeed to captain.

METRIC:

Age range of both captains and their crews:

5	All working ages are well represented
4	Slightly skewed toward younger or older
3	Skewed toward younger or older
2	Almost entirely younger or older, but working age
1	Harvesters primarily younger or older than working age

EXAMPLES:

- In the Alaska salmon fishery there are young harvesters working alongside older ones and the average age is well-balanced so the fishery scored a 5.
- Conversely, in the Alaska crab fishery there was anecdotal evidence that the majority of harvesters are older with only a few young ones (greenhorns) brought on each year. This was given a 3 with a quality score of C since the scorer was uncertain whether the skew towards older harvesters that they observed was representative of the entire fishery.

HARVEST SECTOR PERFORMANCE

MARKET PERFORMANCE

Ex-vessel Price Compared to Historic High

RATIONALE:
If the fishery is generating wealth, it is expected that the orientation of the fishery will shift from competing for fishery resource access, to market access and development. This should be observable in stable or increasing ex-vessel prices.

METRIC:
The indicator is the ratio of current ex-vessel prices to the average of the three highest annual ex-vessel prices in the past 10 years. Adjust by local CPI if inflation was significant.

5	Above 95 percent
4	85 to 95 percent
3	70 to 85 percent
2	50 to 70 percent
1	Below 50 percent

SCORING GUIDANCE:
In fisheries where there is no historic data on prices, try to get participants to estimate general trends about whether prices have been rising or falling and by how much. If data is available, report in excel spreadsheet's 'Historical Data' tab and fill in data as below. Note that this is ex-vessel prices and not wholesale or post-processing prices.

EXAMPLE:
Suppose the following data comes from an artisanal fishery in Liberia:

Prices:		Average inflation rate: 6% (no need to adjust by local CPI)
2012	200 LRD/kg	Average of 3 highest: 225 LRD/kg
2011	198 LRD/kg	Ratio: 200 LRD/kg (current)/225 LRD/kg = 0.89
2010	180 LRD/kg	Score is a 4
2009	**210 LRD**/kg	
2008	**230 LRD**/kg	
2007	**235 LRD**/kg	
2006	200 LRD/kg	
2005	190 LRD/kg	
2004	120 LRD/kg/kg	
2003	100 LRD	

Post-Harvest Performance

Market Performance

Final Market Use

Rationale:
The use of the fishery product that is finally consumed reflects the extent to which the fishery, its processing and trade products are maximizing the potential value from the resource.

Metric:
Premium Products are typically distinct to species, or species and source. Where a supply chain is diverse, score each and weight by value.

5	Premium human consumption (premium quality and products)
4	High-value human consumption
3	Moderate-value human consumption
2	Low-value human consumption
1	Fish meal/animal feed/bait or non-consumptive

Scoring Guidance:
Ensure that for single species fisheries where there are multiple products (e.g. Alaska salmon is canned and sold fresh) each product is scored and weighted by value. In multispecies fisheries simplify and use the product form that dominates total value for each species and then weight each species by its contribution to total value across the fishery. This metric is meant to be scored relative to the global seafood trade. This is not meant to reflect relative product quality within a given species (i.e. quality of Japanese sardine relative to Chinese sardine). If we were scoring the Japanese sardine fishery then we should be scoring the product relative to the finest ahi tuna.

Examples:

- Fisheries that have received a 5: Australian Gulf Prawn, Alaska halibut, Alaska crab (high-value product marketed with specific source names)
- 4: Alaska salmon, New England groundfish, Lake Victoria Nile Perch (fillets)
- 3: Alaska Pollock, Louisiana shrimp, Philippines Blue Crab (surimi, fermented)
- 2: Senegal artisanal, Lake Victoria dagaa, Gambia oysters (smoked/dried)
- 1: Peruvian anchovy (animal feed)

POST-HARVEST PERFORMANCE

MARKET PERFORMANCE

International Trade

RATIONALE:

Maximizing the wealth generation potential of a fishery requires delivering the product to the people who value it most. The level of exports reflects how well the fishery has maximized its wealth potential by accessing the market that is willing to pay the most for the product globally. Although there are valid reasons why exporting might be less profitable, such as high local demand for specialized products, in general the greatest returns are from taking advantage of higher willingness to pay in international markets.

METRIC:

Percentage of the fishery's value that is from fish exported to higher value international markets (outside of the country of origin) for consumption:

5	90-100% export
4	60-90% export
3	30-60% export
2	2-30% export
1	Virtually no export

SCORING GUIDANCE:

When products are exported for processing and then reimported for consumption this still counts as export. Note the emphasis on export to higher value markets. This means that a Ghanaian fishery that distributes some smoked fish to Burkina Faso does not count as exports because this is not considered a higher value market.
In addition, regional trade within a country should not be counted as exports.

The calculation should be: Total value of exports/Total value of fishery
Total value is calculated based on wholesale prices and quantities.

POST-HARVEST PERFORMANCE

MARKET PERFORMANCE

Final Market Wealth

RATIONALE:

The income of the people who finally consume the fishery product reflects the extent to which the fishery, its processing, and trade products are maximizing the potential value from the resource. Products that are being sold in wealthier countries are competing favorably, reflecting high-quality, effective marketing, and are drawing wealth to the fishery. Bins are based on US CIA's rank of per capita GDP of all countries (https://www.cia.gov/library/publications/the-world-factbook/rankorder/2004rank.html).

METRIC:

Average per capita GDP of the consumer of a fishery's primary final product. If multiple important products, weight by value:

5	Greater than 35,000USD
4	Greater than 25,000USD
3	Greater than 12,500USD
2	Greater than 5,000USD
1	Less than 5,000USD

EXAMPLE:

Assume that 70% of Alaskan Pollock is converted into surimi and consumed by Americans, 20% is exported to France and sold as frozen filets, and the remaining 10% is roe that is consumed in Russia. The calculation should be .7*US GDP +.2*FR GDP+.1*RU GDP = .7*52,000+.2*40,000+.1*14,000>35,000 so the score is 5.

Obviously, an actual calculation could be more complicated with more countries importing Pollock and each country importing each of the three product forms. In that case, focus on the top 5 importers by volume and weight the GDP of each by the percent of Pollock export revenues that they take in.

POST-HARVEST PERFORMANCE

MARKET PERFORMANCE

Wholesale Price Compared to Similar Products

RATIONALE:
The extent to which a fishery is realizing its wealth generation potential is captured by comparing the price that fishery receives with the price for substantially similar products from other fisheries.

METRIC:
Ratio of average price for wholesale fish product from the fishery, to the global average price for similar species. Convert the price of fish to global currency for comparison (i.e. make sure that both prices are in USD when composing the ratio).

5	More than twice global average
4	120-200% global average
3	Within 20% of global average
2	50-80% of global average
1	Less than half global average

SCORING GUIDANCE:
For products that are traded internationally, it will probably be easiest to compare the export prices. For multiproduct species, use the score that dominates value. Note that these are wholesale prices and not ex-vessel prices.

For US fisheries this website will be useful: http://www.st.nmfs.noaa.gov/commercial-fisheries/foreign-trade/applications/trade-by-product

EXAMPLES:

- Alaska salmon is traded on international markets for $7.5/kilo while Atlantic farmed salmon sells for $7/kilo this means that the ratio is 7.5/7 = a little over 1 and the score is 3.
- Louisiana shrimp could be compared to shrimp from any other country.
- Alaska Pollock could be compared to hoki or hake.

POST-HARVEST PERFORMANCE

MARKET PERFORMANCE

Capacity of Firms to Export to the US and EU

RATIONALE:
Companies with unreliable, low quality or unsecure supply chains may not be able to export to the US or EU without detention. The more freely a company can export to the US or EU, the broader the market. Access reflects the success of quality control systems and breadth of market. It is also a measure of the financial risk associated with international trade.

METRIC:
Percentage of a country's fish exports that meet US or EU health and labeling standards. This is usually a country level metric, though individual high-value fisheries sometimes develop their own supply chains; metric refers to all processing capacity for export, including to regional markets.

5	Over 90% meet US and EU health and labeling standards
4	50-90%
3	Less than 50%
2	A small amount of product meets US/EU standards
1	Banned in the US or EU, or cost of compliance with US/EU standards is prohibitive

SCORING GUIDANCE:
Note that this does not mean that the product necessarily *is* exported to the US/EU, it is based on the regulations and practices guiding the production and whether these satisfy the standards of these countries. There may be other importing countries with stricter standards than the US/EU; clearly, exceeding US/EU standards counts towards meeting them. In general, most fish that is dried/smoked in artisanal fisheries would not meet the standards for export.

EXAMPLES:
Some fisheries score high because they are targeting higher value markets; although they are located in developing countries, the majority of the processing is done in accordance with global standards and the final product is immediately shipped to global markets.

- Some fisheries that have scored a 5: Ugandan Nile Perch, Colombian shrimp, Alaska salmon
- 4: Louisiana shrimp, Seychelles sea cucumber
- 3: Gambia artisanal sole
- 2: Senegal artisanal, Ghana artisanal
- 1: Gambia oysters, Mexican shellfish

POST-HARVEST PERFORMANCE

MARKET PERFORMANCE

Ex-vessel to Wholesale Marketing Margins

RATIONALE:
The value added by processing and marketing at the wholesale level is a direct measure of wealth accumulation in the processing sector. When compared across products, it can also represent how well a fishery is realizing the maximum potential value from its landed fish.

METRIC:
Increase in value of processed wholesale product from unprocessed ex-vessel product.
[Wholesale $/lb – Ex-vessel$/lb]/(Ex-vessel $/lb)

5	More than 200% increase in value
4	100-200%
3	50-100%
2	10-50%
1	Less than 10% increase in value

SCORING GUIDANCE:
In a multi-species or multi-product fishery, score each species/product and then weight by value (calculate the percentage of total revenue contributed by that species/product). Do this for the 5 dominant species/products that contribute the most to total revenue.
For US fisheries this website may be useful: http://www.st.nmfs.noaa.gov/commercial-fisheries/foreign-trade/applications/trade-by-product

EXAMPLE:

- Suppose the wholesale price of Kenyan octopus is $4.25/lb and the ex-vessel price is $2/lb. The calculation would be [4.25-2]/2 = 1.125 or 112.5% increase in value so the score is 4.

POST-HARVEST PERFORMANCE

POST-HARVEST, PROCESSING & SUPPORT INDUSTRY PERFORMANCE

Processing Yield

RATIONALE:

Processing yield is a measure of the potential value of the landed fish that is being realized as wealth. Yield will likely be higher in more efficient processing operations and those with a steady supply of landed product where there is time to take more care in processing and develop downline customers who will pay a premium for reliable forward contracts for premium products. They may also be able to turn processing byproducts (bones, blood) into revenue streams, increasing value per landed weight.

METRIC:

Ratio of actual processing yield (kilos/pounds) to the maximum yield technically achievable:

5	At feasible frontier
4	Within 5% of the feasible frontier
3	Within 10%
2	Within 25%
1	Less than 75% of maximum yield

SCORING GUIDANCE:

In a multi-species or multi-product fishery, score each species/product and then weight by value (calculate the percentage of total revenue contributed by that species/product) for the species/products that contribute the most to total revenue. When products go through multiple layers of processing, this metric refers to primary processing by the first buyer.

In this metric, the emphasis is on the final weight/technically feasible final weight and not on initial starting weight. Estimates of the technical frontier should consider the possibility of converting skin and bones into fish meal. However, if the primary product is fillets then there is no need to consider the yield on byproducts such as fish oil.

EXAMPLE:

- Suppose the two primary products in the Alaska Pollock fishery are surimi and fillets. The yield on surimi scores a 5 and fillets score a 4, but surimi contributes 70% of total revenue so the fishery scores a 5 (0.7*5+0.3*4=4.7, which is rounded up to 5).

Post-Harvest Performance

Post-Harvest, Processing & Support Industry Performance

Shrink

Rationale:

Shrink is the loss of target product that occurs from primary processing through to distribution and is a measure of the potential value of the landed fish that is being realized as wealth. This metric will likely be lower in more efficient processing operations and those with a steady supply of landed product where there is time to take more care in processing and develop downline customers who will pay a premium for reliable forward contracts for premium products. With an efficient processing and handling system there will be very little lost from shore to retail and the processing sector will see economic returns.

Metric:

Percentage of fishery product weight that is lost due to handling, spoilage, theft, bugs, or rats. This is very likely to be an estimate.

5	Less than 5%
4	5-10%
3	10-25%
2	25-50%
1	More than 50%

Scoring Guidance:

In a multi-species or multi-product fishery, score each species/product and then weight by value (calculate the percentage of total revenue contributed by that species/product), for the species/products that contribute the most to total revenue. This metric captures loss through the supply chain, until transferred to the final retailer.

When estimating shrink, consider the product that is lost between the vessel and the first wholesale buyer; do not consider product that is lost or stolen at retail outlets or restaurants. Shrink does not refer to the amount that fish weight changes as the product dries, it refers to lost/spoiled/mishandled product.

Examples:

- In an artisanal LDC fishery where almost all fish landed are used, even spoiled fish may be consumed once fermented, thus shrink may be very low if there is very little theft or damage from pests.
- In the Maldives tuna fishery, shrink occurs as the fish are transferred from the harvester to the processor. If the fish are bruised as they are loaded and unloaded from the tramper, then this is lost product if it is no longer sold.
- Suppose that 10lbs of mackerel are landed, 4lbs are sent to the fresh market and 6lbs are sent to the dried market. Of the 4lbs in the fresh market, 3lbs are sold and 1lb is taken by a seabird. Of the remaining 6lbs in the dried market, 4.8lbs sell for half the price of the fresh mackerel while the other 20% is eaten by rats while it is drying on the beach. In this

case, the primary products are fresh (with a shrink of 25% from the seabird) and dried (with a shrink of 20% from the rats). If fresh fish contributes 2/3 of value while dried fish is 1/3 then the shrink metric should be 2/3*.25+1/3*.2=0.23 and the score is 3.

POST-HARVEST PERFORMANCE

POST-HARVEST, PROCESSING & SUPPORT INDUSTRY PERFORMANCE

Capacity Utilization Rate

RATIONALE:
In many fisheries, a hindrance to wealth accumulation is an excess of capital, even in processing. This may occur because the fishery was once larger than it is now and it is difficult to downsize plants, or because management or biology forces landings to be concentrated in a short period of time. Potential wealth is then consumed in maintaining a larger than necessary facility, or in tying up capital in a facility that is not used to full capacity. In fisheries where landings and processing are concentrated within a short season, this inefficiency may be compounded by using processing technology at a rate that does not support high yields when landings are occurring.

METRIC:
Days open for processing each year. Such days would not normally include religious or civic holidays, or weekly rest days. This should be full time employment days; when the plant is open but only operating at 10% capacity then this should only count for 10% of a day.

5	Virtually year-round
4	75-95% of days
3	50-75%
2	20%-50%
1	Less than 20%

SCORING GUIDANCE AND EXAMPLES:

- In the Tokyo Bay trawl fishery, the primary product is whole fresh fish and it is traded virtually year-round; in this case the score is a 5 even though there is no physical plant the activities of middlemen and fish traders are scored for the processing sector.
- In the Alaska halibut fishery, there used to be a derby that lasted only two days until the TAC was reached; processors would be open for less than 20% of the year and struggle to process all of the TAC very quickly. Now that an ITQ system is in place, the season lasts much longer and processing plants stay open for 50-75% of the year so the score changed from a 1 to a 3.

POST-HARVEST PERFORMANCE

POST-HARVEST, PROCESSING & SUPPORT INDUSTRY PERFORMANCE

Product Improvement

RATIONALE:
One way processors can maximize the value of a product is to market it with improvements that make it more appealing to the consumer, who will then pay more for the product. Sale with a certification, value-enhancing branding or value-added processing can increase wholesale and retail prices, and thus the wealth brought to the fishery.

METRIC:
Proportion of harvest meat weight going into certified, branded, fresh premium, portioned, live or value added products:

5 75-100% of landings are enhanced
4 50-75%
3 25-50%
2 1-25%
1 No landings have enhancements

SCORING GUIDANCE:
Preservation techniques that are used to keep product from spoiling such as smoking/drying/salting/freezing do not count as enhancements.

EXAMPLES:
In some fisheries the majority of the catch is instantly processed into value-added products while in others there is not enough local processing capital or enough demand for enhanced products to merit such efforts. In these cases, the primary concern is typically preservation with the majority of landings being smoked, fermented, or dried in order to preserve them for human consumption but not to add value.

- Some fisheries that have received a 5: Alaska halibut, Alaska crab
- 3: New England groundfish, Norwegian cod
- 1: Kenya artisanal, Lake Victoria dagaa

POST-HARVEST PERFORMANCE

POST-HARVEST, PROCESSING & SUPPORT INDUSTRY PERFORMANCE

Sanitation

RATIONALE:

Sanitation conditions in the landing and processing areas serve as a direct measure of the community benefits that accrue to workers in these sectors and to consumers that eat the products and are less likely to have diseases transmitted by unsanitary conditions. In addition, the sanitation conditions within the processing plants also provide spillover benefits for the larger community as evidence from development economics suggests that peer learning takes place when workers spread their increased knowledge of sanitation to their neighbors and families. Providing information about the health dangers inherent in poor sanitation and training in proper sanitation techniques generates a positive externality for the community.

METRIC:

State of the sanitation conditions in the landing and processing areas. This metric is scored relative to global standards, not local standards.

5	Sanitation in landing and processing areas meets global health standards
4	Basic treatment, but falls short of global standards
3	Human waste is adequately handled, but fish waste presents sanitation issues
2	Functional toilets are available, but fish or fish handlers exposed to untreated sewage
1	Functional toilets are not available in landing or processing areas

SCORING GUIDANCE AND EXAMPLES:

Pit latrines or toilets that are not improved, do not have proper drainage/sewage treatment, and do not allow for proper washing do not count as functional toilets.

These processing facilities meet global health standards:

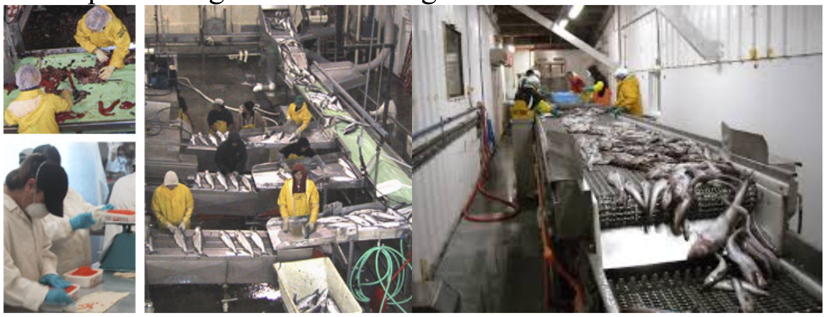

These do not:

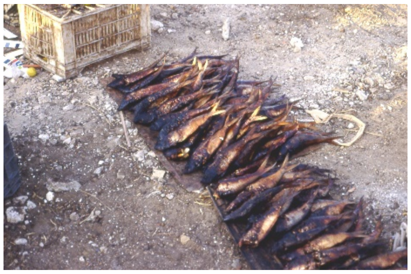

POST-HARVEST PERFORMANCE

POST-HARVEST, PROCESSING & SUPPORT INDUSTRY PERFORMANCE

Regional Support Businesses

RATIONALE:

The strength of the marine support sector is important to realizing the maximum economic gains through efficient harvesting and it also generates important regional multipliers for community well-being. Sales in the support sector are a direct measure of wealth accumulation in the support sector. However, they also reflect the ability of the fishery to access and adopt new technology to make harvesting more efficient and profitable, and the propensity for the fishery to do so, as sales to harvesters support these businesses.

METRIC:

Support Businesses are those that provide critical inputs (e.g., food, ice, gear, boat maintenance) or post-harvest functions (e.g., brokering, logistics).

5	All types of support are plentiful
4	Some types of support are capacity constrained or unavailable
3	Most types of support are capacity constrained or unavailable
2	Support limited to variable inputs
1	Industry support is not locally available

EXAMPLES:

Some fisheries are either located close enough to a major city or generate enough value on their own to ensure that all inputs to both the harvest and post-harvest sectors are available for purchase. In others, the remote nature of the fishery combined with the lack of economic returns means that there is no incentive for support businesses to locate in that area and participants often have to travel long distances to find ice, fuel, or boat maintenance. Some fisheries that have received a

- 5: Tokyo Bay Trawl, California urchin, Pacific groundfish (close to regional hubs with many different industries supporting the commercial fleets)
- 4: Indonesian Blue Crab, Alaska Pollock (more remote fisheries, have to travel relatively for brokers)
- 3: New England groundfish, Seychelles nearshore artisanal (most inputs like maintenance and gear are capacity constrained)
- 2: Senegal artisanal, Western Pacific artisanal tuna (support is limited to food and ice but no others)
- 1: Philippines Blue Crab (remote fishery with no support)

POST-HARVEST PERFORMANCE

POST-HARVEST ASSET PERFORMANCE

Borrowing Rate Compared to Risk-Free Rate

RATIONALE:
The size of the premium the capital market demands to make loans in the processing sector is a direct measure of financial risk in the industry. It is locally normalized to reflect the overall riskiness in the region and the opportunities available to local capital.

METRIC:
Average ratio between the interest rate on loans made in the processing industry to risk-free rates over the last three years. If businesses can access international credit markets, that is appropriate comparison; otherwise, use local risk free rate.

5	Less than 1.75; cf. 30-year conforming mortgage
4	Less than 2.5; cf. personal bank loan
3	Less than 4; cf. good credit card rates
2	Less than 7; cf. bad credit card rates
1	Greater than 7; usury

SCORING GUIDANCE:
Calculation: Interest rate in the processing industry / Risk-free interest rate (average over last 3 years).

Note that if processing businesses can access international credit markets, then the international risk-free rate (US 10 year Treasury bill) is an appropriate comparison, but if businesses are forced to use local credit markets then the benchmark should be local risk-free rates (non-exporting Mexican shellfish processors use the Mexican bond, for example). Typically national/municipal government bonds will be the best representative of local risk-free rates. When scoring, it is often easier to ask the next question about the source of capital funds first and then ask about the rates that they pay. As long as there are credit transactions in the fishery this metric should not be NA; strive to get some estimate of the interest rates that harvesters pay.

In some fisheries, there are cultural or religious prohibitions on interest-based lending. If capital is paid out of cash flow, this can be NA. If proxy measures are used to capture time value of capital, develop a best guess for the metric.

EXAMPLES:

- Alaska Pollock processing companies are able to get unsecured business loans from banks in order to purchase new facilities and they pay 6% interest; these processors have access to international credit markets and the 10 year US Treasury Bill rate in 2013 is 2.8% so the ratio is 6/2.8=2.14 and the score is a 4.
- Suppose that female fish smokers in Kenya take loans from a local microfinance organization and it is difficult to get precise data on the rates that they pay, but anecdotal

evidence suggests that it is more than 5 times the rate on Kenya treasury bonds so the score is a 2 with a quality score of B.

- Some governments subsidize processing loans, making these interest rates artificially low; the subsidized loan rate should be scored as is. For example, if the Kenyan government decided to replace the local microfinance corporation with a national loan program that offered loans for less than 2 times the rate on Kenya treasury bonds; then the score would be a 4 and the fishery would not be penalized just because this is not the interest rate that prevails in free market.

POST-HARVEST PERFORMANCE

POST-HARVEST ASSET PERFORMANCE

Source of Capital

RATIONALE:

Whether lending capital from a particular source is even available is a direct measure of how the capital market assesses risk in the fishery's processing sector. If a certain type of lender or investor is not willing to make capital available in the processing sector at any price, it reveals that it is much riskier than other available investments.

METRIC:

Points to be assigned based on category of lenders or investors that are most typically used in the processing sector. Second scoring method offered if the supply chain (e.g., processors further up the supply chain, parent company, exporters) are the primary source of capital.

5 Unsecured business loans from banks/Venture capital;

4 Secured business loans from banks/Public stock offering; investment from elsewhere in the supply chain

3 Loans from banks secured by personal (not business) assets/Government subsidized private lending/Government-run loan programs/International aid agencies; secured loans from elsewhere in supply chain

2 Microlending/Family/Community-based lending; loans from supply chain significantly reduce margins

1 Mafia/No capital available; exploitative relationship from elsewhere in supply chain

SCORING GUIDANCE:

This metric is less refined than the relative rate, but much easier to obtain. Please note in the worksheet which scoring method is used; i.e. whether or not participants further up the supply chain are the primary source of capital. Processors could be obtaining credit from middlemen, fish traders, or their parent company.

EXAMPLES:

- In a Lake fishery in Malawi, processors finance their operations by borrowing from their family members who are farmers so this is a 2.
- In the artisanal fishery in Ghana processors primarily obtain the funds to purchase new drying racks from middlemen in exchange for lower wholesale prices later on – this is also a 2 because these types of loans significantly reduce margins but are not exploitative.
- Both Alaska Pollock and Nile perch scored a 5 because these are large processing companies that rely on venture capital.
- In Bangladesh, there is no borrowing due to Muslim culture so the score was a 1.
- In Peruvian anchovies, the artisanal and industrial sectors were being scored simultaneously and the artisanal mainly borrow from family while the industrial get unsecured loans from banks so these two were averaged for a final score of 3.

POST-HARVEST PERFORMANCE

POST-HARVEST ASSET PERFORMANCE

Age of Facilities

RATIONALE:

The age of the facilities used in processing harvests, primarily processing plants and storage facilities, reflects several dimensions of fishery wealth. First, it is a direct measure of wealth that has been accumulated from the fishery and reinvested in capital. Second, it is a measure of the potential wealth in the fishery, as newer facilities will be more efficient and less costly to operate. Third, if processors are willing to invest in new capital, it reflects their assessment that the fishery will be profitable into the future. Finally, if new facilities are funded by private loans, newer facilities reflect the capital market's assessment that the fishery is sufficiently low risk to warrant investment.

METRIC:

Average age of the key durable processing capital unit (plants, catcher-processor vessels).

5	1st quarter of expected life; less than 7 years for a building
4	2nd quarter of expected life; 7-15 years
3	third quarter of expected life; 16-20 years
2	4th quarter of expected life; 21-25 years
1	Exceeding expected life; Greater than 25 years

SCORING GUIDANCE AND EXAMPLES:

Average the scores of the top two forms of durable processing capital. Assuming that processing of Pollock takes place on catcher-processors (65%), in shore-based plants (30%) and in Native communities' transient camps (less than 5%) then average the score for the catcher-processors and the plants only. A hut built to smoke fish in an LDC does not have the same lifespan as a modernized processing plant and often such huts have to be rebuilt every season. The hut should not necessarily score the same as the modern plant if they are both 5 years old.

POST-HARVEST PERFORMANCE

PROCESSING OWNERS & MANAGERS

Earnings Compared to Regional Average Earnings

RATIONALE:
This is a direct measure of the type of agents who are attracted to this fishery and become the processing owners and managers. Scaling earnings by average regional earnings reflects whether the fishery is able to attract the most talented workers in the community and is doing well at wealth generation relative to local standards.

METRIC:
Ratio of annual earnings per owner/manager to the regional average earnings. This metric can include wealth accumulated to traders/middlemen if they represent an important part of the supply chain.

5	More than 50% above the regional average
4	Between 10 and 50% above regional average
3	Within 10% of the regional average
2	Between 50 and 90% of the regional average
1	Less than half of the regional average

SCORING GUIDANCE:
This is meant to measure what type of people this fishery attracts so we want to count all their income for an entire year from any sources. These earnings should be compared to regional/national levels depending on the economic sphere of the processors. Economic sphere is defined as the region where they conduct the majority of their economic activity, i.e. village if all economic activity is within the village, but nation if participates in national markets as a consumer. This is a tough metric in seasonal or part-time fisheries where most processors also have other jobs that provide some, or even primary, income but an effort should be made to estimate the earnings of the typical owner from all sources. Make sure that this variable and the following metrics in the owner category are scored for the people who own the means of production; this could be traders if the primary market is fresh.

EXAMPLES:

- In the Alaska crab fishery, most processing owners only receive income from fishing and live out of state doing no work in the off season. In this case, the ratio would be their annual income from crab processing / average annual incomes in the US (assume this is $200,000/$50,000 which is 4 and a score of 5).
- In the Kenyan artisanal fishery, most processing owners are women who also spend part of the year doing work in the fields or running small restaurants/shops. They do not usually travel outside their home village. In this case the ratio is their income from all three sources divided by average incomes in the village (assume this is $1400/$1200 which is 1.2 and the score is 4).

- On Lake Victoria, there is no data on processing owner's earnings or typical household incomes. However, it is clear that owners/managers earn at least a little more than other households in their villages because they live in more expensive homes and are able to afford vehicles. This was scored a 4 with a quality score of B since the scorer was sure that the owners were above the regional average but was unsure by what percentage.

POST-HARVEST PERFORMANCE

PROCESSING OWNERS & MANAGERS

Manager Wages Compared to Non-fishery Wages

RATIONALE:

The processing owner or manager wage is a direct measure of fishery wealth that accumulates to processing managers. It is normalized by wages typical of alternate jobs within the region to provide an indicator of the relative standard of living afforded to managers, and also reflect whether the industry is able to attract the most skilled managers.

METRIC:

Ratio of processing owner's average daily wage in this fishery to average daily wage in the owner's alternative occupations within their economic sphere (e.g., jobs in the village that they qualify for if all economic activity is within the village, but if labor markets are fluid then this should be national average wages in jobs that the owners/managers expect to be able to obtain).

5	More than 50% above the alternative wage
4	Between 10 and 50% above the alternative wage
3	Within 10% of the alternative wage
2	Between 50 and 90% of the alternative wage
1	Less than half of the alternative wage

SCORING GUIDANCE:

This is meant to measure the average personal opportunity cost of participating in this fishery, thus the alternative wage should be the answer to the question of "If you couldn't process fish how much would you get paid?" Look at the average daily wage for processing owners/managers when participating in the fishery and then compare it to the wage in their next best alternative. If the processing owners think that without the fishery they would be lawyers then look up the wages of lawyers, but if they think that they could be subsistence farming then compare it to that wage. Again, this should all be relative to wages within their economic sphere so consider national wages if labor markets are fluid, but restrict the comparison to wages within the village/region if owners seldom leave their local community and do not have the means to do so.

EXAMPLES:

- In the Alaska crab fishery, most processing owners are very successful businessmen who believe that the next best alternative they are qualified for is working as a manager at a large company and earning $500/day (a national average) so the ratio is 800/500=1.6 and the score is 5. In this instance, they face very different alternatives from the harvesting crew because they have very different educational attainment.
- Suppose that in an African fishery, the beach mamas know that if they could not dry fish then they would be farmers and they estimate that they would earn more than 50% less than they currently do so the score is also 5.

POST-HARVEST PERFORMANCE

PROCESSING OWNERS & MANAGERS

Education Access

RATIONALE:

A community that is successfully using its resources will be able to provide high levels of education to its children, ensuring a step beyond resource dependence in the next generation. If processing is an important part of this community, processing owner's families will have access to education.

METRIC:

This metric is based on the highest level of education that is politically and financially accessible to families of processing owners/managers, rather than the actual attainment levels of current processing owners/managers. The level of education accessible to (available and affordable) the families (i.e., children) of processing owners/managers.

5	Higher education is accessible
4	High school level education or advanced technical training is accessible
3	Middle school level education or simple technical training is accessible
2	Basic literacy and arithmetic training is accessible
1	Formal education is not accessible

SCORING GUIDANCE:

This metric is meant to capture whether processing owners/managers can afford to provide education for their children. Just because there is a high school in the village does not mean that they can afford to send their children – consider school fees, tuition, and opportunity cost. Note that learning to fish or process fish as an on the job apprenticeship does not count as formal technical training. Advanced technical training involves science/technology and most apprenticeships in LDCs do not count and should be classified as simple technical training at the most.

EXAMPLES:

- In Uganda, children of processors seldom go to school beyond primary levels because they are needed to work in the fields or on the boats and the score is a 2.
- In the Alaska Pollock fishery and many other industrialized fisheries, the processing owners are very wealthy businessmen who can definitely afford to send their children to university so the score is a 5.
- In some rural fisheries, processing owners make enough money to afford university tuition, but the university is far away and they cannot afford to pay for the transport and housing so most of their children only attend high school in the local village and do not go to university; this is a 4.

Post-Harvest Performance

Processing Owners & Managers

Access to Health Care

Rationale:

A community that is successfully using its resources will be able to provide high levels of health care, ensuring a quality of life and decreasing health risk. If processing is an important part of this community, processing owner's families will have access to the best available health care.

Metric:

This metric is based on the quality of health care that is politically and financially accessible to processing owners and managers. The level of health care accessible to (available and affordable) the processing owners/managers and their families.

5	Global standard treatment for illness is accessible
4	Licensed doctors provide trauma, surgical and drug treatments
3	Nurses or medical practitioners provide emergency and routine drug treatments
2	Basic and simple drug treatment is accessible
1	Medical or drug treatment is not accessible

Scoring Guidance:

This metric is meant to capture whether processing owners can afford to provide health care for themselves and their children. Just because there is a clinic in the village does not mean that they can afford it – consider medical fees, travel time and opportunity cost. If there is a hospital located in the main city 4 hours away then global standard treatment for illness is not accessible because if there was a major trauma then the injured party would likely die before reaching medical assistance (unless there is a Coast Guard helicopter assigned to transport injured processors/family members). Note that the scores for this metric are likely to be the same across processing owners and workers if they and their families live in the same communities and make enough money to afford the health care that is offered there. It will be different if owners/managers can afford to travel to urban centers for emergency or surgical procedures while workers cannot.

Examples:

- In the Alaska crab fishery, most of the processing owners' families live in suburban communities where any kind of health care is accessible and they can afford any type of procedure so the score is a 5.
- In rural Bangladesh, there is a small clinic with nurses equipped for emergencies but the sanitation is poor and there are no doctors. Processors seldom travel to the hospital in the capitol because the cost of travel is prohibitive. This is a 3.

POST-HARVEST PERFORMANCE

PROCESSING OWNERS & MANAGERS

Social Standing of Processing Managers

RATIONALE:

This is a proxy for income associated with owning or running processing plants, which may be much easier to collect than actual wage information. Social standing reflects whether the fishery is able to attract the most talented workers in the community and is doing well at wealth generation relative to local standards.

METRIC:

5	Among the most respected in the community, comparable with civic and religious leaders and professionals, such as doctors and lawyers
4	Comparable to management and white collar jobs
3	Comparable to skilled labor jobs
2	Comparable to unskilled blue collar or service jobs
1	Among the least respected, such as slaves or indentured servants

SCORING GUIDANCE:

This is meant to reflect the amount of esteem that processing owners receive in their local community. Make sure that answers for this metric make sense relative to the social status of the other fishery occupations (boat owners, crew, and processing workers). Consider their social status within their primary community. The comparison group should be the region where they spend the majority of their time and income.

EXAMPLES:

- Processing owners/managers in the Alaska pollock fishery are held in very high esteem in the large cities like Seattle where they live – some can even afford private jets and are definitely on par with civic and religious leaders, this is a 5.
- In the Liberian artisanal fishery, fishing is prized by the local community but the processing is done by women who get less respect in this society so the score is a 2.

POST-HARVEST PERFORMANCE

PROCESSING & SUPPORT INDUSTRY PERFORMANCE

Nonresident Ownership of Processing Capacity

RATIONALE:
The ability of a country or region to improve itself using its resources depends on its ability to maintain local multipliers by keeping wealth within the region. A large portion of nonresident owned processing reflects that much of the processing wealth will be leaving the region, failing to boost the regional economy. In developing regions, it may also reflect an inability of local resource users to generate sufficient capital to process.

METRIC:
Proportion of ex-vessel value processed by regionally owned processing capital. "Local" is defined as coming from, and spending their earnings within, the local fishing community. Nationals who are transient nonresidents, or considered outsiders in the fishing community, are not local

5	95-100% local
4	70-95% local
3	35-70% local
2	5-35% local
1	Virtually no locally owned processing

SCORING GUIDANCE AND EXAMPLES:
- In the case of the Alaska salmon fishery, local should be defined as coming from the state of Alaska and not restricted to residents of rural Alaska.
- Foreign nationals who are not technically citizens but who live, raise their families, and spend their money in the region should be considered local.
- In Liberia there is a large population of Ghanaians who have lived in Liberia for generations and do not use their earnings to support people in other regions so they are considered local.
- In the Alaska Pollock fishery, the three main processing companies have their headquarters in Seattle or Japan so the score is a 1.

POST-HARVEST PERFORMANCE

PROCESSING WORKERS

Earnings Compared to Regional Average Earnings

RATIONALE:
This is a direct measure of the type of agents who are attracted to this fishery and become the processing workers. Scaling earnings by average regional earnings reflects whether the fishery is able to attract the most talented workers in the community and is doing well at wealth generation relative to local standards.

METRIC:
Ratio of annual earnings per processing worker to the regional average earnings.

5	More than 50% above the regional average
4	Between 10 and 50% above the regional average
3	Within 10% of the regional average
2	Between 50 and 90% of the regional average
1	Less than half of the regional average

SCORING GUIDANCE:
This is meant to measure what type of people this fishery attracts so we want to count all their income for an entire year from any sources. These earnings should be compared to regional/national levels depending on the economic sphere of the processing workers. Economic sphere is defined as the region where they conduct the majority of their economic activity, i.e. village if all economic activity is within the village, but nation if participates in national markets as a consumer. This is a tough metric in seasonal or part-time fisheries where most processors also have other jobs that provide some, or even primary, income but an effort should be made to estimate the earnings of the typical worker from all sources. Make sure that this variable and the following metrics in the worker category are scored for the people who work for wages and not those who own the processing facilities or who are self-employed and engaged in trading/selling the fish.

EXAMPLES:

- In the Alaska salmon fishery, most processing workers receive income from salmon processing and also work as dishwashers in the off season. They live out of state and get flown in to work at the processing plant. In this case, the ratio would be their annual income from crab processing and dishwashing / average annual incomes in the US (assume this is $46,000/$50,000 which is 0.92 and a score of 3).
- In the Kenyan artisanal demersal fishery, most processing workers are women who also spend part of the year doing work in the fields or running small restaurants/shops. They do not usually travel outside their home village. In this case the ratio is their income from all three sources divided by average incomes in the village (assume this is $1000/$1200 which is 0.83 and the score is 2).
- On Lake Victoria, there is no data on processing workers' earnings or typical household incomes. However, it is clear that workers earn approximately the same amount as other

households in their villages because they live in the same areas and can afford similar homes. This was scored a 3 with a quality score of B since the scorer was sure that the workers were within 50% of the regional average.

POST-HARVEST PERFORMANCE

PROCESSING WORKERS

Worker Wages Compared to Non-fishery Wages

RATIONALE:
The processing worker wage is a direct measure of fishery wealth that accumulates to processing workers. It is normalized by wages typical of alternate jobs within the region to provide an indicator of the relative standard of living afforded to workers, and also reflect whether the fishery is able to attract the most skilled workers.

METRIC:
Average ratio of a processing worker's average daily wage in this fishery to the average daily wage in the worker's alternative occupations within their economic sphere (e.g., jobs in the village that they qualify for if all economic activity is within the village, but if labor markets are fluid then this should be national average wages in jobs that the workers expect to be able to obtain).

5	More than 50% above the alternative wage
4	Between 10 and 50% above the alternative wage
3	Within 10% of the alternative wage
2	Between 50 and 90% of the alternative wage
1	Less than half of the alternative wage

SCORING GUIDANCE:
This is meant to measure the average personal opportunity cost of participating in this fishery, thus the alternative wage should be the answer to the question of "If you couldn't process fish how much would you get paid?" Look at the average daily wage for processing workers when participating in the fishery and then compare it to the wage in their next best alternative. If the processing workers think that without the fishery they would be washing dishes then look up the wages of dishwashers, but if they think that they could be subsistence farming then compare it to that wage. Again, this should all be relative to wages within their economic sphere so consider national wages if labor markets are fluid, but restrict the comparison to wages within the village/region if workers seldom leave their local community and do not have the means to do so.

EXAMPLES:

- In the New England groundfish fishery, most processing workers are recent immigrants who believe that the next best alternative they are qualified for is working as a dishwasher at a restaurant for minimum wage and earning $50/day (a national average) so the ratio is their daily wage of $100 in the fishery divided by $50=2 and the score is 5. In this instance, they face very different alternatives from the harvesting crew because they have different educational attainment and possibly different immigration status.
- In Sierra Leone, the beach mamas know that if they could not dry fish then they would be farmers and they estimate that they would earn more than 50% less than they currently do so the score is also 5.

POST-HARVEST PERFORMANCE
PROCESSING WORKERS

Education Access

RATIONALE:
A community that is successfully using its resources will be able to provide high levels of education to its children, ensuring a step beyond resource dependence in the next generation. If processing is an important part of this community, processing worker's families will have access to education.

METRIC:
This metric is based on the highest level of education that is politically and financially accessible to families of processing workers, rather than the actual attainment levels of current processing workers. The level of education accessible to (available and affordable) the families (e.g, children) of processing workers.

5	Higher education is accessible
4	High school level education or advanced technical training is accessible
3	Middle school level education or simple technical training is accessible
2	Basic literacy and arithmetic training is accessible
1	Formal education is not accessible

SCORING GUIDANCE:
This measure is meant to capture whether processing workers can afford to provide education for their children. Just because there is a high school in the village does not mean that they can afford to send their children – consider school fees, tuition, and opportunity cost. Note that learning to fish or process fish as an on the job apprenticeship does not count as formal technical training. Advanced technical training involves science/technology and most apprenticeships in LDCs do not count and should be classified as simple technical training at the most.

EXAMPLES:
- In Uganda, children of processing workers seldom go to school beyond primary levels because they are needed to work in the fields or on the boats and the score is a 2.
- In the Alaska pollock fishery and many other industrialized fisheries, the processing workers earn enough to send their children to public universities, this is a 5.
- In some rural fisheries, processing workers make enough money to afford university tuition, but the university is far away and they cannot afford to pay for the transport and housing so most of their children only attend high school in the local village and do not go to university; this is a 4.

POST-HARVEST PERFORMANCE

PROCESSING WORKERS

Access to Health Care

RATIONALE:

A community that is successfully using its resources will be able to provide high levels of health care, ensuring a quality of life and decreasing health risk. If processing is an important part of this community, processing worker's families will have access to the best available health care.

METRIC:

This metric is based on the quality of health care that is politically and financially accessible to processing workers. The level of health care accessible to (available and affordable) the processing workers and their families.

5	Global standard treatment for illness is accessible
4	Licensed doctors provide trauma, surgical and drug treatments
3	Nurses or medical practitioners provide emergency and routine drug treatments
2	Basic and simple drug treatment is accessible
1	Medical or drug treatment is not accessible

SCORING GUIDANCE:

This metric is meant to capture whether processing workers can afford to provide health care for themselves and their children. Just because there is a clinic in the village does not mean that they can afford it – consider medical fees, travel time and opportunity cost. If there is a hospital located in the main city 4 hours away then global standard treatment for illness is not accessible because if there was a major trauma then the injured party would likely die before reaching medical assistance (unless there is a Coast Guard helicopter assigned to transport injured processors/family members). Note that the scores for this metric are likely to be the same across processing owners and workers if they and their families live in the same communities and make enough money to afford the health care that is offered there. It will be different if owners/managers can afford to travel to urban centers for emergency or surgical procedures while workers cannot.

EXAMPLES:

- In the Alaska crab fishery, most of the processing workers' families live in suburban communities where any kind of health care is accessible and they can afford most types of procedures so the score is a 5.
- In rural Bangladesh, there is a small clinic with nurses equipped for emergencies but the sanitation is poor and there are no doctors. Processors seldom travel to the hospital in the capitol because the cost of travel is prohibitive. This is a 3.

POST-HARVEST PERFORMANCE

PROCESSING WORKERS

Social Standing of Processing Workers

RATIONALE:
This is a proxy for income associated with working in processing plants, which may be much easier to collect than actual wage information. Social standing reflects whether the fishery is able to attract the most talented workers in the community and is doing well at wealth generation relative to local standards.

METRIC:

5	Among the most respected in the community, comparable with civic and religious leaders and professionals, such as doctors and lawyers
4	Comparable to management and white collar jobs
3	Comparable to skilled labor jobs
2	Comparable to unskilled blue collar or service jobs
1	Among the least respected, such as slaves or indentured servants

SCORING GUIDANCE:
This is meant to reflect the amount of esteem that processing workers receive in their local community. Make sure that answers for this metric make sense relative to the social status of the other fishery occupations (boat owners, crew, and processing owners). Consider their social status within their primary community. The comparison group should be the region where they spend the majority of their time and income.

EXAMPLES:

- Processing workers in the Alaska pollock fishery are held in fairly low esteem in the large cities like Minneapolis where they live –they are viewed as equivalent to unskilled blue collar jobs, this is a 2.
- In the Liberian artisanal fishery, fishing is prized by the local community but the processing is done by women who get less respect in this society so the score is also a 2.

POST-HARVEST PERFORMANCE

PROCESSING WORKERS

Proportion of Nonresident Employment

RATIONALE:
The ability of a country or region to improve itself using its resources depends on its ability to maintain local multipliers by keeping wealth within the region. A large portion of nonresident processing workers reflects that much of the processing wealth will be leaving the region, failing to boost the regional economy.

METRIC:
Proportion of processing workers employed who are local. “Local” is defined as coming from, and spending their earnings within, the local fishing community. Nationals who are transient nonresidents, or considered outsiders in the fishing community, are not local.

5	95-100% local
4	71-95% local
3	36-70% local
2	5-35% local
1	Virtually no local processing crew

SCORING GUIDANCE AND EXAMPLES:
In the case of the Alaska salmon fishery, local should be defined as coming from the state of Alaska and not restricted to residents of rural Alaska. Foreign nationals who are not technically citizens but who live, raise their families, and spend their money in the region should be considered local. For example, in Liberia there is a large population of Ghanaians who have lived in Liberia for generations and do not use their earnings to support people in other regions so they are considered local. In the Alaska Pollock fishery, the vast majority of processing workers are flown in for the season from distant cities like Minneapolis so the score is a 1.

POST-HARVEST PERFORMANCE

PROCESSING WORKERS

Worker Experience

RATIONALE:
The rate at which workers turn over in the fishery is an indirect measure of several key variables. First, it reflects wealth accumulation to workers, because a worker will only stay in the fishery if the wage is comparable to, or better than, other jobs he could obtain. Second, worker longevity often means the workers are resident in the community, and thus their earnings stay in the community and are spent locally, rather than being sent away by itinerant or immigrant workers. Third, experienced workers develop specialized knowledge and refined skills that make processing more efficient, so the fishery is better able to reach its wealth-generating potential.

METRIC:
Average years of experience of processing workers.

5	More than 10 years (skilled career workers)
4	5-10 years
3	3-5 years
2	1-3 years
1	0 full years of experience (mostly new workers each season)

EXAMPLE:

- In the case of the Alaska salmon fishery, most processing workers are new each season so the fishery scored a 1.

III. Fishery Performance Indicators

Inputs

Enabling the Creation of Sustainable Incomes and Ecosystem Health

III. FISHERY PERFORMANCE INDICATORS - Inputs

This section identifies 54 inputs that may lead to the generation of sustainable livelihoods and healthy ecosystems (see Table 2 below). Each metric (last column in Table 2) is individually explained in the following pages and is accompanied by examples from the set of existing case studies. In addition, each metric includes a rationale that demonstrates the existing theoretical or empirical arguments from the fisheries literature that justify the inclusion of the metric.

Table 2. Fishery Performance Indicators–Inputs

Component	Dimension	Measure
Macro Factors	General Environmental Performance	Environmental Performance Index (EPI)
	Exogenous Environmental Factors	Disease and Pathogens
		Natural Disasters and Catastrophes
		Pollution Shocks and Accidents
		Level of Chronic Pollution (Stock effects)
		Level of Chronic Pollution (Consumption effects)
	Governance	Governance Quality
		Governance Responsiveness
	Economic Conditions	Index of Economic Freedom
		Gross Domestic Product (GDP) Per Capita
Property Rights & Responsibility	Fishing Access Rights	Proportion of Harvest Managed Under Limited Access
		Transferability
		Security
		Durability
		Flexibility
		Exclusivity
	Harvest Rights	Proportion of Harvest Managed with Rights-based Management
		Transferability
		Security
		Durability
		Flexibility
		Exclusivity
Co-Management	Collective Action	Proportion of Harvesters in Industry Organizations
		Harvester Organization Influence on Fishery Management & Access
		Harvester Organization Influence on Business & Marketing
	Participation	Days in Stakeholder Meetings
		Industry Financial Support for Management
	Community	Leadership
		Social Cohesion
	Gender	Business Management Influence
		Resource Management Influence
		Labor Participation in Harvest Sector
		Labor Participation in Post-Harvest Sector
Management	Management Inputs	Management Expenditure to Value of Harvest
		Enforcement Capability
		Management Jurisdiction
		Level of Subsidies
	Data	Data Availability
		Data Analysis
	Management Methods	MPAs and Sanctuaries
		Spatial Management
		Fishing Mortality Limits
Post-Harvest	Markets & Market Institutions	Landings Pricing System
		Availability of Ex-vessel Price & Quantity Information
		Number of Buyers
		Degree of Vertical Integration
		Level of Tariffs
		Level of Non-tariff Barriers
	Infrastructure	International Shipping Service
		Road Quality
		Technology Adoption
		Extension Service
		Reliability of Utilities/Electricity
		Access to Ice & Refrigeration

MACRO FACTORS

GENERAL ENVIRONMENTAL PERFORMANCE

Environmental Performance Index (EPI)

RATIONALE:
Wealth creation is dependent on the general condition of the environment. A review of current research shows that fisheries are predominantly affected by agricultural runoff, municipal sewage, oil pollution, and trace metals, all of which are reflected in a country's general environmental health (Islam and Tanaka, 2004). An Environmental Performance Index (EPI) has been developed to evaluate: 1) environmental health and 2) ecosystem vitality (Esty *et al*. 2008) at the national level for the majority of countries around the globe.

METRIC:
The EPI considers factors such as disease, water quality, air pollution, biodiversity, natural resources and climate change. The EPI ranges from 1-100. Score is by 2010 EPI quintiles:

5	EPI of 69-100
4	62-69
3	56-62
2	47-56
1	1-47

SCORING GUIDANCE:
Visit the website http://epi.yale.edu/epi2012/countryprofiles and select the country from the drop down menu or visit http://epi.yale.edu/epi2012/map and select the country on the map. The score used should be the aggregate "Environmental Performance Indicator" score and not the score for one of the individual components. Make sure that the raw score is not reported, but instead place it within one of the above bins and determine whether this is a score of 1-5. If the country is not given an EPI score, a best guess can be made based on prevailing environmental conditions and conditions in neighboring countries. If the fishery is transnational, then weight the EPI scores of each country depending on the portion of landings value that occur in the country.

MACRO FACTORS

EXOGENOUS ENVIRONMENTAL FACTORS

Disease and Pathogens

RATIONALE:
Even a well-managed fishery can fail to accumulate wealth if exogenous events or conditions threaten the stock, or the harvestability of the stock (Behringer et al., 2012). As the theoretical models of parasitology suggest, the prevalence of disease amongst a population of fish can have impacts on the stock independent of the level of exploitation in the fishery (Dobson and May, 1987). This metric is intended primarily to identify when other management inputs will not be correlated with outcomes for reasons of that are exogenous to the fishery. In particular, this metric incorporates the effect of diseases and pathogens that have been shown to affect harvest values independent of management action (Asche et al., 2010).

METRIC:
This metric is based on the extent to which harvest value is thought to be adversely affected by exogenous disease, pathogens, toxic algaes or similar factors (e.g., lobster shell disease or red tides).

5	Harvest value unaffected by disease
4	Harvest value reduced by less than 10%
3	Harvest value reduced by 10-30%
2	Harvest value reduced by more than 30%
1	Harvest value almost completely eliminated by disease

SCORING GUIDANCE:
This score should be based on empirical or anecdotal evidence of disease affecting harvest values. Even if diseased fish are immediately thrown back, they still impose a time cost and affect harvest values. The impact on harvest value could come through reduced catch or through lower prices for the affected portion of catch.

EXAMPLES:
Some fisheries where harvests have been known to be affected by disease are:

- Pacific Salmon (infected by the parasite *Henneguya salminicola*)
- Caribbean spiny lobster (commonly infected by the pathogenic Pav1 virus)
- Scallops (affected by toxic algae blooms).
- The Florida spiny lobster fishery scored a 3 for this metric as that was the best guess for how the Pav1 virus affects harvest values.

MACRO FACTORS

EXOGENOUS ENVIRONMENTAL FACTORS

Natural Disasters and Catastrophes

RATIONALE:

Even a well-managed fishery can fail to accumulate wealth if exogenous events or conditions threaten the stock, or the harvestability of the stock. This metric is intended primarily to identify when other management inputs will not be correlated with outcomes for reasons exogenous to the fishery. In particular, this metric incorporates the effect of natural disasters which have been shown to affect harvest values independent of management action (Athukorala and Resosudarmo, 2005).

METRIC:

This metric is based on the extent to which harvest values are thought to be adversely affected by natural disasters such as earthquakes, volcanoes, tsunamis, hurricanes and typhoons. These are typically one-time events, not long-term ecosystem scale shifts induced by climate change (e.g., shifts in temperature or salinity). Harvest values can be affected through stock effects or damage to harvest capacity.

5	Harvest value unaffected by disaster
4	Harvest value reduced by less than 10%
3	Harvest value reduced by 10-30%
2	Harvest value reduced by more than 30%
1	Harvest value almost completely eliminated by disaster

SCORING GUIDANCE:

This score should be based on empirical or anecdotal evidence of natural disasters affecting harvest values. Even if an earthquake had no effect on the stock of fish, it may have destroyed vessels or landing facilities that would lead to a reduction in catch. Note that this does not refer to potential or theoretical natural disasters but only to the effects of actual historic events. Natural disasters that occurred in the past should only be included in the score if they continue to affect harvest values in the present. For example, the 2011 Japanese earthquake and ensuing tsunami should only impact the 2013 score of Japanese fisheries if the fleets have yet to be rebuilt to full capacity, the stocks have yet to recover

A potentially useful source for the impact of hurricanes/weather on US fisheries:
http://weather.noaa.gov/

EXAMPLES:

- The 2004 Indonesian tsunami killed thousands of harvesters, temporarily displaced many rural fishing communities and destroyed their harvest capital. If the affected fisheries were being scored in the years immediately after this disaster when fleets had yet to recover, the score should reflect a decline in harvest value (probably scoring a 1 or 2) even though the disaster did not take place in the same year.

- Episodic coral bleaching that results from extreme and temporary shifts in ocean temperature is another example of a natural event that would fit in this category; this led to reef fisheries in the south of Kenya scoring a 3 two years after the initial event.
- The Alaska crab fishery scored a 4 since encroaching sea ice sometimes shortens the fishing season and reduces harvest value.

MACRO FACTORS
EXOGENOUS ENVIRONMENTAL FACTORS

Pollution Shocks and Accidents

RATIONALE:
Even a well-managed fishery can fail to accumulate wealth if exogenous events or conditions threaten the stock, or the harvestability of the stock. This metric is intended primarily to identify when other management inputs will not be correlated with outcomes for reasons exogenous to the fishery. In particular, this metric incorporates the effect of pollution shocks that have been shown to affect harvest values independent of management action (Cohen, 1995).

METRIC:
This metric is based on the extent to which harvest value in the reference year is thought to be adversely affected by pollution shocks, such as oil spills, industrial accidents, peak runoff events, or piracy. These are typically one-time events, not chronically high levels of pollution.

5	Harvest value unaffected by shocks
4	Harvest value reduced by less than 10%
3	Harvest value reduced by 10-30%
2	Harvest value reduced by more than 30%
1	Harvest value almost completely closed by shocks

SCORING GUIDANCE:
This score should be based on empirical or anecdotal evidence of pollution shocks affecting harvest values. Note that this should not be based on the potential for theoretical accidents, but only on the effect of actual shocks that have occurred. Also note that the influence of runoff or dumping that occurs every year should not show up in this metric; that is considered chronic pollution and it is included in the next metric. Oil spills and other industrial accidents that occurred in the past should only be included in the score if they continue to affect harvest values in the present. For example, the 1989 Exxon Valdez oil spill should only have impacted the 1990 score of Alaskan fisheries if the stocks had yet to recover. If markets still discount Japanese fish in 2013 to reflect radiation fears from the nuclear plant meltdown in 2011, this would be considered a shock.

EXAMPLES:
- In addition to Exxon Valdez, other examples of pollution shocks or accidents that would fit in this category are hijacking by Somali pirates in the Indian Ocean tuna fishery and the 2010 Deepwater Horizon large scale oil spill in the Gulf of Mexico that scared consumers away from buying seafood.
- Another example of a one-time pollution shock is an inland fishery in Bangladesh where the harvesters reported vandalism/poisoning of their fish stocks in the reference year, which lead to the fishery scoring a 3.

MACRO FACTORS

EXOGENOUS ENVIRONMENTAL FACTORS

Level of Chronic Pollution (Stock Effects)

RATIONALE:
Even a well-managed fishery can fail to accumulate wealth if exogenous events or conditions threaten the stock, or the harvestability of the stock. This metric is intended primarily to identify when other management inputs will not be correlated with outcomes for reasons exogenous to the fishery. In particular, this metric incorporates the effect of chronic pollution that has been shown to affect harvest values through diminished stock health (Edinger et al., 1998). As the biological literature asserts, pollution from industrial development and land use is responsible for depleting fish stocks through acidification, sedimentation and loss of habitat (Maitland, 1995).

METRIC:
This metric is based on the level of chronic pollution that is detected in the fishery. Chronic pollution can be either always present, or frequently recurring, such as after each moderate rainfall. Extent to which chronic pollution, such as from industrial or agricultural runoff, affects the stock.

5	Not detectable
4	Minimal detectable levels
3	High levels detected
2	Pollution affects stock growth
1	Pollution leading to severe stock decline

SCORING GUIDANCE:
This metric should be based on empirical or anecdotal evidence of chronic pollution affecting stocks. This metric should not reflect the prevalence of overfishing; when stocks are declining the scorer should attempt to estimate the portion of that decline that can be attributed to chronic pollution and isolate this from the impact of overharvesting. Note that the emphasis for this metric is on the impact of pollution on fish stocks; consumption effects will be captured in the next metric. Also note that this metric should not include the impact of one-time pollution shocks or accidents as that was captured in the last metric.

EXAMPLES:
- A coastal fishery in Sierra Leone scored a 2 due to the presence of plastic garbage, urban and industrial runoff, and increasing siltation due to mining activities.
- In contrast, the Alaska salmon, halibut, and crab fisheries all scored a 5; due to the remote nature of these fisheries and the lack of industrial activity, there is no evidence of chronic pollution.

MACRO FACTORS

EXOGENOUS ENVIRONMENTAL FACTORS

Level of Chronic Pollution (Consumption Effects)

RATIONALE:

Even a well-managed fishery can fail to accumulate wealth if exogenous events or conditions threaten the stock, or the harvestability of the stock. This metric is intended primarily to identify when other management inputs will not be correlated with outcomes for reasons exogenous to the fishery. In particular, this metric incorporates the effect of chronic pollution that has been shown to affect harvest values through decreased consumer demand. Shimshack et al. (2007) demonstrated that an FDA advisory about methyl-mercury levels in canned fish products caused both at-risk individuals and non-targeted individuals to lower their consumption.

METRIC:

This metric is based on the level of chronic pollution that is detected in the fishery. Chronic pollution can be either always present, or frequently recurring, such as after each moderate rainfall. Extent to which chronic pollution limits consumption.

5	No consumption affected
4	Minimal consumption affected
3	Official consumption advisories
2	Temporarily ban harvest for consumption
1	Completely closed for consumption

SCORING GUIDANCE:

This metric should be based on empirical or anecdotal evidence of chronic pollution affecting consumption. Note that the emphasis for this metric is on how consumers perceive the fish as result of information about pollution; the stock effects were captured in the previous metric. Also note that this metric should not include the impact of one-time pollution shocks or accidents as that was captured in a previous metric. When local markets are unaffected by pollution because local consumers are unaware of the threat or do not care about its health effects, the fishery should obtain a high score. However, if there is a sense that export is impeded because foreign market consumers have concerns about pollution (and this is the binding constraint, rather than health codes or market limitations) then the score should reflect this.

EXAMPLES:

- A fishery in Bangladesh scored a 5; although there have been no scientific studies on the pollution levels, all the fish product goes exclusively to local markets where consumers are not concerned about pollution issues.
- An artisanal fishery in Ghana scored a 4 because although there was evidence of agrochemical runoff and consumers noticed that the taste of fish from a certain region had changed, it had minimal impact on their price and quantity consumed.
- Government bans on import of Japanese seafood products following the Fukushima nuclear disaster are not an example of chronic pollution so the Japanese fisheries should continue to score high in this metric but should score lower in natural disasters and pollution shocks if consumption continues to be affected.

- Species such as tilefish, swordfish, shark, and mackerel for which the EPA and FDA have released joint warnings about the dangers of mercury poisoning should receive a score of 3.

MACRO FACTORS
GOVERNANCE

Governance Quality

RATIONALE:
Good governance, starting with a functional central government, can be an essential condition for sustainable fisheries and wealth creation (Hilborn, 2007a). As Hilborn (2007a) asserts, even if decentralization and community-based management is the ideal form of management for a fishery, such local systems are still dependent upon well-functioning central governments for recognition and support. Local management is frequently undermined by bribery and corruption within the central government that allows foreign fleets or unlicensed harvesters unlimited access (Hilborn, 2007b). The World Bank has developed a Worldwide Governance Indicator which considers six dimensions: Voice & Accountability, Political Stability and Absence of Violence/Terrorism, Government Effectiveness, Regulatory Quality, Rule of Law and Control of Corruption (Kaufman, *et al*. 2008).

METRIC:
The Governance Indicators (Kaufman, D., A. Kraay, and M. Mastruzzi, 2008) assign countries to ranks based on six dimensions. This metric is the average of four indicators in the World Bank's Governance Indicators, each scored [-2.5, 2.5]

- Government Effectiveness
- Regulatory Quality
- Rule of Law
- Control of Corruption

5	Above 0.92 (highest-performing 2010 quintile)
4	0.10 to 0.92
3	-0.43 to 0.10
2	-0.81 to -0.43
1	Below -0.81 (lowest-performing 2010 quintile)

SCORING GUIDANCE:
Visit the website http://info.worldbank.org/governance/wgi/index.aspx#reports then click on the "Table View" tab, lastly select the country and the most recent year of data available from the drop down menu. Average the "Governance Score" for the four indicators listed above. Make sure that the raw score is not reported, but instead place it within one of the above bins and determine whether this is a score of 1-5. If the fishery is transnational then weight the WGI scores of each country depending on the portion of total revenue that occurs in the country.

MACRO FACTORS

GOVERNANCE

Governance Responsiveness

RATIONALE:

Good governance, starting with a functional central government, is an essential condition for sustainable fisheries and wealth creation (Hilborn, 2007a). As Hilborn (2007a) asserts, even if decentralization and community-based management is the ideal form of management for a fishery, such local systems are still dependent upon well-functioning central governments for recognition and support. Two alternate input metrics for macro governance were introduced in order to determine whether factors such as control of corruption or responsiveness to local concerns play a larger role in determining outcomes. The World Bank has developed a Worldwide Governance Indicator which considers six dimensions: Voice & Accountability, Political Stability and Absence of Violence/Terrorism, Government Effectiveness, Regulatory Quality, Rule of Law and Control of Corruption (Kaufman, *et al*. 2008).

METRIC:

The Governance Indicators (Kaufman, D., A. Kraay, and M. Mastruzzi, 2008) assign countries to ranks based on six dimensions. This metric is the average of two indicators in the World Bank's Governance Indicators, each scored [-2.5, 2.5]

- Voice and Accountability
- Political Stability

5	Above 0.96 (highest-performing 2010 quintile)
4	0.41 to 0.96
3	-0.24 to 0.41
2	-0.82 to -0.24
1	Below -0.82 (lowest-performing 2010 quintile)

SCORING GUIDANCE:

Visit the website http://info.worldbank.org/governance/wgi/index.aspx#reports then click on the "Table View" tab, lastly select the country and the most recent year of data available from the drop down menu. Average the "Governance Score" for the two indicators listed above. Make sure that the raw score is not reported, but instead place it within one of the above bins and determine whether this is a score of 1-5. If the fishery is transnational then weight the WGI scores of each country depending on the portion of total revenue that occurs in the country.

MACRO FACTORS

ECONOMIC CONDITION

Index of Economic Freedom

RATIONALE:

Wealth creation is dependent on the institutional setting and economic conditions in a given country. Edwards (1999) demonstrates that the macroeconomic changes within the United States from 1880-1995 had a large impact on harvesting patterns independent of the management regime. The Heritage Foundation/*Wall Street Journal, Index of Economic Freedom* (IEF) reflects the overall economic freedom of the nation within which the fishery sector operates (Miller and Holmes, 2009). The *Index of Economic Freedom* includes 10 broad institutional factors:

- Business freedom
- Trade freedom
- Fiscal freedom
- Government size
- Monetary freedom
- Investment freedom
- Financial freedom
- Property rights
- Freedom from corruption
- Labor freedom

Construction of the index relies on several other studies for its data sources, including the World Bank's *Doing Business* Economist Intelligence Unit (The World Bank 2009a), the US Department of Commerce, the World Bank's *World Development Indicators* (The World Bank, 2009b), Eurostat, International Monetary Fund reports, Transparency International's, *Corruption Perceptions Index* (Transparency International, 2009) and several other documents.

METRIC:

Country's score from the Heritage Foundation's Index of Economic Freedom. A detailed discussion of these factors and methodology is found in Miller and Holmes (2009). Bins defined based on 2010 percentiles.

5	IEF of 69.2-100
4	62.5-69.1
3	57.1-62.4
2	50.5-57.0
1	1-50.5

SCORING GUIDANCE:

Visit the website http://www.heritage.org/index/default then click on "Explore the Data" and find the country in the list. Consult the "Overall Score" column. Make sure that the raw score is not reported, but instead place it within one of the above bins and determine whether this is a score of 1-5. If the country does not have an overall score then average the dimensions for which it is scored, but make note of which columns are missing data in the worksheet. If the fishery is

transnational then weight the IEF scores of each country depending on the portion of total revenue that occurs in the country.

MACRO FACTORS

ECONOMIC CONDITION

Gross Domestic Product (GDP) Per Capita

RATIONALE:

Richer nations are more likely able to afford the institutions and technological factors that are necessary for effective management and sustainable generation of rents. Hilborn (2007b) asserts that the type of management institutions that are best suited to wealthy nations with strong central governments are likely to be very different from the decentralized strategies that perform better in small-scale fisheries located in developing countries.

METRIC:

Country's per capita GDP on a purchasing power parity basis. Bin boundaries based on 2010 quintile of the US CIA's rank of per capita GDP for all countries. Dollars are 2010 USD.

5	Greater than 30,000USD
4	Greater than 12,400USD
3	Greater than 6,000USD
2	Greater than 2,500USD
1	Less than 2,500USD

SCORING GUIDANCE:

Visit the website https://www.cia.gov/library/publications/the-world-factbook/rankorder/2004rank.html then find the country in the list. If the fishery is transnational then weight the GDP of each country depending on the portion of total revenue that occurs in the country.

PROPERTY RIGHTS & RESPONSIBILITY

FISHING ACCESS RIGHTS

Proportion of Harvest Managed Under Limited Access

RATIONALE:
Limited-access fisheries are an essential step in eliminating the open-access common property problem of rent dissipation (Clark, 1980).

METRIC:
The proportion of total harvest value that is under limited-access fishing regulation. This can include both regulatory and de facto access rights. Fisheries where there is a gatekeeper regulatory institution such as a beach management unit or a chief fisherman whom entrants must talk to or buy a permit from prior to gaining access count as limited access for the purposes of this metric.

5	Virtually all
4	70-95%
3	35-70%
2	5-35%
1	Virtually none

SCORING GUIDANCE:
Access rights are defined as the institutions that give harvesters the right of entry into the fishery; they are different from harvest rights that allow harvesters to claim a well-defined portion of total harvest. It is possible to have strong access rights without any guarantee of landings (e.g., Olympic fishery with a race to the fish) but it is unlikely that a system of quotas that assigns harvest rights will not also have access rights (typically quotas are assigned along with a permit to access the resource). Limited-access systems can be employed in many different ways with different levels of effectiveness. In a fishery where there is a gatekeeper who sells/distributes permits to access but distributes them without limits this will still count towards the percentage of total harvest under limited access but this fishery should score very low in the exclusivity of access rights metric. ITQ systems that also require permits in order to participate should be scored as having strong access rights as well as strong harvest rights. Fisheries where the entire catch is managed under the same regime are likely to score a 1 or 5 unless there is some bycatch from another open access fishery (as in Florida spiny lobster), competition from subsistence fishermen who are not subject to limited access arrangements, or illegal fishing that circumvents the limited access regulations. This references commercial activity, so recreational activity affecting the same stock does not influence the scoring (though it does affect Exclusivity below).

EXAMPLES:

- In a Ghanaian fishery one must be a member of the tribe in order to fish from the beaches; there is no formal limit to access because non-tribe members can buy access rights and the children of tribe members will inherit the right to access. Still count this as limited access but with low exclusivity. This fishery scored a 4 because there was some illegal fishing from non-tribe members who did not consult the chief fisherman.

- In the Florida Spiny lobster fishery there are trap tags which are a strong access right, but there is also some bycatch from the open access shrimp fishery so the fishery scored a 4.
- In the Alaska salmon fishery all entrants must purchase one of a limited number of permits and subsistence fishers have also been allocated permits, this fishery scored a 5.

PROPERTY RIGHTS & RESPONSIBILITY

FISHING ACCESS RIGHTS

Transferability Index

RATIONALE:
Transferability is essential for a functioning market to allocate resources to their best use. If rights are not transferable, financing is undermined because the property may not be accepted as collateral. If the markets for the rights are not efficient, then the value of the right will not be transparent, and its price will not necessarily reflect the value. This will lead to misallocation of resources and inefficiencies, as well as undermine sustainability and wealth creation (Anderson, 2007, 2002).

METRIC:
NA if no limited access gatekeeper, but it can be scored if there is even a nominal system for granting access rights.

5	Very Strong: Fully transferable through well-established, efficient market institutions
4	Strong: Fully transferable, but institutions are poor or illiquid
3	Moderate: Transferable, but with severe restrictions on who can hold, or how much
2	Weak: Transferable only under highly restricted and limited condition
1	Access rights not transferable

EXAMPLES:

- In the Alaska crab fishery, permits are allocated to harvesters who are free to trade with anyone so the score is a 5.
- In the Alaska salmon fishery, there are restrictions regarding the transfer of permits outside of certain regions and there are limits to how many one entity can hold so the score is a 3.
- In the artisanal fishery in Senegal, access is inherited and cannot be transferred to anyone other than the tribe member so the score is a 1.

PROPERTY RIGHTS & RESPONSIBILITY

FISHING ACCESS RIGHTS

Security Index

RATIONALE:
When property rights are insecure, regardless of whether the reason is crime, civil unrest, war, government instability or government's use of eminent domain, it causes owners to be more exploitive with resources. Uncertainty implicitly increases the discount rate and undermines financing (Anderson, 2007 and 2002).

METRIC:
Extent to which the government reduces or threatens to change the access rights. Even if no limited access, can be scored to reflect the extent of other restrictions that limit erosion of access rights (though the score will probably be low).

5 Very Strong: Access rights are completely respected by the government
4 Strong: Rights are mostly respected by the government; generally survive changes in government administration
3 Moderate: Rights are at risk of retraction with changes in administration
2 Weak: Rights are highly threatened or there is high political uncertainty
1 None: Access rights are not protected

SCORING GUIDANCE:
This metric is meant to reflect the resource users' beliefs about how secure their access rights are; this means whether they themselves expect to be able to continue to access the fish resources. This does not measure the exclusivity of the right, or how much they expect the government to dilute their right by allowing in other users. This metric is trying to get at whether they believe that they personally will be allowed to access, not whether they anticipate others intruding.

EXAMPLES:
- In the Alaska crab fishery, permit holders know that both the state and federal governments are stable and highly unlikely to revoke their access rights. They are also aware that they have the lobbying power to ensure that their access rights are secure. The fishery scored a 5.
- In the artisanal fishery of Liberia, official responsibility for fisheries management still resides at the national level. Recent civil warfare and unpredictable behavior by the central government means that local harvesting groups are unsure whether their right to access the fishery will be undermined by political unrest. This fishery scored a 2 due to the high degree of political uncertainty and lack of support from the central government.

PROPERTY RIGHTS & RESPONSIBILITY

FISHING ACCESS RIGHTS

Durability Index

RATIONALE:

Short-duration property rights create more exploitive management. This implicitly increases the discount rate, thus undermining sustainability and wealth creation (Anderson, 2007, 2002).

METRIC:

Duration of the property right. Even if no limited access, can be scored to reflect harvesters' expectations of continued access.

5	Very Strong: > 10 years to perpetuity
4	Strong: 6 to 10 years
3	Moderate: 1 to 5 years
2	Weak: Seasonal
1	None: None/daily

SCORING GUIDANCE AND EXAMPLES:

This metric is meant to reflect the resource users' *beliefs* about how long their access rights will endure. This metric is meant to capture the durability of the right in practice and not strictly legally.

- For example, if a fisherman in Indonesia has a one year license but knows that so long as he does not violate any regulations then he will be able to renew this license for the foreseeable future, then this should score a 5 and not a 3.
- If the access rights are renewable with reapplication and the harvesters expect to be able to continue to access then score based on these expectations. If users have an ostensible lifetime right but there is a civil war that leads to them feeling insecure, then they should score high in durability and low in security.
- In the Seychelles nearshore lobster fishery the annual licenses are allocated on a first-come first-served basis so there is no guarantee that the license will be renewed after the first year. This fishery scored a 3.

PROPERTY RIGHTS & RESPONSIBILITY

FISHING ACCESS RIGHTS

Flexibility Index

RATIONALE:

Under strong property rights, all decisions regarding use, management and technology employed are controlled by the owner. Placing the choice of fishing time, location, gear and handling practices within the owner's control should lead to more efficient choices, although if effort is limited by total allowable catch there may be overcapitalization relative to the efficient equilibrium (Wilen, 1979).

METRIC:

Ability of right holders to be flexible in the timing and production technology employed. Low scores will reflect restrictions that force inefficiencies. Even without limited access, there may still be scorable restrictions (gear, seasons, areas) that limit access flexibility.

5	Very Strong: All decisions on time of harvest, gear used and handling practices are in the owner's control
4	Strong: Minimal restrictions on time of harvest and technology
3	Moderate: Modest restrictions on time of harvest and technology
2	Weak: Significant restrictions on time of harvest and technology
1	Time of harvest, gear used and handling practices are not in the owner's control

SCORING GUIDANCE AND EXAMPLES:

This metric is meant to capture how flexible the regulations are in practice; not strictly what is legally recorded but whether the regulations are actually enforced.

- For example, there is a norm in Ghana that harvesters do not fish one day a week and are prohibited from using seine nets with small mesh. If the harvesters ignore these rules and fish whenever they want with whatever gear they choose then the fishery scores a 5, but if the rules are followed then it scores a 3.
- The emphasis is on regulations that force technical inefficiencies, i.e. if it is easier and cheaper to catch octopus with a spear gun but that technique is banned in order to help the octopus population recover then we would say that there are significant restrictions on the harvest technology.
- If the best fishing for Pollock is in an area of the continental shelf that is closed to habitat or bycatch reasons and there are additional restrictions on the type of gear and the timing of harvest then this scores a 2.

PROPERTY RIGHTS & RESPONSIBILITY

FISHING ACCESS RIGHTS

Exclusivity Index

RATIONALE:
Under strong property rights, all decisions and access to the property are controlled by the owner. With well-defined rights, externalities are internalized and net benefits are captured. Those that produce externalities that infringe on the property right are held responsible. If externalities are not internalized, costs are undervalued, market signals are biased, resources are misallocated and sustainability and wealth creation are undermined (Anderson, 2007, 2002).

METRIC:
Ability of right holders to exclude those who do not have the right from affecting the resource or market. Can still be scored to capture extent of de facto intrusion if access is not limited (should still score if the fishery is open access).

5	Very Strong: All decisions and access to the property are controlled by the right's owner. There are a limited amount of access rights granted and no intrusion from those without rights such as recreational/bycatch fisheries.
4	Strong: Little intrusion on resource by those without rights and there are a limited amount of access rights granted.
3	Moderate: Modest intrusion on resource by those without rights. There is some effort to restrict the amount of access rights distributed.
2	Weak: Significant intrusion on resource by those without rights or little limit on the amount of access rights distributed.
1	None: Completely unrestricted open access, despite putative right. No limit on the amount of access rights distributed.

SCORING GUIDANCE:
This metric is meant to measure both illegal intrusion by outsiders engaged in illegal or unregulated commercial fishing, or though excessive take beyond their traditional or allotted quantity by 1) commercial fishermen in targeting other species; 2) recreational fishermen; or 3) subsistence fishermen.

In fisheries where there is competition from recreational or subsistence fishers that fall outside the commercial fisheries' regulations, this would be sometimes be considered unregulated catch that should subtract from the exclusivity of limited access. If the recreational fishery falls within historical norms for the allocation of the recreational sector and has effective management then this does not decrease the score. However, if the recreational sector has no limit on the amount of fish they can land, or routinely exceeds the allocation for their sector, then this would lower the exclusivity of the access right.

EXAMPLES:

- This metric is meant to capture whether access rights are distributed with or without limits. If the chief fisherman in Ghana decides to allow anyone to fish so long as they buy

a permit from, or curry favor with him then this fishery is not exclusive and it should score a 1.

- In the chocolate clam fishery of Mexico there is modest competition from recreational users who are allowed to harvest from the beds closest to shore and are not granted a specific allocation or limited in the number of clams that they can harvest. Since this species typically lives in shallow beds, and the recreational fishery is not limited the exclusivity of access rights is lower although there are strict limits on the amount of permits that are distributed to commercial harvesters. The fishery scored a 4.
- In the Alaska salmon fishery, there are strict regulations in place for both recreational and subsistence users. Their catch mainly falls within the historic norm for the allocation of these sectors so the fishery scores a 4 and the score is lowered from a 5 due to small amounts of bycatch from other commercial fisheries such as pollock.

PROPERTY RIGHTS & RESPONSIBILITY

HARVEST RIGHTS

Proportion of Harvest Managed with Rights-based Management

RATIONALE:
Rights-based management that assigns a portion of the harvest to individuals or communities such as Individual/Community Quotas, catch shares or Territorial Use Rights (TURFs) over contained/sedentary species establish expectations of an exclusive right to a quantity of landings, and induce economic incentives to allocate resources efficiently and generate wealth (Clark, 1980, Branch, 2009, and Nowlis, 2012). In addition, they also appear to reduce risk and uncertainty within the fishery (Essington, 2010). Some researchers argue that such tenure systems are less effective in the face of production externalities (Boyce, 1992) and also lead to the exclusion of some community members who are then unable to smooth risk by participating in multiple occupations (Allison and Ellis, 2001).

METRIC:
The proportion of total harvest value that is under harvest rights-based fisheries management. Rights include those for some fixed quantity of fish (e.g., a quota), or a fixed share of landings in an area (e.g., a TURF gives 100% of landings in an area). A TURF does not give harvest rights unless the species harvested are sedentary or their movement is completely contained within as single right holder's territory. Rights can be held by individuals or communities, and can include de facto and de jure rights. (Input rights, like trap tags, are strong access rights, but not harvest rights included in this section.)

5	Virtually all
4	70-95%
3	35-70%
2	5-35%
1	Virtually none

SCORING GUIDANCE:
Rights-based systems can be employed in many different ways with different levels of effectiveness. In general, having the right to access the resource does not guarantee the right to harvest the resource due to competition with other holders of the access right. Individual rights over sedentary species and quotas for a set amount of landings of non-sedentary species are the primary forms of harvest rights in the database. Community quotas count as harvest rights; the remaining harvest right metrics should be scored for the individual within the community quota system.

EXAMPLES:

- For example, the Alaska salmon fishery has strong permit-based access rights but no harvest rights because there is no quota or TURF system in place.
- The Bangladesh floodplain fishery or Gambian oyster beds are an example of fisheries where TURFs grant harvest rights.
- In contrast, a TURF on the West coast of Africa does not grant harvest rights over migratory pelagic species and should not be considered a harvest right because the fish

are not guaranteed to be landed in that area. In a Ghanaian fishery you must be a member of the tribe in order to fish from the beaches; there are no formal harvest rights because the primary species targeted are migratory and the stock is not contained within the area that the tribe controls. This fishery scored a 1 for this metric, although it scored 4 in the existence of access rights.

- In the Baltic Sea cod fishery, the stock is shared by multiple management countries and Denmark is the only country to implement an ITQ system. Since Danish landings are around 40%, this fishery scores a 3.
- In the directed Alaska halibut fishery, all entrants must purchase quota and since both subsistence and recreational harvesters are carefully regulated, this fishery scored a 5.

Transferability Index

RATIONALE:
Transferability is essential for a functioning market to allocate resources to for their best use. If rights are not transferable, financing is undermined because the property may not be accepted as collateral. If the markets for the rights are not efficient, then the value of the right will not be transparent, and its price will not necessarily reflect the value. This will lead to misallocation of resources and inefficiencies, as well as undermine sustainability and wealth creation (Anderson, 2007, 2002). Arnason (2009) shows that competing resource users can attain an economically efficient allocation that maximizes social and environmental objectives as well if rights are fully transferable.

METRIC:
NA if there is no harvest right.

5	Very Strong: Fully transferable through well-established, efficient market institutions
4	Strong: Fully transferable, but institutions are poor or illiquid
3	Moderate: Transferable, but with severe restrictions on who can hold, or how much
2	Weak: Transferable only under highly restricted and limited conditions
1	Harvest rights not transferable

SCORING GUIDANCE:
Transferability is one area where fisheries with both access and harvest rights (permits and quota required to harvest) are likely to have different scores for access rights and harvest rights, since the regulations controlling transfer of permits are often very different from those that dictate the transfer of quota.

EXAMPLES:
- In the Alaska crab fishery, permits are allocated to harvesters who are free to trade with anyone so the score for access rights transferability is a 5, whereas quota cannot be transferred as freely and there are some consolidation restrictions leading to a score of 4.
- In the Suruga shrimp fishery in Japan, there is TURF that encompasses the habitat of this species so there are harvest rights. These rights belong to the harvesting coops and not to the individual harvesters and since transfer to non-members is outlawed the fishery scored a 2.

PROPERTY RIGHTS & RESPONSIBILITY

HARVEST RIGHTS

Security Index

RATIONALE:
When property rights are insecure, regardless of whether the reason is crime, civil unrest, war, government instability or government's use of eminent domain, it causes owners to be more exploitive with resources. Uncertainty implicitly increases the discount rate and undermines financing (Anderson, 2007 and 2002).

METRIC:
Extent to which the government threatens to reduce or eliminate the harvest rights. NA if there is no harvest right.

5	Very Strong: Harvest rights are completely respected by the government
4	Strong: Rights are mostly respected by the government and generally survive changes in government administration
3	Moderate: Rights are at risk of retraction with changes in administration
2	Weak: Rights are highly threatened or there is high political uncertainty
1	None: Harvest rights are not protected

SCORING GUIDANCE:
This metric is meant to reflect the resource users' beliefs about how secure their harvest rights are; this means whether they themselves expect to be able to continue to harvest the fish resources. This does not measure the exclusivity of the right, or how much they expect the government to dilute their right. This metric is trying to get at whether they believe that they personally will be allowed to harvest, not whether they anticipate others intruding.

EXAMPLES:
- In the Alaska Pollock fishery, quota holders know that both the state and federal governments are stable and highly unlikely to revoke their harvest rights. They are also aware that they have the lobbying power to ensure that their access rights are secure. The fishery scored a 5.
- In the chocolate clam fishery of Mexico, harvesters feel secure in their right to access the fishery and do not fear that the government will retract their access rights so they scored a 5 for security of access rights. However, their quota is allocated on a seasonal basis and is less secure than their right of access; there is some minor concern that the quota allocations could shift with changes in government administration, so the security of harvest rights is just strong, a 4.

PROPERTY RIGHTS & RESPONSIBILITY

HARVEST RIGHTS

Durability Index

RATIONALE:
Short-duration property rights create more exploitive management. This implicitly increases the discount rate, thus undermining sustainability and wealth creation (Anderson, 2007 and 2002).

METRIC:
Duration of the harvest right. NA if there is no harvest right.

5	Very Strong: > 10 years to perpetuity
4	Strong: 6 to 10 years
3	Moderate: 1 to 5 years
2	Weak: Seasonal
1	None: None/daily

SCORING GUIDANCE AND EXAMPLES:
This metric is meant to reflect the resource users' *beliefs* about how long their harvest rights will endure, the durability of the right in practice and not strictly legally.

- For example, if a halibut fisherman in Alaska has a one year quota but knows that so long as he does not violate any regulations then he will be able to renew this quota for the foreseeable future then this should score a 5 and not a 3.
- If the harvest rights are renewable with reapplication and the harvesters expect to be able to continue to harvest then score based on these expectations. However, the durability scores for harvest may be lower than for access rights since in many quota systems the individual amount of quota given out depends on the overall TAC which may change from year to year.

PROPERTY RIGHTS & RESPONSIBILITY

HARVEST RIGHTS

Flexibility Index

RATIONALE:
Under strong property rights all decisions regarding use, management and technology employed to extract value from the property are controlled by the owner. Fishing time, location, gear and handling practices are in the owner's control. Some claim that (otherwise unrestricted) harvest rights are the most flexible and therefore the most successfully adaptive management tool (Moloney and Pearse, 1979).

METRIC:
Ability of right holders to be flexible in the timing and production technology employed. NA if there is no harvest right.

5	Very Strong: All decisions on time of harvest, gear used and handling practices are in the owner's control
4	Strong: Minimal restrictions on time of harvest and technology
3	Moderate: Modest restrictions on time of harvest and technology
2	Weak: Significant restrictions on time of harvest and technology
1	Time of harvest, gear used and handling practices are not in the owner's control

SCORING GUIDANCE AND EXAMPLES:
This metric is meant to capture how flexible the regulations are in practice; not strictly what is legally recorded but whether the regulations are actually enforced. For example, there is a norm in Ghana that harvesters do not fish one day a week and are prohibited from using seine nets with small mesh. If the harvesters ignore these rules and fish whenever they want with whatever gear they choose then the fishery scores a 5, but if the rules are followed then it scores a 3. The emphasis is on regulations that force technical inefficiencies, i.e. if it is easier and cheaper to catch octopus with a spear gun but that technique is banned in order to help the octopus population recover then we would say that there are significant restrictions on the harvest technology.

The flexibility score for harvest rights is likely equal to the access rights flexibility score unless there is a multispecies quota fishery with a choke species. In that case, you could have the flexibility to access the resource and use any type of gear, but you would only be able to harvest until your quota for the choke species ran out. This means that the flexibility of the harvest right is lower.

PROPERTY RIGHTS & RESPONSIBILITY

HARVEST RIGHTS

Exclusivity Index

RATIONALE:
Under strong property rights all decisions and access to the property are controlled by the owner. With well-defined rights, externalities are internalized and net benefits are captured. Those that produce externalities that infringe on the property right are held responsible. If externalities are not internalized, costs are undervalued, market signals are biased, resources are misallocated and sustainability and wealth creation are undermined (Anderson, 2007, 2002).

METRIC:
Ability of right holders to exclude those who do not have the right from affecting the resource or market. NA if there is no harvest right.

5	Very Strong: Management prevents harvest in excess of rights allocation; no intrusion by outsiders
4	Strong: Management allows little harvest in excess of allocation; little intrusion by those without rights
3	Moderate: Modest harvest in excess of rights allocation; modest intrusion on resource by those without rights
2	Weak: Harvest in excess of rights allocation significantly affects resource or markets; significant intrusion on resource by those without rights
1	None: Completely unrestricted open access, despite putative right

SCORING GUIDANCE:
This metric is meant to measure both illegal intrusion by outsiders through illegal or unregulated commercial fishing, or through excessive take beyond their allotted quantity by 1) commercial fishermen in targeting other species; 2) recreational fishermen; or 3) subsistence fishermen. It is also meant to capture whether harvest rights are distributed with or without limits. If the regulators decided to allocate an unlimited or excessive amount of harvest quota such that the harvesters were not assured that they would be able to harvest the full amount of the resource that they were allocated then this would not be an exclusive right. Rights that are diluted through over-allocation should score low in exclusivity.

EXAMPLE:

- In the Mexican Chocolate clam fishery, harvesters are required to have quota when harvesting clams, but in practice they often harvest up to 50% more than their allocation and there is no enforcement. In addition, harvesters are responsible for the stock assessments that set the annual TAC so they have an incentive to manipulate these figures which further reduces exclusivity. This fishery scored a 2.

CO-MANAGEMENT
COLLECTIVE ACTION

Proportion of Harvesters in Industry Organizations

RATIONALE:
The degree to which producers are organized into cooperatives or associations that can act collectively to influence distribution/sharing of resources and facilitate both buying and selling power, which will theoretically contribute to more effective management of the resource than centralized top-down control by agents distant from the fishery (Jentoft, 1998 and Jentoft and McCay, 1995).

METRIC:
Proportion of harvest where the primary harvesters consider themselves to be members of organized associations. This captures whether the harvesters are organized to influence outcomes, and thus can include organization along company lines in industrialized fisheries.

5	Virtually all
4	70-95%
3	35-70%%
2	5-35%
1	Virtually none

SCORING GUIDANCE:
This metric measures whether or not harvesters are involved in organizations that are capable of influencing management or coordinating business transactions. The next two scores indicate how effective these organizations are at influencing management or coordinating joint business arrangements. It may be difficult to distinguish between associations that are focused on joint marketing or management collective action, but it should be possible to get an idea of harvester organizations' goals if direct meetings are arranged.

EXAMPLES:
- If Liberian fishermen are very active in their harvester cooperative and they frequently buy fuel together but do not attempt to influence management practices then this fishery should score high in this metric and in the business influence metric but low in management influence.
- In the Alaska Pollock fishery, where many vessels and access and harvest rights are owned by companies, individual captains or crew may not be members of a single association, but companies have employees whose job it is to participate in the industry associations. Since the company is the decision-making unit about how to fish, this scores a 5.

CO-MANAGEMENT

COLLECTIVE ACTION

Harvester Organization Influence on Management & Access

RATIONALE:
Harvesting organizations can influence management and access by directly managing access rights (e.g., cooperatives or community quota systems) or by taking political action to influence the access they and others have through the management authority. Such harvester participation may facilitate management that increases wealth accumulation to harvesters. The literature on co-management asserts that harvesters possess greater specialized knowledge of the fishery that allows them to make effective and equitable contributions to management plans (McCay and Jentoft, 1996). In addition, harvesters who are more involved in creating regulation have been shown to be more likely to comply with the regulations (Pomeroy and Berkes, 1997).

METRIC:
Qualitative metric of how much influence harvesting organizations have, either directly or through political collective action, on management and access to the fishery:

5	Harvester organizations determine allocation of resources
4	Harvester organizations have significant influence in determining allocation
3	Harvester organizations are politically active, but not controlling
2	Harvester organizations conduct social or informal monitoring of participation and allocation
1	Harvester organizations make no active effort or have no capacity to influence management

SCORING GUIDANCE:
This measures how effective harvester organizations are at influencing de facto management. It may be difficult to distinguish between associations that are focused on joint marketing or management collective action, but it should be possible to get an idea of harvester organizations' goals if direct meetings are arranged.

EXAMPLES:
- If Liberian fishermen are very active in their harvester cooperative and they determine who has access to the beach then they score highly in this metric even though they have no say in the central government's policy.
- Japanese harvesting cooperatives have a long history of being extremely active in lobbying the central government for increased allocation and effectively determining the allocation of resources within their TURFs so the score is a 5.

CO-MANAGEMENT
COLLECTIVE ACTION

Harvester Organization Influence on Business & Marketing

RATIONALE:
Harvesting organizations can influence business and marketing by working to exert market power in either purchasing of inputs (e.g., marine services or insurance), adopting prices that improve market value, or by collectively marketing products, in order to reduce costs or increase revenue. Such joint activity may increase wealth accumulation to harvesters. (Jentoft, 1985)

METRIC:
Qualitative metric of how much influence harvesting organizations have, either directly or through political collective action, on business operations and marketing in the fishery.

5	Harvesting organizations cooperatively determine marketing and operational details
4	Extensive joint marketing
3	Large subgroups facilitating marketing; joint purchasing
2	Small subgroups cooperating in purchasing or operations
1	No active effort or capacity to influence business operations

SCORING GUIDANCE:
This measures how effective harvester organizations are at conducting joint business or marketing. It may be difficult to distinguish between associations that are focused on joint marketing or management collective action, but it should be possible to get an idea of harvester organizations' goals if direct meetings are arranged.

EXAMPLES:
- Suppose that Ghanaian fishermen are very active in their harvester cooperative and they collectively bargain with processing companies to set ex-vessel prices; they should score highly in this metric even if there is no advertising in the fishery.
- In the New England groundfish fishery the fishermen's associations are politically active in generating ideas for alternative management so they scored a 3 in the previous metric. However, prices and marketing continue to be done through bilateral relationships between individual captains and dealers so they only score a 1 for this metric.

CO-MANAGEMENT
PARTICIPATION

Days in Stakeholder Meetings

RATIONALE:
This metric is a proxy for the efficiency of the management process and stakeholder participation. Stakeholder participation injects stakeholders' knowledge into management, and may increase legitimacy and compliance (Jentoft, 1998 and Jentoft and McCay, 1995). However, it may also increase management costs and present opportunities for lobbying and rent seeking that increases the time required to implement management, or weakens implemented regulations to prevent wealth generation (Bennett et al., 2001).

METRIC:
Days in stakeholder meetings per year spent by a participant in the fishery who is active in management. Note these are days with meetings, not FTE days. Include meetings of councils with public participation.

5	More than 24 per year
4	12-24
3	6-11
2	1-5
1	None

SCORING GUIDANCE:
This includes time in meetings of harvester organizations directed at management, as well as at private and public meetings of management bodies themselves. The meetings with management must provide an opportunity for stakeholder input, and not simply be public hearings viewable by stakeholders. The metric refers to stakeholders who are active in management, who probably attend more days than the average stakeholder.

EXAMPLES:
- In the Alaska Pollock fishery, stakeholders who are active in management spend an average of 30 days in formal council meetings or at informal meetings between processor organizations and fisheries management staff so the fishery scored a 5.
- In the artisanal fishery of Ghana, stakeholders meet only once per year with national officials responsible for fisheries policy and organize their own local meetings three times per year so the score is a 2.

CO- MANAGEMENT
PARTICIPATION

Industry Financial Support for Management

RATIONALE:
If the industry pays for the cost of management, it is likely that efficiency will be improved and the concomitant control over management exerted by the industry will lead to improved outcomes for harvesters, especially wealth generation. Some researchers claim that user participation is a key determinant of whether a management system generates equity, resilience, efficiency, and stewardship (Hanna, 1995).

METRIC:
Proportion of the fishery management budget paid for by the harvesting or processing sector.

5	Virtually all
4	50-95%
3	5-50%
2	1-5%
1	None

SCORING GUIDANCE:
This is currently uncommon, but in some fisheries, industry supports research and/or management costs. This does not include money from development agencies or NGOs that are more closely aligned with government than the industry.

EXAMPLES:
- In the Alaska crab fishery it was estimated that a minority of the fisheries management budget comes from permit/quota fees paid by harvesters and processors. Since this is the only contribution that they make to management budgets, the fishery scored a 3 with a quality score of B since the scorer was unsure of the exact percentage but knew it was neither 0 nor 100%.
- In the chocolate clam fishery of Mexico, harvesters are responsible for conducting their own stock assessments and since enforcement is minimal it was estimated that these costs represent the majority of the fisheries management budget. The fishery scored a 4 with a quality score of B since the scorer was sure that the percentage was greater than 5%.

CO- MANAGEMENT

COMMUNITY

Leadership

RATIONALE:

There is empirical and theoretical evidence that strong community leadership can alleviate common property dilemmas (Gutiérrez et al., 2011 and Ostrom, 1990). Researchers hypothesize that prominent community leaders are particularly important in situations where central governments have limited control and may be responsible for successfully managing aquatic resources and securing the livelihoods of communities depending on them.

METRIC:

Qualitative metric of whether the fishing community has strong leadership capable of envisioning and implementing effective management (this role may be provided by processors). Bins 2 and 4 may be scored as midpoints between descriptions.

5 Widely recognized individual leader, or small group of individual leaders, who provides vision for management and is able to attract stakeholders to that vision

3 Ex officio leadership stations that maintain management institutions, but are not currently providing strong vision

1 No recognized leader providing vision for fishery stakeholders

SCORING GUIDANCE:

Most fisheries with or without formal organizations have someone whose job it is to be a leader. This person may be the leader, but the individual may not hold a formal post. This metric captures the effectiveness of that leader at catalyzing change for the better.

EXAMPLES:

- In the Alaska crab fishery there is a small group of leaders who represent the harvest and post-harvest sectors at management meetings and are able to attract stakeholders to their vision. These leaders were instrumental in the implementation of a quota system so the fishery scored a 5.
- In the bivalve fishery of La Paz, Mexico, the heads of cooperatives serve as leaders who lead the efforts to educate illegal harvesters and ensure that the fishery is managed sustainably. This fishery scored a 4 because while there is a vision for improving the fishery that is held by the leadership it was thought that their ability to spread that vision and attract stakeholders could be improved and does not yet extend to the post-harvest sector.

CO- MANAGEMENT

COMMUNITY

Social Cohesion

RATIONALE:
There is empirical and theoretical evidence that strong social cohesion can alleviate common property dilemmas by helping participants coordinate mutually beneficial solutions (Gutiérrez et al., 2011 and Ostrom, 1990). Researchers hypothesize that social cohesion is particularly important in situations where central governments have limited control over regulations and may be responsible for successfully managing aquatic resources and securing the livelihoods of communities depending on them.

METRIC:
This metric measures whether the resource users are socially connected and interact regularly in fishing and non-fishing spheres. Score one point for each of the following:

- Common locations for gathering and meeting on a regular basis for non-fishery business, culture or commerce
- Presence of shared social norms that facilitate transactional trust
- Presence of shared public institutions (government, schools, markets)
- Absence of differences in social status or caste that prevent interaction
- Absence of religious differences and/or conflict
- Absence of cultural, ethnic or tribal differences that obstruct interaction

5	6 points
4	5 points
3	3-4 points
2	1-2 points
1	0 points

SCORING GUIDANCE:
Please be sure to note the social cohesion attributes that were present in the fishery within the FPI worksheet.

EXAMPLES:

- In the pole and line skipjack tuna fishery in the Maldives, the stakeholders are culturally homogenous so there are a lack of cultural/ethnic/tribal differences, the presence of shared social norms, a lack of religious differences, common locations for gathering and the presence of shared public institutions. Since there are differences in social status or caste that prevent interaction and owners do not routinely socialize with the laborers this fishery scored a 4 instead of a 5.
- In the New England groundfish fishery there is a noticeable lack of social cohesion and antagonism between stakeholders. The fishery scored a 2 because while there are shared public institutions and common locations for gathering the remaining attributes of social cohesion are missing.

CO- MANAGEMENT
GENDER

Business Management Influence

RATIONALE:
Development practitioners are interested in examining the role of women within the fishery, especially with regards to implications that gender equality may have for the performance of the community within the harvest and post-harvest sectors. There is some evidence that the mechanization and commercialization of small-scale fish traders leads to women being marginalized in the fishery (Hapke, 2011), and these input variables will allow testing of that hypothesis. Others conjecture that management plans that exclude women are ignoring the full scope of resource threats and prone to create conflict through the marginalization and exclusion of the female half of the local population (Diamond et al., 2003).

METRIC:
Extent of women's influence (not just participation) in the management of harvesting and post-harvest businesses, including decision-making, ownership and financing. This will not typically include development project staff or other "outsiders." Bins 2 and 4 may be scored as midpoints between descriptions.

5	Business management dominated by women
3	Business management is balanced between women and men
1	Business management dominated by men

EXAMPLES:
- In the artisanal fishery of Ghana, the women dominate the post-harvest sector but are not directly involved in the harvest sector, although there was some speculation that they influence their harvester husbands' business decisions. This fishery scored a 4 because of the strength of women's role in the post-harvest sector; there is a Fish Mommy who sets the daily ex-vessel price for the entire beach and has a large influence over both sectors.
- In the Alaska crab fishery, the vast majority of harvesters and processors are male and they are also fishing from remote locations far from the influence of their wives and other female family members. This fishery scored a 1 because it was not thought that women had much influence on business decisions.

CO- MANAGEMENT
GENDER

Resource Management Influence

RATIONALE:
Development practitioners are interested in examining the role of women within the fishery, especially with regards to implications that gender equality may have for the performance of the community within the harvest and post-harvest sectors. There is some evidence that the mechanization and commercialization of small-scale fish traders leads to women being marginalized in the fishery (Hapke, 2011) and these input variables will allow testing of that hypothesis. Others conjecture that management plans that exclude women are ignoring the full scope of resource threats and prone to create conflict through the marginalization and exclusion of the female half of the local population (Diamond et al., 2003).

METRIC:
Extent of women's influence (not just participation) in the management of the resource, including scientific and resource access and allocation decisions. Influential people can be members of the harvesting or post-harvest sectors, scientists, or community members who do not work in the fishing sector. This will not typically include development project staff or other "outsiders." Bins 2 and 4 may be scored as midpoints between descriptions.

5	Resource management dominated by women
3	Resource management is balanced between women and men
1	Resource management dominated by men

EXAMPLE:
- In the artisanal fishery of Ghana, the women dominate the post-harvest sector but are not directly involved in the harvest sector and thus do not play a role in exploitation level, allocation or access decisions. This fishery scored a 1 because despite the strength of women's role in the post-harvest sector, the Chief Fisherman is the one who determines how many people can fish from the beach and enforces size/gear regulations.

CO- MANAGEMENT

GENDER

Labor Participation in Harvest Sector

RATIONALE:

Development practitioners are interested in examining the role of women within the fishery, especially with regards to implications that gender equality may have for the performance of the community within the harvest and post-harvest sectors. There is some evidence that the mechanization and commercialization of small-scale fish traders leads to women being marginalized in the fishery (Hapke, 2011) and these input variables will allow testing of that hypothesis. Others conjecture that management plans that exclude women are ignoring the full scope of resource threats and prone to create conflict through the marginalization and exclusion of the female half of the local population (Diamond et al., 2003).

METRIC:

Proportion of those involved in the harvest sector labor pool, either as captains or crew who are women.

5	80-100% are women
4	60-80% are women
3	40-60% are women
2	20-40% are women
1	Less than 20% are women

EXAMPLES:

- The oyster fishery of Gambia is an exception to the global norm in fisheries. This fishery scored a 5 for this metric since nearly all the harvesters are women.
- The Alaska salmon fishery scored a 1 because there are very few captains or crew who are women.

CO- MANAGEMENT
GENDER

Labor Participation in Post-Harvest Sector

RATIONALE:
Development practitioners are interested in examining the role of women within the fishery, especially with regards to implications that gender equality may have for the performance of the community within the harvest and post-harvest sectors. There is some evidence that the mechanization and commercialization of small-scale fish traders leads to women being marginalized in the fishery (Hapke, 2011) and these input variables will allow testing of that hypothesis. Others conjecture that management plans that exclude women are ignoring the full scope of resource threats and prone to create conflict through the marginalization and exclusion of the female half of the local population (Diamond et al., 2003).

METRIC:
Proportion of those involved in the post-harvest sector labor pool, as buyers, sellers, managers, or workers who are women.

5	80-100% are women
4	60-80% are women
3	40-60% are women
2	20-40% are women
1	Less than 20% are women

EXAMPLES:
- The artisanal fishery of Ghana scored a 4 because almost all local processors are women but there are some traders and middlemen who are male.
- The Alaska pollock fishery scored a 1 because while there are some workers in the processing plants who are female, it is still less than 20% which can probably be attributed to the plants' remote locations.

MANAGEMENT

MANAGEMENT INPUTS

Management Expenditure to Value of Harvest

RATIONALE:
This is a measure of the cost of fisheries management in proportion to the value of fisheries. Efficiency in management is essential for ensuring that human well-being is being properly aligned with ecosystem health. It has been argued that rights-based management systems such as ITQs are effective at self-regulating and minimizing management expenditures (Arnason, 2007)

METRIC:
Government, industry, and aid agency expenditures on fishery management activities including research, enforcement, and management capacity development (but not infrastructure) relative to the ex-vessel value of the harvest.

5	Less than 5% of ex-vessel harvest value
4	5-25%
3	25-50%
2	50-100%
1	More than the ex-vessel harvest value

EXAMPLE:
- In the Ecuadorian purse seine tuna fishery, the ex-vessel value of the harvest is estimated to be between $1.5 and 3 billion USD and the proposed budget for management was around $6.7 million USD so the ratio is at most 0.5% and the fishery scored a 5. Although the management budget estimate was imprecise it would need to be about 10 times higher to change the bin so the quality score is an A.

MANAGEMENT

MANAGEMENT INPUTS

Enforcement Capability

RATIONALE:
There is empirical and theoretical evidence that poorly enforced management is ineffective at resolving the common property problem in fisheries (Sutinen and Andersen, 1985 and Sutinen et al. 1989). Researchers hypothesize that management with the capacity to strictly enforce regulation is essential for well-managed ecosystems, efficient use of resources, and community well-being.

METRIC:
Enforcement capacity includes that of the government, fishing organization or any other group that can effectively enforce management.

5	Strong capacity to enforce regulations for entire coastline, both nearshore and offshore
4	Capacity to enforce regulations for nearshore, but limited offshore
3	Capacity to enforce nearshore in most of the ports, very limited capacity offshore;
2	Capacity to enforce only in major ports, minimal effective capacity offshore
1	No capacity to enforce

SCORING GUIDANCE:
Different fisheries, with different management systems and different opportunities to subvert rules, have different enforcement needs. Although the most common case is related to space, and thus described in the scoring metric, the scoring should be based on how effectively enforcement capacity meets enforcement needs.

EXAMPLES:
- The Alaska pollock fishery has 200% observer coverage, meaning that every instance of salmon bycatch will be reported and there is no way for the harvesters to not report their bycatch, even offshore, so the fishery scored a 5.
- Within the Indonesian artisanal longline tuna fishery there is no capacity to enforce regulations and there is widespread concern over illegal fishing by Filipino vessels in the Banda Sea so the fishery scored a 1.

MANAGEMENT

MANAGEMENT INPUTS

Management Jurisdiction

RATIONALE:
There is evidence that the effective management of shared fish stocks is an important challenge for achieving long-term sustainable fisheries (e.g., Munro, 2004). Researchers hypothesize that ineffective management regimes have externalities for bordering fisheries with shared stocks and the coordination of fishery policy is critical to the success of management.

METRIC:
Extent to which the life cycle or range of a stock can be managed under a single coordinated plan, or through which ineffective management in one jurisdiction can undermine efforts in another.

5	Stock's life cycle is within a single management jurisdiction, or multiple jurisdictions have an effective, formal system for joint management throughout the range
4	Effective coordination institution facilitates joint management throughout the region of primary importance
3	There is a coordination structure, but it does not have binding authority
2	Informal institutions for coordinating management
1	Jurisdictions effectively manage the same stock independently

EXAMPLES:

- Management of the Alaska halibut fishery is coordinated by the US and Canada through by the International Pacific Halibut Commission so that there is an effective and formal system for joint management throughout the stock’s life cycle range and the fishery scores a 5.
- On Lake Victoria the Nile perch fishery is shared by Tanzania, Kenya, and Uganda but the three countries do not make any significant efforts to coordinate management plans so the fishery scored a 1.

MANAGEMENT

MANAGEMENT INPUTS

Level of Subsidies

RATIONALE:

Subsidies distort resource allocation and pricing and may increase effort levels at the expense of resource health (Clark et al., 2005). Lower subsidies are indicative of greater market efficiency and reduced overcapacity (Pauly et al., 2002). Subsidies include: preferential tax rates, input cost reductions, price supports, special borrowing rates, undervaluing resources (ex., leases), payments-in-kind and other related actions giving preference to groups of harvesters or processors.

METRIC:

Receive one point each for four key categories of "bad" subsidies:

- Fuel subsidies (not including reduced highways taxes)
- Fish access payment subsidies
- Capital or capital loan subsidies
- Price support (through inputs or direct payments).

5	No subsidies
4	1 subsidy category
3	2 subsidy categories
2	3 subsidy categories
1	4 subsidy categories

EXAMPLES:

- The Alaska salmon fishery does not receive any of the named subsidies so the fishery scored a 5.
- In the pole and line skipjack tuna fishery in the Maldives, the government sometimes builds boats (capital subsidy), there is a price minimum (price support) and there are fuel subsidies so the fishery scored a 2.

MANAGEMENT
DATA

Data Availability

RATIONALE:
Most researchers agree that a fishery management program will be more effective in achieving its social and biological goals if it collects data on which to evaluate policy changes, either retrospectively or prospectively (Zeller et al., 2005 and Walters and Hilborn, 1976). Some argue that the process of data collection can facilitate cooperation amongst stakeholders (Kaplan and McCay, 2004) while others assert that due to the infeasibility of universal fisheries data collection, it is better to design data-less management strategies (Johannes, 1998).

METRIC:

5	Annual (or other appropriate period) sampling for stock assessment, landings and economic data available
4	Consistently collected and comprehensive landings and price data available
3	Limited reliable landings or price data available; data irregularly collected or based on large samples
2	Available data based on small samples, or missing data, significantly impedes making inferences needed for management
1	No data is centrally collected

EXAMPLES:

- The Alaska salmon fishery has extensive escapement counting and stock assessment modeling each year and there is also individual-level fish ticket data that tracks all landings so the fishery scored a 5.
- In the artisanal fishery of Liberia, there is some effort by local and national agencies to keep track of landings but there is no capacity for sophisticated stock assessments, few scales available at landing sites, not enough management personnel for full coverage, and very little effort to track economic data such as prices so the fishery scored a 2.

MANAGEMENT
DATA

Data Analysis

RATIONALE:
Most researchers agree that a fishery management program will be more effective in achieving its social and biological goals if it practices adaptive management and analyzes data to evaluate policy changes, either retrospectively or prospectively (Zeller et al., 2005 and Walters and Hilborn, 1976). Some argue that the process of data collection can facilitate cooperation amongst stakeholders (Kaplan and McCay, 2004) while others assert that due to the infeasibility of universal fisheries data collection, it is better to design data-less management strategies (Johannes, 1998).

METRIC:

5	Biological and economic data used in prospective analysis of management
4	Biological data dominates simple prospective analysis
3	Biological or economic data is used to track performance retrospectively
2	Data is used inconsistently or irregularly
1	No data analysis conducted in management process

EXAMPLES:
- The Alaska pollock fishery utilizes detailed stock surveys and fishery dependent data in state-of-the-art stock assessment models to set allowable biological catch, and market models of demand for Pollock and other whitefish influence harvesters decisions, so the fishery scored a 5.
- In the artisanal fishery of Liberia there is some effort at the national level to retrospectively estimate landings over time but this is done inconsistently and so the fishery scored a 2.

MANAGEMENT

MANAGEMENT METHODS

MPAs and Sanctuaries

RATIONALE:

There is empirical and theoretical evidence (Walters, 2000 and Sumaila et al., 2000) that marine protected areas are a useful management tool for limiting the ecosystem effects of fishing, including biological and socio-economic impacts. Certain models and case studies suggest that the establishment of MPAs can mitigate overfishing while others assert that this management technique merely induces increased effort at the periphery of the sanctuary and is useful only with sedentary or multi-species stocks (Hilborn et al., 2004). Inclusion of this metric will allow for tests of both hypotheses.

METRIC:

Percentage of area used in species life cycle where fishing is closed or highly restricted. Include total area under rolling or seasonal closures.

5	More than 25%
4	10-25%
3	5-10%
2	Less than 5%
1	None

EXAMPLE:

- The Alaska pollock fishery has some area closures to prevent salmon bycatch and to protect Steller sea lions. It was estimated that these represented approximately 5-10% of the Bering Sea so the fishery scored a 3.

MANAGEMENT

MANAGEMENT METHODS

Spatial Management

RATIONALE:
Empirical and theoretical literature suggest that spatial management techniques such as TURFs can increase the productivity and profitability of a spatially heterogeneous multispecies fishery (Holland, 2003 and Sanchirico and Wilen, 2005). This metric will allow for tests of whether TURFs/zones without MPAs are an effective management tool.

METRIC:
Proportion of fishing ground managed through either direct control by TURF or designated community management regions, or through indirect control by limiting access points (launch or landing sites).

5	75-100%
4	50-75%
3	25-50%
2	Less than 25%
1	None

EXAMPLES:
- Most Japanese fisheries are managed through TURFs, giving local harvesting cooperatives the authority to manage the fishing within their area. Both the Suruga pink shrimp fishery and the Tokyo Bay trawl fishery scored a 5.
- In the artisanal fishery of Liberia there was a TURF recently established for one region where authority to control access was devolved to the local community management association but this is less than 25% of the total portion of the fishing grounds so this fishery scored a 2.
- Although the Alaska Pollock fishery has some spatial closures for ecosystem and bycatch reasons, these are captured in the above metric. Since quota does not have a spatial designation this fishery scored a 1.

MANAGEMENT

MANAGEMENT METHODS

Fishing Mortality Limits

RATIONALE:
There is conflicting evidence on the effectiveness of total allowable catch limits that are not accompanied by limited access or harvest rights (Daan, 1997 and Copes, 1986). Further study of which types of management regimes are enhanced by the implementation of TAC and what circumstances help a TAC function well is necessary.

METRIC:
Extent to which fishing mortality is an explicit instrument of management.

5 Hard TAC established against which nearly all fishing mortality is counted
4 Hard TAC established, but there are sources of unaccounted mortality totaling less than 10%; or TAC is adjusted from biological guideline to compensate for sources of greater unaccounted mortality
3 There is a guideline mortality level that is generally met; hard TAC exceeded 10-50% by unaccounted mortality
2 Frequently exceeded guideline; hard TAC exceeded by more than 50%
1 Fishery does not have an explicit mortality target

EXAMPLES:
- The Alaska pollock fishery has a TAC with 200% observer coverage (two onboard observers working 12 hour shifts) so nearly all fishing mortality is accounted for and the fishery is closed once all harvesters have met their quota and the TAC is attained so this fishery scored a 5.
- On Lake Victoria there is no TAC and landings are infrequently counted; the fishery scored a 1 since the management strategy does not include an explicit mortality target.
- In the chocolate clam fishery of Mexico there is a hard TAC but harvesters are responsible for self-reporting their landings and there are no impartial observers. It is estimated that they exceed their allocation by approximately 20-40% so the fishery scored a 3.

POST-HARVEST

MARKETS & MARKET INSTITUTIONS

Landings Pricing System

RATIONALE:
Fair and efficient price discovery systems are essential for efficient resource use and wealth creation (Jensen, 2007 and Kaplan, 2000). Crucial to this is the ability of harvesters to move among ex-vessel buyers to those offering the best prices on a per-landing basis.

METRIC:
Proportion of the harvest sold in a transparent daily competitive pricing mechanism, such as an auction or centralized ex-vessel to wholesale market wherein sellers interact with many buyers and prices are public information.

5	Virtually all
4	70-95%
3	35-70%
2	5-35%
1	Virtually none

EXAMPLES:

- In some freshwater artisanal fisheries outside of Dhaka, Bangladesh, all landings are sold through a display auction with an auctioneer from the community and the number of buyers ranges from 20 to 200 so each fishery scored a 5.
- In the pole and line skipjack tuna fishery in the Maldives, the prices given by the processors are effectively posted offer and although the harvesters can call around to get them easily there is no auction or centralized market and prices are not negotiated within a framework of competition and public information so the fishery scored a 1.

Post-Harvest

Markets & Market Institutions

Availability of Ex-vessel Price & Quantity Information

Rationale:

Market transparency is essential for efficient resource use and wealth creation. Market transparency is characterized by readily available, accurate price and quantity information. Fair and efficient price discovery systems are essential for efficient resource use and wealth creation (Jensen, 2007 and Kaplan, 2000).

Metric:

Scores the ability of the market to provide timely information to harvesters to which they can react by changing what or when they land.

5	Complete, accurate price and quantity information available to market participants immediately
4	Reliable price and quantity information is available prior to the next market clearing
3	Price information is available but no timely quantity information
2	Price and quantity information are inaccurate, lagged or available to only a few
1	No information available

Examples:

- In the Indian Ocean distant water purse seine tuna fishery, the EU fleet has the ability to switch between zones and land their harvest based on current market prices so the fishery scored a 4. It did not score a 5 because the information is not available instantaneously and boats can’t get to alternate landing sites before repricing.
- In the Florida spiny lobster fishery, the price and quantity information are lagged by a few months and this inhibits the ability of harvesters to land their catch within the optimal market so the fishery scored a 2.

POST-HARVEST
MARKETS & MARKET INSTITUTIONS

Number of Buyers

RATIONALE:
This metric is an indicator of relative market power. If the market is dominated by a single (or very few) buyers or sellers, price will favor the side with greater market power. As Adelaja et al. (1998) demonstrate, there is evidence that when property rights are allocated, the number of buyers in the fishery will decline drastically and there is a possibility that monopoly power will skew the distribution of benefits.

METRIC:
Typical number of buyers of ex-vessel product accessible to a seller in a given market. If there are many landing sites, this is the buyers per landing site. If harvesters are generally indentured to a single buyer through credit relationships, there is one buyer.

5	Highly competitive
4	4-6 buyers
3	2-3 competing buyers
2	A small number of coordinating buyers
1	There is one buyer

EXAMPLES:
- In the artisanal fisheries of Bangladesh ,all landings are sold through a display auction with an auctioneer from the community and the scorer had precise data on the number of buyers (from 20 to 200 per fishery) so each fishery scored a 5 with a quality score of A.
- In the Indonesian artisanal tuna fishery, there are 11-14 middlemen but harvesters are bound by strong familial ties and customs that dictate that they only sell to a particular middleman, so there is effectively one buyer for each harvester and therefore the fishery scored a 1.

Post-Harvest

Markets & Market Institutions

Degree of Vertical Integration

Rationale:

Vertical integration facilitates the flow of information from the retailer to the harvest sector and tends to reduce transaction costs between market levels. As Clark and Munro (1980) argue, the degree of competition between the harvest and post-harvest sectors has important implications for the performance of the fishery.

Metric:

Proportion of harvest where the primary harvester and primary processor/distributor are same firm. The role of vertical integration here is to ensure harvest and delivery of fish under a common management, increasing efficiency and reducing transactions costs.

5	Virtually all
4	70-95%
3	35-70%
2	5-35%
1	Virtually none

Examples:

- In the Alaska pollock fishery, the majority of landings come from vessels that are either outright owned by processing companies or tied to processors through extensive contracts. The reason for the second arrangement is that foreign processing companies are prohibited from majority ownership in vessels. It was estimated that these landings are 70-95% of the total, so the fishery scored a 4.
- In the Lake Victoria Nile perch fishery, processors of only up to 35% of landings can afford to have a boat and hire a crew, making processing and harvesting fall within the same economic entity (here firms and individual owners are equivalent), so the fishery scored a 2.
- In the artisanal fisheries of Bangladesh, all fish buying and processing operations are separate from the harvest sector and the same businesses do not get involved in both, so the fisheries each scored a 1.

POST-HARVEST

MARKETS & MARKET INSTITUTIONS

Level of Tariffs

RATIONALE:

Lower tariffs broaden the market, improve price discovery, and increase the opportunity to create wealth. As Hannesson (2001) argues, the impact of decreasing tariffs and liberalizing the fisheries trade will largely depend on the management systems that are in place; in open access fisheries there is a long run threat to stocks in exporting countries whereas catch control or effectively managed fisheries are predicted to see gains from trade without stocks being threatened.

METRIC:

Official tariff rates charged for exports or imports to consumption markets.

5	Virtually none
4	0.5%-2.5%
3	2.5-5%
2	5%-10%
1	Over 10%

EXAMPLES:

- In the Alaska salmon fishery, there are no export or import tariffs charged between the US and the primary consumption markets in Japan and the US, so the score was a 5.
- The primary consumption market, the US, charges a 15% import tariff on Indonesian blue crab so the score for this fishery was a 1.

POST-HARVEST
MARKETS & MARKET INSTITUTIONS

Level of Non-tariff Barriers

RATIONALE:
Lower non-tariff barriers broaden the market, improve price discovery, and increase the opportunity to create wealth. As Stone (1997) argues, non-tariff barriers such as subsidies to the fishing sector increase overcapacity and generate inefficiencies by encouraging harvest in overexploited fisheries.

METRIC:
Nontariff barriers include: quantity restrictions (import quotas), regulatory restrictions, investment restrictions, customs restrictions and direct government intervention:

5	Are not used to limit international trade
4	Have very limited impact on international trade
3	Act to impede some international trade
2	Act to impede a majority of potential international trade
1	Act to effectively impede a significant amount of international trade

EXAMPLES:
- In the Indonesian blue crab fishery, the major trade restriction is import tariffs but there are also regulatory compliance issues that have a minor effect on trade so the fishery scored a 4.
- The artisanal fisheries of Bangladesh have significant barriers to trade resulting from restrictions to the processing sector, including investment zoning, health restrictions and heavy fees. This represents a major impediment to international trade so the fisheries each scored a 2.

POST-HARVEST

INFRASTRUCTURE

International Shipping Service

RATIONALE:
In order to have access to a broader market, competitively priced international shipping is essential. Some claim that improved access to higher value markets will increase pressure on the resource in the open access context (Liese et al., 2007) while others argue that there are large economic returns from increased international trade and few detrimental side effects on stocks in the short run (Schmitt and Kramer, 2009).

METRIC:
The quality of the service available to access global high value markets, such as the US or EU (regardless of whether product is currently exported and which service is currently used). Average of the two metrics below:

5	Ocean shipping services are readily available at lower than average rates
4	Ocean shipping services are readily available at average rates
3	Ocean shipping services are readily available at higher than average rates
2	Ocean shipping services are available but irregular
1	International shipping is not available at reasonable rates

5	Air shipping services are readily available at lower than average rates
4	Air shipping services are readily available at average rates
3	Air shipping services are readily available at higher than average rates
2	Air shipping services are available but irregular
1	International shipping is not available at reasonable rates

EXAMPLES:
- Although the Alaska salmon fishery is remote, the value of the resource and the sophistication of infrastructure arrangements ensures that both air and ocean shipping services are available at average rates so the fishery scored a 4.
- The Mexican shellfish fisheries of La Paz are also remote, and although there is an airport and a port nearby, there are very few freighters or cargo planes that arrive so the service was classified as irregular and the fishery scored a 2.

POST-HARVEST

INFRASTRUCTURE

Road Quality Index

RATIONALE:
The quality of roads is directly related to the ability of firms to distribute their products, minimize transportation cost and create wealth. There are competing theories about what improvements to road quality will ultimately mean for fish stocks. Some claim that improved access to higher value markets will increase pressure on the resource in the open access context (Liese et al., 2007) while others argue that there are large economic returns from increased international trade and few detrimental side effects on stocks in the short run (Schmitt and Kramer, 2009).

METRIC:
Travel time-weighted average road quality between the fishery's primary port and the most practical export shipping port for exported product. For non-exported product measure road quality between the primary port and the major consumption center. Score according to:

5	High-quality paved roads and extensive highways
4	Primarily paved two-lane roads and moderate highway
3	Primarily paved two-lane roads and minimal highway
2	Paved two-lane roads and well-graded gravel roads
1	Poorly maintained gravel or dirt roads

SCORING GUIDANCE:
It may be that value is not maximized by accessing the current primary market, but road quality prevents accessing the higher-value market.

This is an example of a 5 (paved and high speed):

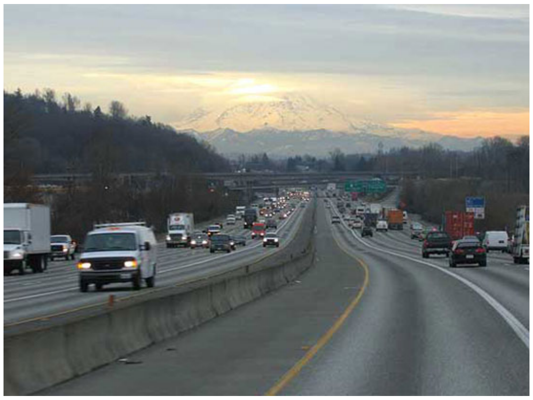

This is an example of a 1 (pitted and slow):

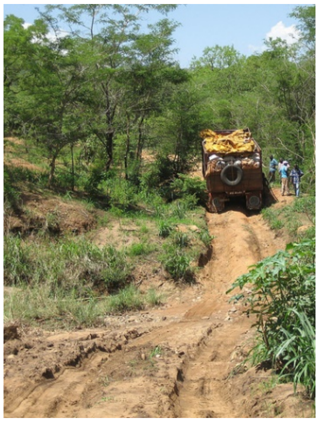

POST-HARVEST

INFRASTRUCTURE

Technology Adoption

RATIONALE:

The availability of the latest communication, processing and production technology is important for firms to maintain global competitiveness and create wealth. As Jensen (2007) demonstrated in the context of South Indian fisheries, improvements in cell phone technology led to a more efficient price discovery system and improved consumer and producer welfare. Others would argue that subsidies to technology will tend to increase overcapacity and overexploitation of the resource (Pauly et al., 2002).

METRIC:

Average level of technology employed in the fishery:

5	Cell phones/fish finders/computers/processing/production technology are readily available
4	Cell phones/fish finders, etc. are common, but some other technology is not always available
3	Cell phones/fish finders, etc. are common, but some other technology is difficult to obtain
2	Cell phones are common, but most other technology is prohibitive
1	Very little advanced technology is accessible for the industry

EXAMPLES:

- Although the Alaska pollock fishery is remote, the harvest and post-harvest sectors employ the most sophisticated technology available including cutting edge processing plants and fish finders so the fishery scored a 5.
- In the artisanal fisheries of Ghana, almost all harvesters have cell phones but any other technology is prohibitively expensive and the fishery scored a 2.

POST-HARVEST

INFRASTRUCTURE

Extension Service

RATIONALE:

Extension services are successful in many countries for transferring technology and information about best management practices, new technology, market conditions and regulatory changes. This information is often essential in a widely dispersed industry to help maximize returns and generate wealth. In the context of agriculture, there is some disagreement about whether the diffusion of information from extension services spreads easily among neighbors (Ryan and Gross, 1943) or whether the benefits from extension services accrue unequally and tend to benefit those least in need of assistance (Goss, 1979).

METRIC:

Degree to which government or NGOs help harvesters improve fishing techniques or management through extension activities.

5	Broad extension service with field offices and close linkage with research community
4	Extension service with moderate field coverage and adequate linkage with the research community
3	Extension service, but with weak links to the research community
2	Minimal, poorly supported extension service
1	No extension service

EXAMPLES:

- In the Alaska halibut fishery, there are multiple field offices for NMFS employees and they spend lots of time working with stakeholders on a broad array of issues from community impacts to biological phenomena. They also have close ties to international academic researchers so the fishery scored a 5.
- In the artisanal fisheries of Bangladesh, consultants from the national department of fisheries are widely available and although their focus is pretty narrow (stocking) they have linkages to the international aquaculture research community so the fishery scored a 4.
- In the Indonesian blue crab fishery, there is little interaction between stakeholders and researchers from either the government or NGOs and there are definitely no field offices so the fishery scored a 1.

POST-HARVEST

INFRASTRUCTURE

Reliability of Utilities/Electricity

RATIONALE:

Reliable utilities are essential for firms to function efficiently and generate wealth. As with roads, there are competing theories about what improvements to utilities will ultimately mean for fish stocks. Some claim that improved access to higher value markets will increase pressure on the resource in the open access context (Liese et al., 2007) while others that there are large economic returns from increased international trade and few detrimental side effects on stocks in the short run (Schmitt and Kramer, 2009).

METRIC:

5 Reliable electrical grid provides power in sufficient quantity to prevent product loss
4 Processors rely on grid, but maintain backup generators
3 Supply chains rely on own generation capacity
2 Supply chain sometimes loses product due to condition or irregular fuel supply for generators
1 Reliable generators or fuel supply not available

SCORING GUIDANCE:

This is a metric where people without a global context have a hard time conceptualizing more reliable electricity. It is important to ask about backup plans and recent losses in product value.

EXAMPLES:

- The Alaska pollock fishery is remote and processing plants are located in the Aleutian islands where electricity is not always reliable; they have very sophisticated backup generators to ensure that this does not cause production delays so the fishery scores a 4.
- Processing for the Pacific groundfish fishery occurs in locations closer to urban centers, so the electricity is very reliable and power outages occur only once or twice a year and do not last more than a couple hours so the score is a 5.
- The artisanal fishery in Kenya relies on generators because the regular electricity is unreliable. Many processors cannot afford generators and others have issues with fuel supply and generators breaking down. These issues sometimes cause processors to lose product and forces processors to smoke or dry fish before it can rot so the fishery scored a 2.

Post-Harvest

Infrastructure

Access to Ice & Refrigeration

Rationale:

Ice or refrigeration is essential for quality control and broadening the market. It has been demonstrated that without access to refrigeration technologies fishery products rapidly deteriorate; 30% of landed fish in the US is lost to microbial activity (Kantor et al., 1997). Under such pressures, there is less flexibility along the supply chain with regards to market access and timing of delivery when ice is not available (Shawyer and Pizzali, 2003).

Metric:

5 Ice is available in various forms and in sufficient capacity to support fresh icing of all fish that needs to be iced
4 Ice is available in various forms, but quantity limits prevent applying to entire catch throughout supply chain
3 Ice is available in limited form and quantity, and thus applied only to most valuable portions of catch
2 Ice is available but capacity constrained; ice often reused, or used through melting stage
1 Ice quantities are extremely limited

Examples:

- The Norwegian cod fishery has access to ice at all points in the supply chain so the fishery scored a 5.
- Within the artisanal fisheries of Bangladesh, some fish are shipped on refrigerated trucks and there is crushed ice in grocery stores, but in open markets there is only block ice and ice is often reused as there are capacity constraints. These fisheries scored a 2.

REFERENCES

Adelaja, A., Menzo, J., McCay, B., 1998. Market Power, Industrial Organization and Tradeable Quotas. Review of Industrial Organization 13, 589–601. doi:10.1023/A:1007799229046

Agrawal, A. (2002). Common Resources and Institutional Sustainability. In National Research Council (Ed.), *The Drama of the Commons*. Washington, DC: The National Academies Press.

Allison EH, Ellis F (2001) The livelihoods approach and management of small-scale fisheries. Mar Policy 25:377-388

Anderson, J.L. 2007. Sustainable Aquaculture: What Does it Mean and How Do We Get There? *Species & System Selection for Sustainable Aquaculture*. PingSun Leung, Cheng-Sheng Lee, and Patricia J. O'Bryen, eds., pp. 9-17. Ames, IA and Oxford, UK: Blackwell Publishing.

Anderson, J.L. 2002. Aquaculture and the Future: Why Economists Should Care. *Marine Resource Economics* 17(2):133-151.

Arnason, R., 2007. Fisheries Self-management under ITQs. Marine Resource Economics 22.

Arnason, R., 2009. Conflicting uses of marine resources: can ITQs promote an efficient solution? Australian Journal of Agricultural & Resource Economics 53, 145–174.

Asche, F., Hansen, H., Tveteras, R., Tveteras, S., 2010. The salmon disease crisis in Chile. Marine Resource Economics 24.

Athukorala, P., Resosudarmo, B.P., 2005. The Indian Ocean Tsunami: Economic Impact, Disaster Management, and Lessons. Asian Economic Papers 4, 1–39.

Behringer, D.C., Butler, M.J., Moss, J., Shields, J.D., 2012. PaV1 infection in the Florida spiny lobster (Panulirus argus) fishery and its effects on trap function and disease transmission. Can. J. Fish. Aquat. Sci. 69, 136–144.

Bennett, E., Neiland, A., Anang, E., Bannerman, P., Atiq Rahman, A., Huq, S., Bhuiya, S., Day, M., Fulford-Gardiner, M., Clerveaux, W., 2001. Towards a better understanding of conflict management in tropical fisheries: evidence from Ghana, Bangladesh and the Caribbean. Marine Policy 25, 365–376.

Boyce, J.R., 1992. Individual transferable quotas and production externalities in a fishery. Natural Resource Modeling 6, 385–408.

Branch, T.A., 2009. How do individual transferable quotas affect marine ecosystems? Fish and Fisheries 10, 39–57.

Cinner, J.E., McClanahan, T.R., MacNeil, M.A., Graham, N.A., Daw, T.M., Mukminin, A., Feary, D.A., Rabearisoa, A.L., Wamukota, A., Jiddawi, N., 2012. Comanagement of coral reef social-ecological systems. Proceedings of the National Academy of Sciences 109, 5219–5222.

Clark, C.W., Munro, G.R., 1980. Fisheries and the processing sector: some implications for management policy. The Bell Journal of Economics 603–616.

Clark, C.W., Munro, G.R., Sumaila, U.R., 2005. Subsidies, buybacks, and sustainable fisheries. Journal of Environmental Economics and Management 50, 47–58. doi:10.1016/j.jeem.2004.11.002

Cohen, M.J., 1995. Technological Disasters and Natural Resource Damage Assessment: An Evaluation of the Exxon Valdez Oil Spill. Land Economics 71, 65–82.

Copes, P., 1986. A critical review of the individual quota as a device in fisheries management. Land economics 62, 278–291.

Costello, C. et al. Status and solutions for the world's unassessed fisheries. Science 338,517–520 (2012)

Cunningham, S., A. Neiland, M. Arbuckle and T. Bostock. 2009. Wealth-based Fisheries Management: Using Fisheries Wealth to Orchestrate Sound Fisheries Policy in Practice. *Marine Resource Economics* 24: 271-87.

Daan, N., 1997. TAC management in North Sea flatfish fisheries. Journal of Sea Research 37, 321–341.

Diamond, N.K., Squillante, L., Hale, L.Z., 2003. Cross currents: navigating gender and population linkages for integrated coastal management. Marine Policy 27, 325–331. doi:10.1016/S0308-597X(03)00044-7

Dobson, A.P., May, R.M., 1987. The effects of parasites on fish populations—theoretical aspects. International Journal for Parasitology 17, 363–370. doi:10.1016/0020-7519(87)90111-1

The Economist. 2009. Grabbing it All. January 3-9: 13-15.

Edinger, E.N., Jompa, J., Limmon, G.V., Widjatmoko, W., Risk, M.J., 1998. Reef degradation and coral biodiversity in indonesia: Effects of land-based pollution, destructive fishing practices and changes over time. Marine Pollution Bulletin 36, 617–630.

Edwards, S.F., 1999. Influence of a Century of Macroeconomic Change on U.S. Fisheries Production. North American Journal of Fisheries Management 19, 1–17.

Essington, Timothy E. "Ecological indicators display reduced variation in North American catch share fisheries." *Proceedings of the National Academy of Sciences* 107, no. 2 (2010): 754-759.

Essington, T.E., Melnychuk, M.C., Branch, T. a., Heppell, S.S., Jensen, O.P., Link, J.S., Martell, S.J.D., Parma, A.M., Pope, J.G., Smith, A.D.M., 2012. Catch shares, fisheries, and ecological stewardship: a comparative analysis of resource responses to a rights-based policy instrument. Conservation Letters 5, 186–195.

Esty, D.C., C. Kim, T. Srebotnjak, M.A. Levy, A. de Sherbinin, and V. Mara. 2008. *2008 Environmental Performance Index*. New Haven, CT: Yale Center for Environmental Law and Policy and New York, NY: Columbia University.

FAO. *Code of Conduct for Responsible Fisheries*. Rome, FAO. 1995. 41 p.

FAO. *Guidelines for the Ecolabelling of Fish and Fishery Products from Marine Capture Fisheries*. Revision 1. Rome, FAO. 2009.

Geheb, K., Kalloch, S., Medard, M., Nyapendi, A.-T., Lwenya, C., Kyangwa, M., 2008. Nile perch and the hungry of Lake Victoria: Gender, status and food in an East African fishery. Food Policy 33, 85–98.

Goss, K. F. (1979). Consequences of diffusion of innovations to rural population. *Rural Sociology* 44:754-772.

Gutiérrez, N.L., Hilborn, R., Defeo, O., 2011. Leadership, social capital and incentives promote successful fisheries. Nature 470, 386–389.

Hanna, S.S., 1995. User participation and fishery management performance within the pacific fishery management council. Ocean & Coastal Management, Property Rights and Fisheries Management 28, 23–44.

Hannesson, R., 2001. Effects of liberalizing trade in fish, fishing services and investment in fishing vessels. OECD Papers 1.

Hapke, H.M., 2001. Petty Traders, Gender, and Development in a South Indian Fishery*. Economic geography 77, 225–249.

Hilborn, R., 2007. Moving to Sustainability by Learning from Successful Fisheries. AMBIO: A Journal of the Human Environment 36, 296–303.

Hilborn, R., Branch, T.A., Ernst, B., Magnusson, A., Minte-Vera, C.V., Scheuerell, M.D., Valero, J.L., 2003. State of the world's fisheries. Annual review of Environment and Resources 28, 359.

Hilborn, R., Stokes, K., Maguire, J.-J., Smith, T., Botsford, L.W., Mangel, M., Orensanz, J., Parma, A., Rice, J., Bell, J., Cochrane, K.L., Garcia, S., Hall, S.J., Kirkwood, G.P., Sainsbury, K., Stefansson, G., Walters, C., 2004. When can marine reserves improve fisheries management? Ocean & Coastal Management 47, 197–205.

Holland, D.S., 2003. Integrating spatial management measures into traditional fishery management systems: the case of the Georges Bank multispecies groundfish fishery. ICES J. Mar. Sci. 60, 915–929.

Jensen, R., 2007. The Digital Provide: Information (Technology), Market Performance, and Welfare in the South Indian Fisheries Sector. The Quarterly Journal of Economics 122, 879–924.

Jentoft, S., 1985. Models of fishery development: The cooperative approach. Marine Policy 9, 322–331.

Jentoft, S., McCay, B., 1995. User participation in fisheries management: lessons drawn from international experiences. Marine Policy 19, 227–246.

Jentoft, S., McCay, B.J., Wilson, D.C., 1998. Social theory and fisheries co-management. Marine Policy 22, 423–436.

Johannes, R.E., 1998. The case for data-less marine resource management: examples from tropical nearshore fin fisheries. Trends in Ecology & Evolution 13, 243–246.

Kaplan, I.M., McCay, B.J., 2004. Cooperative research, co-management and the social dimension of fisheries science and management. Marine Policy 28, 257–258.

Kaplan, I.M., 2000. Seafood auctions, market equity and the buying and selling of fish: lessons on co-management from New England and the Spanish Mediterranean. Marine Policy 24, 165–177.

Kaufman, D., A. Kraay, and M. Mastruzzi. 2008. *Governance Matter VII: Aggregate and Individual Governance Indicators 1996-2007*. Washington, DC: The World Bank.

Knapp, G. 2002. Challenges and Strategies for the Alaska Salmon Industries. ISER, University of Alaska, Anchorage.

Liese, C., Smith, M.D., Kramer, R.A., 2007. Open access in a spatially delineated artisanal fishery: the case of Minahasa, Indonesia. Environment and Development Economics 12, 123–143.

Maitland, P.S., 1995. The conservation of freshwater fish: Past and present experience. Biological Conservation 72, 259–270.

Marine Stewardship Council. 2009. <http://www.msc.org/documents/msc-standards/MSC_environmental_standard_for_sustainable_fishing.pdf>

McCay, B.J., Jentoft, S., 1996. From the bottom up: Participatory issues in fisheries management. Society & Natural Resources 9, 237–250.

Medard, M., Sobo, F., Ngatunga, T., Chirwa, S., 2001. Women and gender participation in the fisheries sector in Lake Victoria, in: Global Symposium on Women in Fisheries, Kaohsiung, Taiwan.

Miller, T. and K. Holmes. 2009. *2009 Index of Economic Freedom*. The Heritage Foundation/*Wall Street Journal* and <http://www.heritage.org/Index/pdf/Index09_Methodology.pdf>.

Moloney, D.G., Pearse, P.H., 1979. Quantitative rights as an instrument for regulating commercial fisheries. Journal of the Fisheries Board of Canada 36, 859–866.

Munro, G.R., 2004. The conservation and management of shared fish stocks: legal and economic aspects. FAO.

Nowlis, J., Van Benthem, A.A., 2012. Do Property Rights Lead to Sustainable Catch Increases? Marine Resource Economics 27, 89–105.

Ostrom, E., 1990. Governing the commons: The evolution of institutions for collective action. Cambridge university press.

Ostrom E, 2009. A general framework for analyzing sustainability of social-ecological systems. Science (New York, N.Y.) 325, 419–22.

Pauly, D., Christensen, V., Guénette, S., Pitcher, T.J., Sumaila, U.R., Walters, C.J., Watson, R., Zeller, D.,
2002. Towards sustainability in world fisheries. Nature 418, 689–695.

Peterson, C.H., Rice, S.D., Short, J.W., Esler, D., Bodkin, J.L., Ballachey, B.E., Irons, D.B., 2003. Long-term ecosystem response to the Exxon Valdez oil spill. Science 302, 2082–2086.

Pomeroy, R.S., Berkes, F., 1997. Two to tango: The role of government in fisheries co-management. Marine Policy 21, 465–480.

Ryan, B., & Gross, N. C. (1943). The diffusion of hybrid seed corn in two Iowa communities. Rural Sociology 8:15-24

Sanchirico, J.N., Wilen, J.E., 2005. Optimal spatial management of renewable resources: matching policy scope to ecosystem scale. Journal of Environmental Economics and Management 50, 23–46.

Schmitt K.M, Kramer D.B, 2009. Road development and market access on Nicaragua's Atlantic coast: Implications for household fishing and farming practices. Environ. Conserv. Environmental Conservation 36, 289–300.

Shahidul Islam, M., Tanaka, M., 2004. Impacts of pollution on coastal and marine ecosystems including coastal and marine fisheries and approach for management: a review and synthesis. Marine Pollution Bulletin 48, 624–649.

Shimshack, J.P., Ward, M.B., Beatty, T.K., 2007. Mercury advisories: information, education, and fish consumption. Journal of Environmental Economics and Management 53, 158–179.

Stone, C.D., 1997. Too Many Fishing Boats, Too Few Fish: Can Trade Laws Trim Subsidies and Restore the Balance in Global Fisheries. Ecology LQ 24, 505.

Sumaila, U.R., Guénette, S., Alder, J., Chuenpagdee, R., 2000. Addressing ecosystem effects of fishing using marine protected areas. ICES Journal of Marine Science: Journal du Conseil 57, 752–760.

Sutinen, J.G., Andersen, P., 1985. The Economics of Fisheries Law Enforcement. Land Economics 61, 387.

Sutinen, J.G., Gauvin, J.R., Gordon, D.V., 1989. An Econometric Study of Regulatory Enforcement and Compliance in the Commercial Inshore Lobster Fishery of Massachusetts, in: Neher, P.A., Arnason, R., Mollett, N. (Eds.), Rights Based Fishing, NATO ASI Series. Springer Netherlands, pp. 415–431.

The Fish Stock Sustainability Index. 2009. <http://www.whitehouse.gov/omb/expectmore/detail/10000036.2007.html>.

The World Bank. 2009a. *Doing Business 2010: Reforming through Difficult Times*. Palgrave Macmillan.

The World Bank. 2009b. *World Development Indicators* <http://web.worldbank.org/WBSITE/EXTERNAL/DATASTATISTICS/0,,contentMDK:20535285~menuPK:1192694~pagePK:64133150~piPK:64133175~theSitePK:239419,00.html>.

The World Bank. 2008. *The Sunken Billions: The Economic Justification for Fisheries Reform.* Agriculture and Rural Development. Washington, DC.

Transparency International. 2009. *Corruption Perceptions Index 2009* <http://www.transparency.org/.>

Walker, J.M., Gardner, R., Ostrom, E., 1990. Rent dissipation in a limited-access common-pool resource: Experimental evidence. Journal of Environmental Economics and Management 19, 203–211.

Walters, C., 2000. Impacts of dispersal, ecological interactions, and fishing effort dynamics on efficacy of marine protected areas: how large should protected areas be? Bulletin of marine science 66, 745–757.

Walters, C. and R. Hilborn. 1976. Adaptive control of fishing systems. J. Fish. Res. Board Canada 33:145–159.

WHO. The World Health Report 2000. Geneva: WHO, 2000.

Wilen, J.E., Botsford, L.W., 2005. Spatial management of fisheries.

Wilen, J.E., 1979. Fisherman Behavior and the Design of Efficient Fisheries Regulation Programs. J. Fish. Res. Bd. Can. 36, 855–858. doi:10.1139/f79-123

Zeller, D., Froese, R., Pauly, D., 2005. On losing and recovering fisheries and marine science data. Marine Policy 29, 69–73. doi:10.1016/j.marpol.2004.02.003
